# ‘Conjugate’ coseismic surface faulting related with the 29 December 2020, Mw 6.4, Petrinja earthquake (Sisak-Moslavina, Croatia)

**DOI:** 10.1038/s41598-021-88378-2

**Published:** 2021-04-28

**Authors:** Emanuele Tondi, Anna Maria Blumetti, Mišo Čičak, Pio Di Manna, Paolo Galli, Chiara Invernizzi, Stefano Mazzoli, Luigi Piccardi, Giorgio Valentini, Eutizio Vittori, Tiziano Volatili

**Affiliations:** 1 School of Science and Technology, Geology Division, University of Camerino, Camerino, Italy; 2 Italian Institute for Environmental Protection and Research - Geological Survey of Italy, Roma, Italy; 3 Water Management Department for Middle and Lower Sava Flood Protection Service, Croatian Waters, Slavonski Brod, Croatia; 4 Civil Protection Department, Presidency of the Council of Ministers, Roma, Italy; 5 Institute of Geosciences and Earth Resources, National Research Council, Firenze, Italy; 6 National Institute of Geophysics and Volcanology, Roma, Italy

**Keywords:** Structural geology, Natural hazards, Geodynamics, Seismology

## Abstract

We provide here a first-hand description of the coseismic surface effects caused by the Mw 6.4 Petrinja earthquake that hit central Croatia on 29 December 2020. This was one of the strongest seismic events that occurred in Croatia in the last two centuries. Field surveys in the epicentral area allowed us to observe and map primary coseismic effects, including geometry and kinematics of surface faulting, as well as secondary effects, such as liquefaction, sinkholes and landslides. The resulting dataset consists of homogeneous georeferenced records identifying 222 observation points, each of which contains a minimum of 5 to a maximum of 14 numeric and string fields of relevant information. The earthquake caused surface faulting defining a typical ‘conjugate’ fault pattern characterized by Y and X shears, tension cracks (T fractures), and compression structures (P shears) within a ca. 10 km wide (across strike), NW–SE striking right-lateral strike-slip shear zone (i.e., the Petrinja Fault Zone, PFZ). We believe that the results of the field survey provide fundamental information to improve the interpretation of seismological, GPS and InSAR data of this earthquake. Moreover, the data related to the surface faulting may impact future studies focused on earthquake processes in active strike-slip settings, integrating the estimates of slip amount and distribution in assessing the hazard associated with capable transcurrent faults.

## Introduction

**Figure 1:**
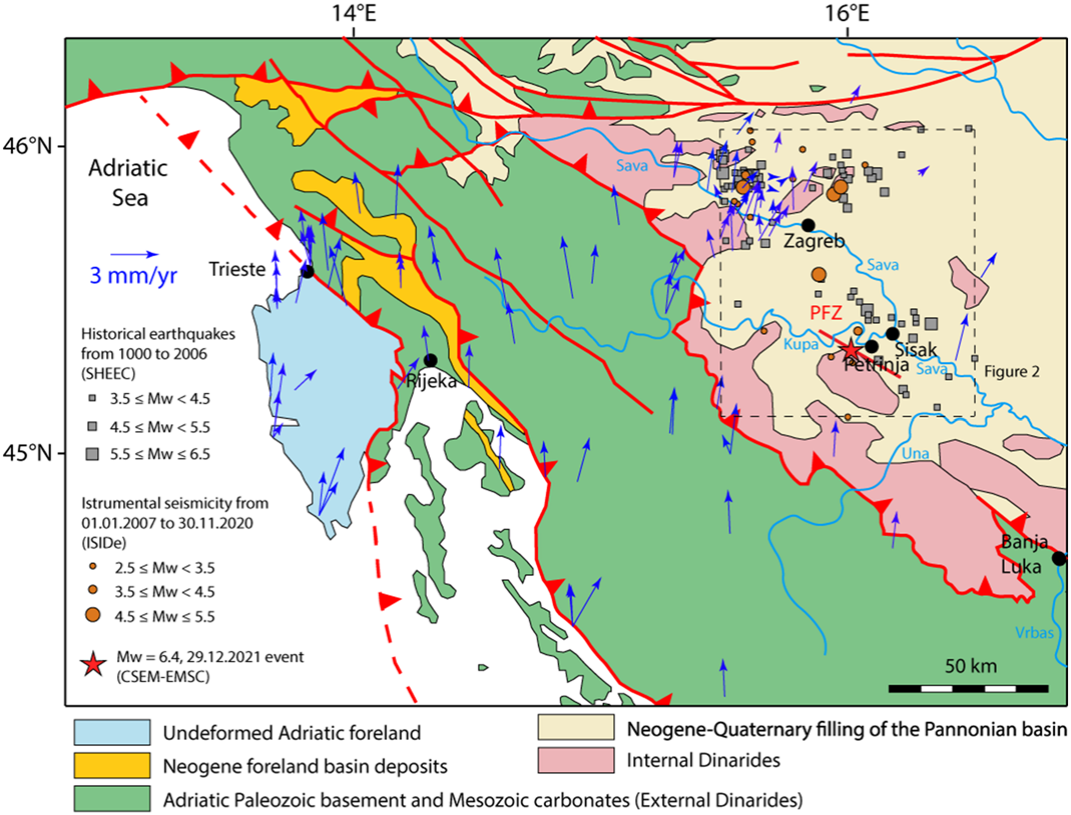
Geodynamic setting of the study area within the Dinarides-Pannonian Basin framework (modified after ^1^). The location of the main fault belonging to the Petrinja Fault Zone is marked as PFZ. Blue arrows display horizontal vector motion of permanent Global Navigation Satellite System (GNSS) stations in the “European fixed” reference frame ^3^. Dashed box shows the location of Fig. 2 with epicentres of historical earthquakes from 1000 to 2006 (grey squares) selected from ^2^ and ^4^ and instrumental seismicity from 01.01.2007 to 30.11.2020 selected from ^5^. The epicentre of the Mw 6.4 event of 29.12.2020 is shown for reference (red star).

**Figure 2:**
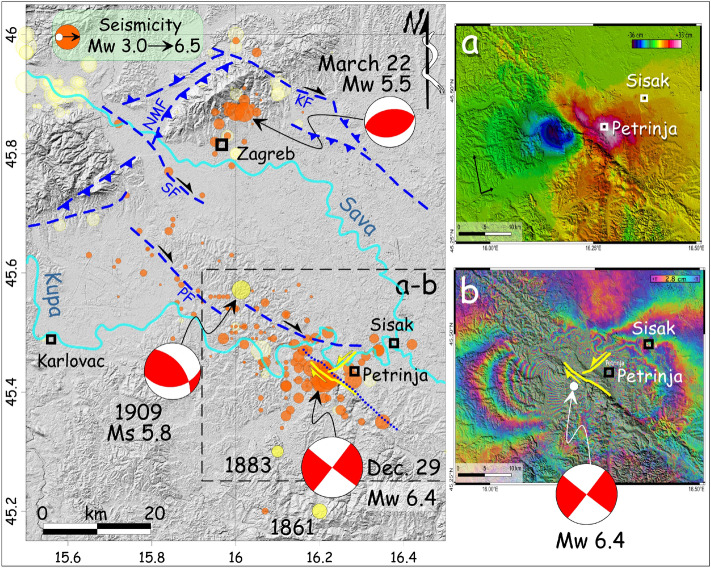
Shaded relief map (from ASTER GDEM data, https://asterweb.jpl.nasa.gov/gdem.asp) of the area affected by the 2020 seismic sequence (orange epicenters, EMSC data; www.emsc-csem.org). Yellow epicenters, historical seismicity^4^. Blue dashed lines, main Quaternary active faults (mod. from^1^); NMF, north Medvednica; SF, Sava; KF, Kasina. PF, Pokuplje fault^20^. Blue dotted line, buried fault in the 1: 100,000 scale Official Geological Map of Croatia^29^. Yellow lines, coseismic surface faulting related to the 29 December 2020 Petrinja earthquake. The focal mechanism of the Kupa Valley earthquake in 1909 is from^20^. Those of 2020 are from^18^. Panel (**a**–**b**) is an interferometric imagery (Sentinel-1A Ascending orbit, 20,201,224-20,201,230, https://scihub.copernicus.eu/dhus/#/home) showing surface motion associated with the 29 December 2020 Mw 6.4 earthquake. In (**b**) each fringe represents a shift of approximately 2.8 cm along the satellite's line of sight (LOS), which is oriented from WSW to ENE; above: actual displacement in cm along the LOS, in blue are the areas with a component of motion toward the satellite (WSW-ward) and in red areas that have moved away. This implies a right-lateral slip in the order of several tens of cm. Interferometry analysis from SNAP tool by ESA v.8.0.0 (https://step.esa.int/main/toolboxes/snap/). The figure was generated by QGIS v. 3.10.2 (https://qgis.org/).

On 29 December 2020 at 11:19 (UTC), a moment magnitude (Mw) 6.4 earthquake struck central Croatia near the city of Petrinja, a settlement of about 25,000 inhabitants in the region of Sisak-Moslavina, causing 7 casualties and thousands homeless. The epicentre was 15 km SW of Sisak, the main town of the region, and 45 km SSE of Zagreb (Figs. 1, 2;^1–5^). The mainshock was preceded the day before by two foreshocks at 05:28 and 06:49 (UTC time), with Mw 5.2 and 4.8, respectively.

The seismic sequence came nine months after the Zagreb earthquake, a mainshock of Mw 5.5 that was followed by a largest aftershock of Mw 4.9, both occurred on March 22, between 4 and 7 km north-northwest of Zagreb^6^ (Figs. 1, 2). In the last two centuries the same area was affected by three moderate earthquakes: on December 18, 1861 (Mw 5.4), on February 11, 1883 (Mw 5.1), and on October 8, 1909 (Mw 5.7)^2^ (Fig. 2). The Mw 6.4 Petrinja earthquake occurred at the boundary of the two main geological provinces of Croatia: the Dinarides mountain belt and the Pannonian Basin (Fig. 1;^7,8^). The Dinarides, a wide NW–SE striking fold-and-thrust belt stretching from southwestern Slovenia to Montenegro along the Adriatic coast of Croatia and inland, are the result of the Alpine collision between the Eurasian and Adriatic tectonic plates (e.g.,^9–12^). The most prominent structures of the Dinarides are NW–SE trending folds and thrusts exposed along the SW margin of the Pannonian Basin, a wide depression located in the interior of the arcuate Carpathian mountain chain. The latter joins the Alps to the west and the Dinarides to the southwest. The Croatian sector of the Pannonian Basin is limited to the southwest by the Sava sub-basin, a NW–SE oriented tectonic depression showing an asymmetrical shape, with a gentle slope to the SW and a steep NE flank^13^. Here, the post-rift sediments are represented by the lower Pannonian (i.e., lower Tortonian) marly limestones, upper Pannonian (i.e., upper Tortonian) and lower Pontian (i.e., lower Messinian) turbidite sandstones as well as upper Pontian and Pliocene deltaic and alluvial depositional systems^14,15^. All these sedimentary units have been deformed by strike-slip tectonics and are characterized by positive flower structures that are nowadays still active and seismogenic^16,17^. This geodynamic framework originated from the cessation of normal faulting in the Pannonian Basin and the ongoing counter clockwise rotation of the Adriatic microplate around a pole located in the Western Alps. This dynamic resulted in inversion tectonics dominated by thrusting and strike-slip faulting along the basin margins (e.g.,^18,19^). The Petrinja earthquake reflects this geodynamic setting, as the focal mechanism suggests a roughly N-S, horizontal maximum compression (Fig. 2). The fault plane solution includes nearly vertical southeast and southwest striking nodal planes. In the Sava sub-basin, active strike-slip fault systems including both NW–SE and NE-SW oriented faults are reported in the literature^1,7^.

Thanks to the flying of the Sentinel 1A radar satellite of ESA (European Space Agency) over the area struck by the earthquake already on December 30 (ascending orbit 146), it was possible to compute an InSAR imaging of the ground deformation caused by the earthquake very soon after the event, allowing to constrain the region where ground effects were mostly to be expected. The scenes utilized for the interferogram formation shown in Fig. 2a,b were acquired on December 18 (slave) and 30 (master). The processing was carried out with the SNAP toolbox of ESA and the SNAPHU software for phase unwrapping. The observed deformation field in the line of sight (LOS) direction shows on the NW side a maximum shortening of the distance from the satellite of ca. 36 cm, and a lengthening of the same distance reaching a maximum of ca. 33 cm centred in the area of Petrinja (east of the ruptured fault). The deformation pattern imaged by the InSAR, considered the ENE-oriented direction of recording, is interpretable as a right-lateral, north-westward slip of the region west of the Petrinja Fault Zone. The fringe geometry has served here basically to direct our field observations toward the areas of highest linear deformation, where tectonic ground ruptures were most likely to be present, and subsequently to compare the InSAR-imaged deformed region with the distribution and size of geological coseismic effects observed in the field.

As observations of coseismic surface effects are of considerable scientific importance, it is necessary to carry out the surveys as soon as possible. As a matter of fact, surface effects may be erased by degradation of fault scarps or by road/infrastructure repair, as well as overprinted by postseismic afterslip^21–23^. The engineers of Croatian Water Management Department started the survey of surface effects immediately after the main shock, while a working group represented by eight researchers of different Italian institutions (University of Camerino, ISPRA, CNR, DPC) began surveying the ground coseismic effects on 10 January 2021, working 8 hours a day per person for the following 8 days. In this report (Map and Dataset) we present earthquake surface ruptures along a ca. 10 km wide (across strike), right-lateral strike-slip shear zone that we term Petrinja Fault Zone (PFZ). Mapping was carried out using both field observations and aerial surveys using a drone and relative photogrammetry elaborations. By integrating our observations with available seismological and geodetic data, we also provide an interpretation and a discussion of the fault ruptures associated with the Mw 6.4 mainshock.

## Methods and data records

The description of surface coseismic effects is very important in earthquake geology, as it provides a unique opportunity to observe short-term time scale deformation. These observations allow a much more robust interpretation of the long-term time scale geological features for seismic and surface faulting hazard evaluation purposes. Data collected in our survey may contribute to update and integrate the worldwide database aimed at assessing fault displacement hazard^24^. Furthermore, the geometry, kinematics, and amount of displacement of fault ruptures propagated from depth during an earthquake constrain the modelling of seismic sources based on inversion of geophysical datasets (e.g., strong motion recordings, GPS time-series and InSAR images).

An Unmanned Aerial Vehicle (UAV) was used as a complementary tool of the traditional field work. We performed aerial Structure from Motion (SfM) photogrammetry, collecting large numbers of overlapping photos to construct 3D, digital, virtual outcrop models (VOMs;^25–27^) using Agisoft Metashape software, following the workflow outlined by^28^. Digital geologic interpretations and structural data extraction were made using the Virtual Reality Geologic Studio software (VRGS).

**Figure 3:**
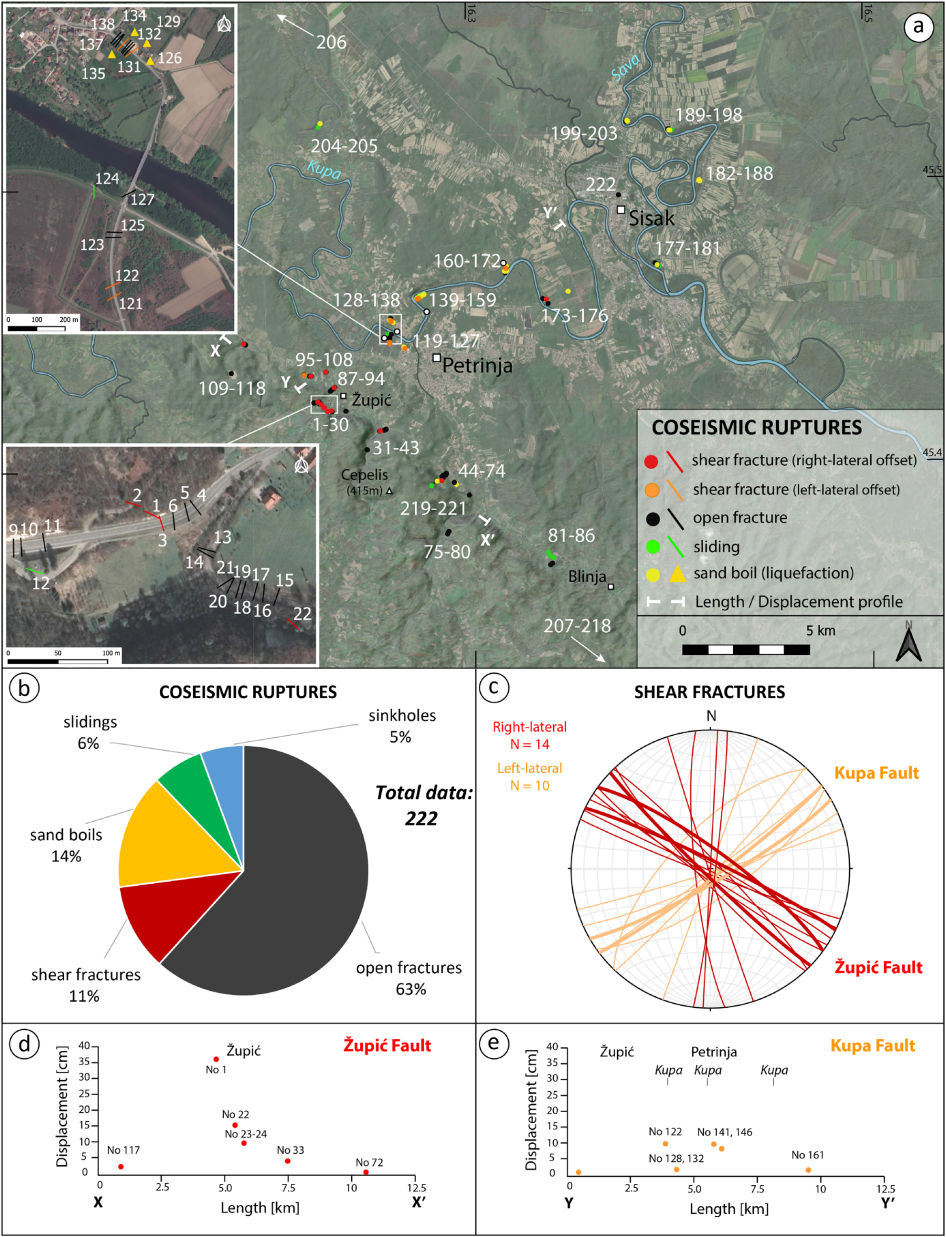
(**a**) Map of surface ruptures measured in the epicentral area of the Petrinja earthquake. Numbers refer to the “No” of records included in the database. (**b**) Pie diagram showing the relative proportion of each main type of measured coseismic ruptures. (**c**) Stereographic plot (lower hemisphere, equal-area projection) of shear fractures; thicker great circles represent right- and left-lateral planes with largest strike-slip offset. Below are displacement/length profiles for the Župić (**d**) and Kupa (**e**) Faults. The map was generated by QGIS v. 3.10.2 (https://qgis.org/); satellite image from Google Earth Pro, v. 7.3.3 (https://www.google.com/earth/); the stereographic projection in (**c**) was created with Stereonet v. 10.4.6 (http://www.geo.cornell.edu/geology/faculty/RWA/programs/stereonet.html).

Our surveying, which has led to the recognition and mapping in the epicentral area of the most significant surface ruptures, their geometry, kinematics, and associated displacement, is summarized in a concise dataset (Table 1) and map (Fig. 3).

**Table 1:** Examples of records extracted from the dataset.

No	Date	Lat	Long	Obs	Sub	Strike [deg]	Dip dir [deg]	Dip [deg]	Len [m]	Ope [cm]	Off [cm]	Rak [deg]	Vec [deg]
1	14/01/21	45.4241	16.2226	Coseismic shear fracture	Soil	296	26	85	60	5	36	10	297/10
2	14/01/21	45.4241	16.2225	Coseismic shear fracture	Soil	290	36	85	8	4	36		
3	11/01/21	45.424	16.2226	Coseismic shear fracture	Road	162	252	89	15	8	10		
4	11/01/21	45.4241	16.2231	Coseismic open fracture	Road	323			15				
5	11/01/21	45.4241	16.223	Coseismic open fracture	Road	346			15

The dataset presented in the Supplementary Material is a text file consisting of 222 records organized into 14 fields. Each record describes a single observation point. The fields have a name and a short name, and are described as follows:

1. NUMBER (short name: No);
2. DATE (short name: Date);
3. LATITUDE (short name: Lat);
4. LONGITUDE (short name: Long);
5. OBSERVATION (short name: Obs): five categories are defined: “Coseismic shear fracture” (ground break displaying a perceivable shear offset of the ground surface, i.e. > 1 cm); “Coseismic open fracture” (ground break with no perceivable shear offset, i.e. <  < 1 cm); “Coseismic sliding” (generic landslide of ascertained coseismic origin); “Coseismic sand boil” (sand volcanism phenomena related to liquefaction induced by the earthquake); “Sinkhole” (ground collapse caused by the earthquake);
6. TYPE OF SUBSTRATUM (short name: Sub): nature of the substratum where the coseismic effect was observed;
7. STRIKE (short name: Strike);
8. DIP DIRECTION (short name: Dip dir);
9. DIP ANGLE (short name: Dip);
10. LENGTH (short name: Len): length measured in meters of a rupture or sliding surface;
11. OPENING (short name: Ope): aperture of a rupture or sliding surface measured in centimetres, orthogonal to the fracture walls;
12. OFFSET (short name: Off): net displacement of a coseismic rupture measured in centimetres;
13. RAKE (short name: Rak): the angle of the slip lineation on the fault plane measured in degrees (in the 0°-180° range);
14. VECTOR (short name: Vec): the trend (range 0°–360°) and plunge (range 0°–90°) of the slip lineation in degrees, measured with respect to the North and the horizontal, respectively.

## Surface rupture description

**Figure 4:**
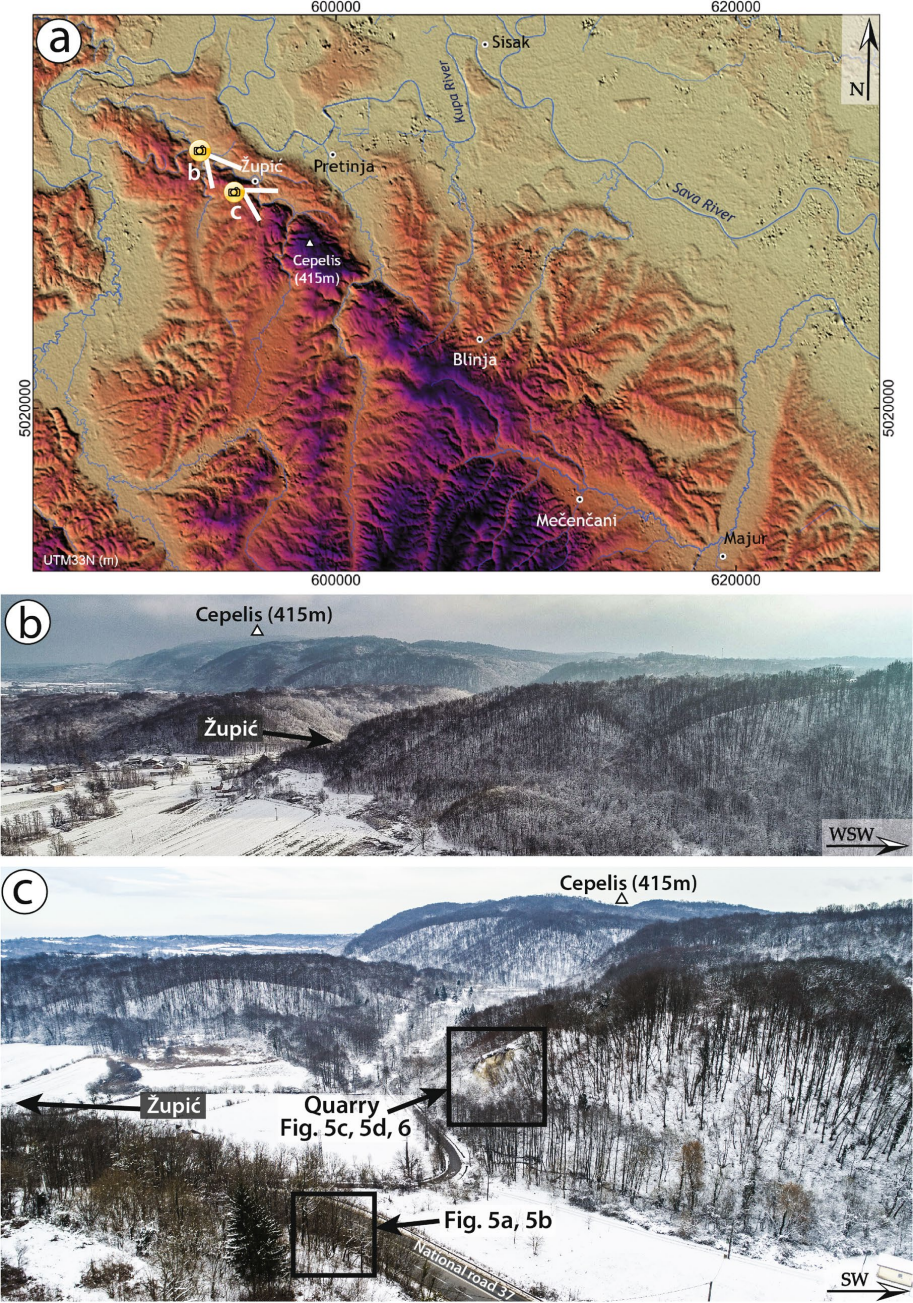
(**a**) Digital Elevation Model (EU-DEM v1.1, 25 m resolution, https://land.copernicus.eu/imagery-in-situ/eu-dem/eu-dem-v1.1) of the epicentral area of the 29 December 2020 Petrinja earthquake. The direction of the panoramic views of (**b**) and (**c**) are also indicated; (**b**) Panoramic view of the Župić area showing a portion of the ridge NW of Župić and the south-eastern ridge in the background (photo acquisition date 12/01/2021); (**c**) Panoramic view of the Župić south-eastern ridge area showing the locations of Figs. 5 and 6 (photo acquisition date 14/01/2021).

The major, long-term, morphotectonic feature in the epicentral area of the Petrinja earthquake is the elongated NW–SE trending ridge that develops between the Kupa river, to the north, and the village of Blinja, to the south, for a length of about 30 km (Fig. 4). This ridge culminates at about mid-length with the Cepelis Peak (415 m a.s.l.) and is affected by rivers deeply carved into the southern, uplifted block. Streams flowing across the ridge reaching their outwash at the foot of its NW flank appear to be locally diverted, changing their direction from orthogonal to parallel to the western slope or even dammed by fault activity.

The 29 December mainshock, whose epicentre was close to the town of Petrinja (Fig. 2), produced surface coseismic effects mostly distributed in the area between Petrinja and Sisak (Fig. 3). The coseismic effects consisted of primary surface ruptures, that are those directly related to the slip along the earthquake fault, and other coseismic effects induced by ground shaking. The latter have left an overall modest signature in the landscape, both permanent (e.g. landslides, sinkholes) and ephemeral (sand boil associated with liquefaction phenomena). Notably, we also observed several coseismic effects aligned along a NE-SW oriented fault that is marked as buried in the geological map at scale 1:100,000^29^.

### Primary effects

The pattern of primary surface ruptures depicts a fault system including two sets of coseismic shear fractures (Fig. 2). The NW–SE-trending shear fractures (Fig. 3c) are characterized by right-lateral strike-slip offset reaching a maximum value of 36 cm (Fig. 3d). On the other hand, the NE-SW-trending shear fractures (Fig. 3c) are characterized by left-lateral strike-slip offsets of up to 10 cm (Fig. 3e).

**Figure 5:**
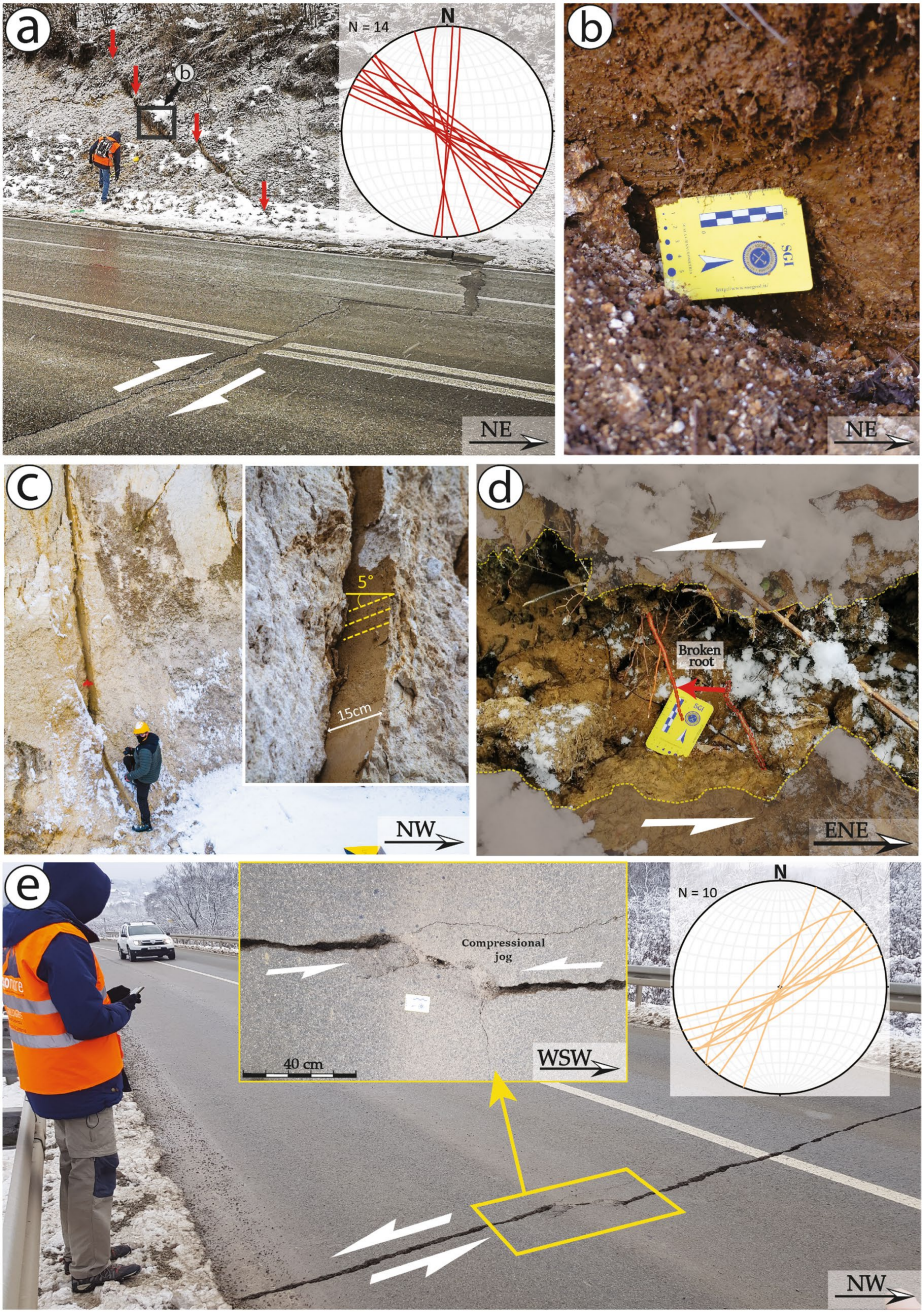
Examples of NW–SE to N-S oriented right-lateral strike-slip surface faulting (**a**–**c**) and NE-SW to E-W oriented left-lateral strike-slip surface faulting (**d**–**e**): (**a**) coseismic rupture with right-lateral offset near the village of Župić (No 1, 14/01/2021) and lower-hemisphere, equal-area projection of the overall coseismic ruptures with right-lateral offset; (**b**) close-up of slickenlines on the fault plane (13/01/2021); (**c**) evidence of right-lateral surface faulting on bedrock (No 22, 11/01/2021), the inset is a close-up of the fault plane showing 15 cm horizontal offset with slickenlines (rake = 5°); (**d**) Broken and displaced root (No 149, 13/01/2021) showing 8 cm left-lateral offset; (**e**) coseismic rupture in paved road near the village of Brest Pokupski (No 121, 12/01/2021) showing 5 cm left-lateral offset and lower-hemisphere, equal-area projection of the overall coseismic ruptures with left-lateral offset. The inset shows a close-up of compressional jog. The numbers (No 1) refer to the observation points (indicated in Fig. 3) of the records in the database, while the dates refer to the photo acquisition.

**Figure 6:**
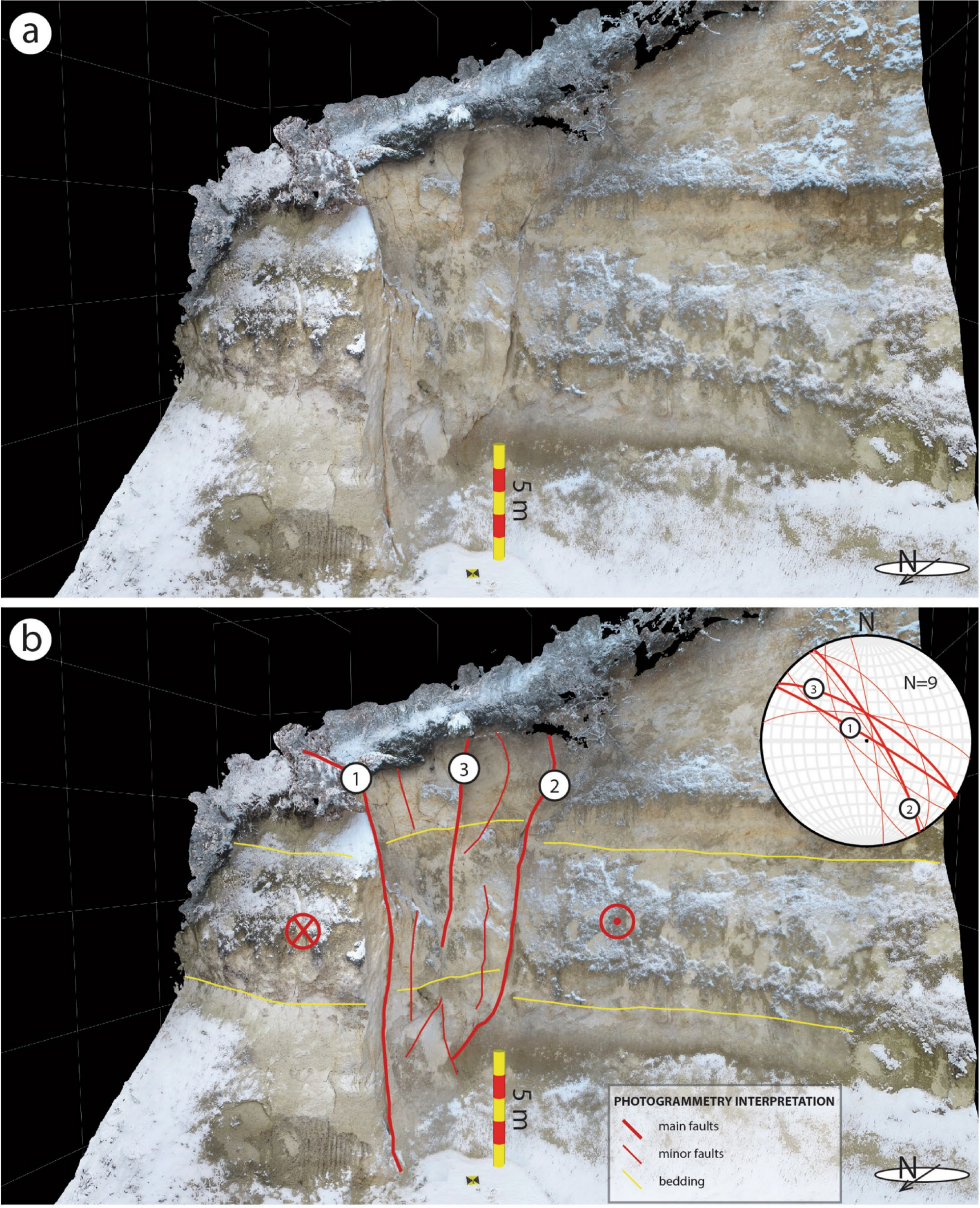
(**a**) Virtual outcrop model (from Agisoft Metashape Professional v. 1.6.2, https://www.agisoft.com/) of the Župić Fault exposed in a quarry in poorly-lithified Pliocene shallow marine calcareous deposits (see also Fig. 5c). (**b**) Geologic interpretation and structural data extracted from the Virtual Reality Geologic Studio software (VRGS v. 2.42, https://www.vrgeoscience.com/), depicts a positive flower structure composed of three main fault planes.

Southwest to Petrinja, an almost continuous NW–SE pattern of primary coseismic surface ruptures was observed for an end-to-end extent of about 15 km along a pre-existing fault zone, here named the Župić Fault (Fig. 3a,c,d). Along this trend, in the vicinity of the village of Župić, we mapped more than 2 km of almost continuous coseismic surface rupture, characterised by > 20 cm mean right-lateral horizontal offset. The local largest offset, up to 36 cm, was observed along the national road 37. Here the coseismic reactivation of the fault produced a right-lateral offset of the roadside scarp surface along a 120°N striking, steeply NE dipping fault plane (observation point No. 1 in Figs. 3a, 5a,b). The rupture could be followed across the road, where it attained an approximate N-S strike (i.e., roughly perpendicular to the road direction, which most probably controlled the rupture propagation in the asphalt); we observed a right-lateral offset of 10 cm, accompanied by an opening of 8 cm. The rupture joined a fault plane in a quarry located ca. 180 m to the SE, which was reactivated as well (observation point No. 22 in Figs. 3, 5c), where poorly lithified Pliocene shallow marine calcareous deposits outcrop, showing a right-lateral offset of ∼15 cm along a 134°N striking, sub-vertical fault plane (Fig. 6a,b). Shear fractures associated with this fault were observed also to the NW (observation points No. 109-118 in Fig. 3) and SE of it (observation points No. 31-43 and 44-74 in Fig. 3), characterized by a right-lateral offset in the range of 2 to 4 cm and accompanied by several open fractures having approximately the same orientation. At these observation points, as well as at the observation points No. 81-86 (Fig. 3), the lack of markers on the road made it difficult or impossible to measure any strike-slip offset. In some cases, it was possible to measure the shear offset by observing the geometry of the fracture and the relative extensional and compressional jogs (see the supplementary photo archive included in the database for the extensive documentation on the observed shear and open fractures). Other minor shear fractures with a right-lateral offset of 1 to 2 cm were seen across a road immediately to the NE of the Župić Fault (observation points 87-94 and 95-108). Moreover, 10 km NE of the Župić fault (observation points No. 173-176), a ∼300 m long right-lateral shear zone was identified in the alluvial plain of the Kupa river. This shear zone was characterized by en echelon open fractures with intervening mole tracks (see Fig. 8 and photos included in the database).

Between Petrinja and Sisak, along the alluvial plain of the Kupa river, a NE-SW pattern of primary surface ruptures was also mapped for an end-to-end extent of about 8 km. This feature is here named Kupa Fault (Fig. 5d,e). It is important to note that these coseismic effects are aligned along a NE-SW striking fault which is marked as buried in the 1: 100,000 scale Official Geological Map of Croatia^29^.

**Figure 7:**
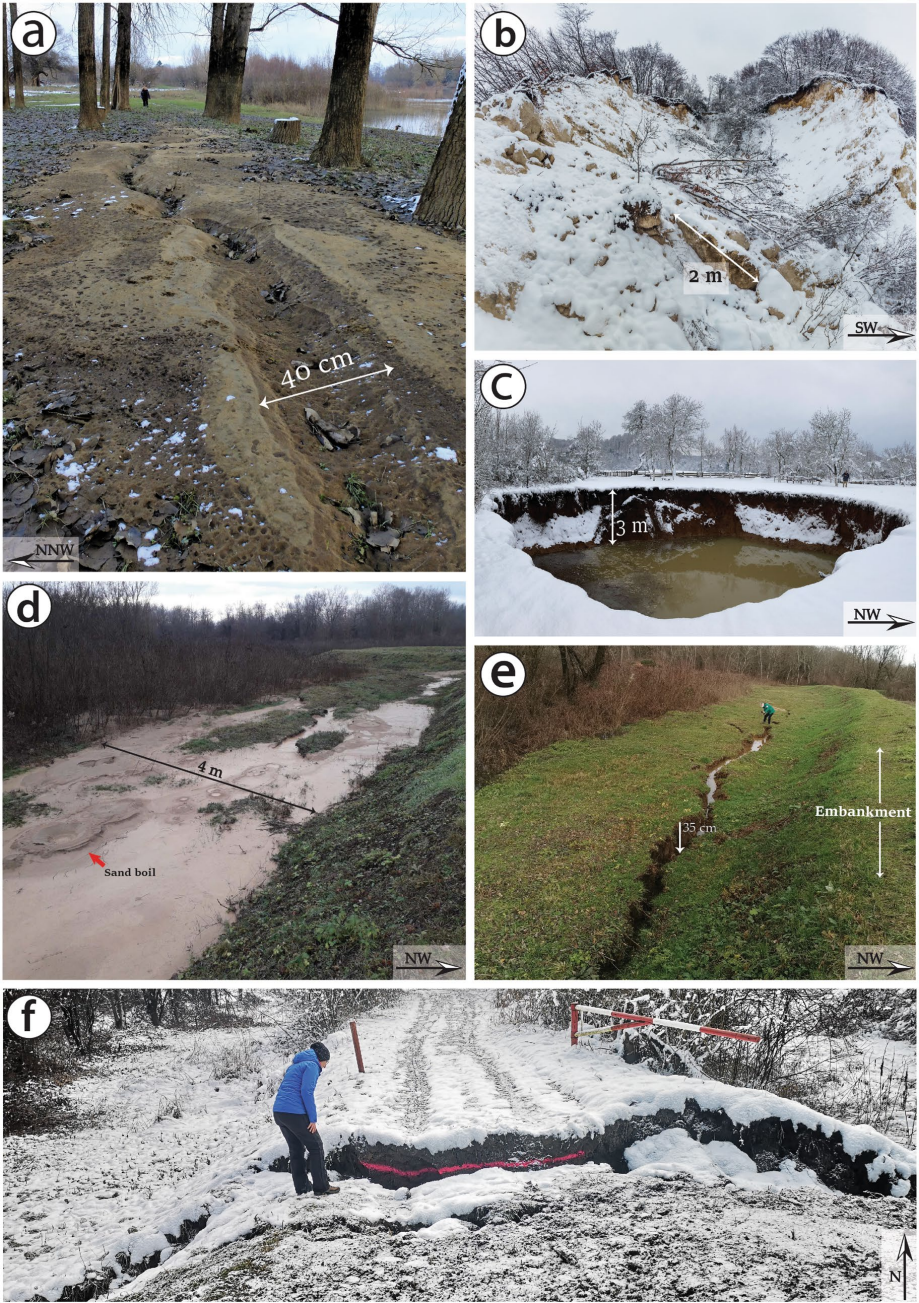
Examples of sliding, rockfall, fracture, sinkhole, and sand volcanism in the epicentral area of the Petrinja earthquake. (**a**) (15/01/2021) and (**d**) (5/01/2021) Examples of sand boil associated with liquefaction phenomena occurred in the valley of the Kupa river (No 165, 166, 205). (**b**) The earthquake triggered some rockfalls along the crown of the quarry close to the village of Hrastovica (No 64, 12/01/2021). (**c**) The largest sinkhole opened in the Mečenčani area with a diameter of about 20 m (No 212, 14/01/2021). (**e**) Large fracture connected to the sinking of the river dam induced by liquefaction below (No 204, 5/01/2021). (**f**) Failure of river embankment induced by liquefaction (No 189, 14/01/2021). The numbers (No 1) refer to the observation points of the records in the dataset indicated in Fig. 3 and the dates refer to the photo acquisition.

The main coseismic surface ruptures along the Kupa Fault consisted of shear fractures displaying > 5 cm left-lateral horizontal offset, observed along the national road 37 (Fig. 5d,e; observation points No. 128-138), as well as shear fractures and en echelon open fractures displaying > 10 cm left-lateral horizontal offset detected along the alluvial plain of the Kupa river (Figs. 5d and 7a,b; observation points No. 139-159). Here, fractures were generally associated with sand boils produced by liquefaction phenomena, which damaged the banks and the dam along the Kupa river (Fig. 7e,f). In this area, the assessment of the amount of the left-lateral offset associated with the NE-SW oriented shear fractures was permitted by the presence of both cut roots and trees located across these fractures (Fig. 5d).

### Secondary effects

Coseismic surface effects related to ground shaking, permanent or ephemeral (e.g., landslides, sinkholes and sand boils), were identified in the epicentral area between Petrinja and Sisak (Fig. 3). In particular, sand liquefaction was widespread along the NE-SW oriented fault zone inside the alluvial plain of the Kupa River (Fig. 7a,d). These phenomena were often clearly associated with surface ruptures represented by shear fractures and en echelon open fractures. Immediately NW of Petrinja, both surface rupture and liquefaction phenomena (i.e., sand boil) had seriously damaged the banks and the dam along the Kupa river (Fig. 7e,f), making it necessary to build an outermost embankment to contain the potential floods.

Landslides mainly matched pre-existing gravitational movements and induced large fractures in the roads. Rockfall and debris fall occurred in quarry crowns and in correspondence of steep scarps (Fig. 7b). The largest one occurred in a quarry close to the Hrastovica village, with a total volume exceeding 70 cubic meters, and single rock blocks up to 2–4 cubic meters. Small landslides were mapped by^30^ on the slope next to the parish church in the village of Viduševac and along the road Kravarsko—D. Hruševac. Minor landsliding was also present along the river embankments as a result of seismic shaking and consequent liquefaction (Fig. 7f). The seismic vibration also induced the compaction of the artificial fillings and embankments, causing the formation of differential settlements and fractures (Fig. 7e).

The very moderate evidence and the limited occurrence of gravitative movements triggered by the earthquake can be related to the low energy of the relief, as the epicentral zone is mainly located in the plain of the Kupa river; furthermore, the mountains south-east of Petrinja are characterized by a smooth morphology, without evident strong changes in slope, except for the escarpment running about NW–SE in the Župić area (Fig. 3).

Collapses and opening of small sinkholes in the ground have been described by the inhabitants in the urban area of Petrinja, but we were unable to document this effect as the holes had been filled with debris soon after their formation. A man (Edison Tomas) now living in Župić told us that some holes had already opened 4–5 h before the main shock in the road close to the house of his daughter in Petrinja with a depth reaching 6 m.

In the Mečenčani area (see Fig. 4), about 20 km southeast of Petrinja, the most impressive effect was represented by the opening of about 30 sinkholes (observation points No 207-218 in Figs. 3, 4). The sinkholes had different dimensions, from one to tens of meters in diameter (Figs. 3, 7c) and were several meters deep. All the sinkholes appeared filled by water up to a depth of about 3 m from the surface, which is the level of the water table in the alluvial deposits. According to the narration of the locals, the collapses occurred after the earthquake, with a delay from a few hours to a few days.

The observation of aerial and satellite images clearly shows that in many cases the areas prone to the sinkhole collapses were already recognizable before the event. Following this approach, many potential sinkholes could be additionally identified by an aerial (drone) survey of the plain in order to identify the sectors of higher hazard. In any case, this preliminary analysis needs to be complemented with geophysical prospecting to complete the mapping of the zones most prone to such highly hazardous phenomenon.

Finally, two likely cases of hydrological anomaly were spotted in the area close to the village of Hrastovica, SE of Petrinja, where a copious flow of water with sand sprung up from the garage of a house and a nearby geyser-similar water fountain with a height of 50–70 cm produced a strong increase of the flow rate of the drainage ditch (observation points No. 219-221 in Fig. 3). Similar geyser-like effects occurred at Brest Popupsky springing from a water well (observation point No. 126 in Fig. 3).

## Discussion

The recognition of coseismic effects in the aftermath of an earthquake is fundamental for individuating primary surface faulting and its structural arrangement. Understanding the relationship between the seismic source at depth and its primary evidence at surface creates the basis for using surface active faults to contribute foreseeing which structure will rupture next. This work also provides new data on surface coseismic faulting in strike-slip domains, which is not a common event in the Alpine-central Mediterranean area.

**Figure 8:**
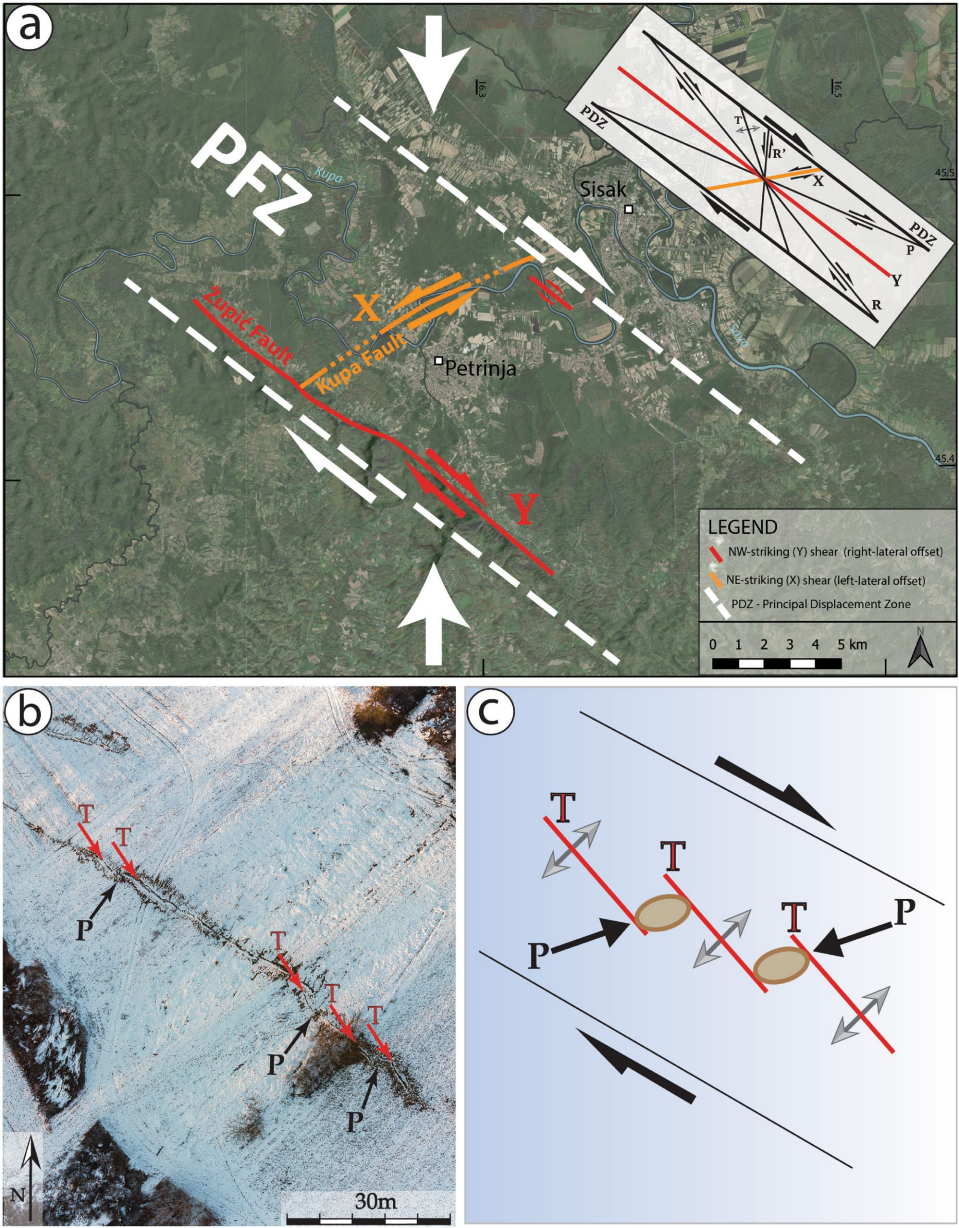
Diagrams showing the main active faults activated during the 29 December 2020 Petrinja earthquake. (**a**) The Petrinja Fault Zone (PFZ) is a ca. 10 km wide right-lateral strike-slip shear zone composed by “conjugate” faults in which the Župić and the Kupa Faults represent (Y) and (X) shears, respectively (satellite image from Google Earth Pro, v. 7.3.3 (https://www.google.com/earth/). The inset shows the structural features of an idealized shear zone. (**b**) Tension cracks (T) and mole tracks (P) are developed coseismically within the NW-striking (Y) and NE-striking (X) shears (observation points No 173-176 in Fig. 3, photo acquisition date 13/01/2021). The NS oriented arrows indicate the horizontal maximum compression based on geological data, geophysical observations ^19^ and the field data obtained in this study. (**c**) Simplified sketch of Fig. 8b.

Coseismic surface faulting of the 2019 earthquake is represented by both aligned and en echelon fault segments, defining the main Župić and Kupa Faults, which display a typical shear zone structural pattern near the town of Petrinja (here named the Petrinja Fault Zone or PFZ). Geometrically, an idealized shear zone consists of six principal elements: R and R’ conjugate shears, T tension fractures, P shears, and X and Y shears, which are all oriented at well-defined angles to the general trend of the shear zone, called the Principal Displacement Zone or PDZ (Fig. 8b). R and R’ shears form a conjugate Riedel shear set^31^. Y and X shears, showing opposite senses of movement, define a ‘conjugate’ set characterised by different angular relationships (i.e., the maximum compression axis is parallel to the bisector of the obtuse angle—rather than the acute angle—between Y and X shears; for this reason, we use the inverted commas for this ‘conjugate’ set, to distinguish it from typical conjugate faults such as R and R’ shears). Previous studies have shown that R and R’ shears, T tension fractures and P shears (mole tracks) may form simultaneously along pre-existing strike-slip faults during large-magnitude earthquakes (e.g.,^32–34^). On the other hand, X shears forming as ‘conjugate’ faults to the Y faults within coseismic surface rupture zones are not well known to date. The coseismic development of X shears has only recently been reported as part of the surface ruptures produced by the 2014 Yutian Mw 6.9 (Tibetan) earthquake^35^; however, their kinematic nature and formation mechanisms remain unclear. Therefore, the coseismic ‘conjugate’ fault system described in this study represents a rare case demonstrating the simultaneous activation of X and Y shear faults during an earthquake. Coseismic surface deformation associated with the Petrinja earthquake also includes en echelon tension cracks (T fractures) and mole tracks (or P shears) associated with strike-slip faulting (Fig. 8a,b). Changes in the orientation of the various structures are a function of the magnitude and localization of the shear strain, reflecting the different stages in the evolution of the strike-slip shear zone (e.g.,^36–39^).

The InSAR imaging of the deformation field (Fig. 1) is in good agreement with the field observations, both in terms of concentration of effects—which are most evident where the InSAR shows the highest displacement—and in terms of type of displacement expected for a dominantly dextral-slip event. The InSAR imaging of the deformation was particularly helpful to locate the fault and assess its sense of displacement. On the other hand, the related offsets could not be accurately quantified by this elaboration. The deformation pattern of Fig. 2A points to a total strike-slip dextral displacement of about 70 cm for the two blocks across the Župić Fault. However, the InSAR-derived displacement evaluated directly on the fault trace is of about 30 cm. This discrepancy is a commonly observed feature^40^, testifying for a near surface distributed deformation that often accommodates a substantial part of the actual fault slip. Our field measurements of right-lateral offset along the Župić Fault, reaching a maximum value of 36 cm (Fig. 3b), are therefore fully consistent with the InSAR data. Moreover, the InSAR-derived deformation pattern clearly defines two domains characterised by a different behaviour within the SE-slipping, northeastern block of the Župić Fault. These two domains are separated by the map trace of the Kupa Fault, this being consistent with its left-lateral coseismic motion (Fig. 3c). Since in general the north–south component of the displacement is not resolved by InSAR due to the orientation of the satellite orbits, left-lateral slip along the Kupa Fault is mainly marked by horizontal components of motion in the E-W direction. Such a motion results in an east-ward displacement of the block east of the fault, and a west-ward motion of the block west of it. While the former adds to the general east-ward movement of the northeastern block of the Župić Fault, the latter subtracts to it, thus consistently explaining the observed displacement field.

Consistently with the focal mechanisms of the December 29 mainshock, and also of the March 22, 2020, and of the 1909 event, our field investigations and the analytic results of coseismic ‘conjugate’ shear structures reveal that the direction of the principal compressive stress is horizontal and roughly N-S trending in the study area. Coseismic surface ruptures occurred along the PFZ, which represents the Principal Displacement Zone or PDZ in Fig. 8b. Accordingly, we suggest that coseismic ‘conjugate’ faulting during the 2020 Petrinja earthquake was mainly controlled by the pre-existing, active strike-slip PFZ within the framework of the present tectonic stress associated with the ongoing motion of Adria with respect to the Eurasian Plate. Roughly N-S convergence across the plate boundary, marked by the NW striking Dinaride chain, is recorded by horizontal vector motion of permanent GNSS stations (Fig. 1). This motion resulted in partitioning of the deformation into dominant thrusting in the Adriatic frontal part of the Dinarides^41^ and belt-parallel dextral strike-slip faulting in its interior (including the area of the present study), as it is typical in regions of oblique plate convergence^42^.

## Conclusions

Following the 29 December 2020, Mw 6.4 Petrinja earthquake, a complex surface faulting pattern was observed and mapped in the field along the causative PFZ. Based on our study of the co-seismic shear structures, we can draw the following conclusions:

1. The co-seismic shear structures were produced by this earthquake along the pre-existing right-lateral strike-slip PFZ, and they are mainly characterized by Y and X shears, tension cracks (T fractures), and mole tracks (P shears).
2. The ‘conjugate’ fault structures comprise two sets of coseismic shears that are striking NW–SE and NE-SW. The NW–SE-trending structure represents a Y shear with right-lateral strike-slip displacement of up to 36 cm, including left-stepping en echelon tension cracks (T) and mole tracks (P). On the other hand, the NE-SW-trending structure represents a X shear with left-lateral displacement of up to 10 cm, including right-stepping en echelon cracks (T) and mole tracks (P), which are concentrated in a zone of < 5 m around individual rupture zones.

Our findings suggest that the coseismic ‘conjugate’ Y and X faulting is mainly controlled by the pre-existing, active PFZ within the framework of the ongoing northward ‘push’ of the Adria Plate along the margins of the Pannonian Basin. The regional geodynamic setting of partitioned transpression results in active thrusting in the outer Dinarides and dominant strike-slip faulting in the interior of the belt, as it occurs in the epicentral area of the 29 December 2020, Mw 6.4, Petrinja earthquake.

The mapped pattern of coseismic fault ruptures is relevant for improving the assessment of the seismic and surface faulting hazard of this region, beside the danger related to landslides, liquefaction and sinkholes. More in general, the prompt, accurate mapping of the coseismic ruptures associated with this moderate magnitude earthquake contributes to improve our understanding of earthquake faulting processes and to better forecast the impact of the more energetic earthquakes expected in the Alpine-Dinarides-Albanides orogen, where the knowledge regarding such phenomena is still modest.

## Supplementary Information

Supplementary Information 1.

Supplementary Information 2.

Supplementary Information 3.

## Data Availability

All data generated or analysed during this study are included in this published article (and its Supplementary Information files).
