# Supplementary material for: ‘Conjugate’ coseismic surface faulting related with the 29 December 2020, Mw 6.4, Petrinja earthquake (Sisak-Moslavina, Croatia)
Source: Sci Rep. 2021 Apr 28;11:9150. doi: 10.1038/s41598-021-88378-2 (PMC8080844; doi:10.1038/s41598-021-88378-2)
Supplement: Supplementary file 2 — Supplementary Information 2. [file 41598_2021_88378_MOESM2_ESM.docx]

**‘CONJUGATE’ COSEISMIC SURFACE FAULTING RELATED WITH THE 29 DECEMBER 2020, MW 6.4, PETRINJA EARTHQUAKE (SISAK-MOSLAVINA, CROATIA)**

Emanuele Tondi^1, 6^, Anna Maria Blumetti^2^, Mišo Čičak^3^, Pio Di Manna^2^, Paolo Galli^4^, Chiara Invernizzi^1^, Stefano Mazzoli^1^, Luigi Piccardi^5^, Giorgio Valentini^1^, Eutizio Vittori^5^, Tiziano Volatili^1^*

^1^ School of Science and Technology, Geology Division, University of Camerino, Italy

^2^ Italian Institute for Environmental Protection and Research - Geological Survey of Italy, Roma, Italy

^3^ Water Management Department for Middle and Lower Sava Flood Protection Service, Croatian Waters

^4^ [Civil Protection Department, Presidency of the Council of Ministers, Roma, Italy](https://www.researchgate.net/false)

^5^ Institute of Geosciences and Earth Resources, National Research Council, Firenze, Italy

^6^ National Institute of Geophysics and Volcanology, Italy

*email: [tiziano.volatili@unicam.it](mailto:tiziano.volatili@unicam.it)

**SUPPLEMENTARY MATERIALS**

1. **Dataset:** The dataset presented in the supplementary materials consists of 222 records organized into 14 fields. For a detailed fields description refer to the main manuscript text.
2. **Supplementary_photo_archive**: photos acquired during the field data acquisition, numbered according to the “No” reported in the Dataset.
