# Supplementary figures and images for: ‘Conjugate’ coseismic surface faulting related with the 29 December 2020, Mw 6.4, Petrinja earthquake (Sisak-Moslavina, Croatia) (part 2 of 2)
Source: Sci Rep. 2021 Apr 28;11:9150. doi: 10.1038/s41598-021-88378-2 (PMC8080844; doi:10.1038/s41598-021-88378-2)

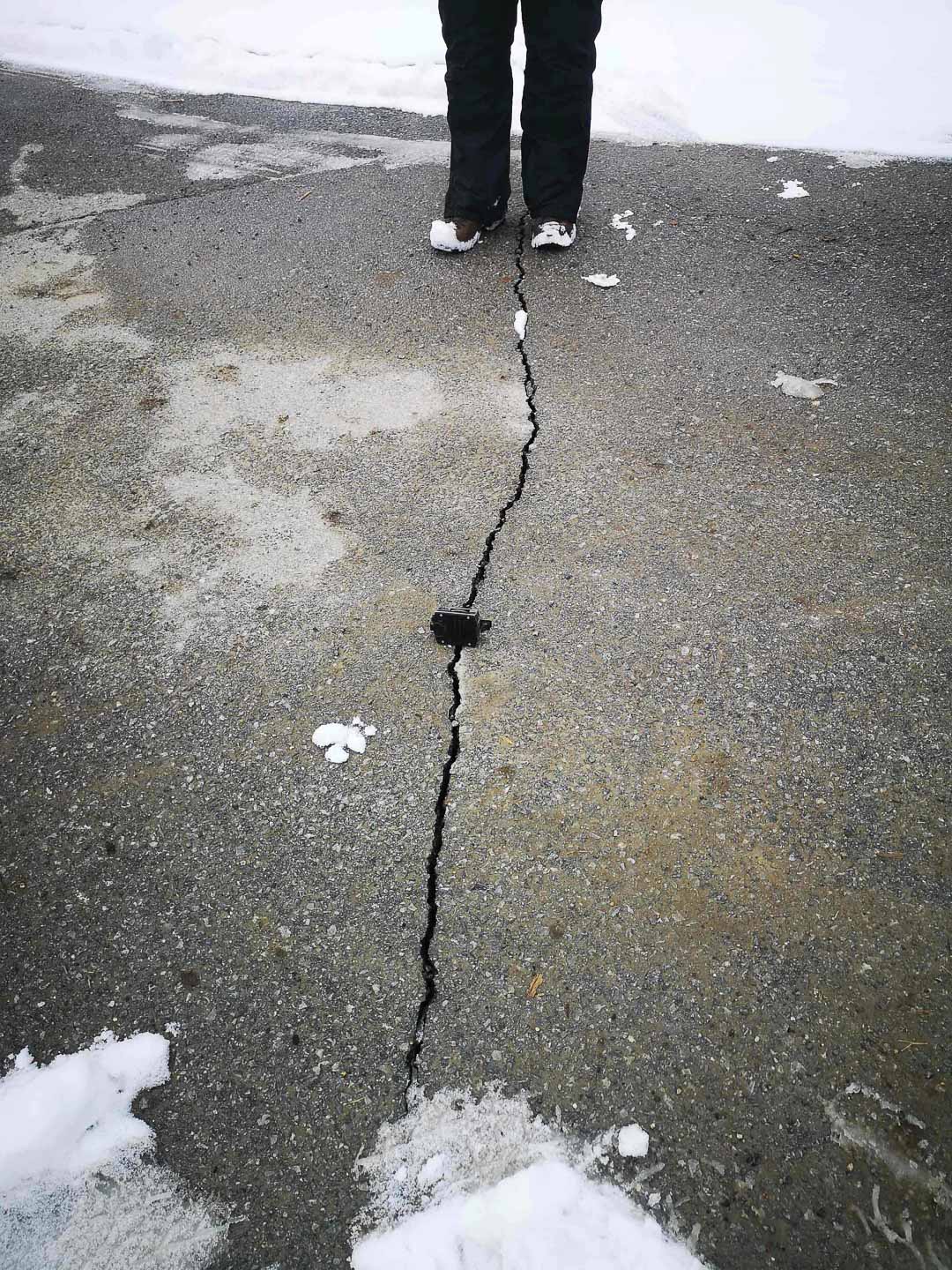

Supplement: Supplementary file 3 — Supplementary Information 3. [file 41598_2021_88378_MOESM3_ESM.zip › 84 (12-01-2021).jpg]

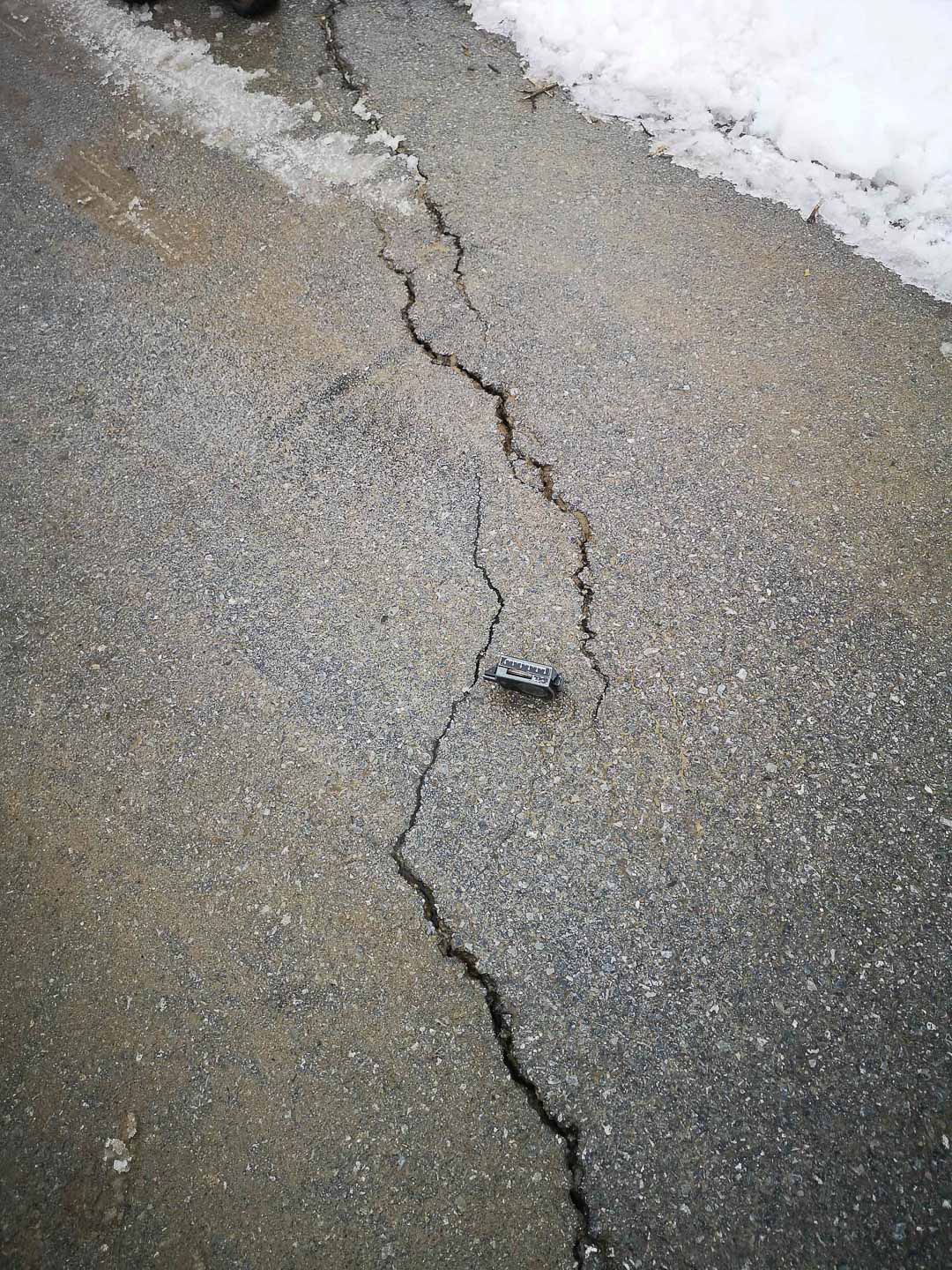

Supplement: Supplementary file 3 — Supplementary Information 3. [file 41598_2021_88378_MOESM3_ESM.zip › 85 (12-01-2021).jpg]

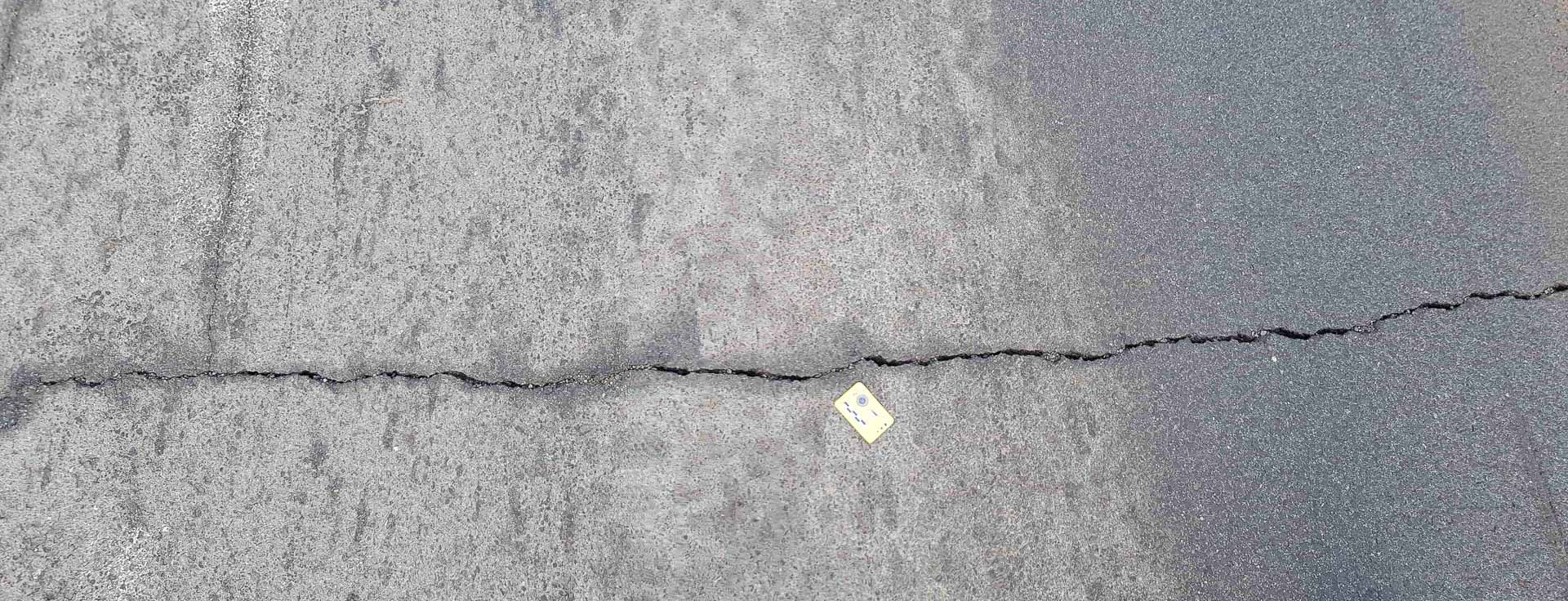

Supplement: Supplementary file 3 — Supplementary Information 3. [file 41598_2021_88378_MOESM3_ESM.zip › 93a (12-01-2021).jpg]

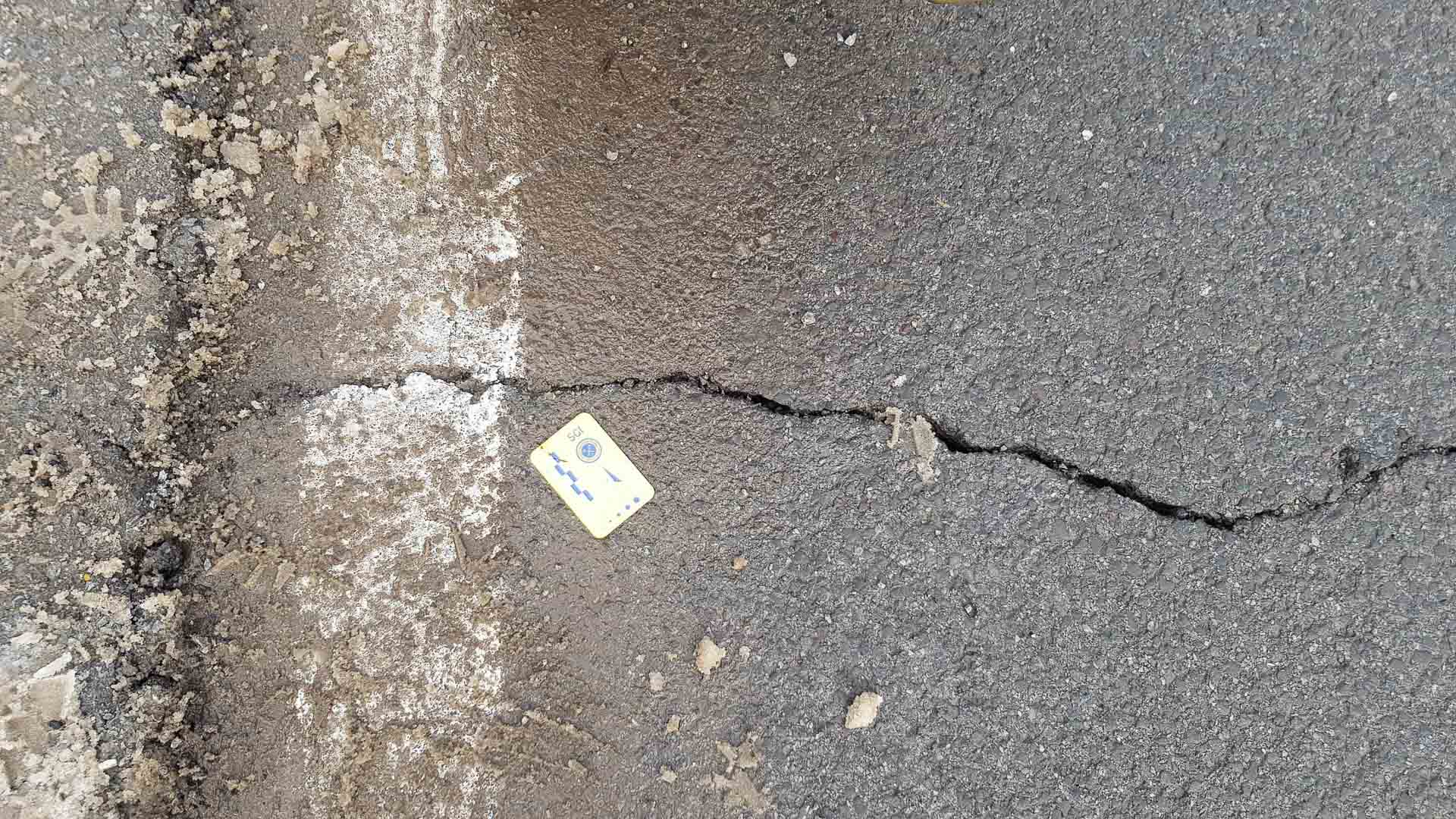

Supplement: Supplementary file 3 — Supplementary Information 3. [file 41598_2021_88378_MOESM3_ESM.zip › 93b (12-01-2021).jpg]

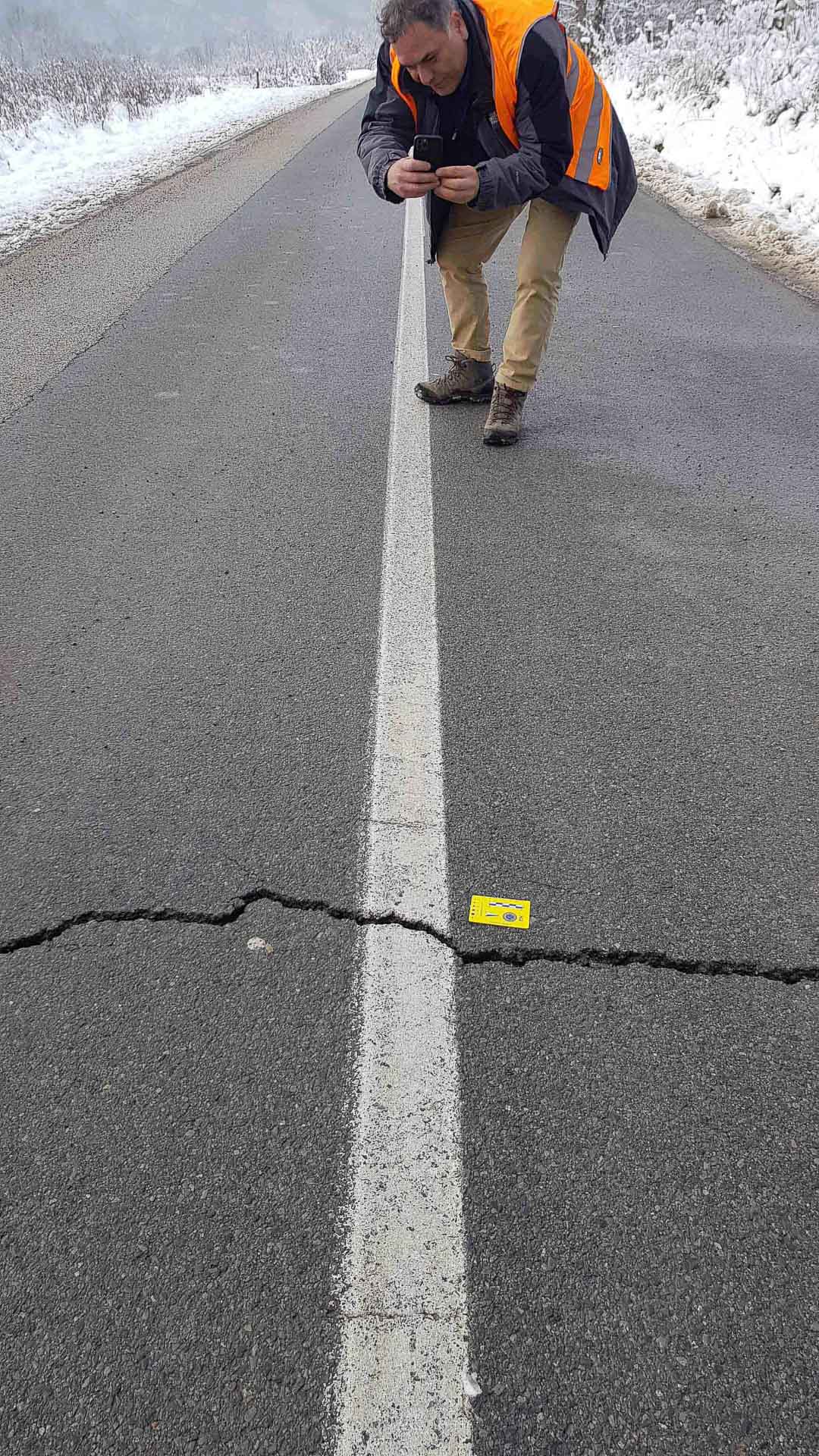

Supplement: Supplementary file 3 — Supplementary Information 3. [file 41598_2021_88378_MOESM3_ESM.zip › 96a (12-01-2021).jpg]

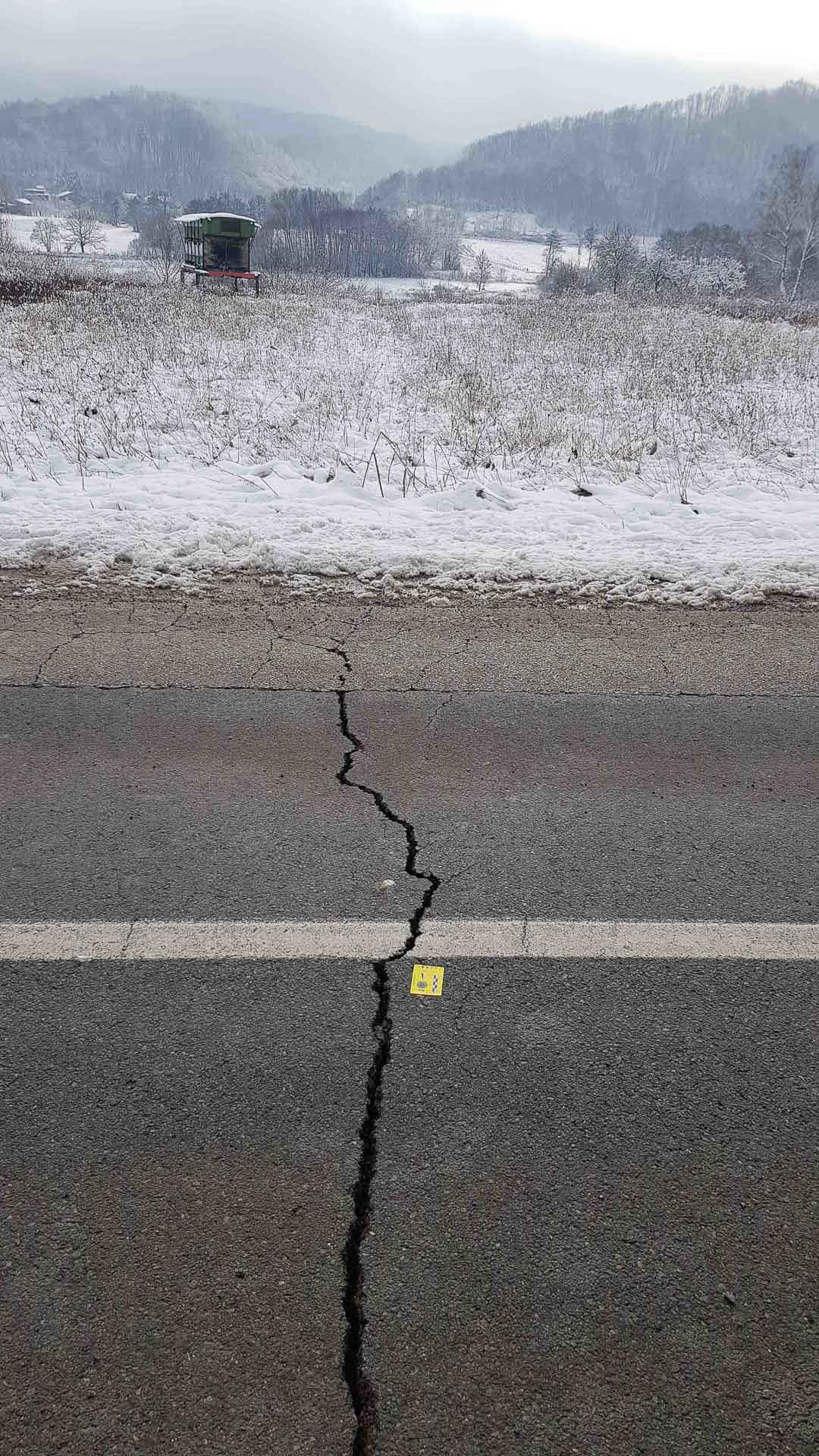

Supplement: Supplementary file 3 — Supplementary Information 3. [file 41598_2021_88378_MOESM3_ESM.zip › 96b (12-01-2021).jpg]

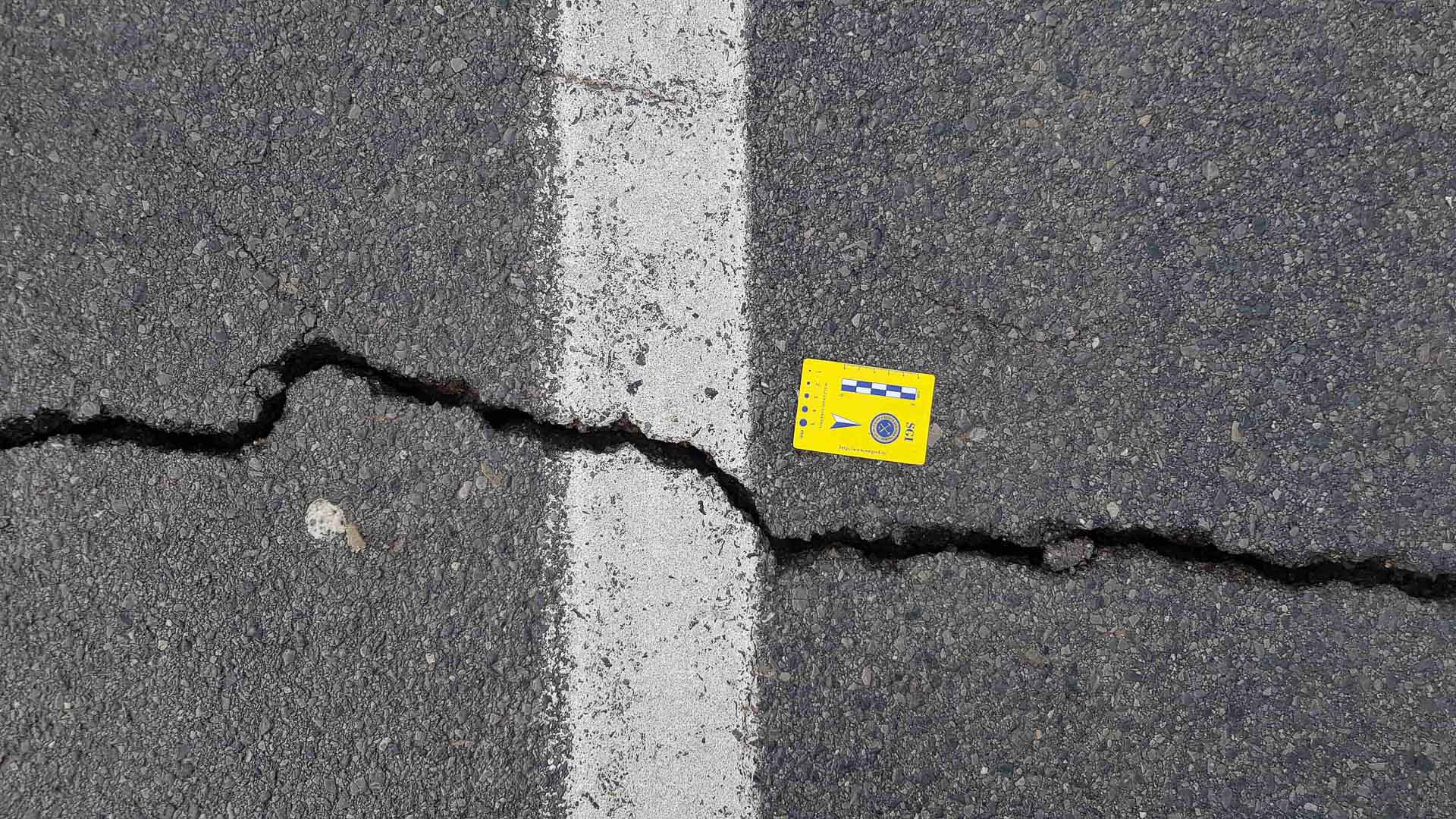

Supplement: Supplementary file 3 — Supplementary Information 3. [file 41598_2021_88378_MOESM3_ESM.zip › 96c (12-01-2021).jpg]
