# Supplementary figures and images for: ‘Conjugate’ coseismic surface faulting related with the 29 December 2020, Mw 6.4, Petrinja earthquake (Sisak-Moslavina, Croatia) (part 1 of 2)
Source: Sci Rep. 2021 Apr 28;11:9150. doi: 10.1038/s41598-021-88378-2 (PMC8080844; doi:10.1038/s41598-021-88378-2)

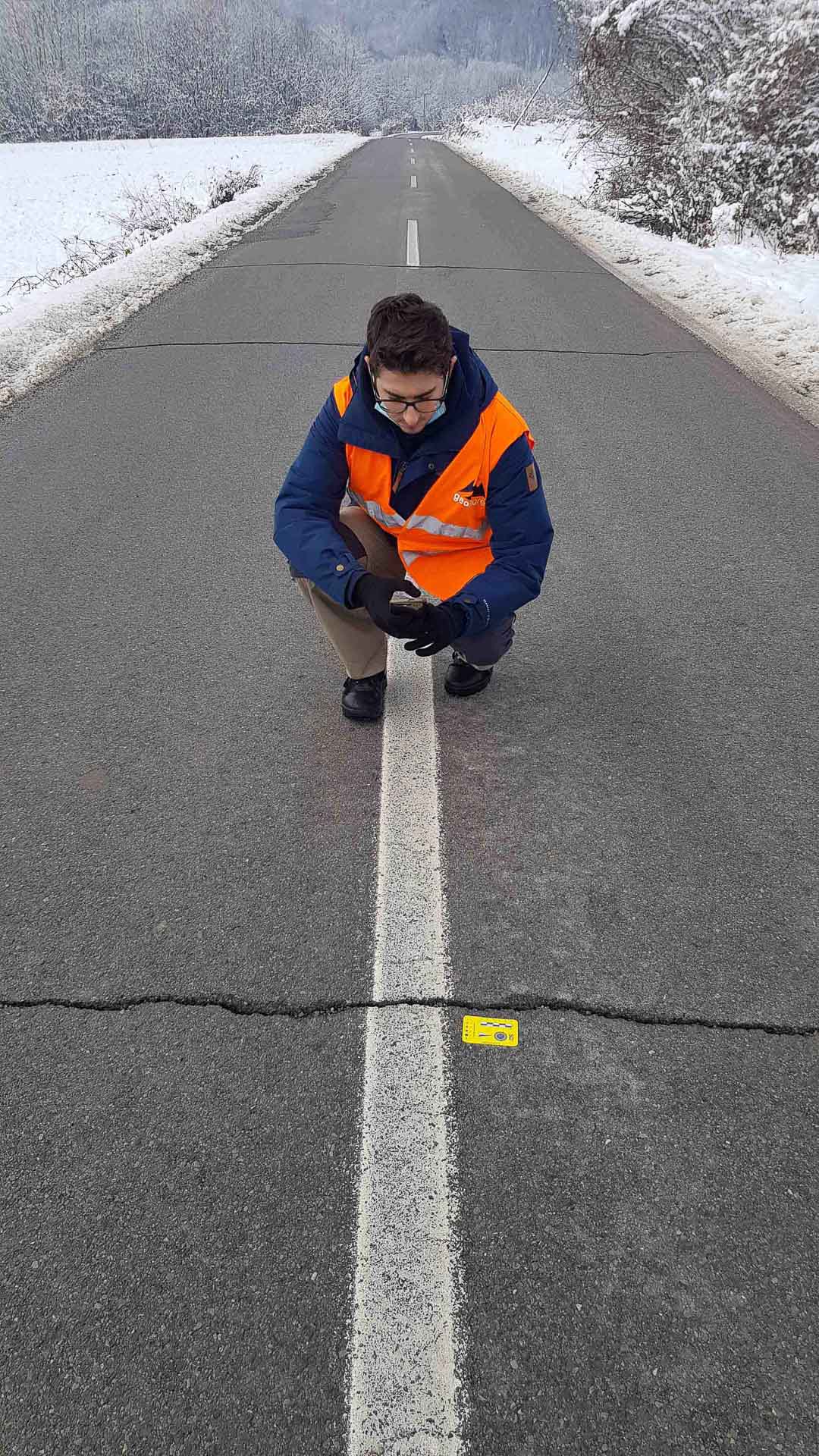

Supplement: Supplementary file 3 — Supplementary Information 3. [file 41598_2021_88378_MOESM3_ESM.zip › 100a (12-01-2021).jpg]

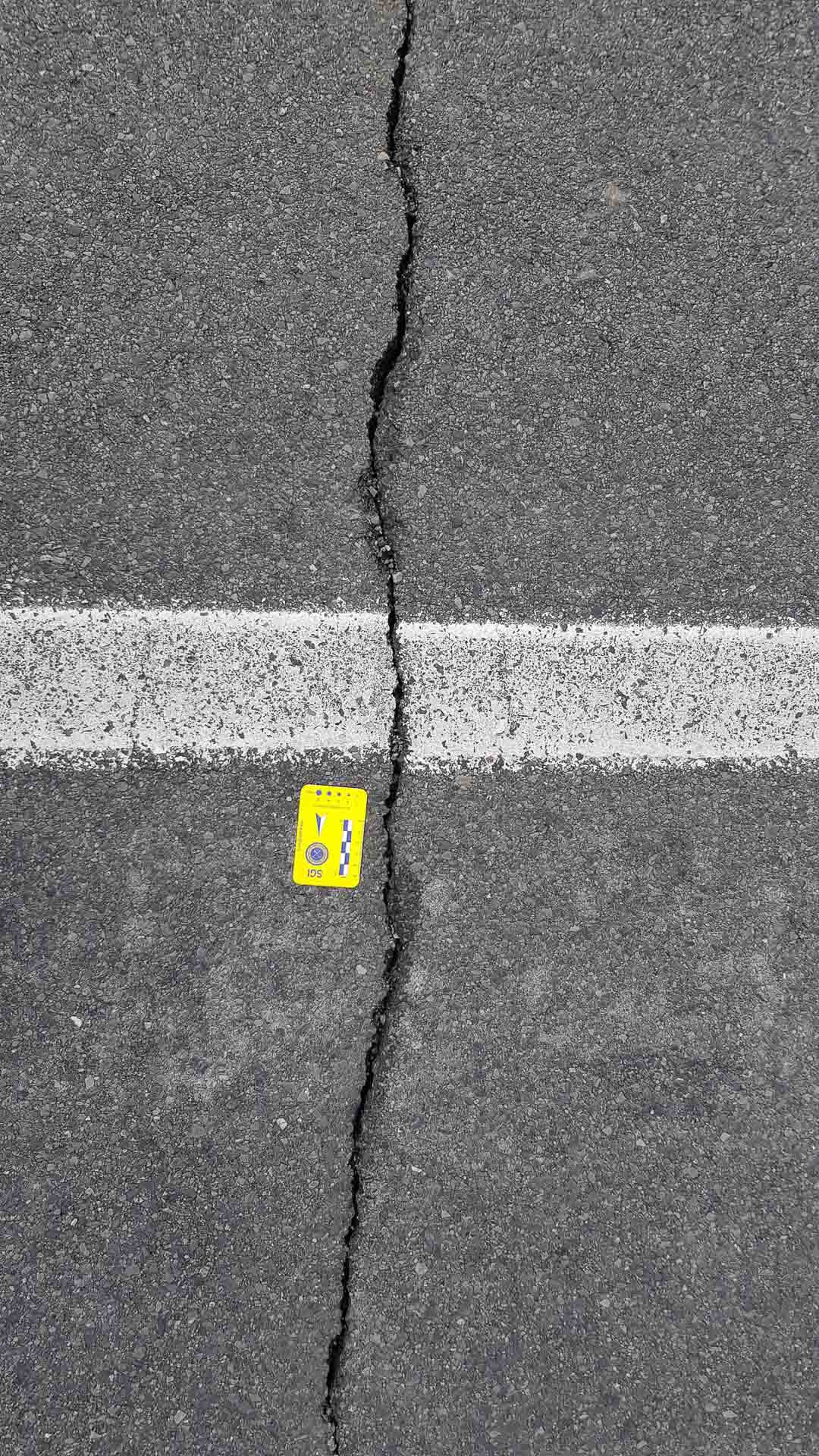

Supplement: Supplementary file 3 — Supplementary Information 3. [file 41598_2021_88378_MOESM3_ESM.zip › 100b (12-01-2021).jpg]

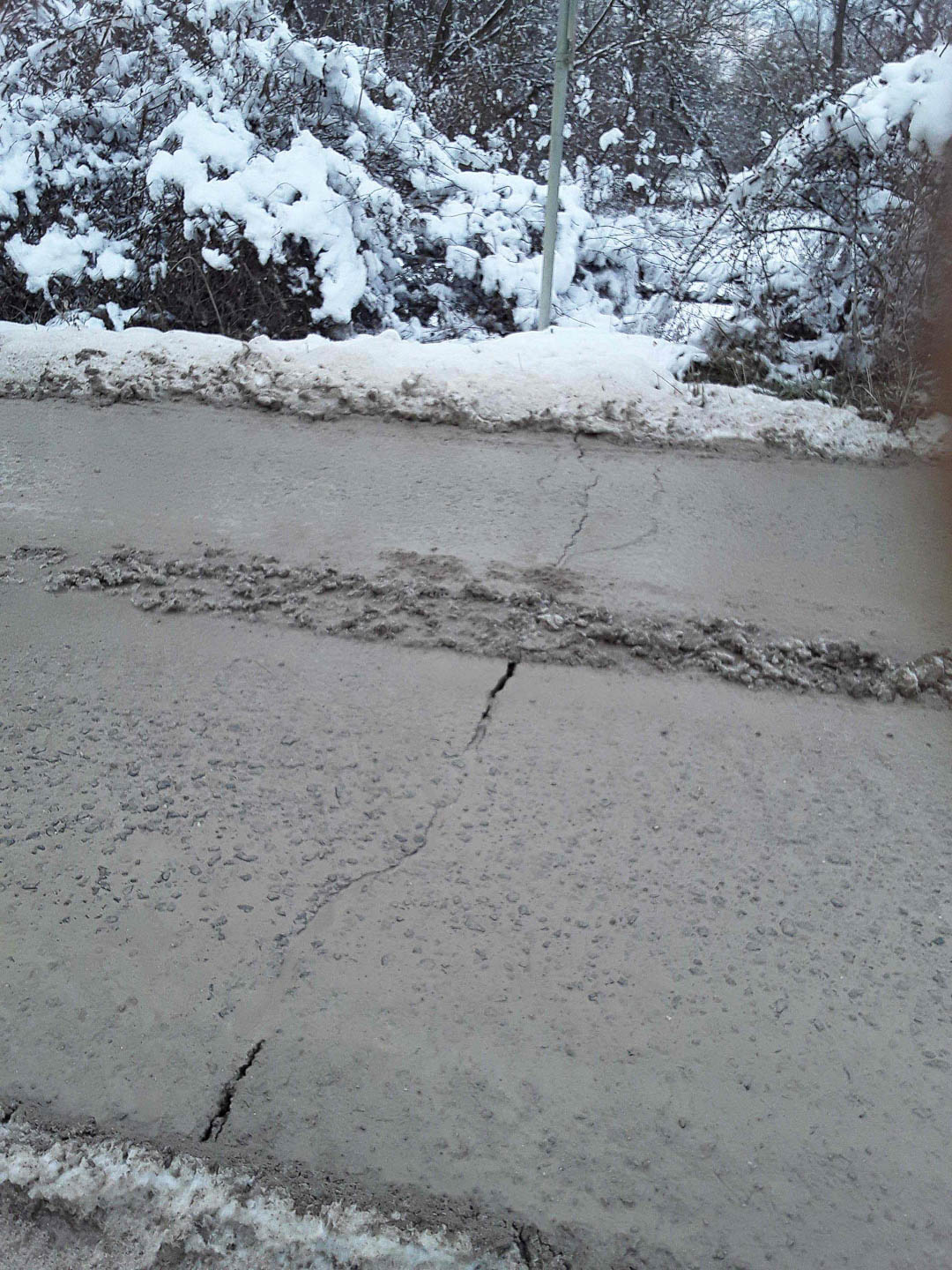

Supplement: Supplementary file 3 — Supplementary Information 3. [file 41598_2021_88378_MOESM3_ESM.zip › 117 (12-01-2021).jpg]

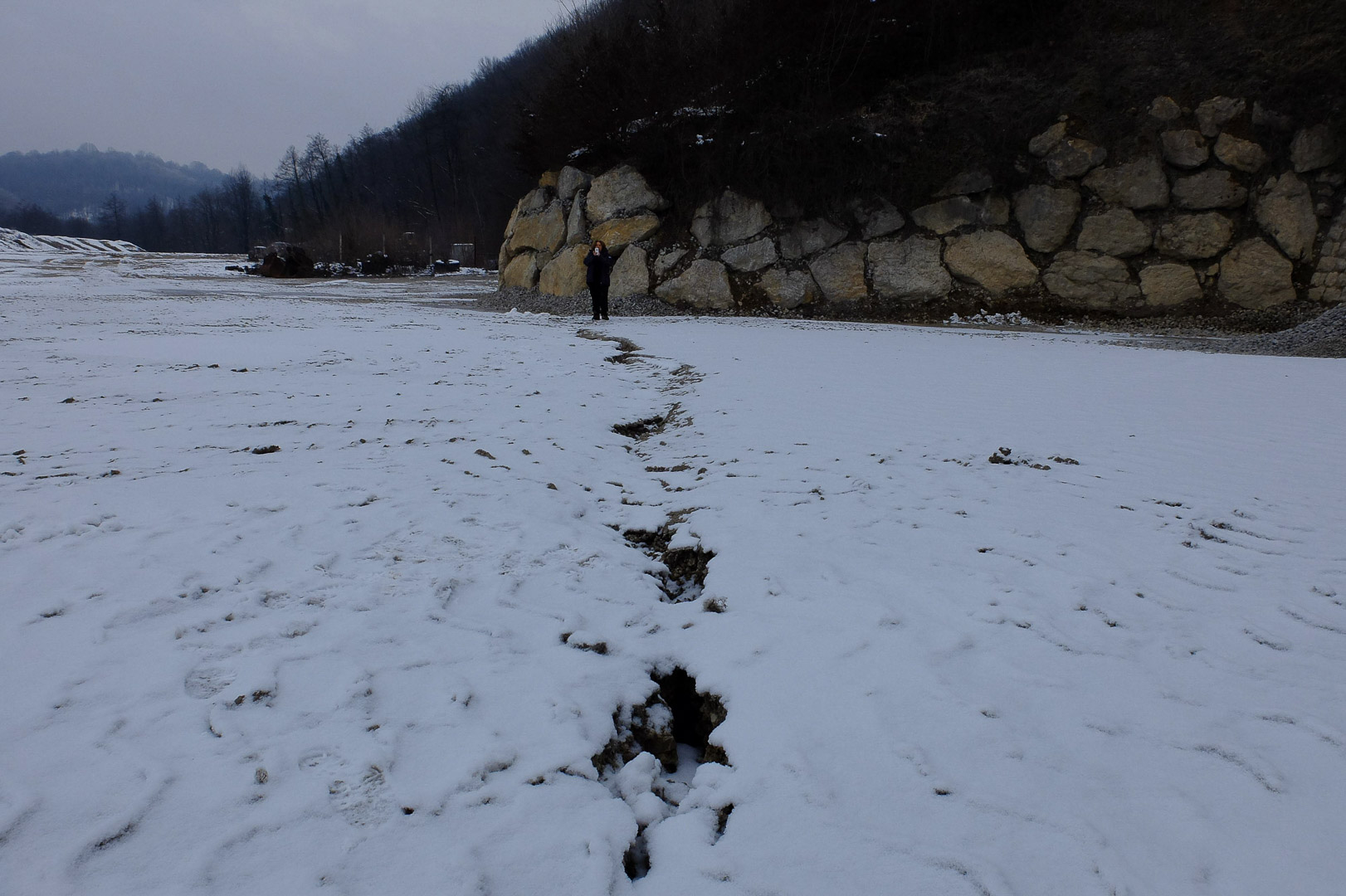

Supplement: Supplementary file 3 — Supplementary Information 3. [file 41598_2021_88378_MOESM3_ESM.zip › 118a (17-01-2021).jpg]

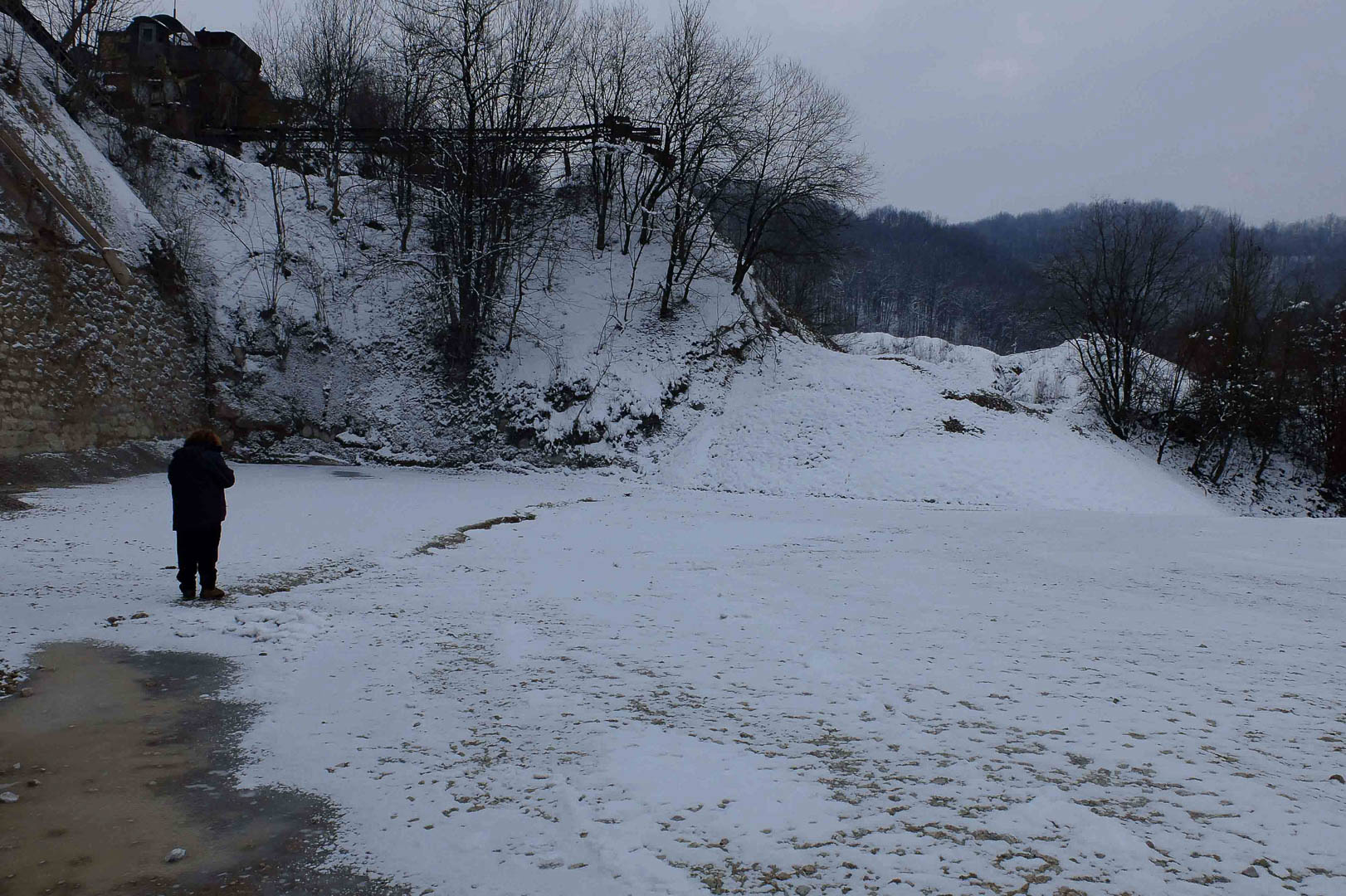

Supplement: Supplementary file 3 — Supplementary Information 3. [file 41598_2021_88378_MOESM3_ESM.zip › 118b (17-01-2021).jpg]

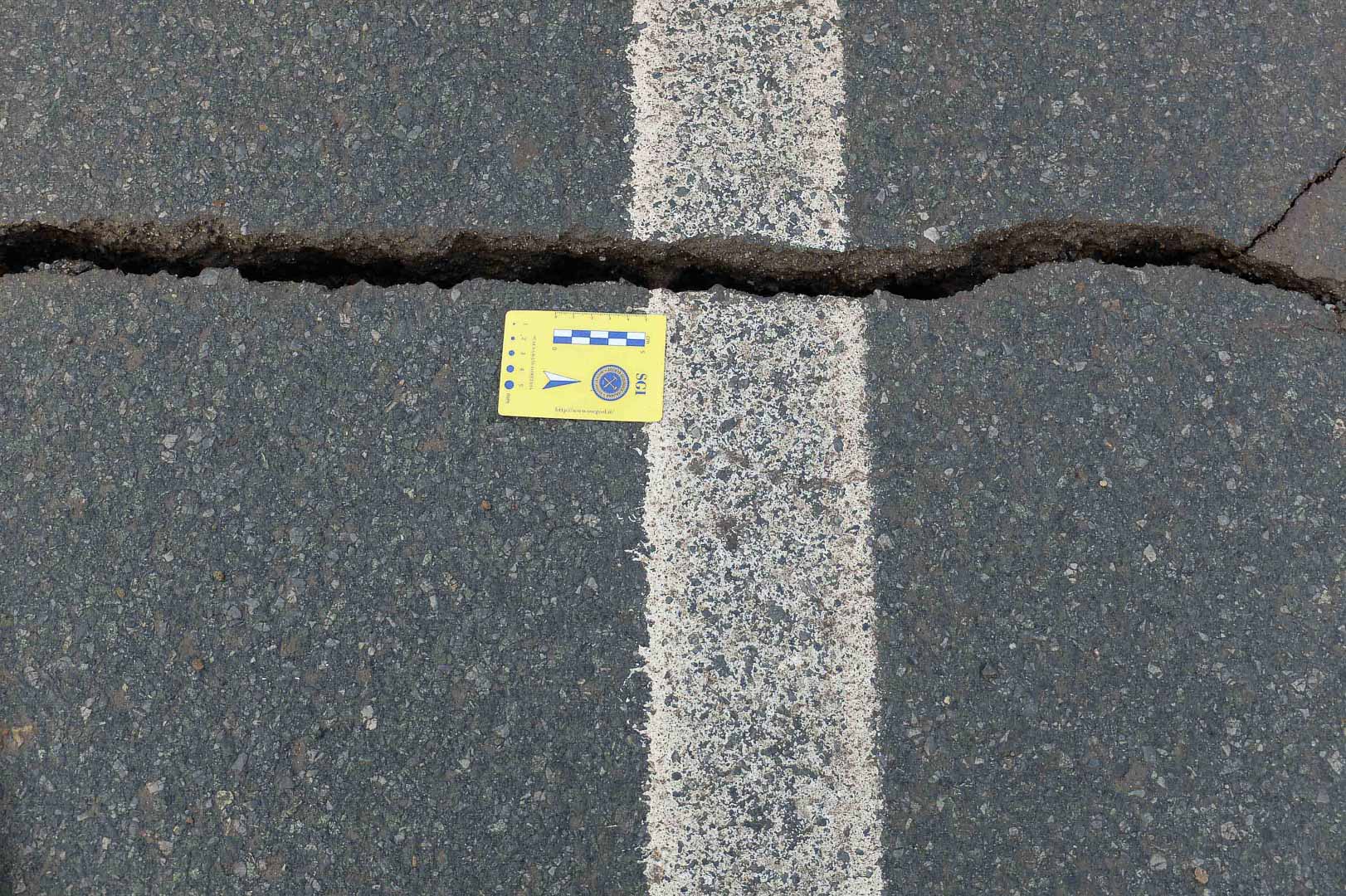

Supplement: Supplementary file 3 — Supplementary Information 3. [file 41598_2021_88378_MOESM3_ESM.zip › 120a (13-01-2021).jpg]

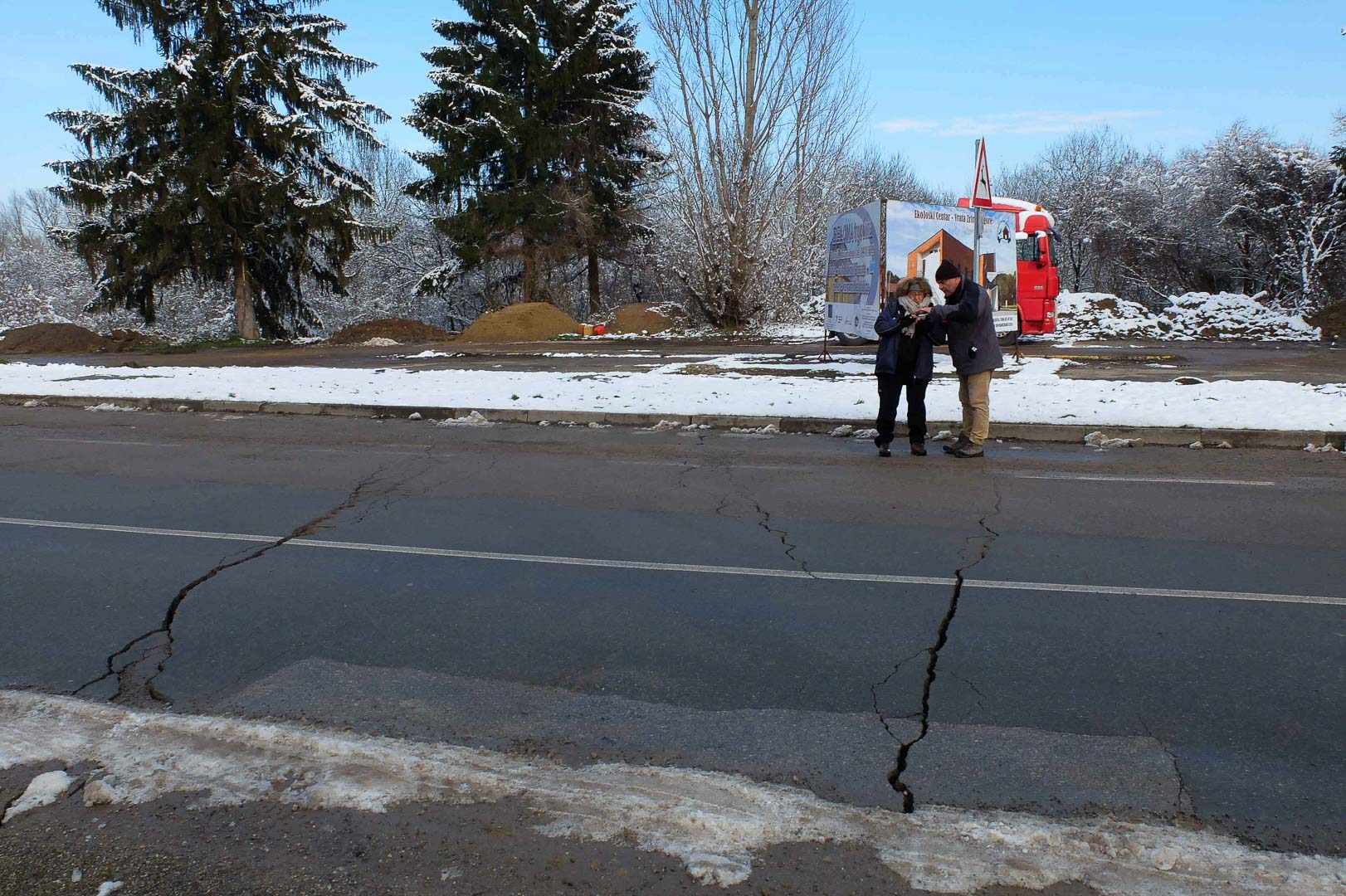

Supplement: Supplementary file 3 — Supplementary Information 3. [file 41598_2021_88378_MOESM3_ESM.zip › 120b (13-01-2021).jpg]

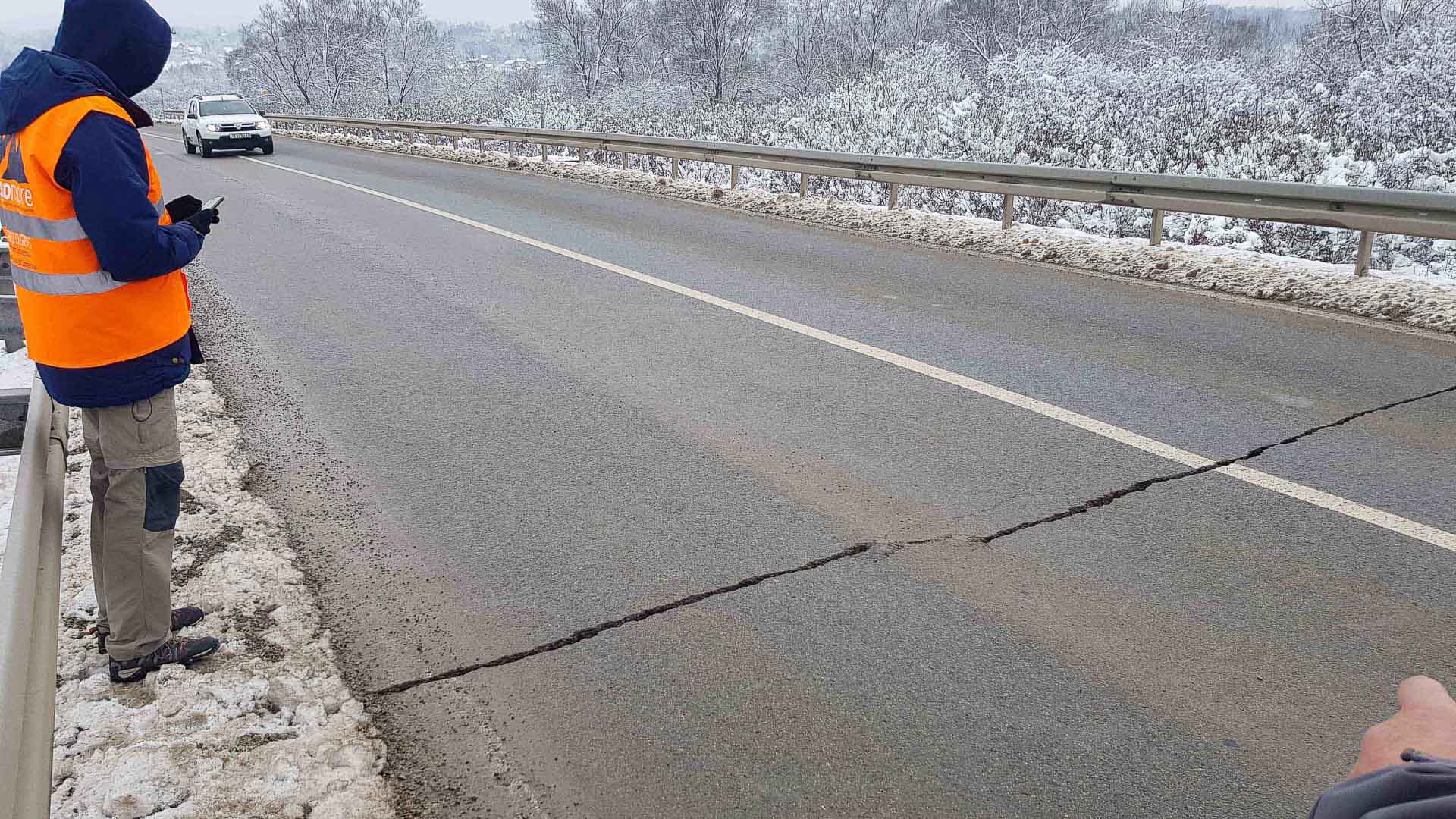

Supplement: Supplementary file 3 — Supplementary Information 3. [file 41598_2021_88378_MOESM3_ESM.zip › 121a (12-01-2021).jpg]

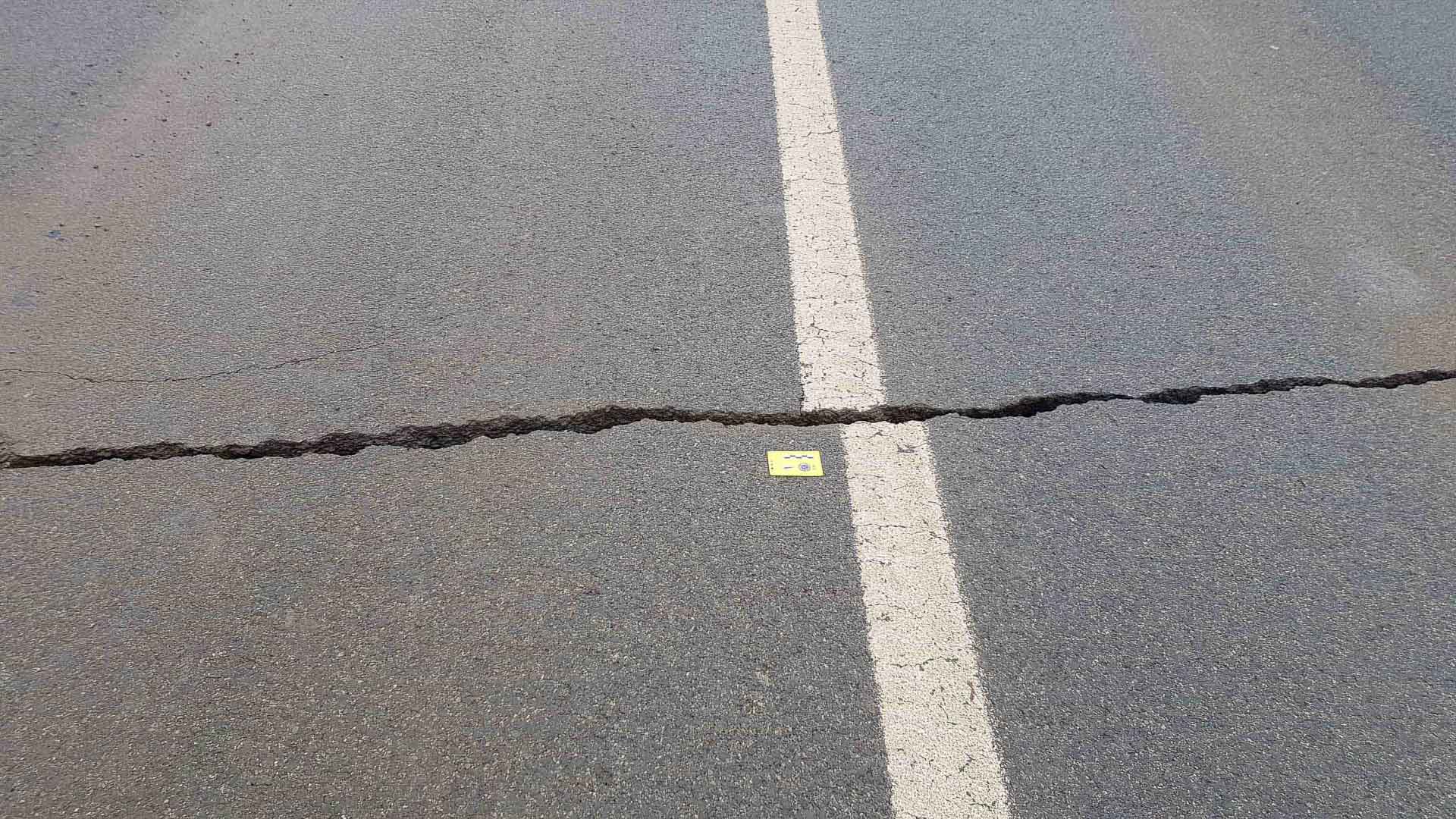

Supplement: Supplementary file 3 — Supplementary Information 3. [file 41598_2021_88378_MOESM3_ESM.zip › 121b (12-01-2021).jpg]

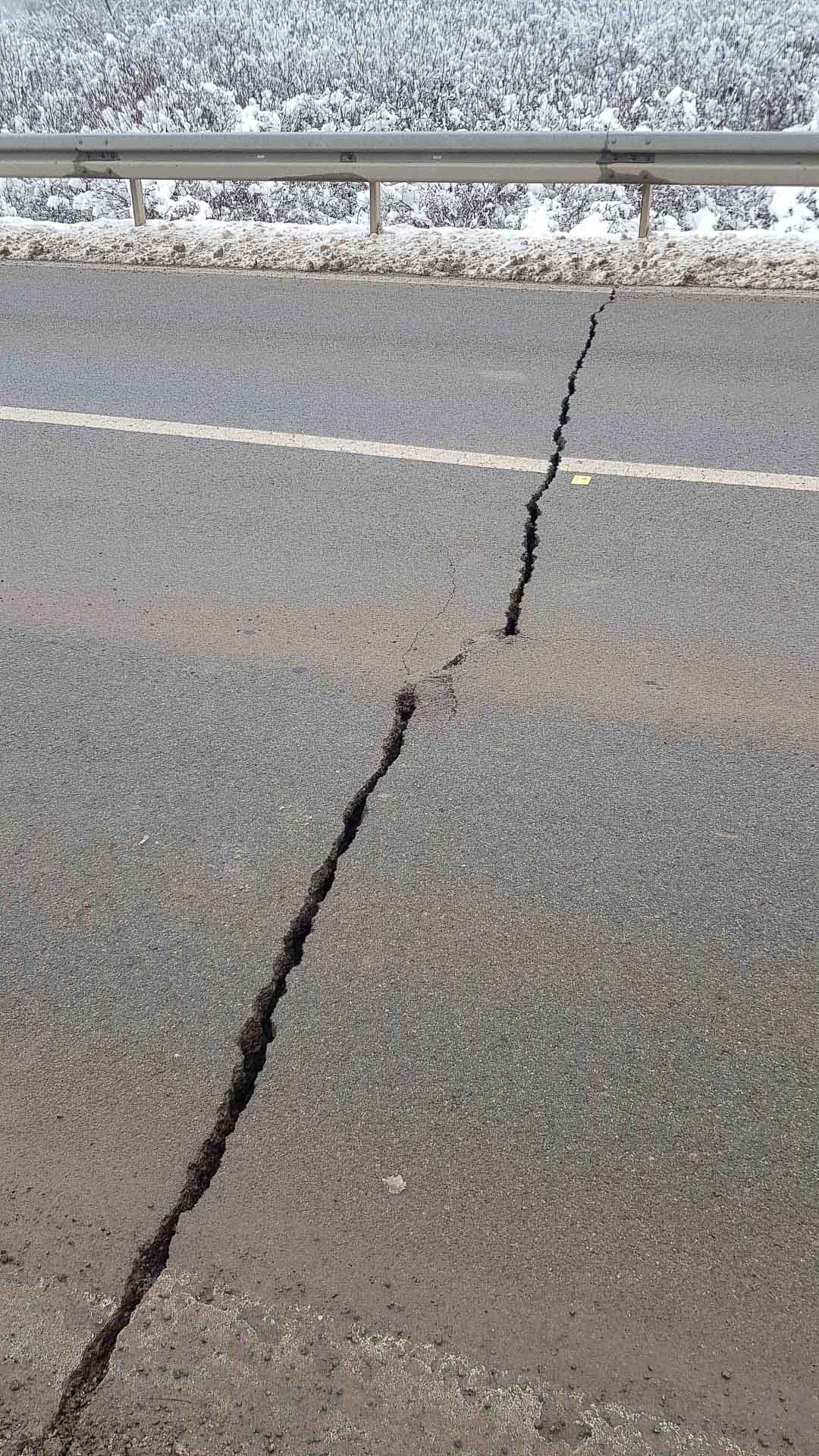

Supplement: Supplementary file 3 — Supplementary Information 3. [file 41598_2021_88378_MOESM3_ESM.zip › 121c (12-01-2021).jpg]

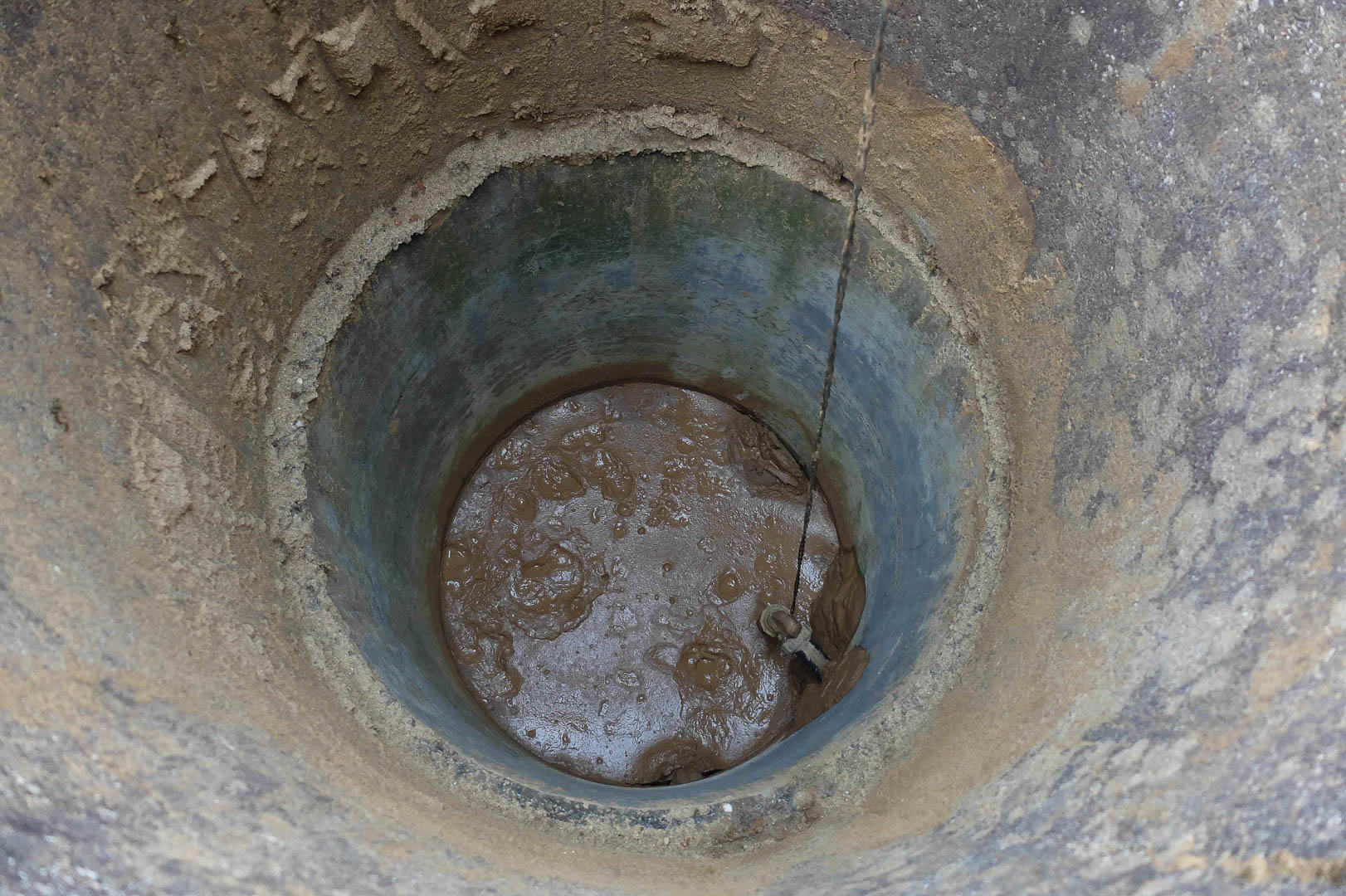

Supplement: Supplementary file 3 — Supplementary Information 3. [file 41598_2021_88378_MOESM3_ESM.zip › 126 (13-01-2021).jpg]

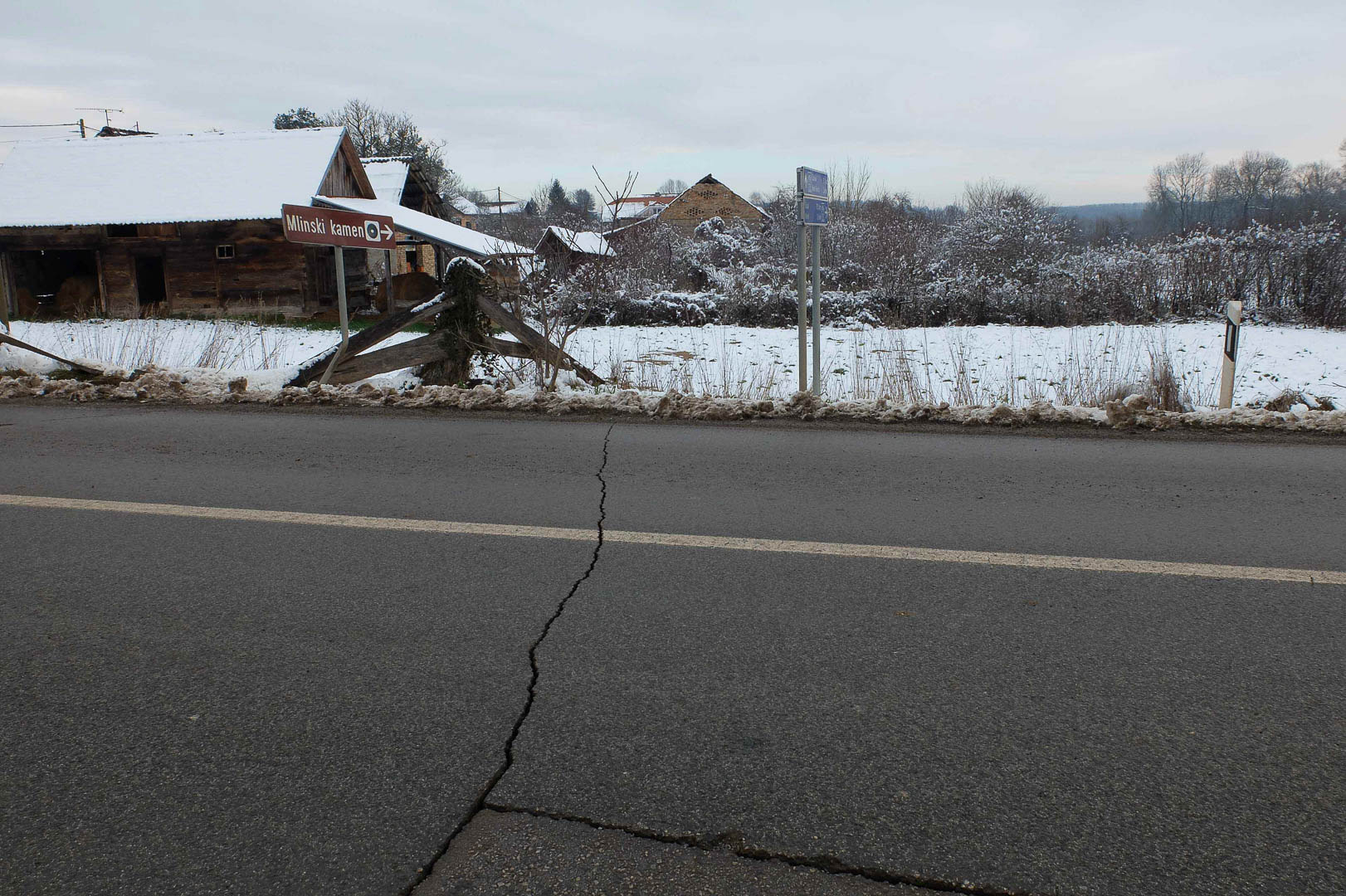

Supplement: Supplementary file 3 — Supplementary Information 3. [file 41598_2021_88378_MOESM3_ESM.zip › 128a (13-01-2021).jpg]

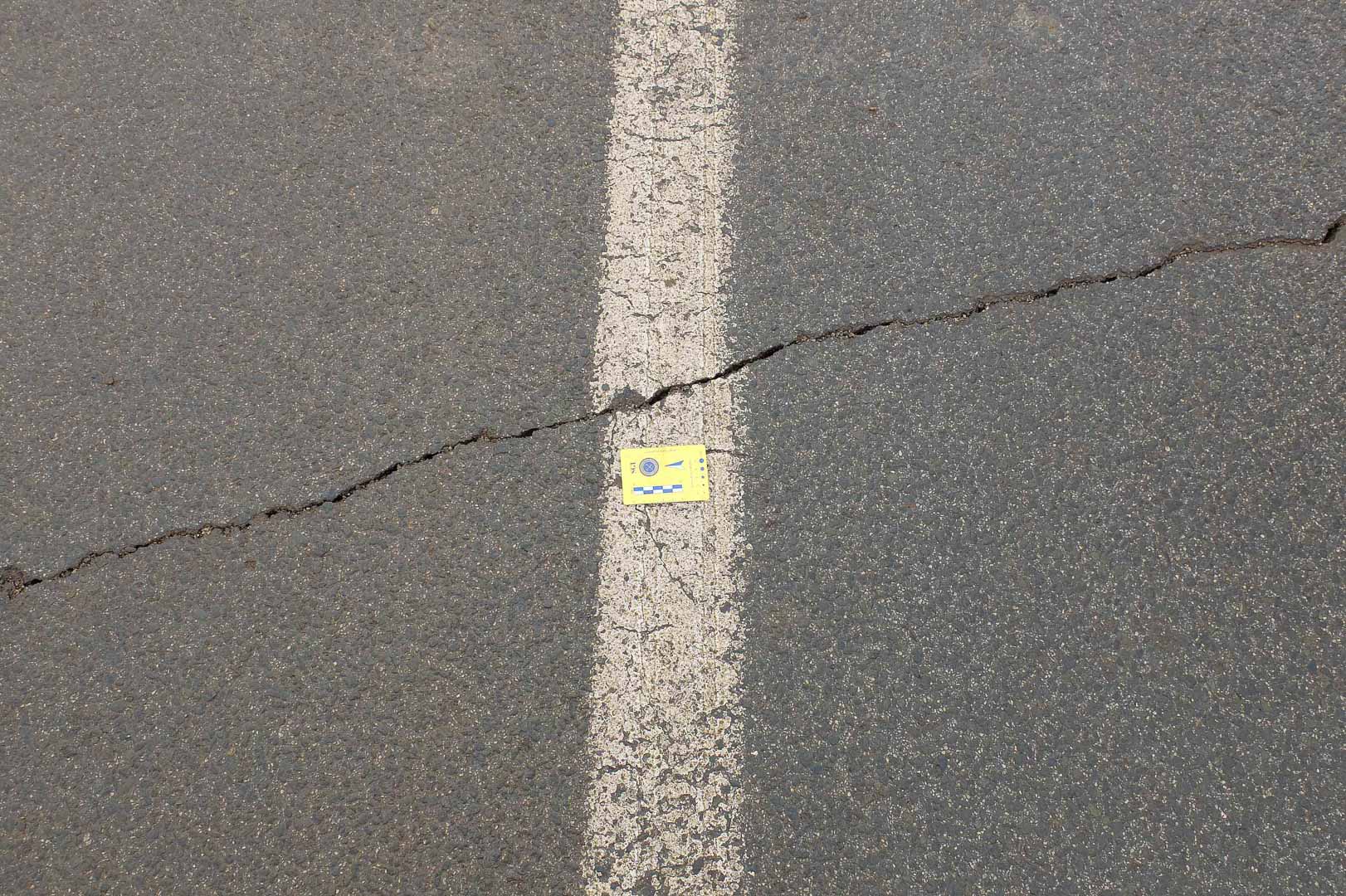

Supplement: Supplementary file 3 — Supplementary Information 3. [file 41598_2021_88378_MOESM3_ESM.zip › 128b (13-01-2021).jpg]

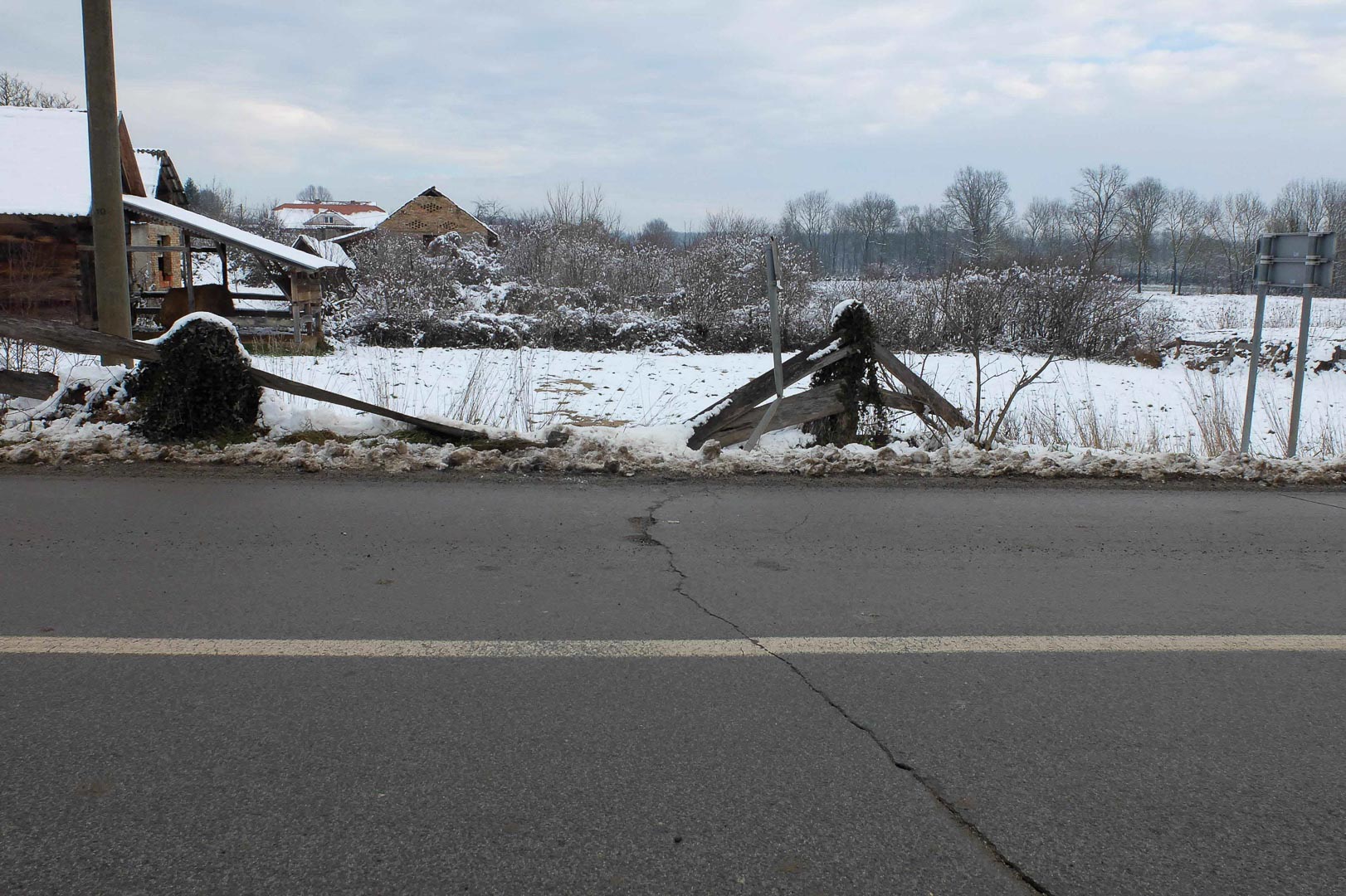

Supplement: Supplementary file 3 — Supplementary Information 3. [file 41598_2021_88378_MOESM3_ESM.zip › 129 (13-01-2021).jpg]

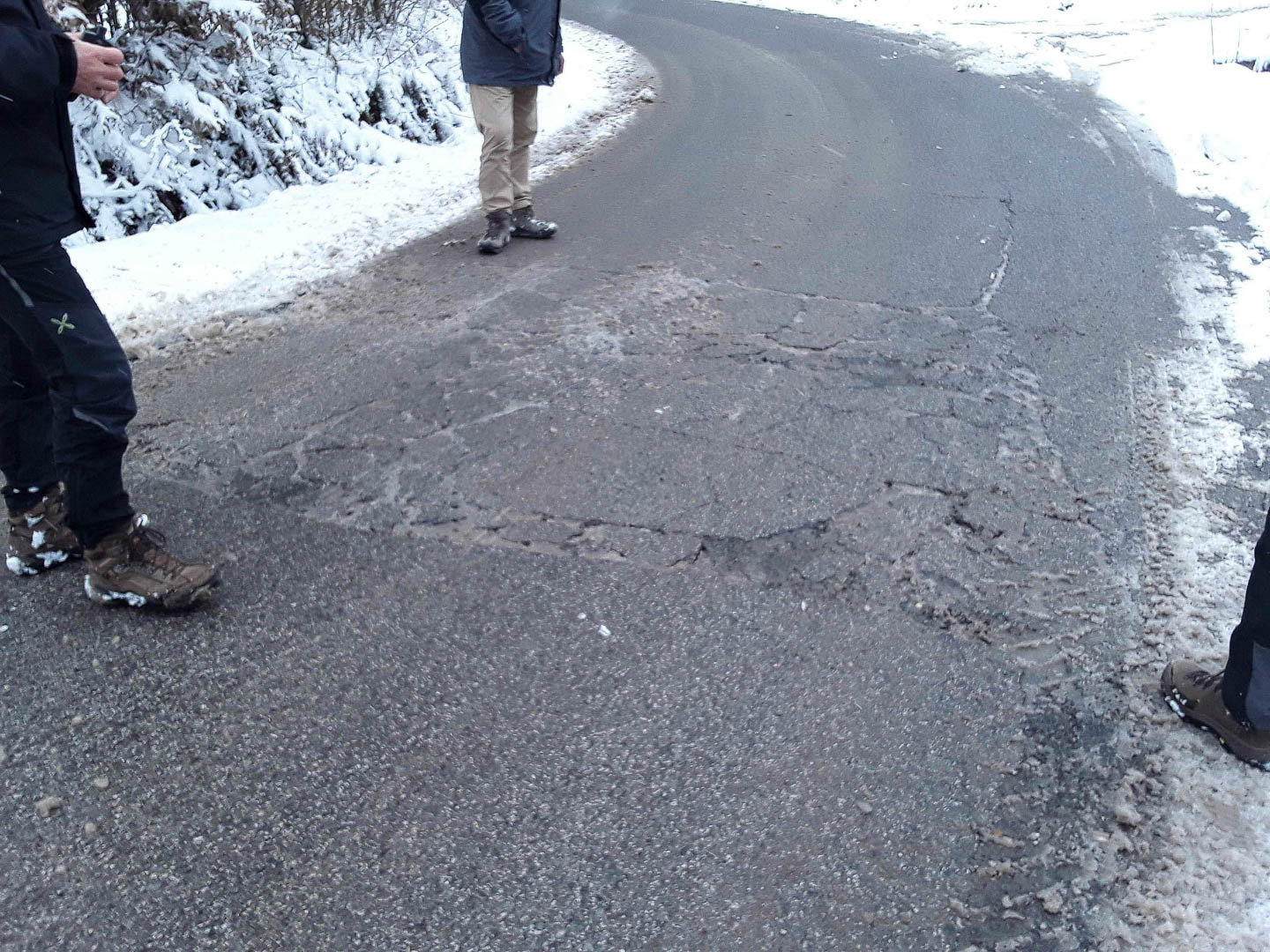

Supplement: Supplementary file 3 — Supplementary Information 3. [file 41598_2021_88378_MOESM3_ESM.zip › 13 (11-01-2021).jpg]

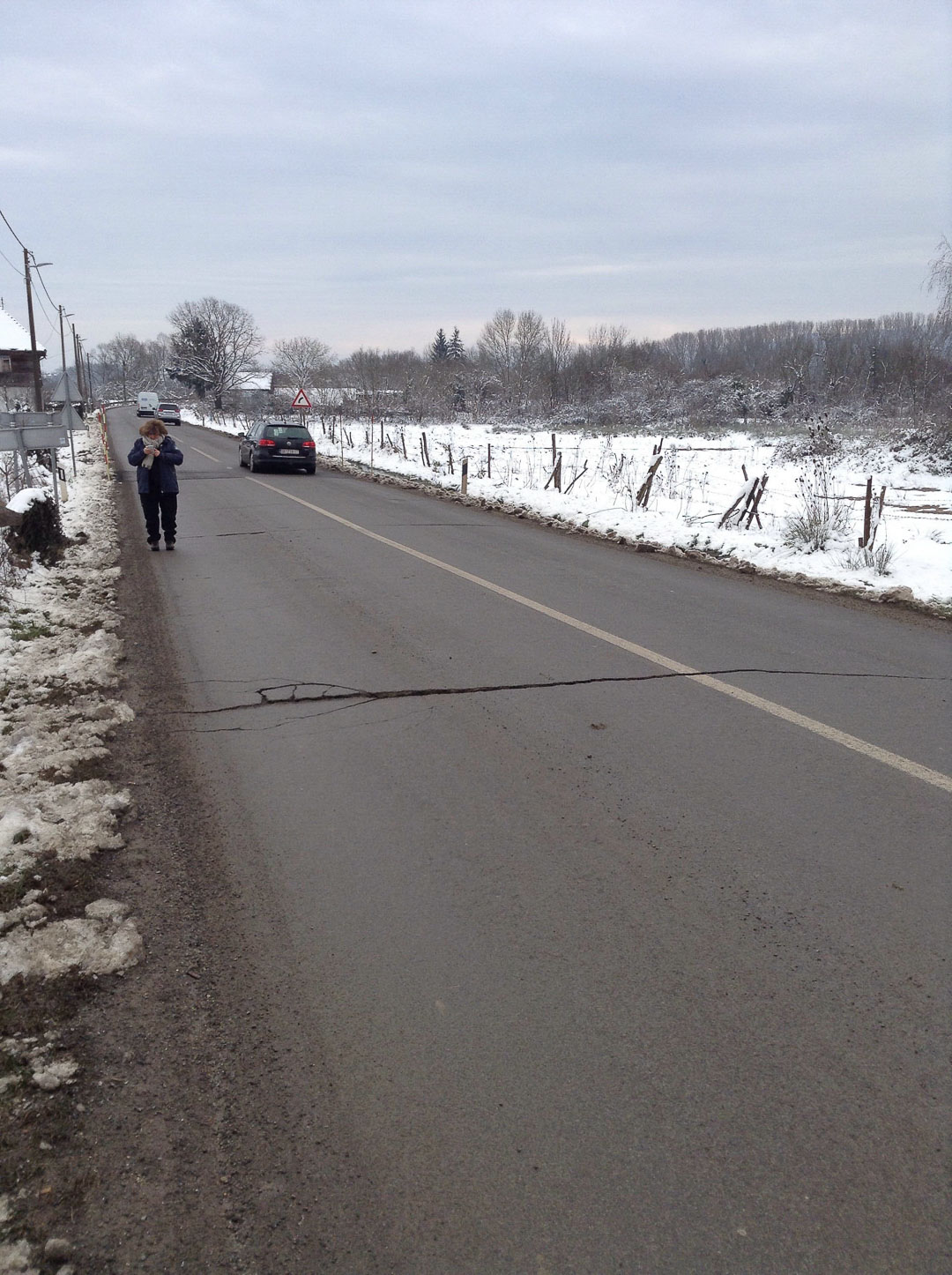

Supplement: Supplementary file 3 — Supplementary Information 3. [file 41598_2021_88378_MOESM3_ESM.zip › 133 (13-01-2021).jpg]

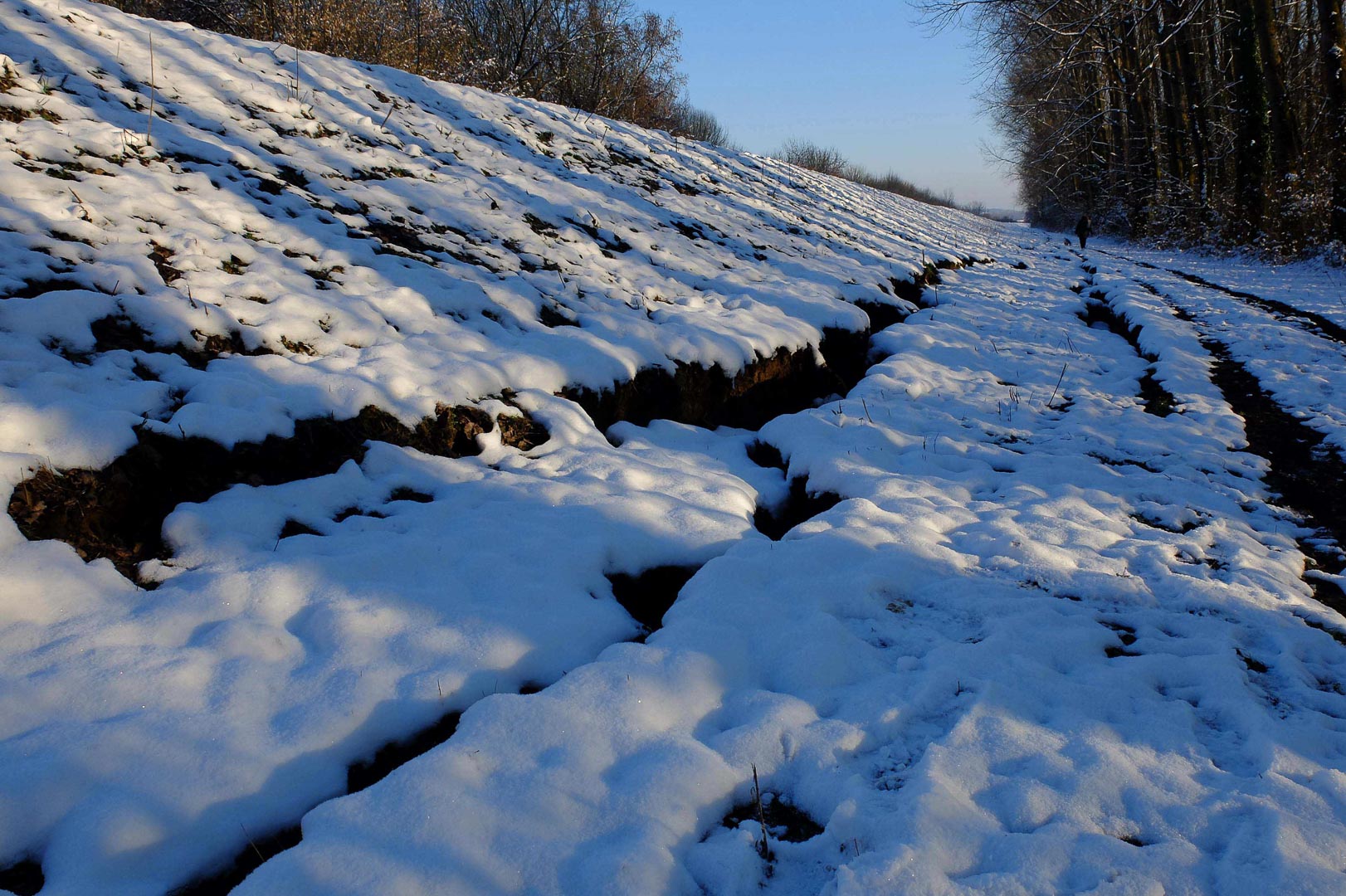

Supplement: Supplementary file 3 — Supplementary Information 3. [file 41598_2021_88378_MOESM3_ESM.zip › 139 (13-01-2021).jpg]

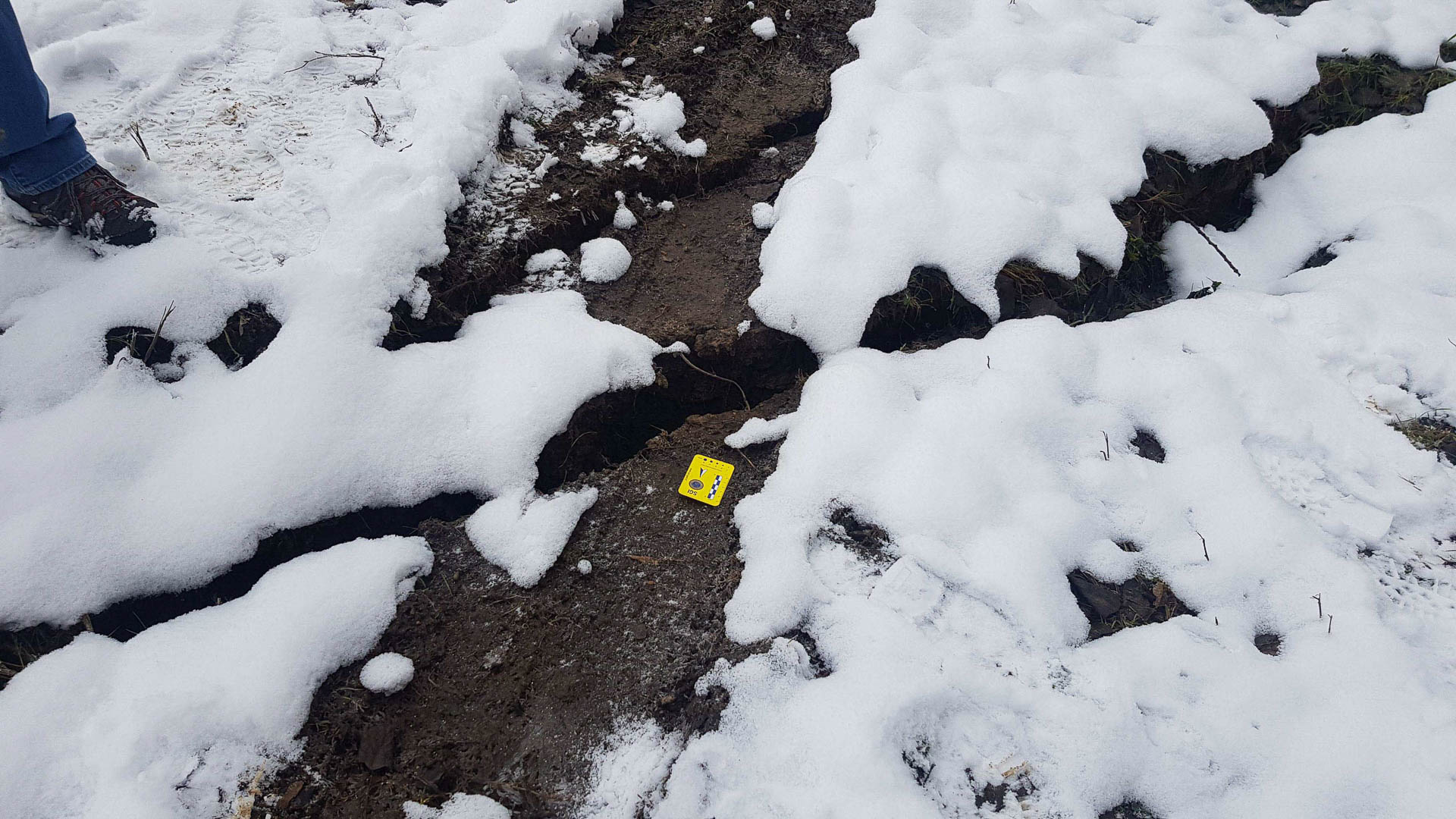

Supplement: Supplementary file 3 — Supplementary Information 3. [file 41598_2021_88378_MOESM3_ESM.zip › 141 (13-01-2021).jpg]

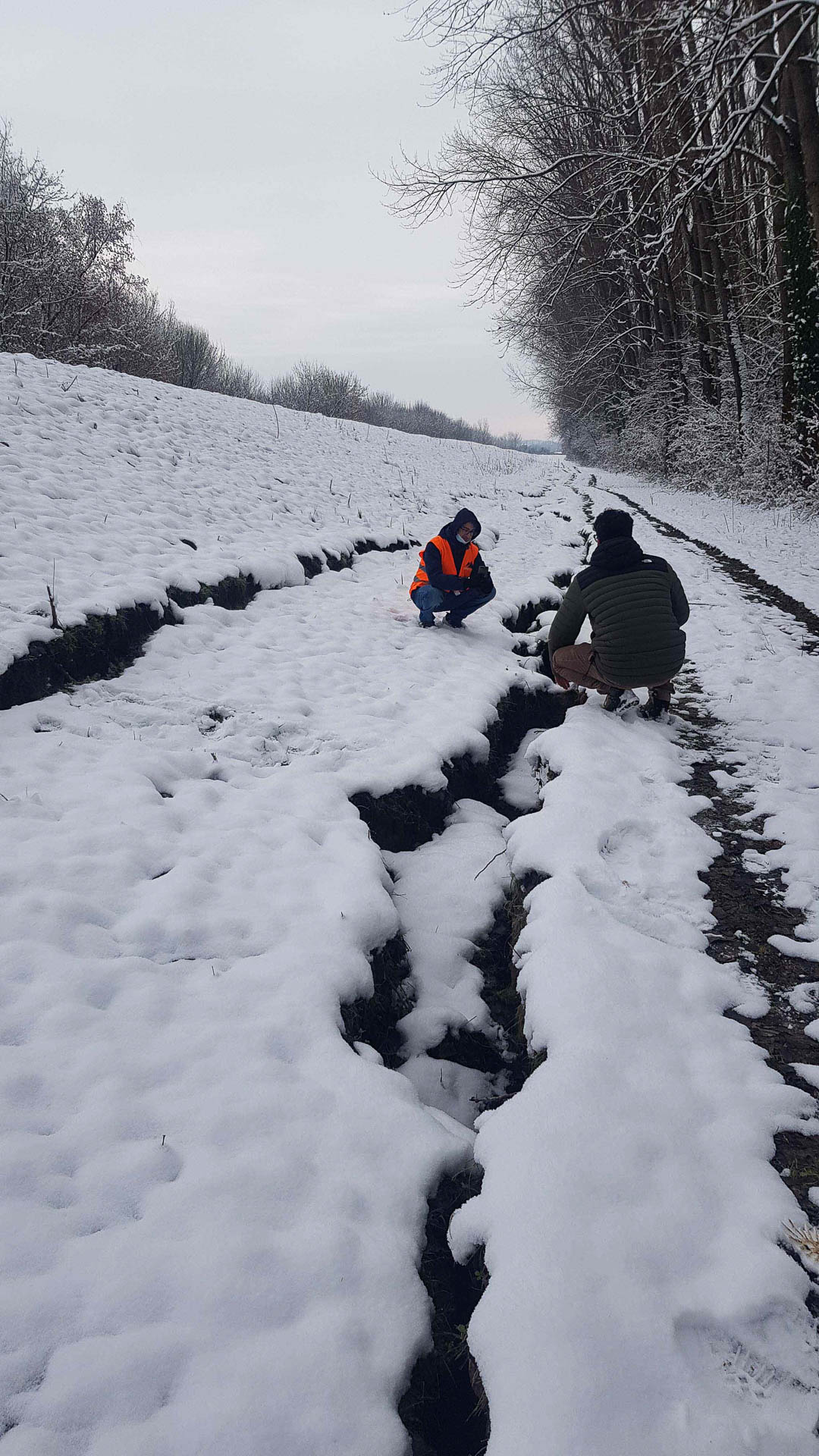

Supplement: Supplementary file 3 — Supplementary Information 3. [file 41598_2021_88378_MOESM3_ESM.zip › 142 (13-01-2021).jpg]

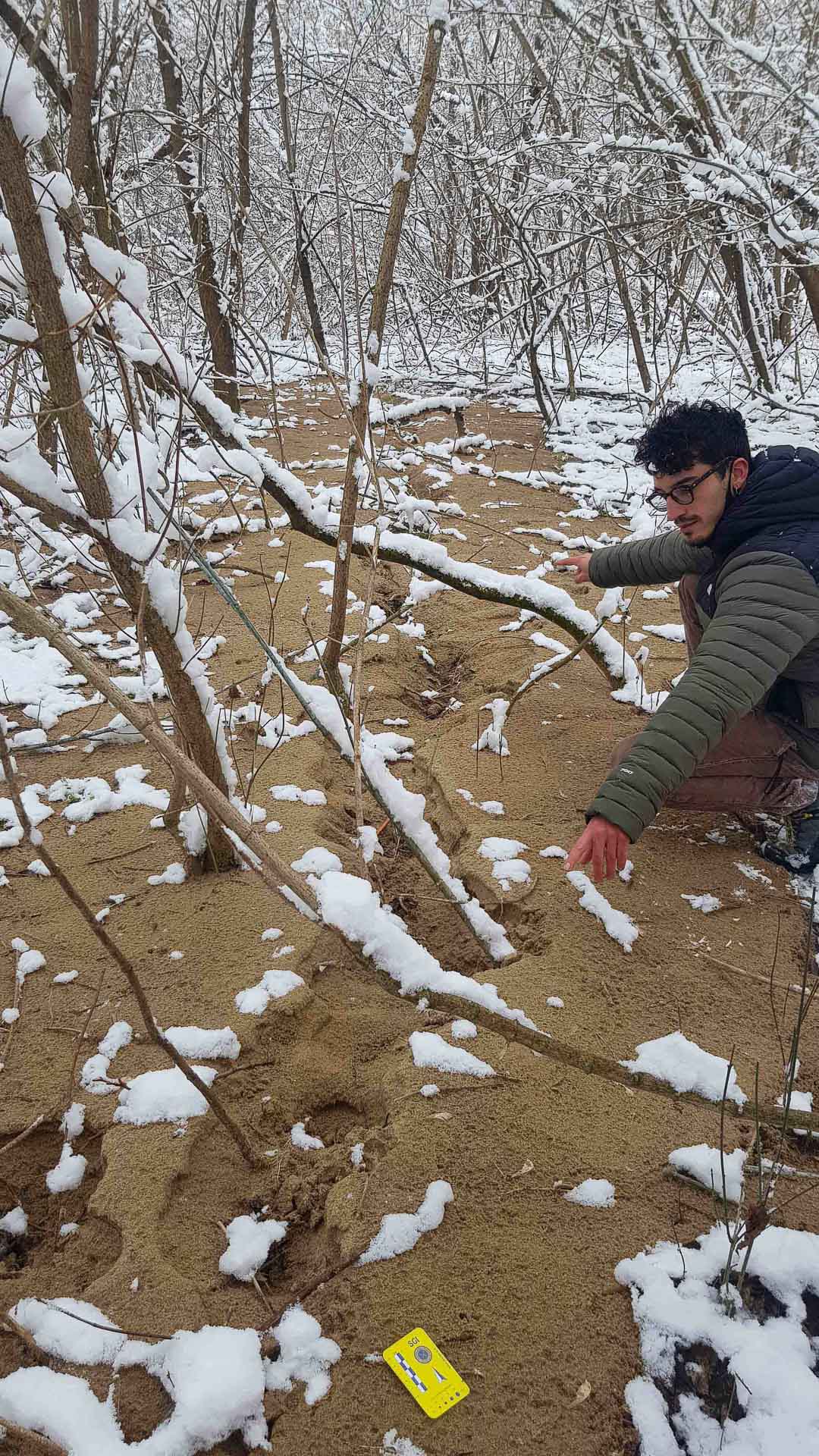

Supplement: Supplementary file 3 — Supplementary Information 3. [file 41598_2021_88378_MOESM3_ESM.zip › 144 (13-01-2021).jpg]

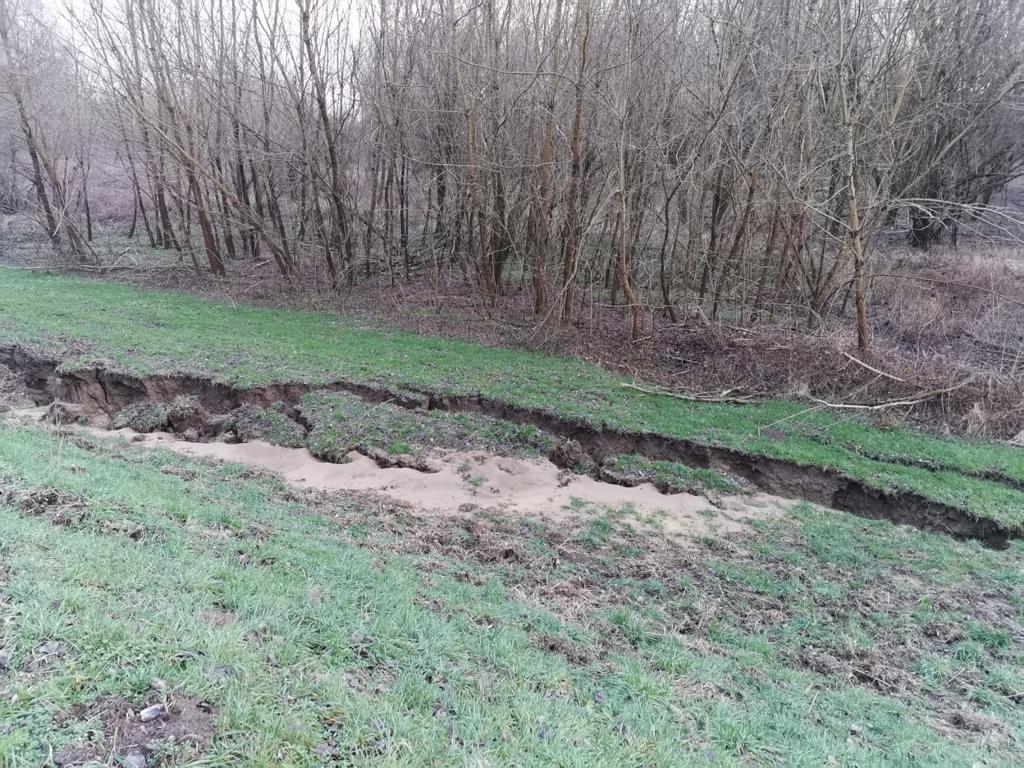

Supplement: Supplementary file 3 — Supplementary Information 3. [file 41598_2021_88378_MOESM3_ESM.zip › 147 (30-12-2020).jpg]

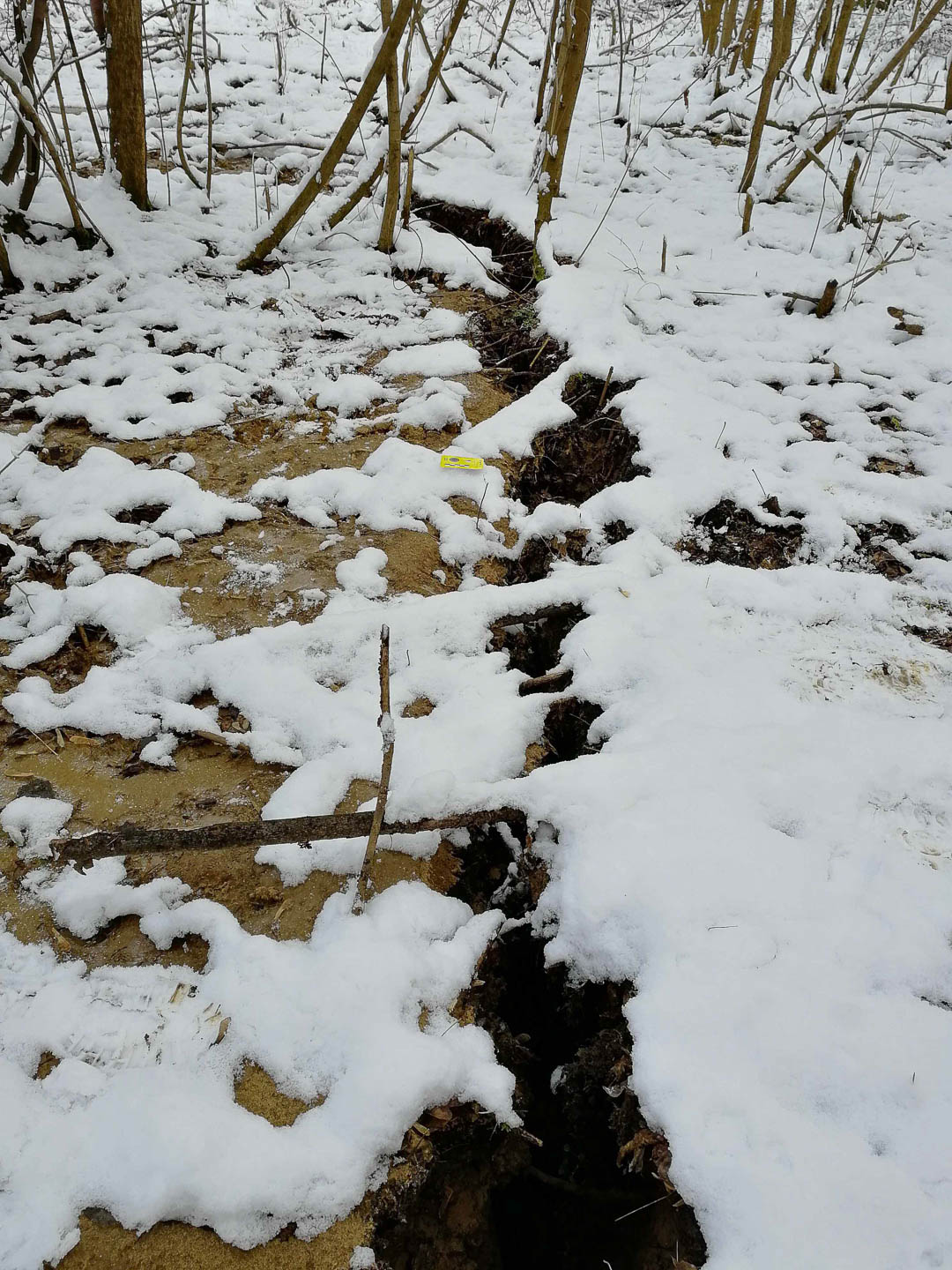

Supplement: Supplementary file 3 — Supplementary Information 3. [file 41598_2021_88378_MOESM3_ESM.zip › 149a (13-01-2021).jpg]

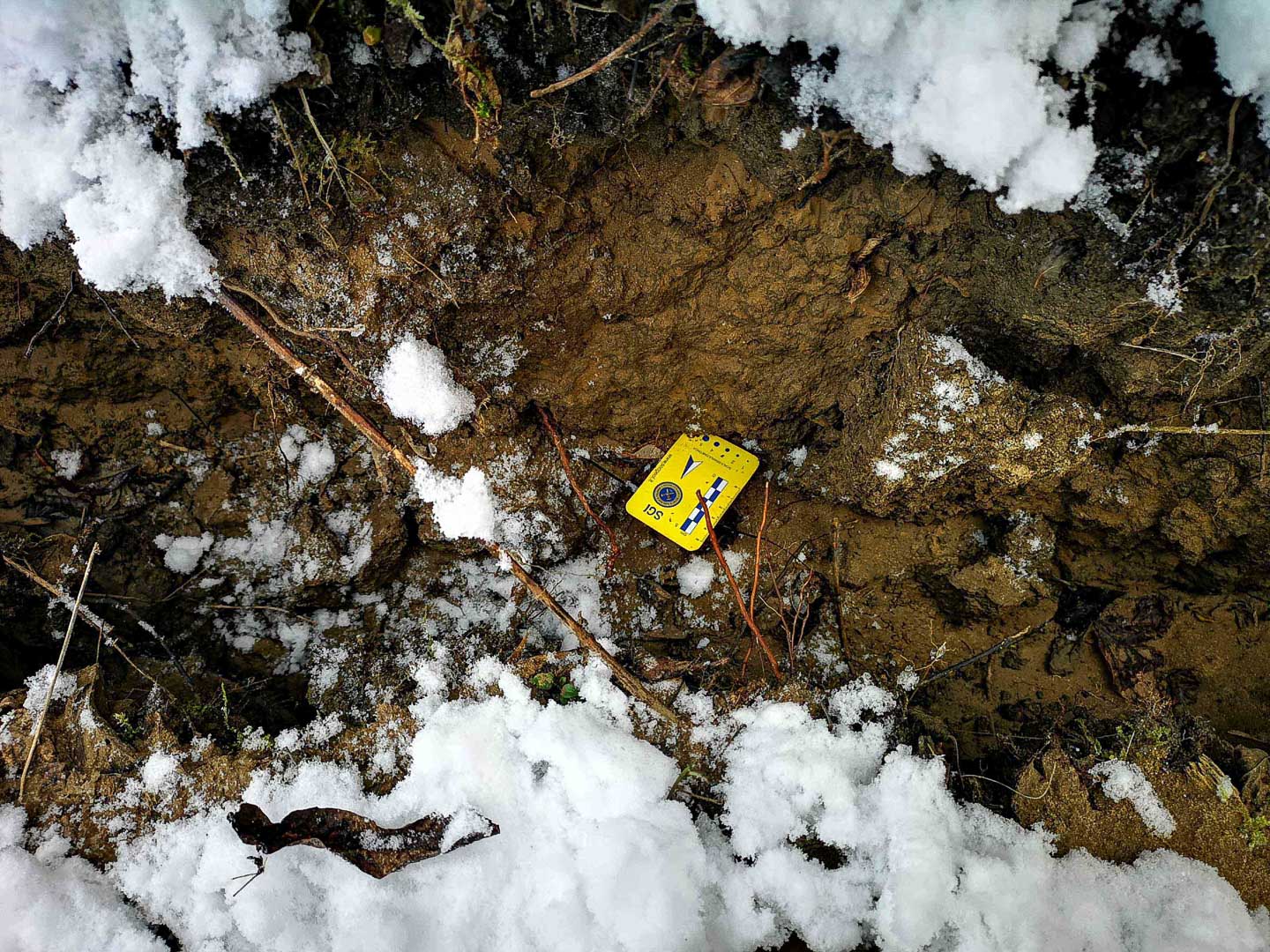

Supplement: Supplementary file 3 — Supplementary Information 3. [file 41598_2021_88378_MOESM3_ESM.zip › 149b (13-01-2021).jpg]

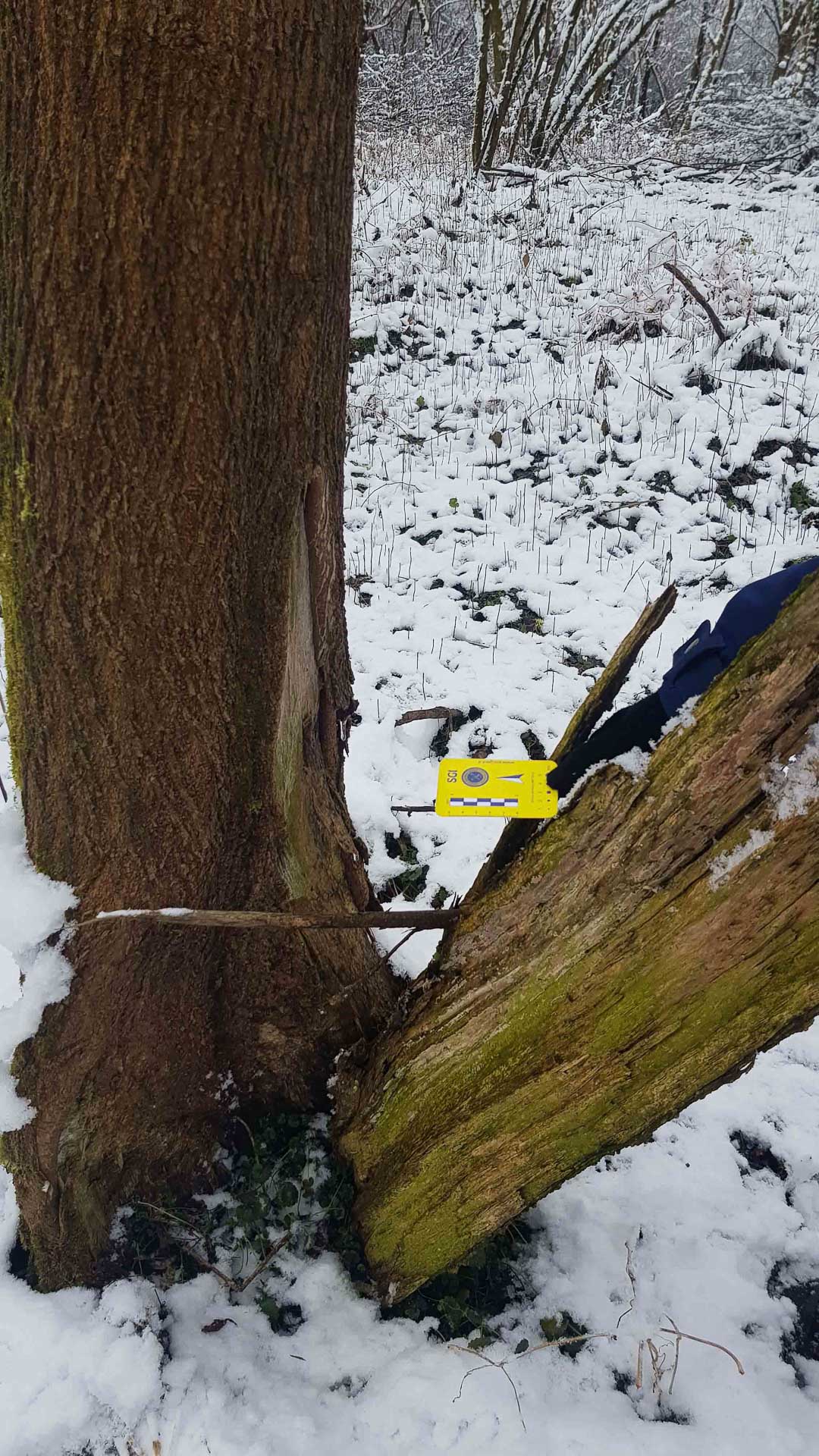

Supplement: Supplementary file 3 — Supplementary Information 3. [file 41598_2021_88378_MOESM3_ESM.zip › 149c (13-01-2021).jpg]

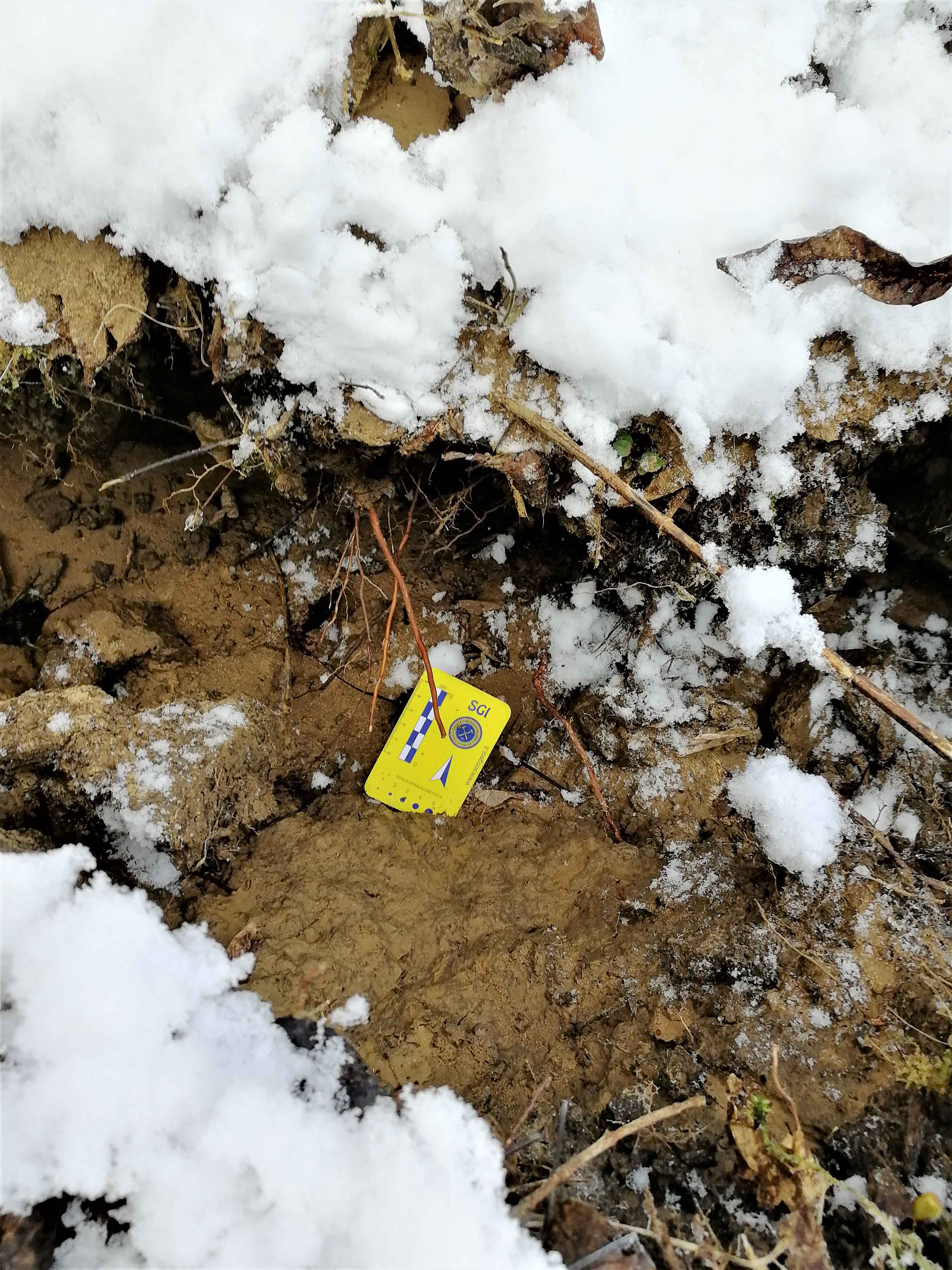

Supplement: Supplementary file 3 — Supplementary Information 3. [file 41598_2021_88378_MOESM3_ESM.zip › 149d (13-01-2021).jpg]

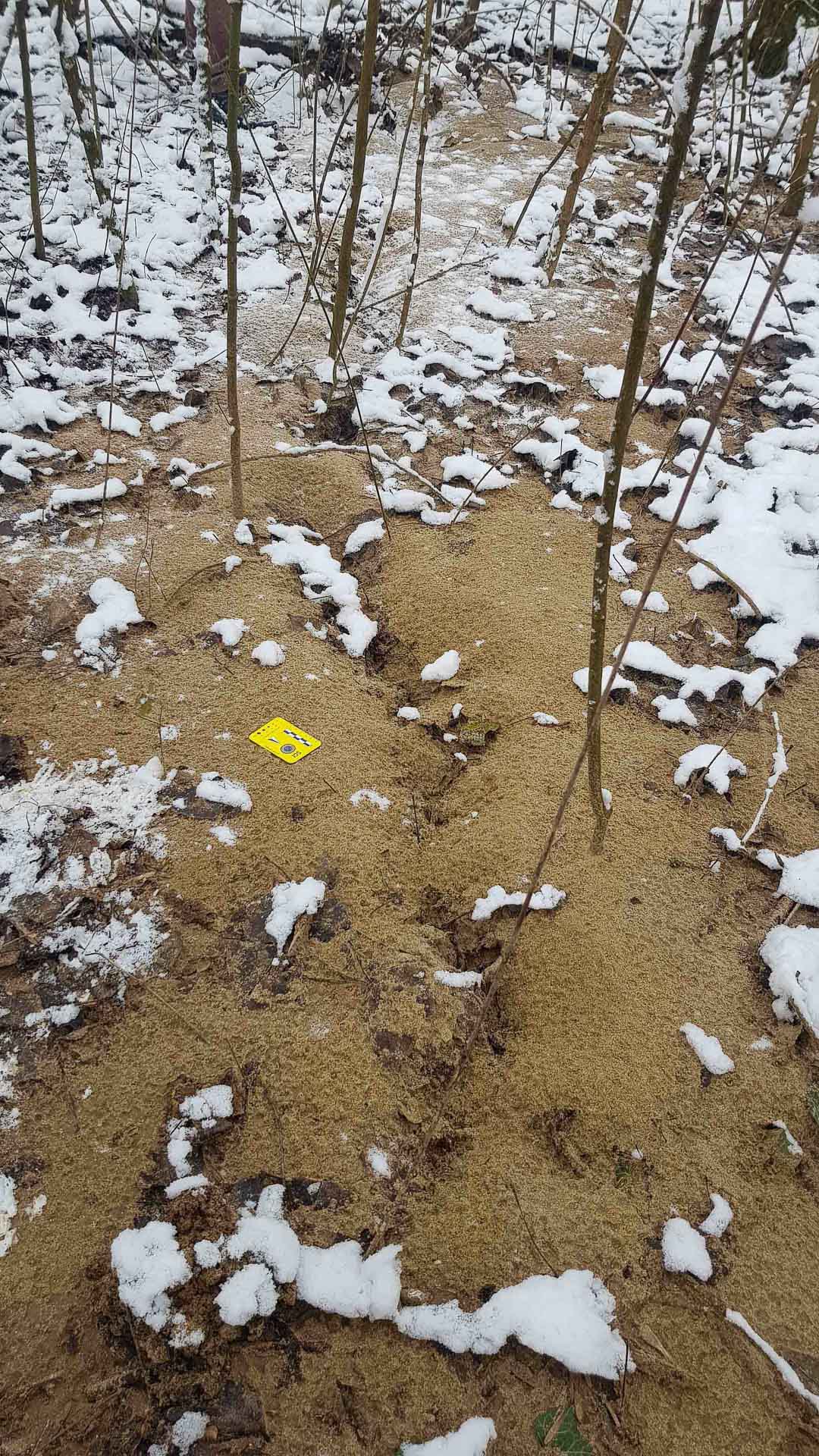

Supplement: Supplementary file 3 — Supplementary Information 3. [file 41598_2021_88378_MOESM3_ESM.zip › 154 (13-01-2021).jpg]

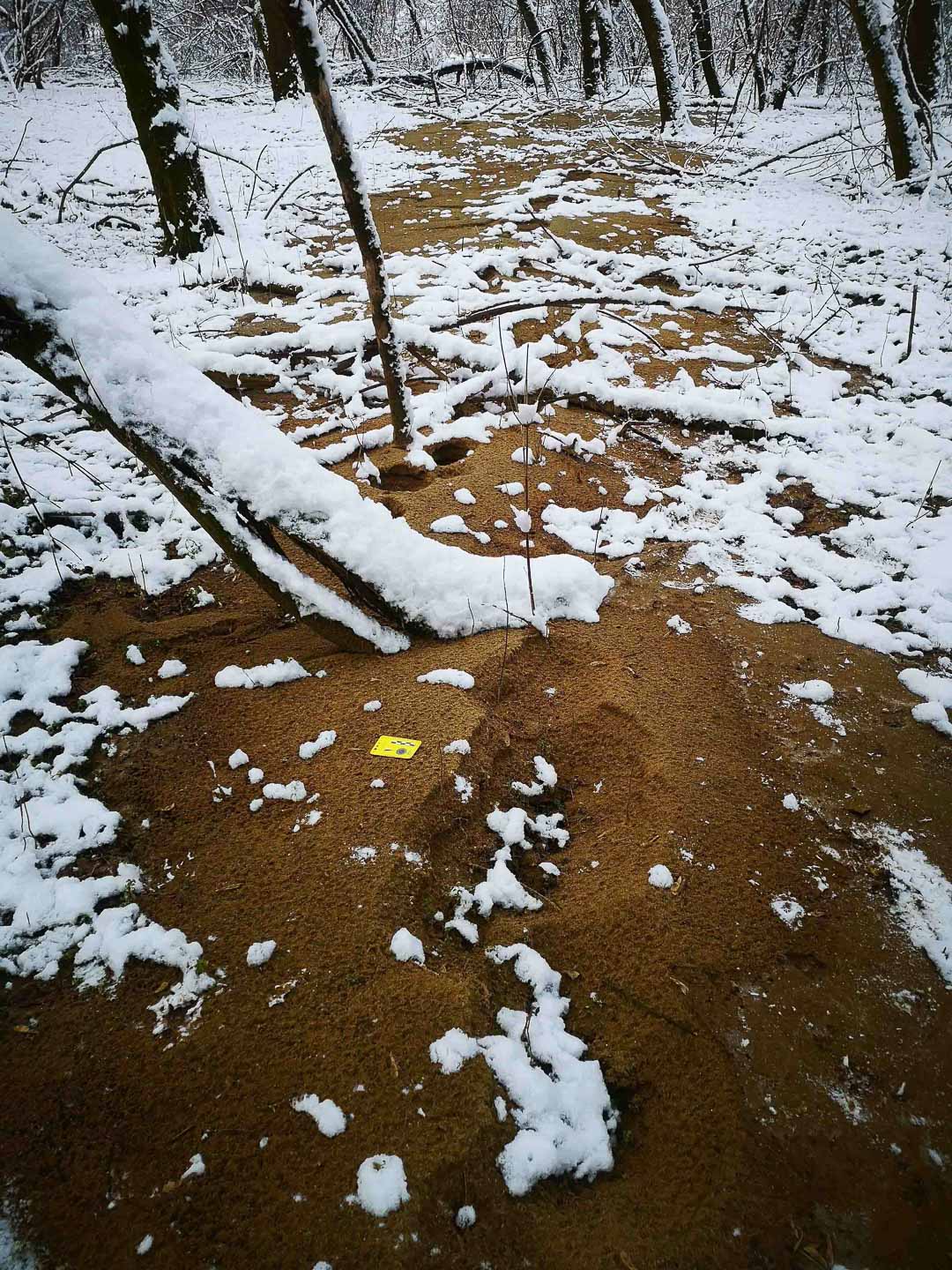

Supplement: Supplementary file 3 — Supplementary Information 3. [file 41598_2021_88378_MOESM3_ESM.zip › 155 (13-01-2021).jpg]

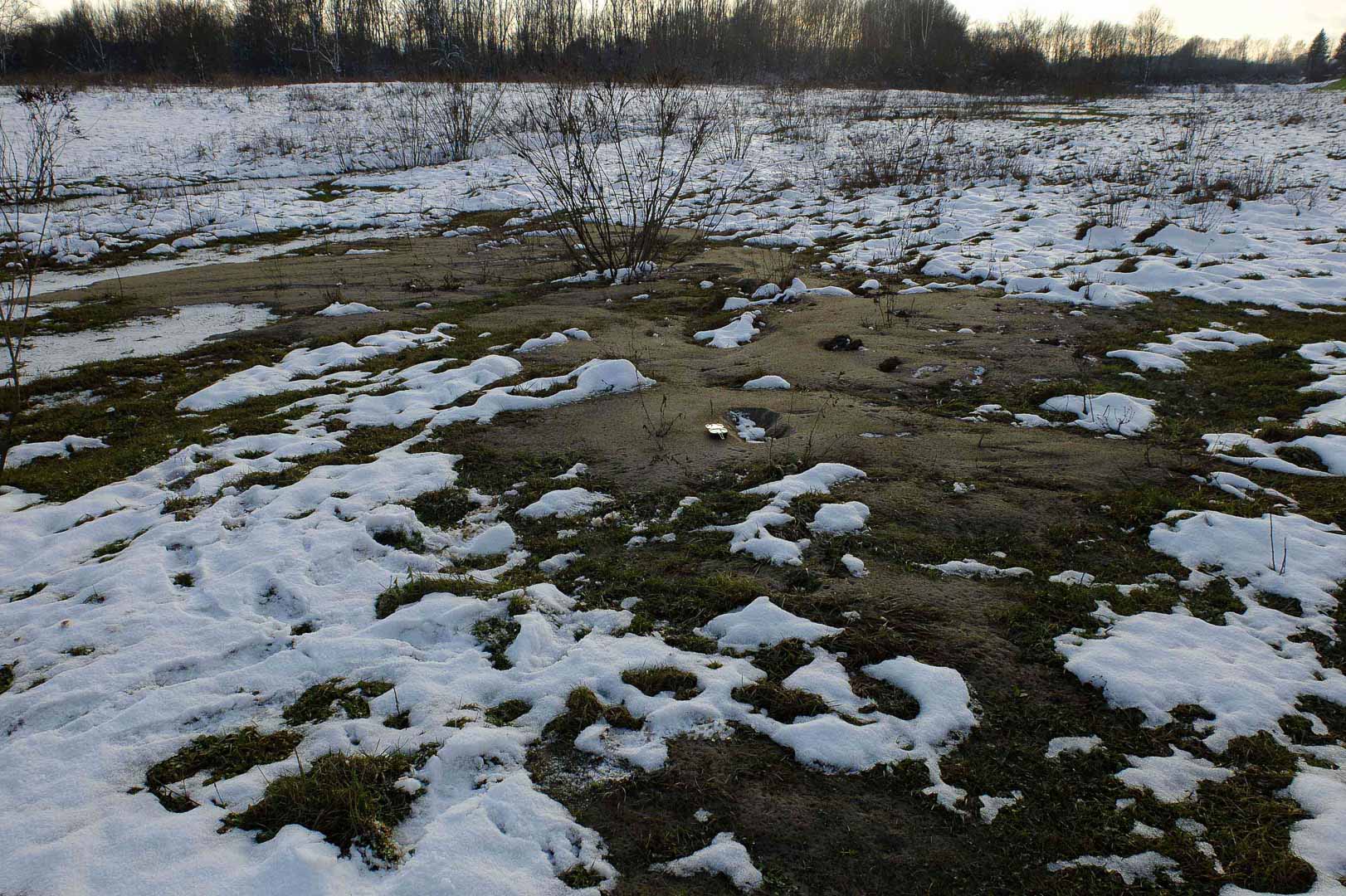

Supplement: Supplementary file 3 — Supplementary Information 3. [file 41598_2021_88378_MOESM3_ESM.zip › 160 (15-01-2021).jpg]

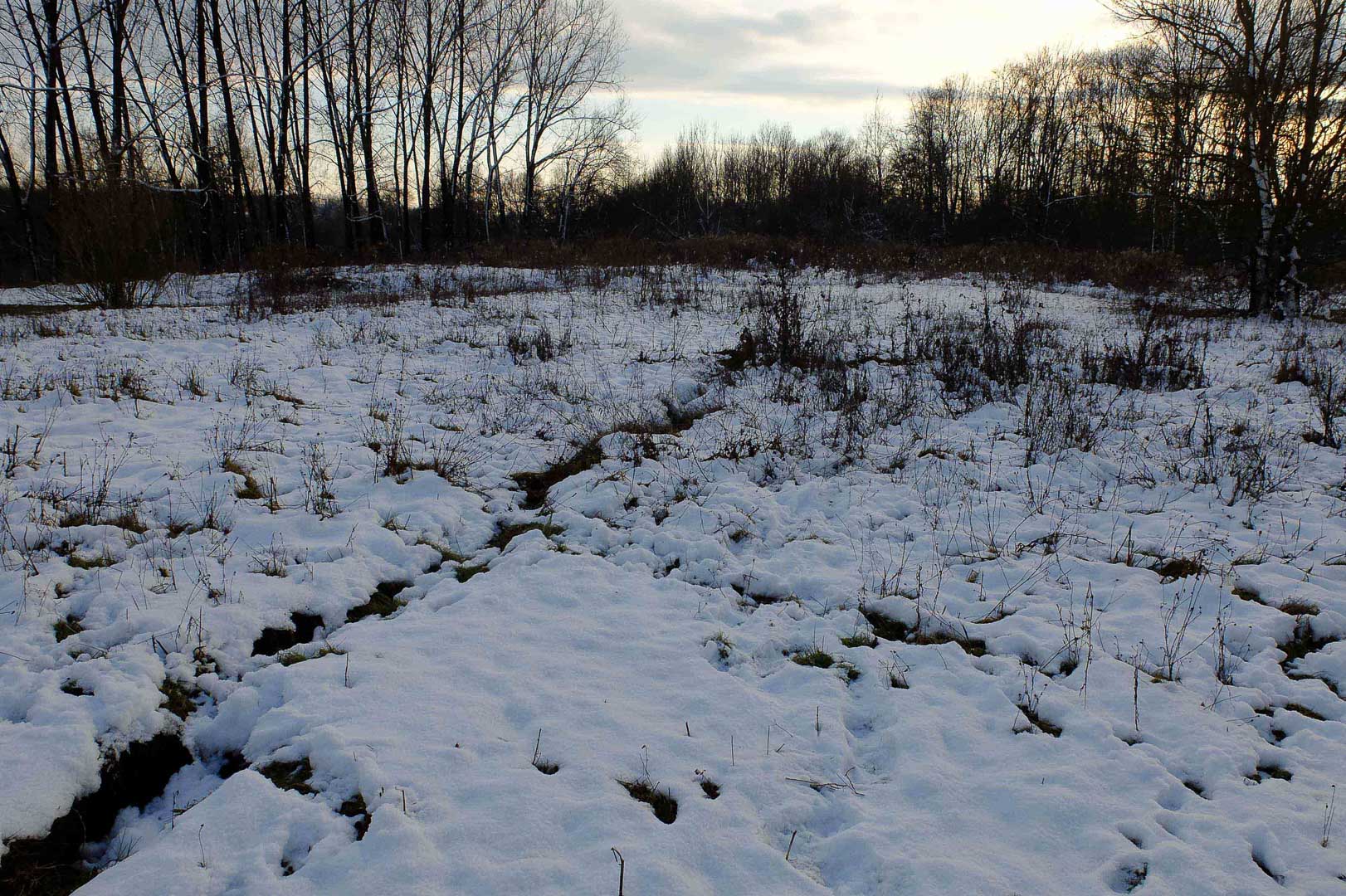

Supplement: Supplementary file 3 — Supplementary Information 3. [file 41598_2021_88378_MOESM3_ESM.zip › 161 (15-01-2021).jpg]

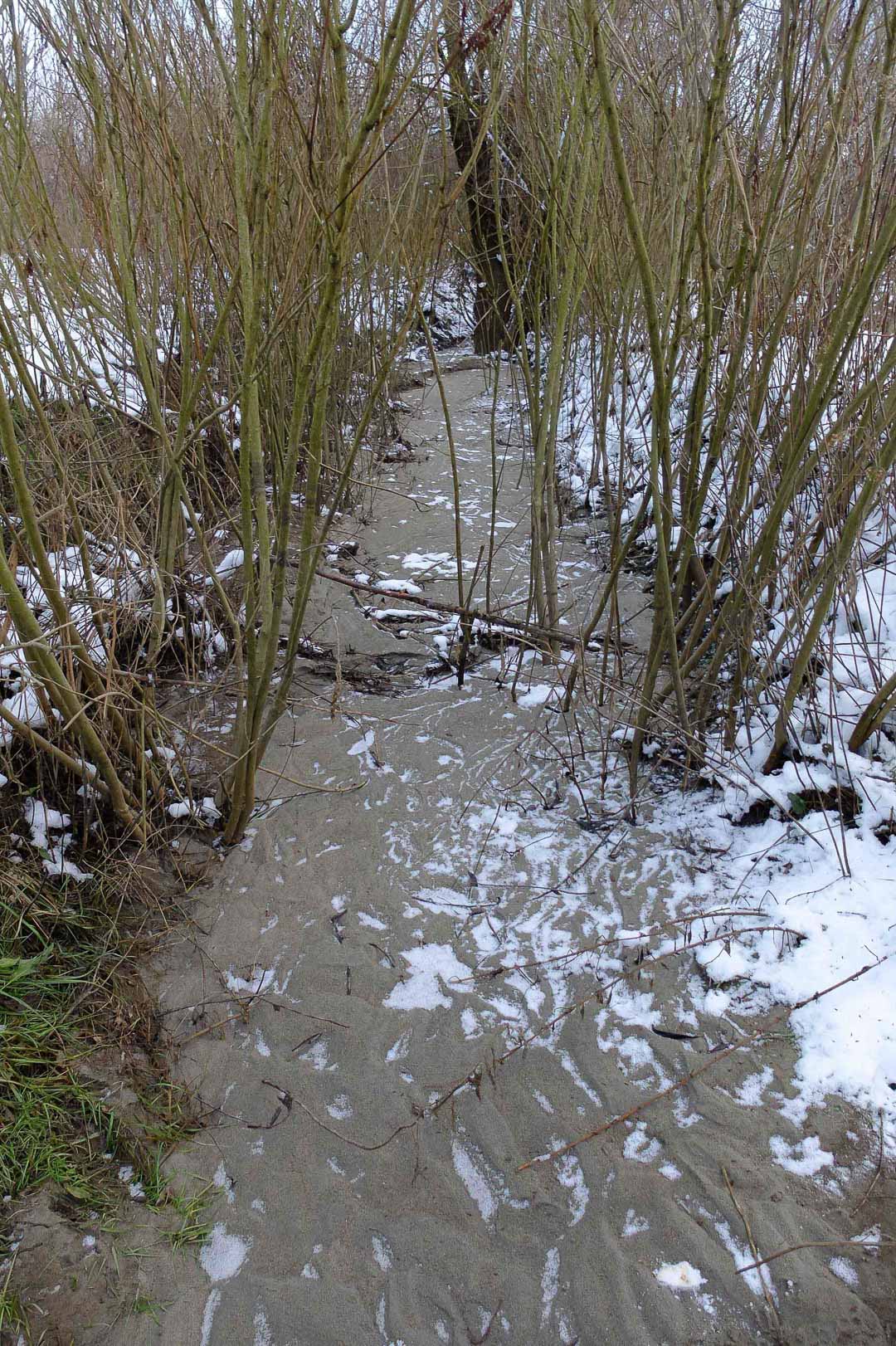

Supplement: Supplementary file 3 — Supplementary Information 3. [file 41598_2021_88378_MOESM3_ESM.zip › 164 (15-01-2021).jpg]

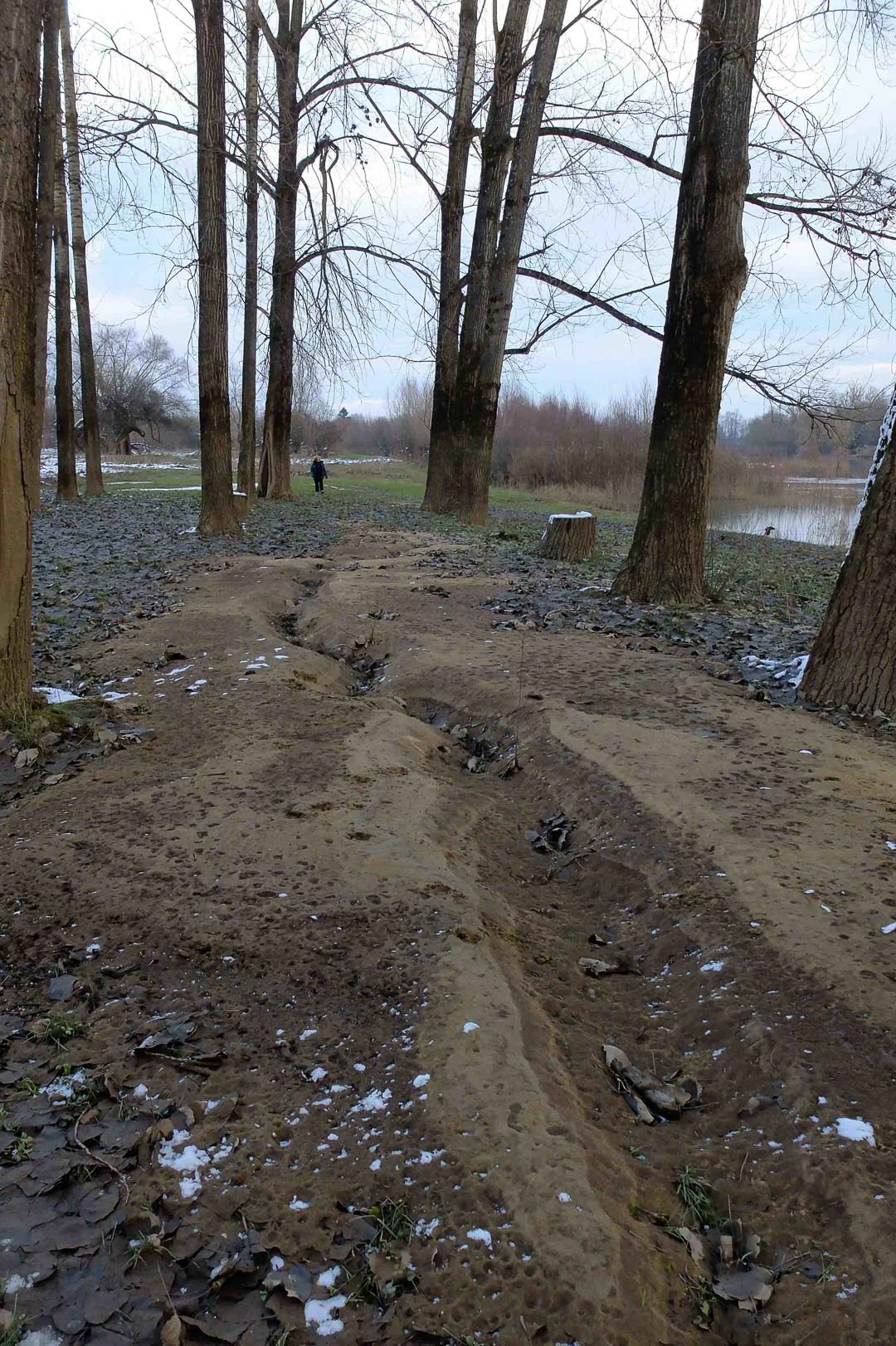

Supplement: Supplementary file 3 — Supplementary Information 3. [file 41598_2021_88378_MOESM3_ESM.zip › 165a (15-01-2021).jpg]

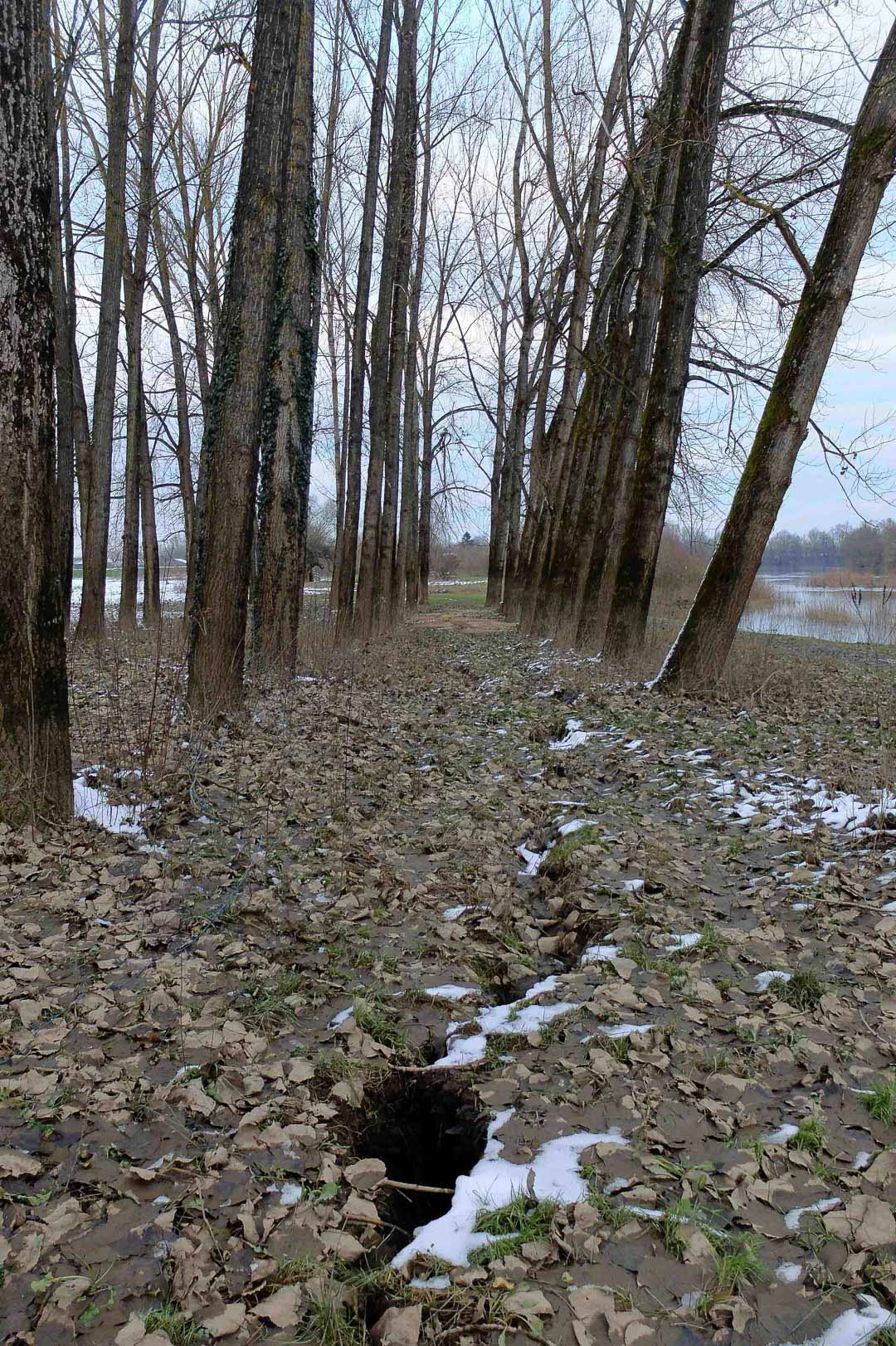

Supplement: Supplementary file 3 — Supplementary Information 3. [file 41598_2021_88378_MOESM3_ESM.zip › 165b (15-01-2021).jpg]

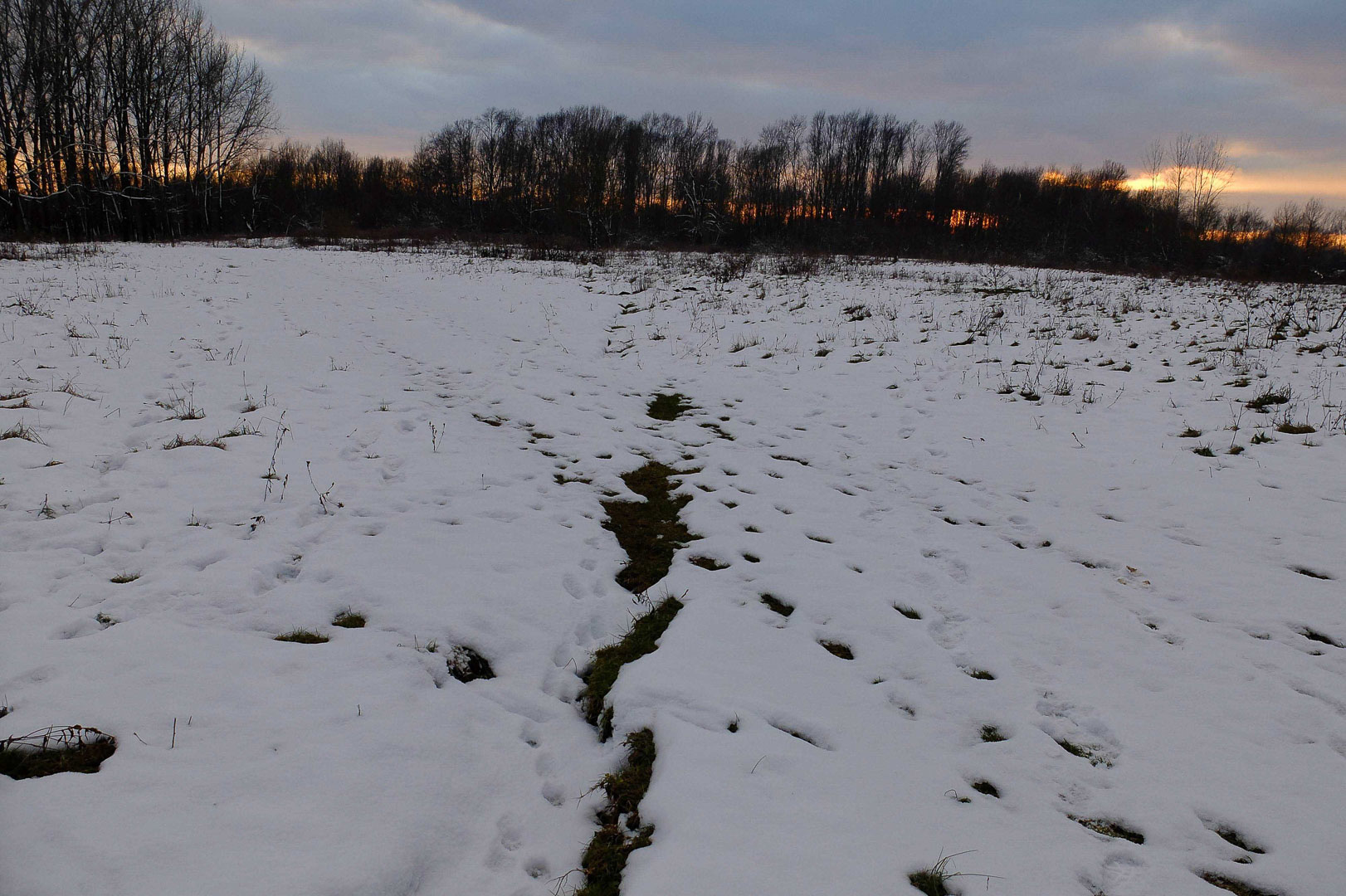

Supplement: Supplementary file 3 — Supplementary Information 3. [file 41598_2021_88378_MOESM3_ESM.zip › 167 (15-01-2021).jpg]

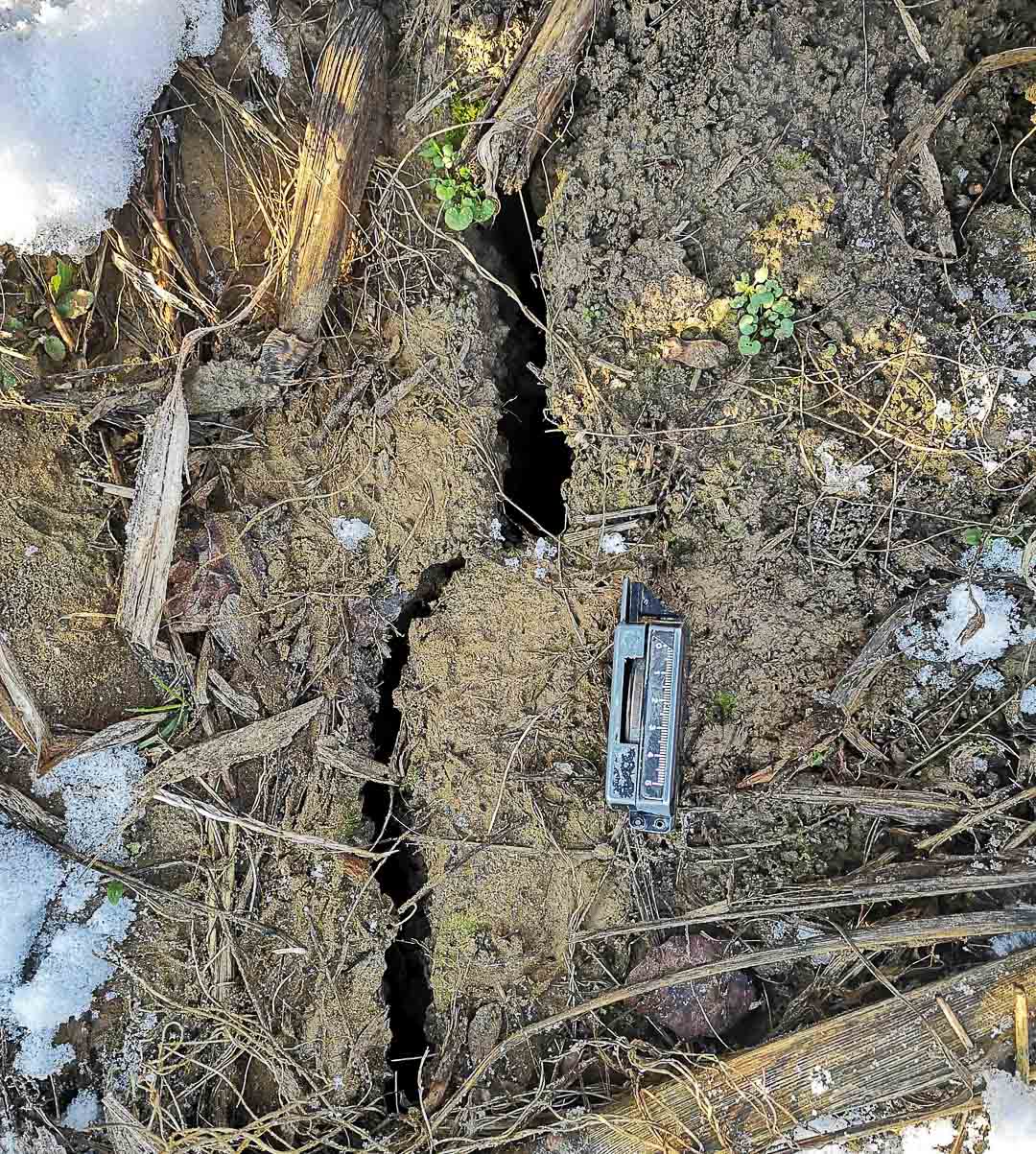

Supplement: Supplementary file 3 — Supplementary Information 3. [file 41598_2021_88378_MOESM3_ESM.zip › 173 (13-01-2021).jpg]

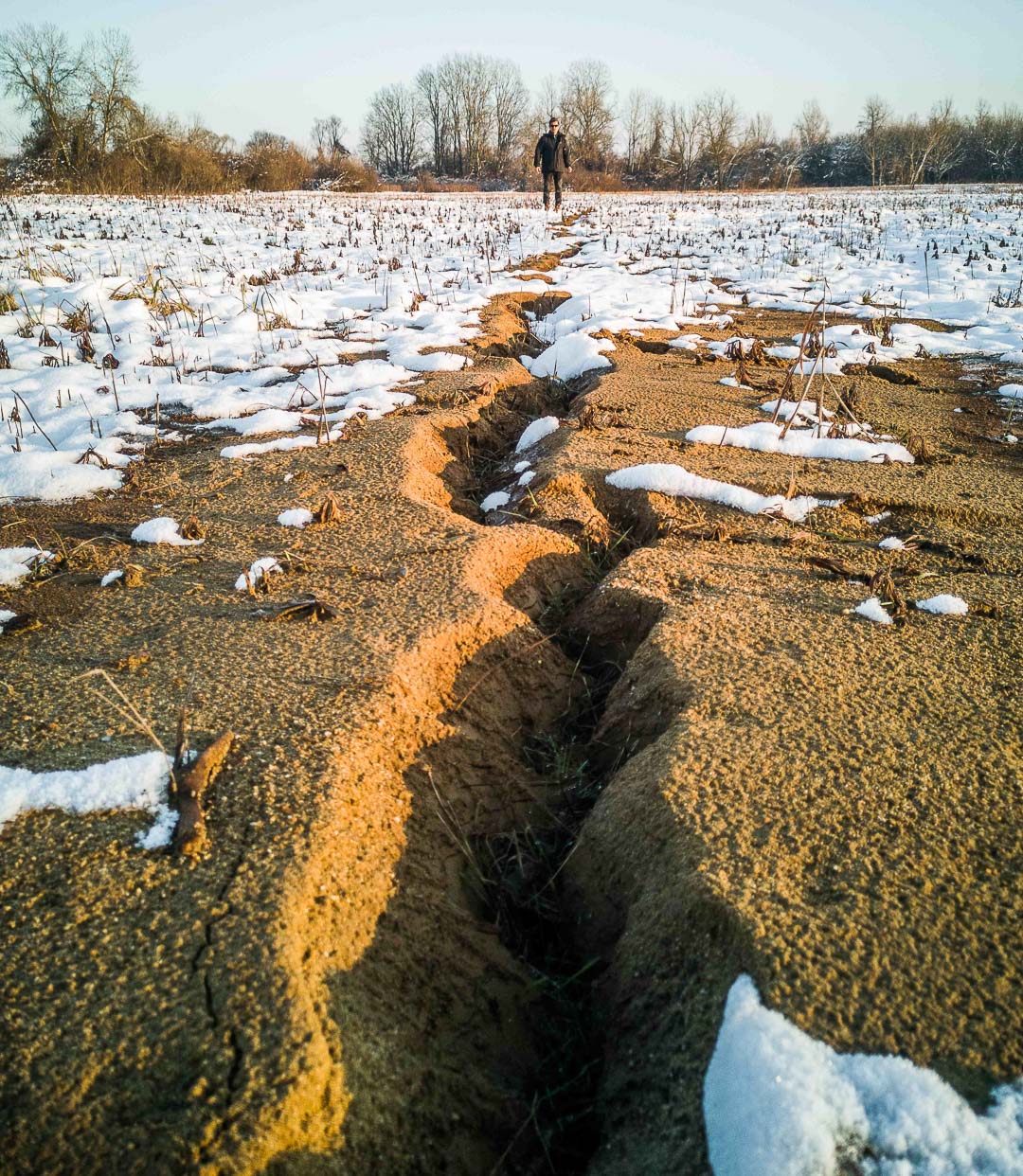

Supplement: Supplementary file 3 — Supplementary Information 3. [file 41598_2021_88378_MOESM3_ESM.zip › 174a (13-01-2021).jpg]

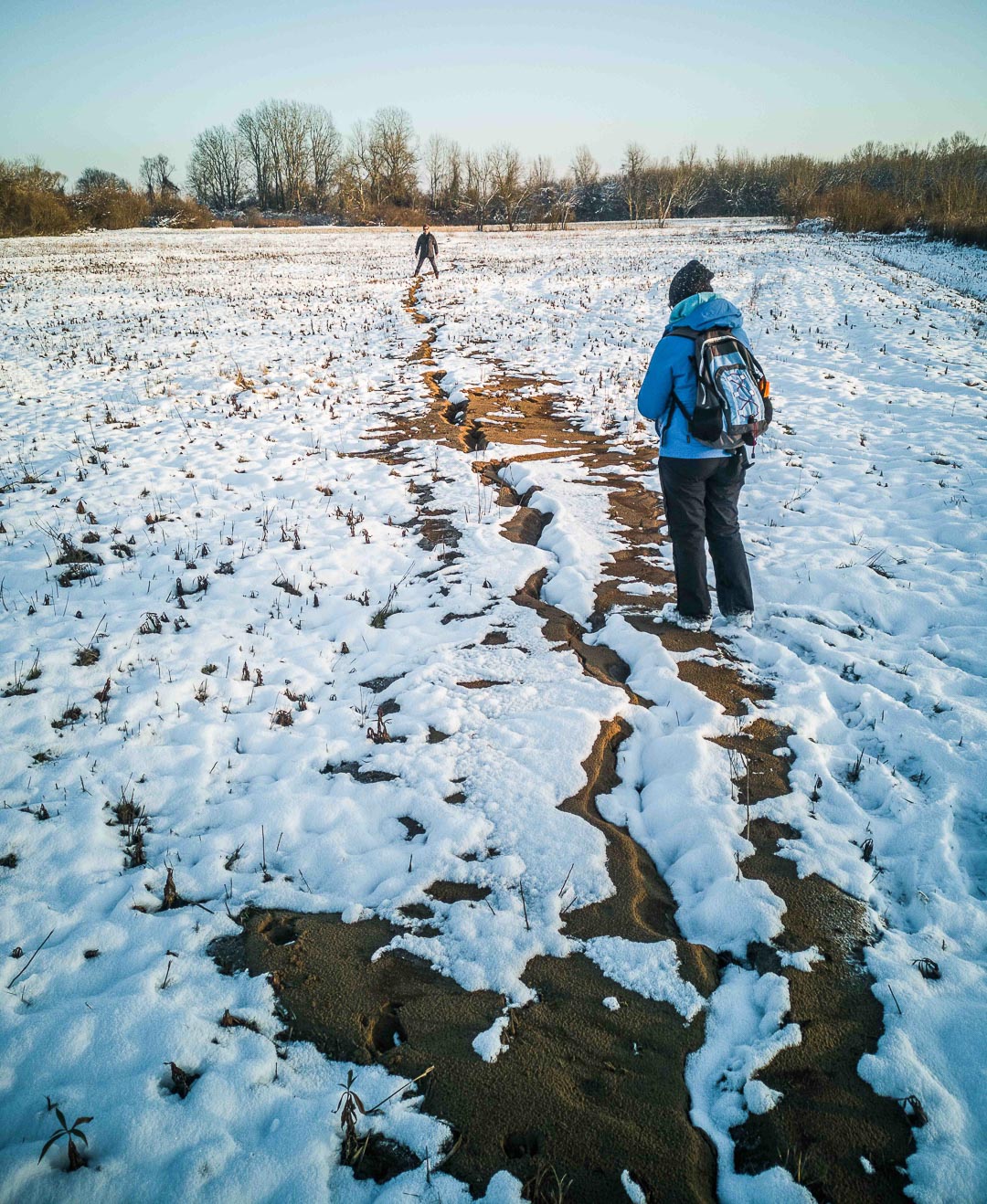

Supplement: Supplementary file 3 — Supplementary Information 3. [file 41598_2021_88378_MOESM3_ESM.zip › 174b (13-01-2021).jpg]

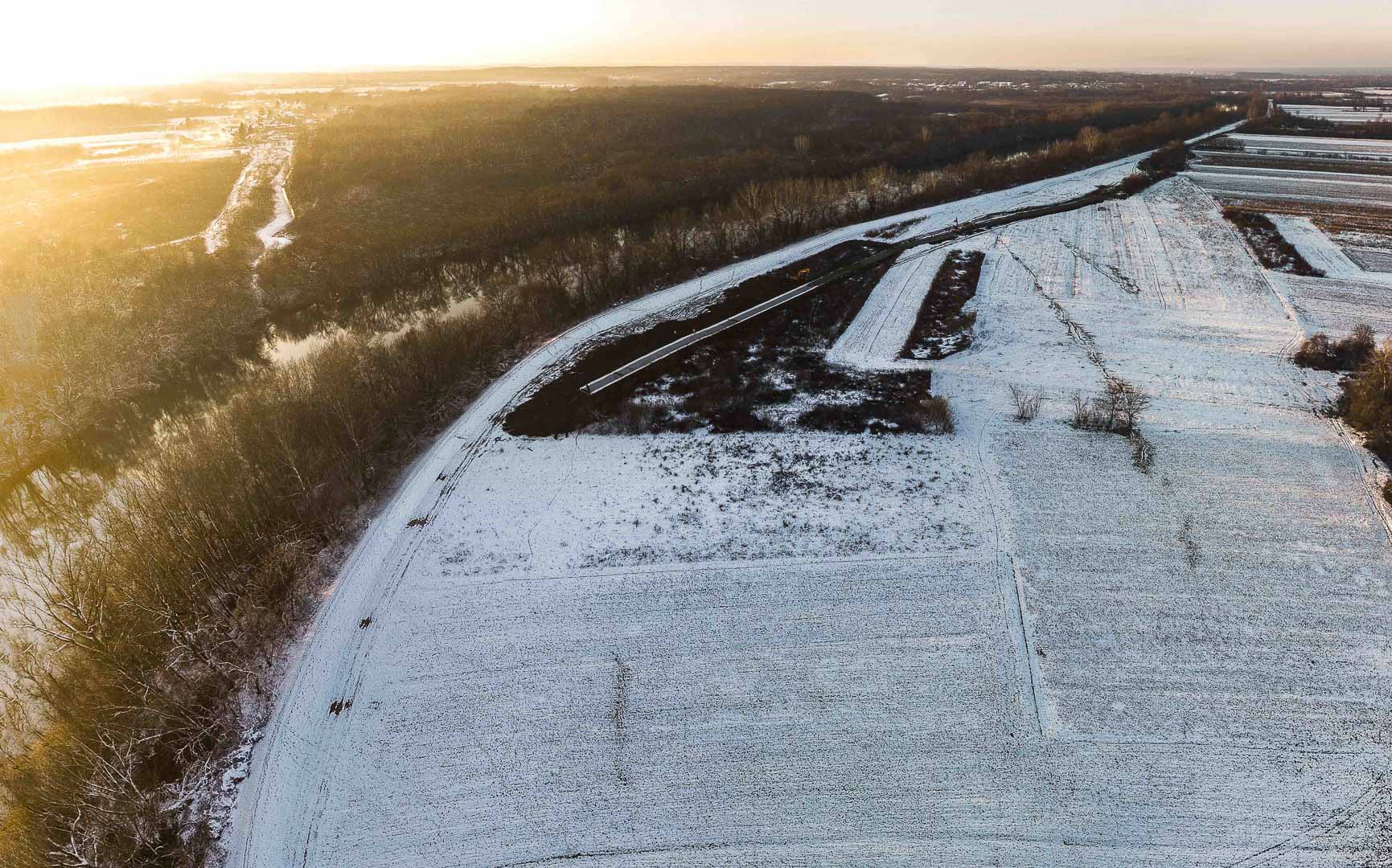

Supplement: Supplementary file 3 — Supplementary Information 3. [file 41598_2021_88378_MOESM3_ESM.zip › 175 (13-01-2021).jpg]

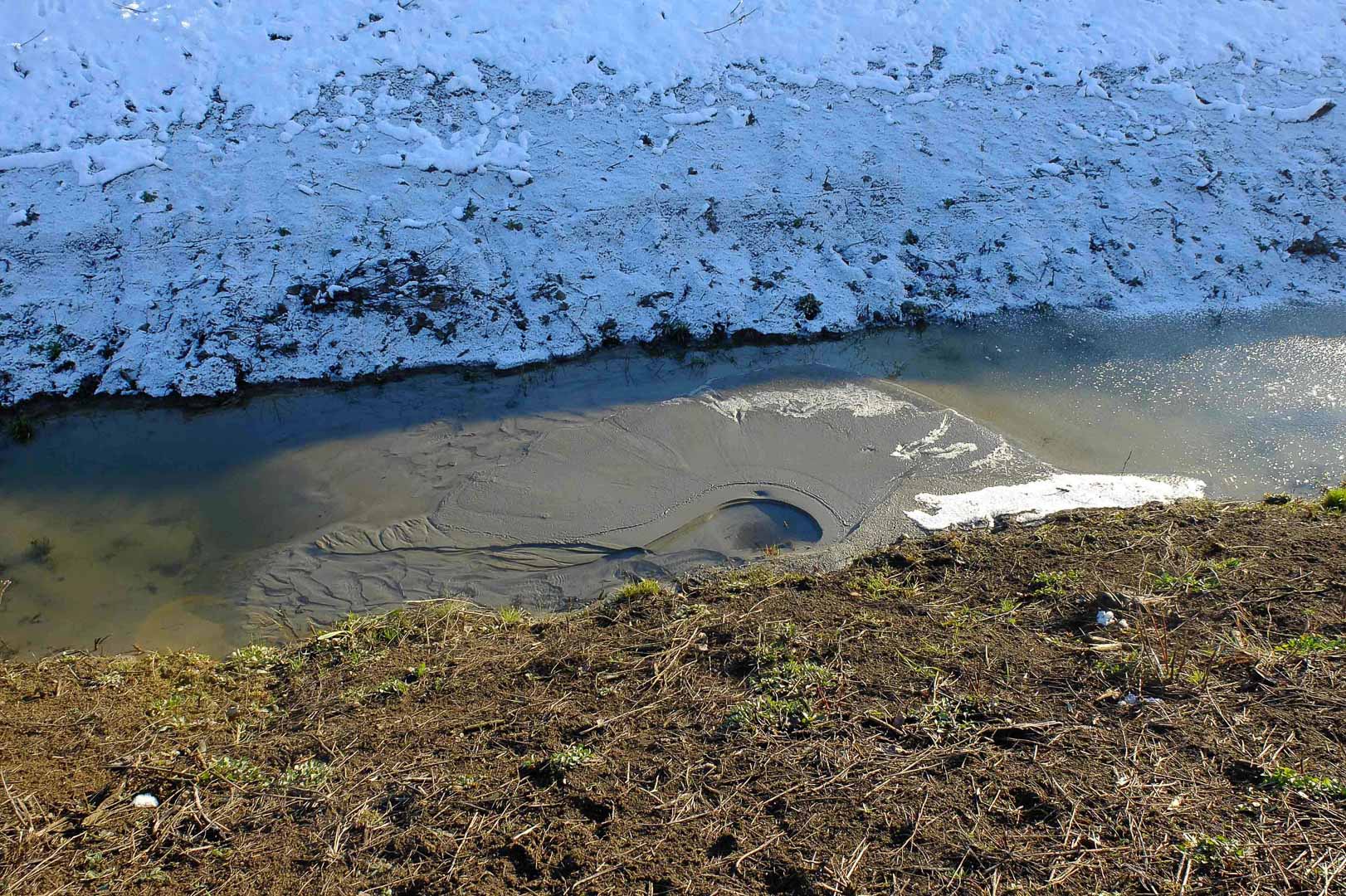

Supplement: Supplementary file 3 — Supplementary Information 3. [file 41598_2021_88378_MOESM3_ESM.zip › 176 (15-01-2021).jpg]

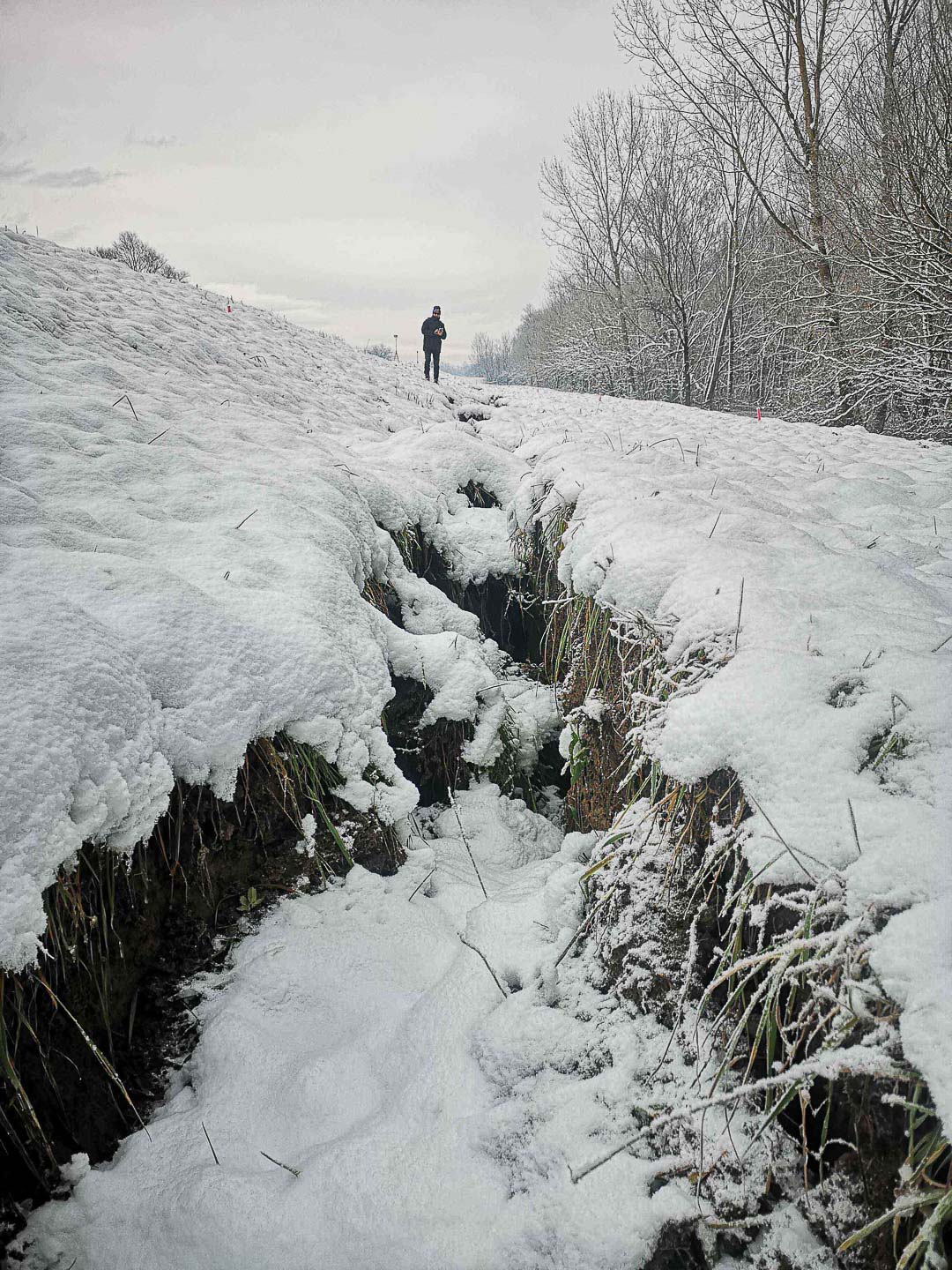

Supplement: Supplementary file 3 — Supplementary Information 3. [file 41598_2021_88378_MOESM3_ESM.zip › 177 (14-01-2021).jpg]

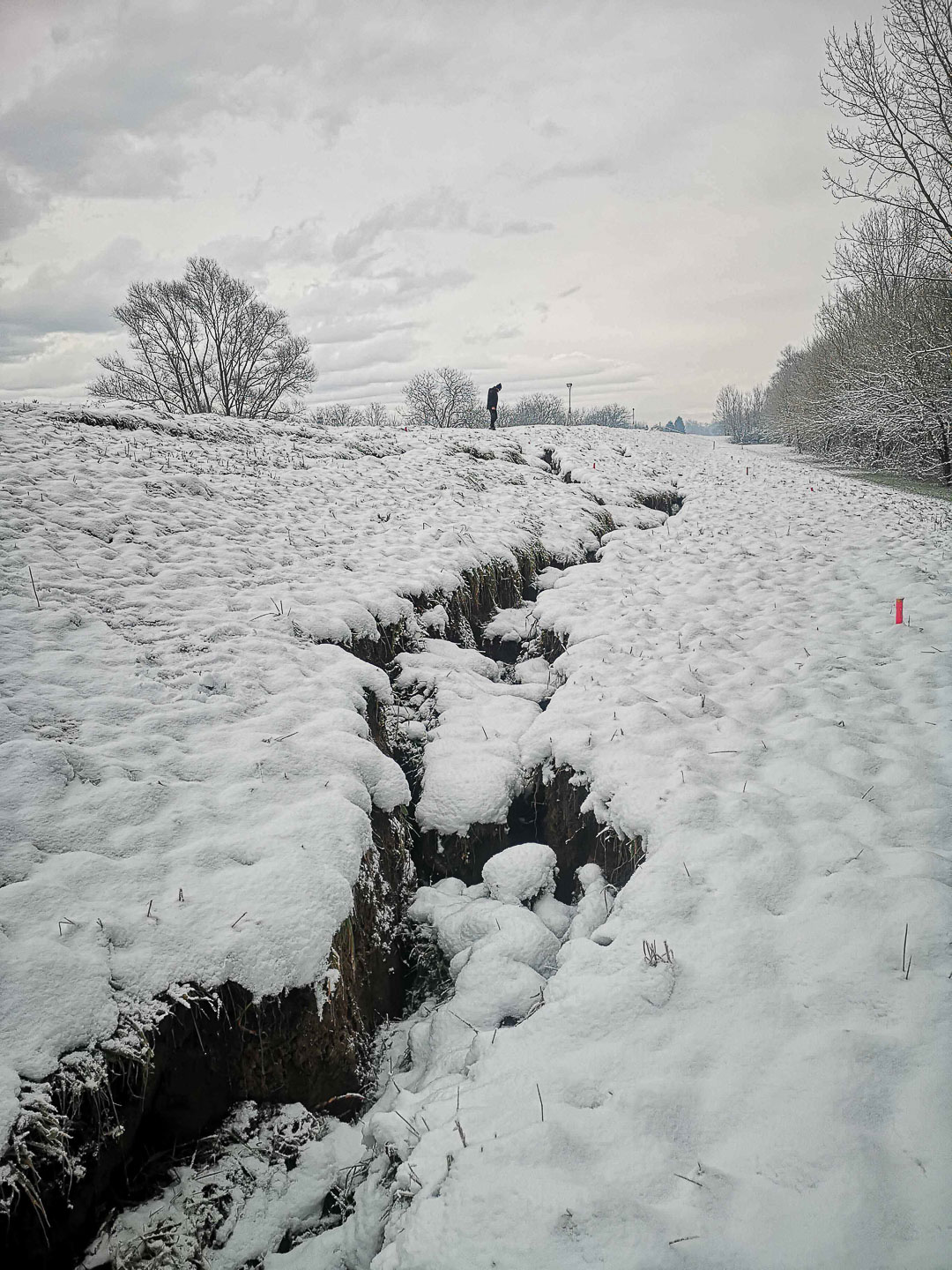

Supplement: Supplementary file 3 — Supplementary Information 3. [file 41598_2021_88378_MOESM3_ESM.zip › 178 (14-01-2021).jpg]

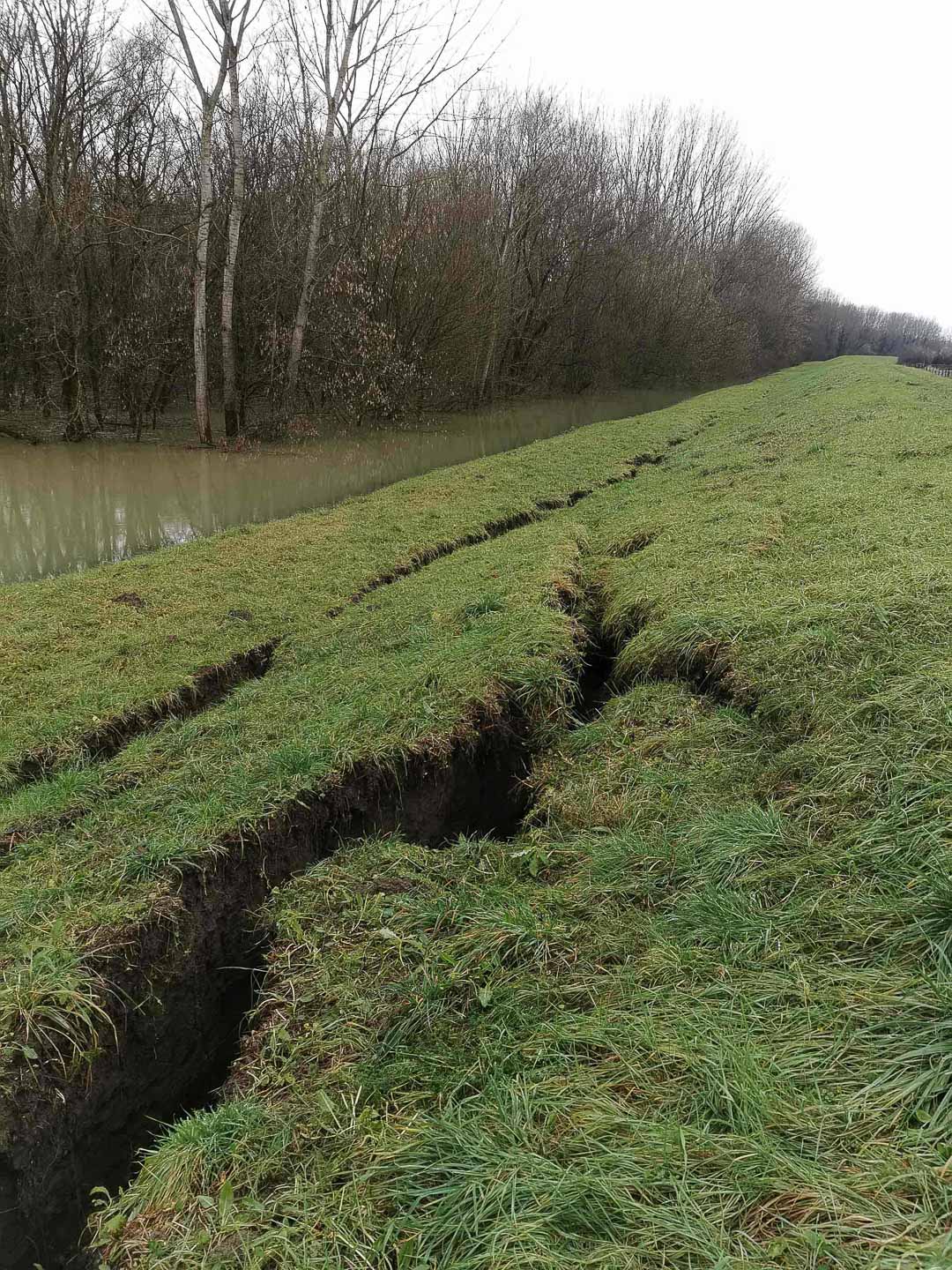

Supplement: Supplementary file 3 — Supplementary Information 3. [file 41598_2021_88378_MOESM3_ESM.zip › 179 (31-12-2020).jpg]

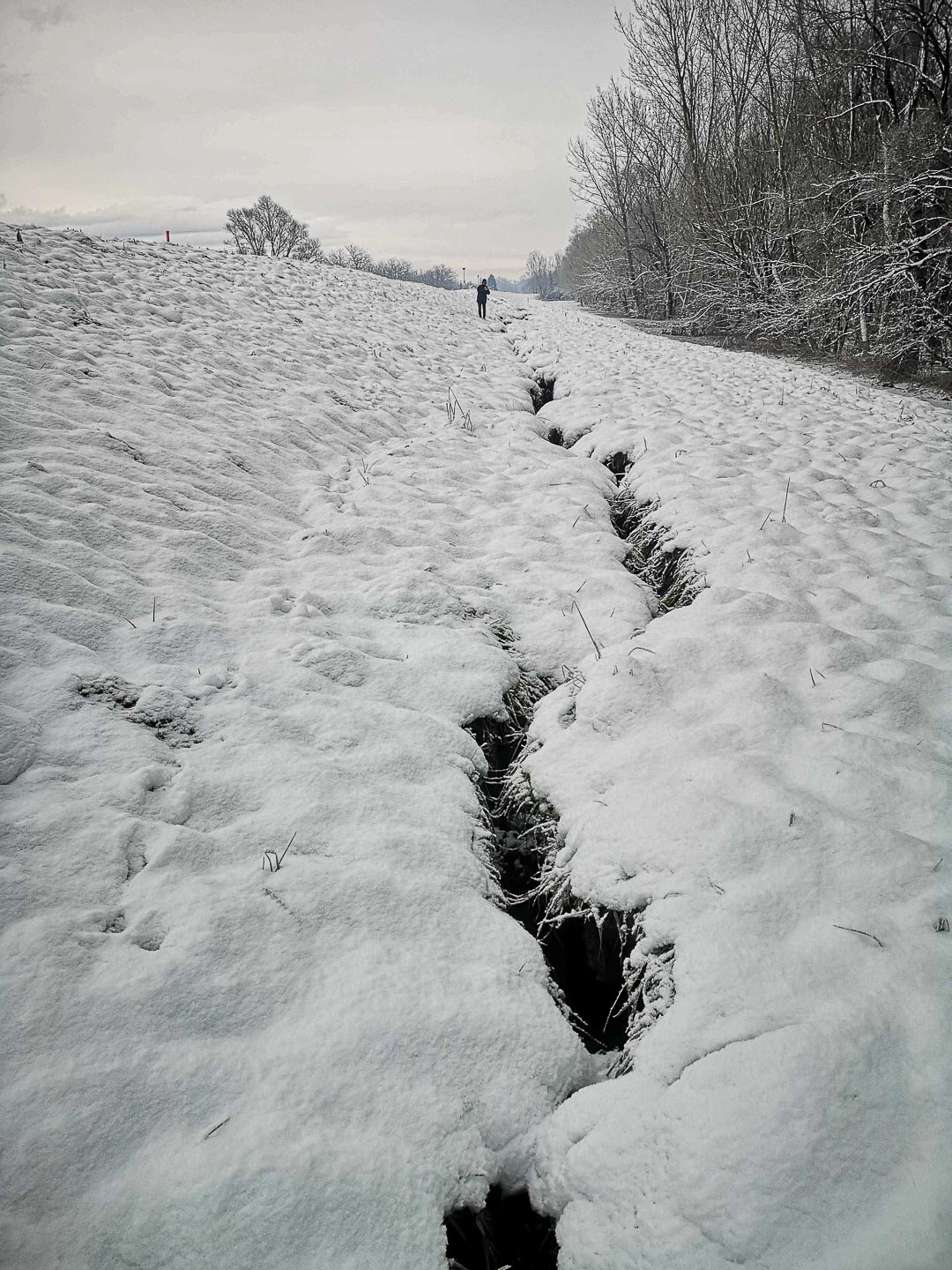

Supplement: Supplementary file 3 — Supplementary Information 3. [file 41598_2021_88378_MOESM3_ESM.zip › 180 (14-01-2021).jpg]

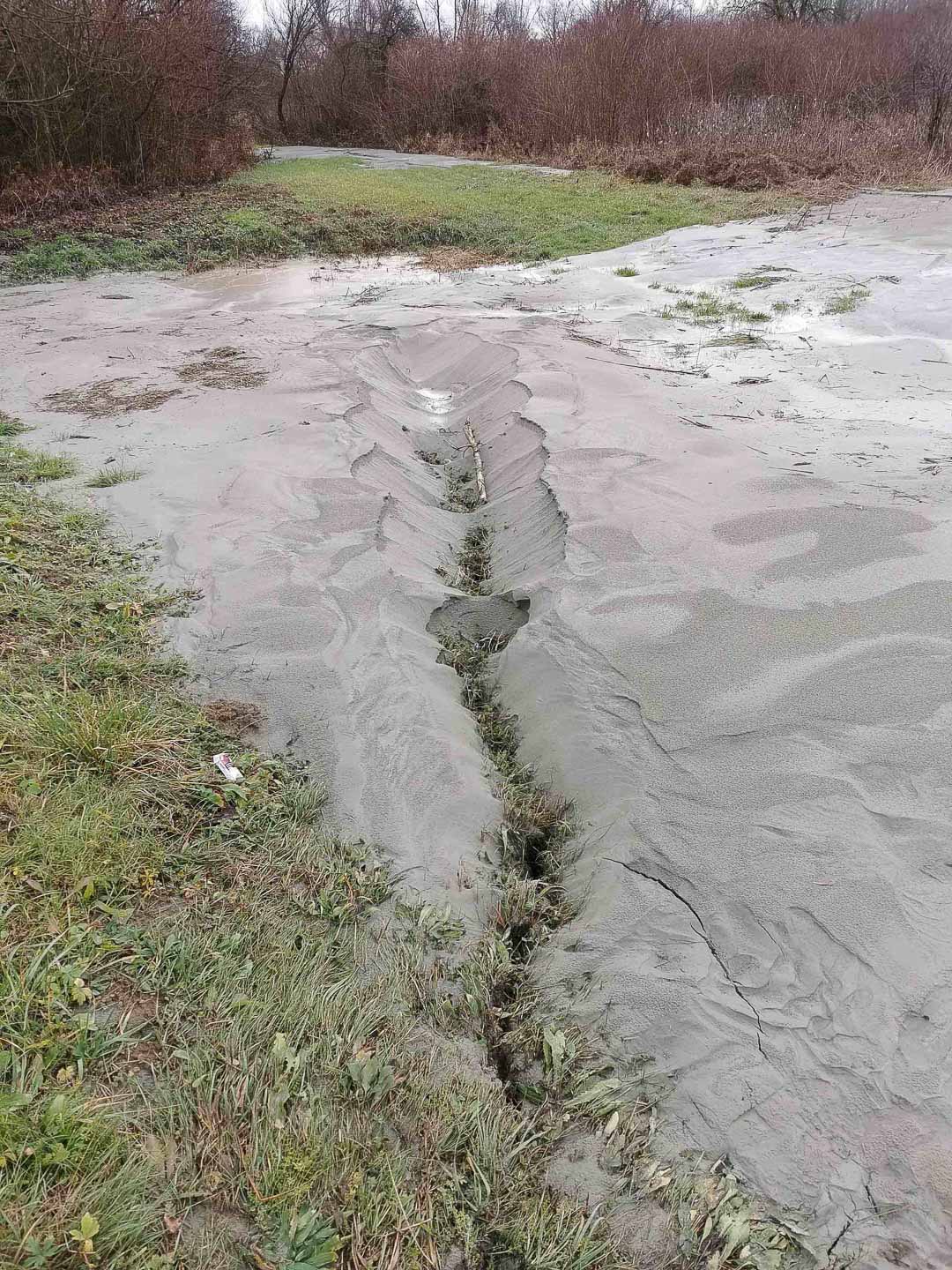

Supplement: Supplementary file 3 — Supplementary Information 3. [file 41598_2021_88378_MOESM3_ESM.zip › 185 (30-12-2020).jpg]

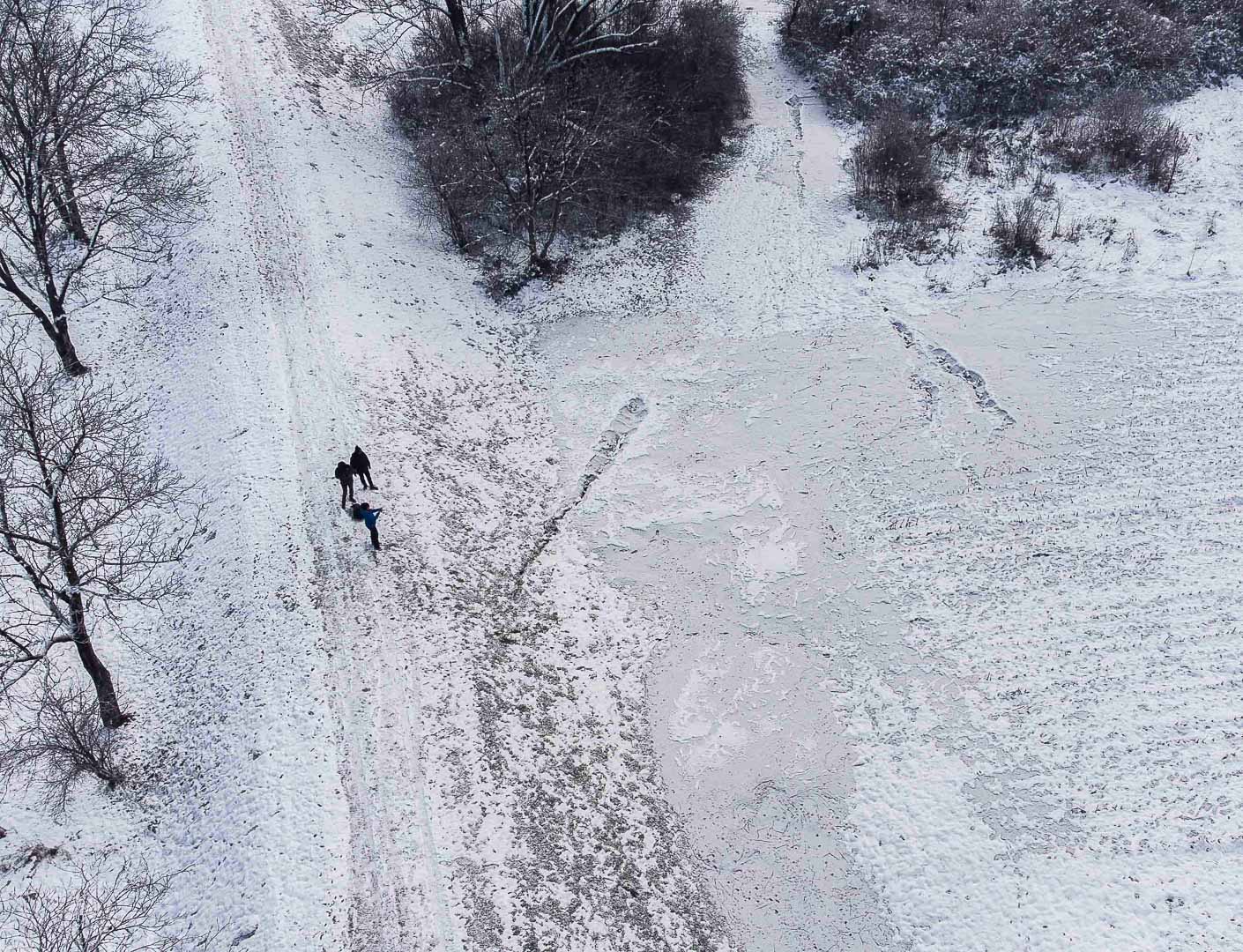

Supplement: Supplementary file 3 — Supplementary Information 3. [file 41598_2021_88378_MOESM3_ESM.zip › 188 (14-01-2021).jpg]

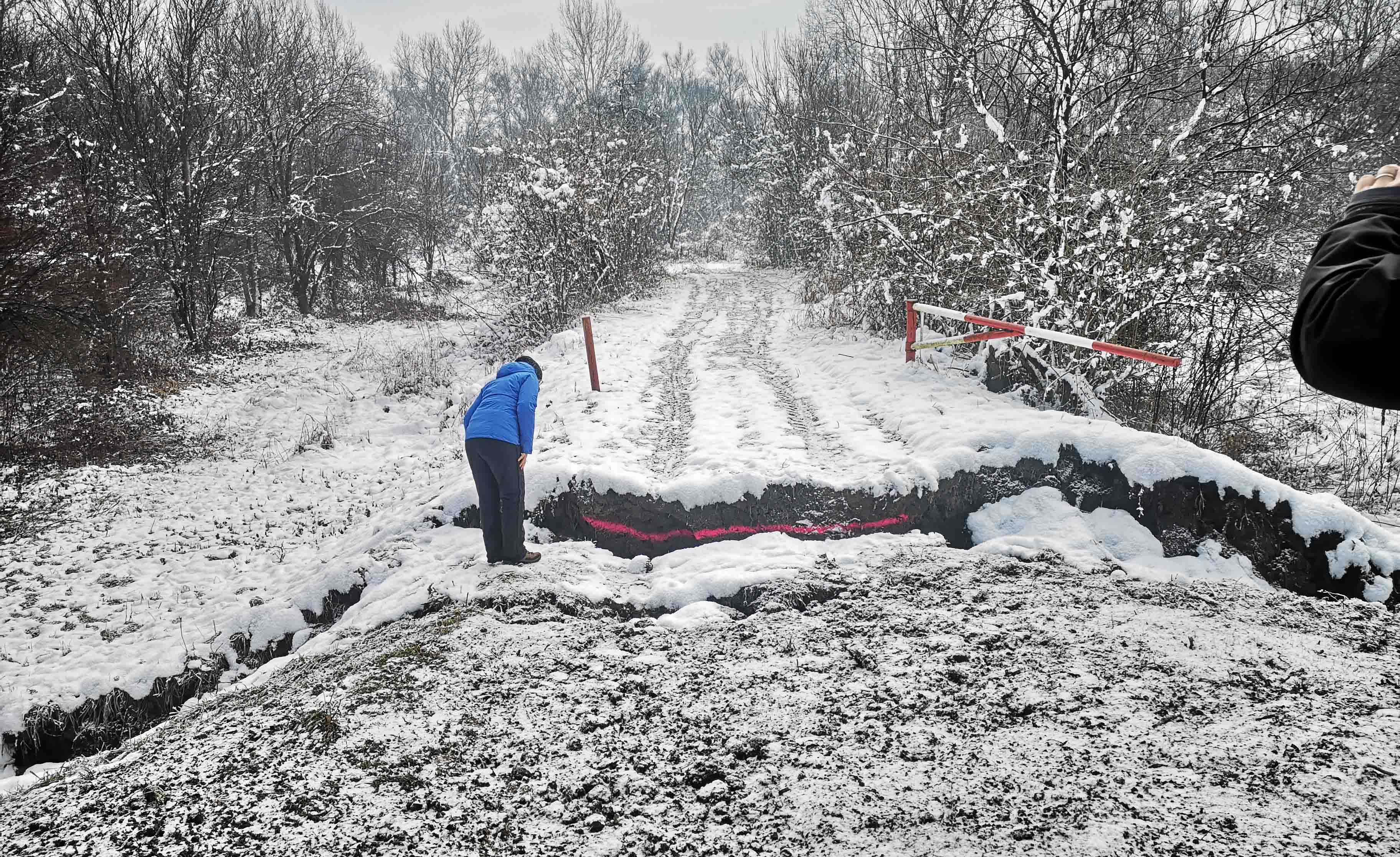

Supplement: Supplementary file 3 — Supplementary Information 3. [file 41598_2021_88378_MOESM3_ESM.zip › 189 (14-01-2021).jpg]

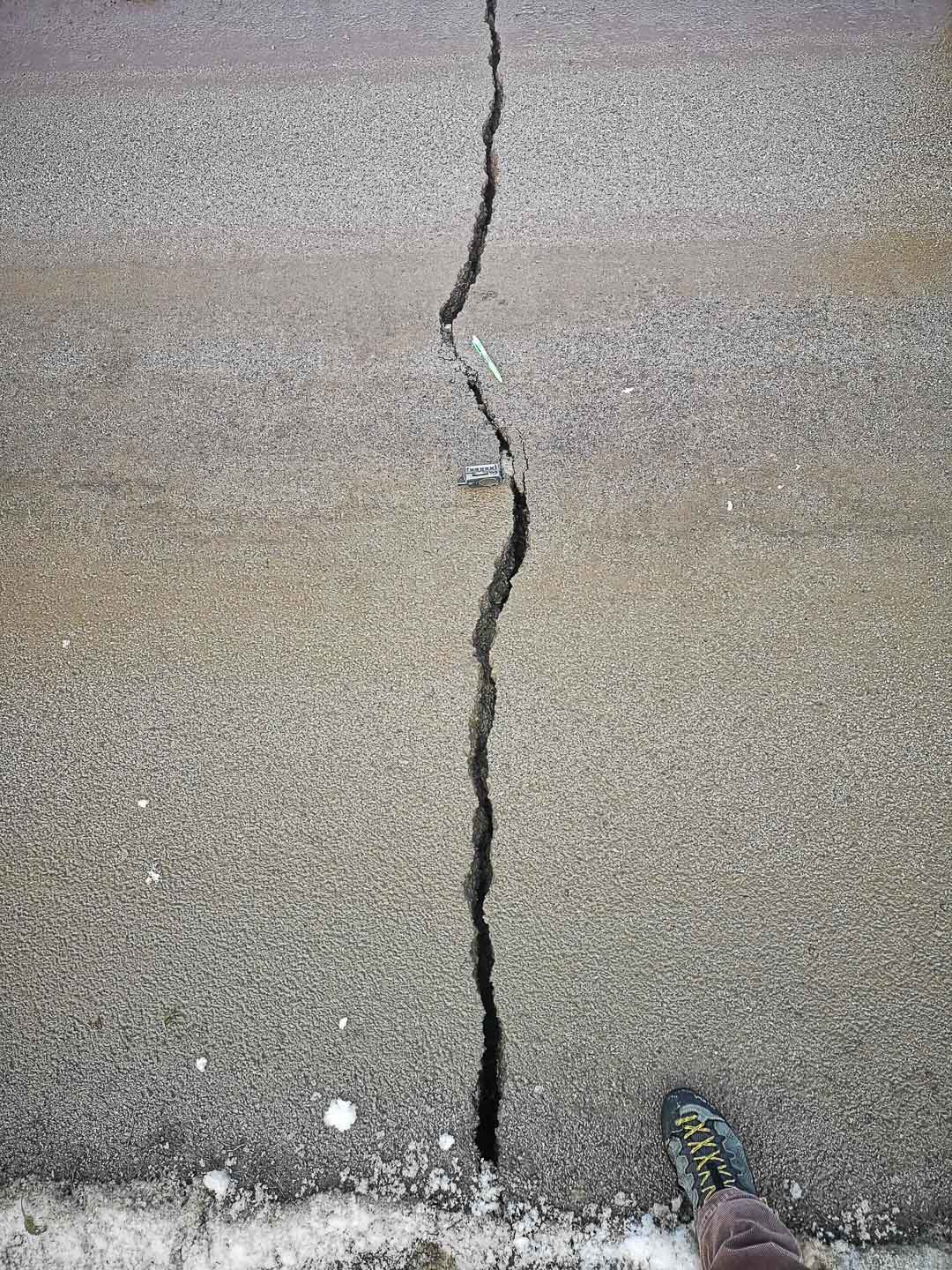

Supplement: Supplementary file 3 — Supplementary Information 3. [file 41598_2021_88378_MOESM3_ESM.zip › 196 (14-01-2021).jpg]

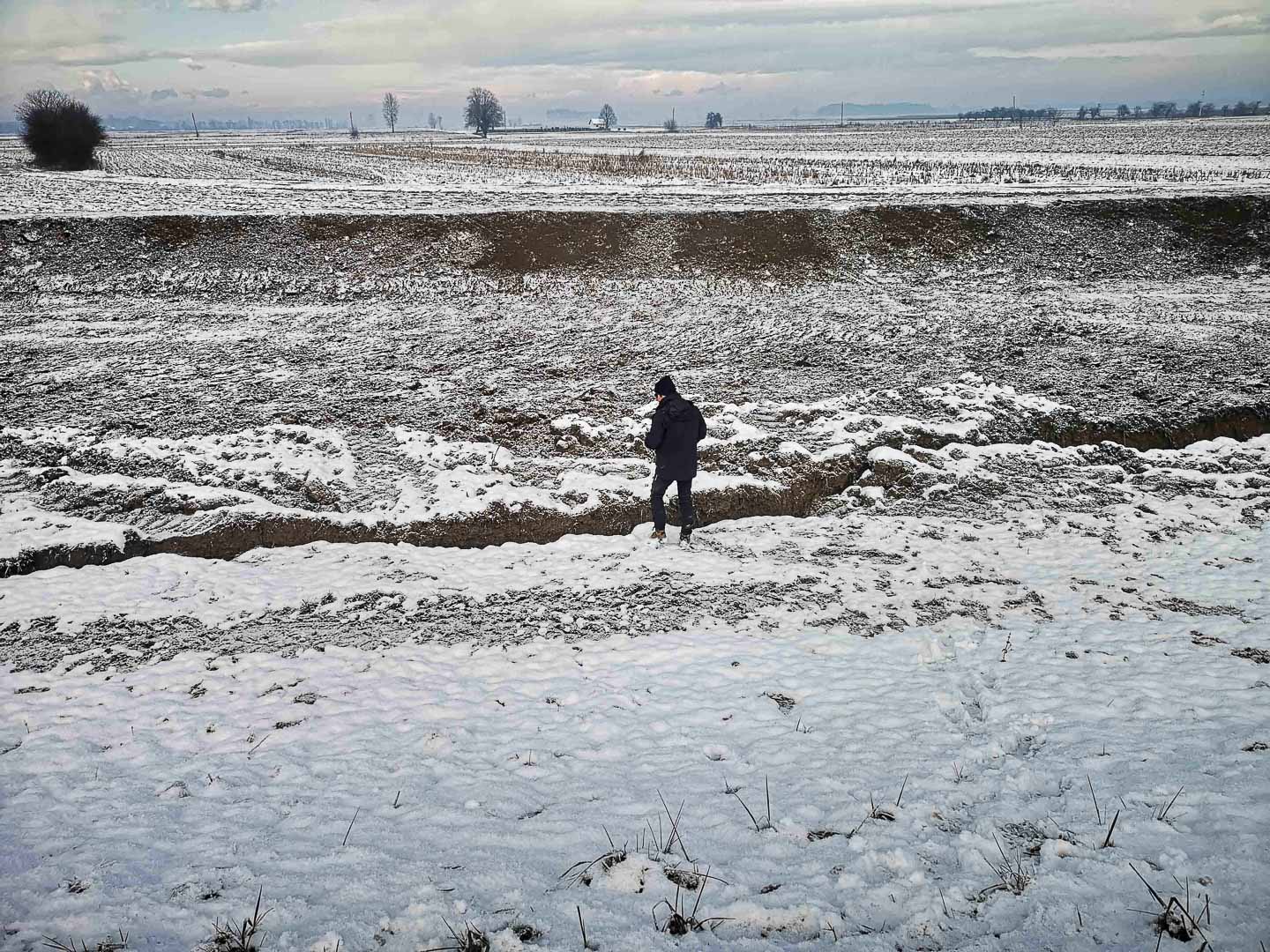

Supplement: Supplementary file 3 — Supplementary Information 3. [file 41598_2021_88378_MOESM3_ESM.zip › 197 (14-01-2021).jpg]

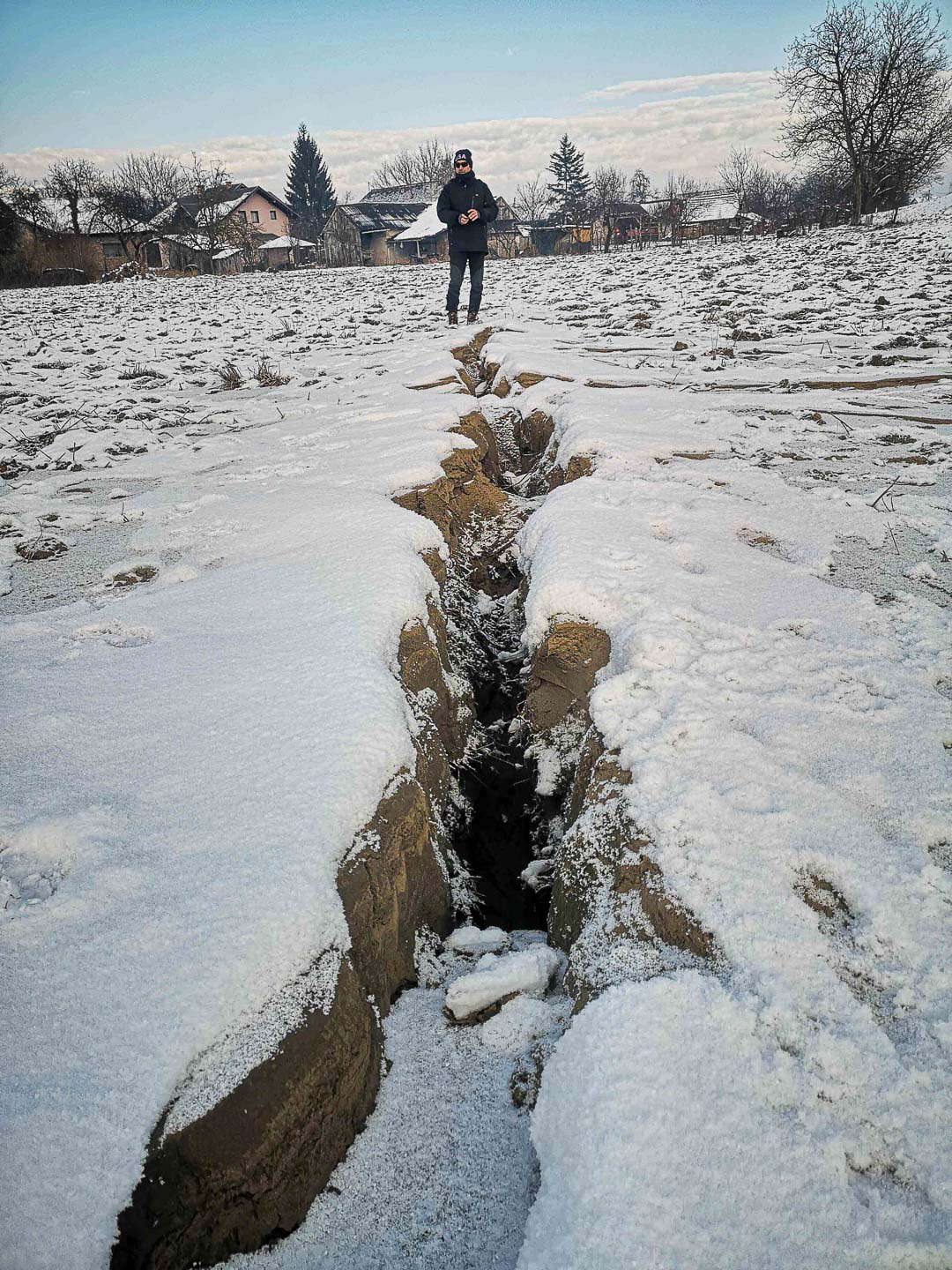

Supplement: Supplementary file 3 — Supplementary Information 3. [file 41598_2021_88378_MOESM3_ESM.zip › 199 (14-01-2021).jpg]

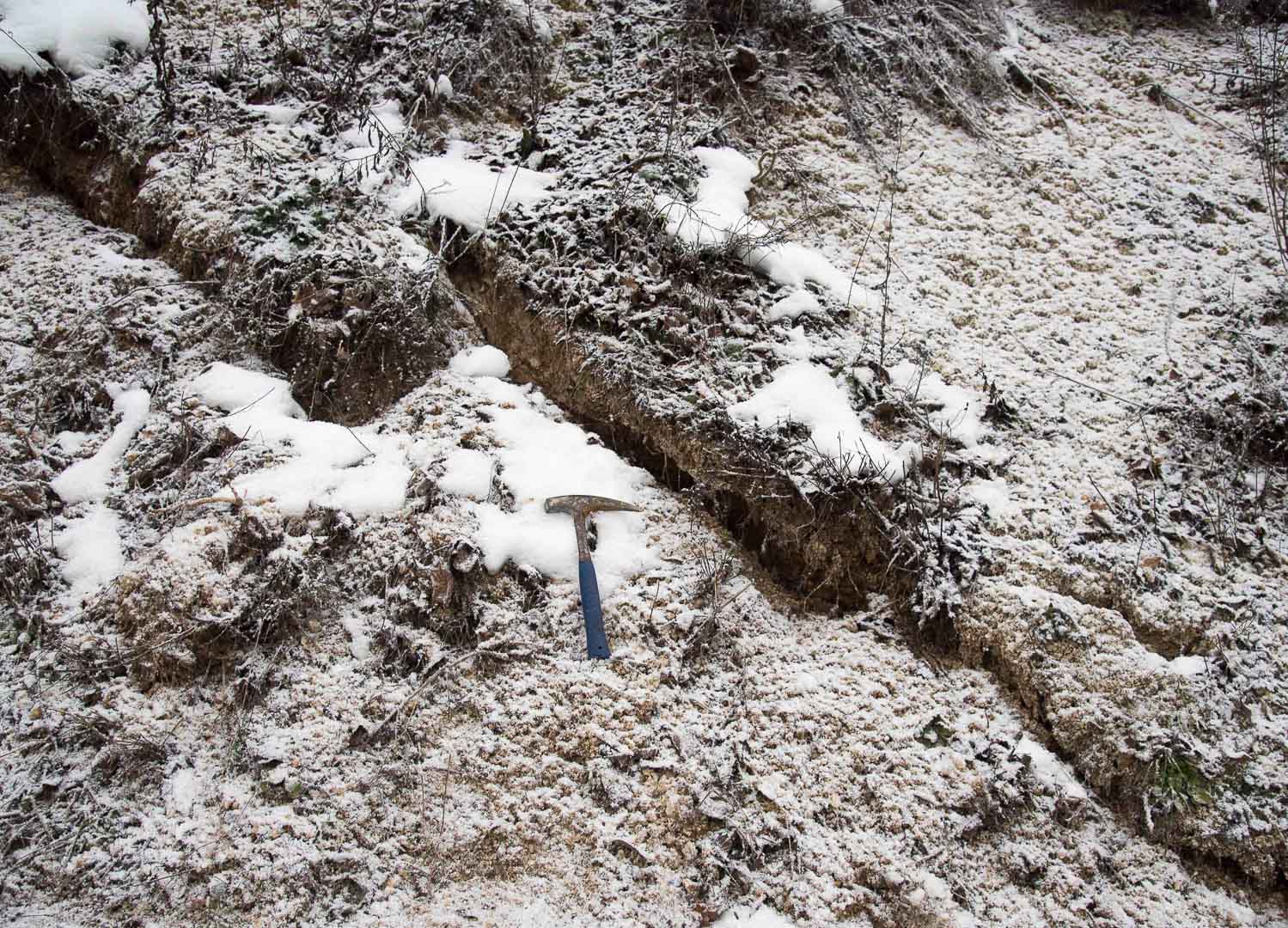

Supplement: Supplementary file 3 — Supplementary Information 3. [file 41598_2021_88378_MOESM3_ESM.zip › 1a (15-01-2021).jpg]

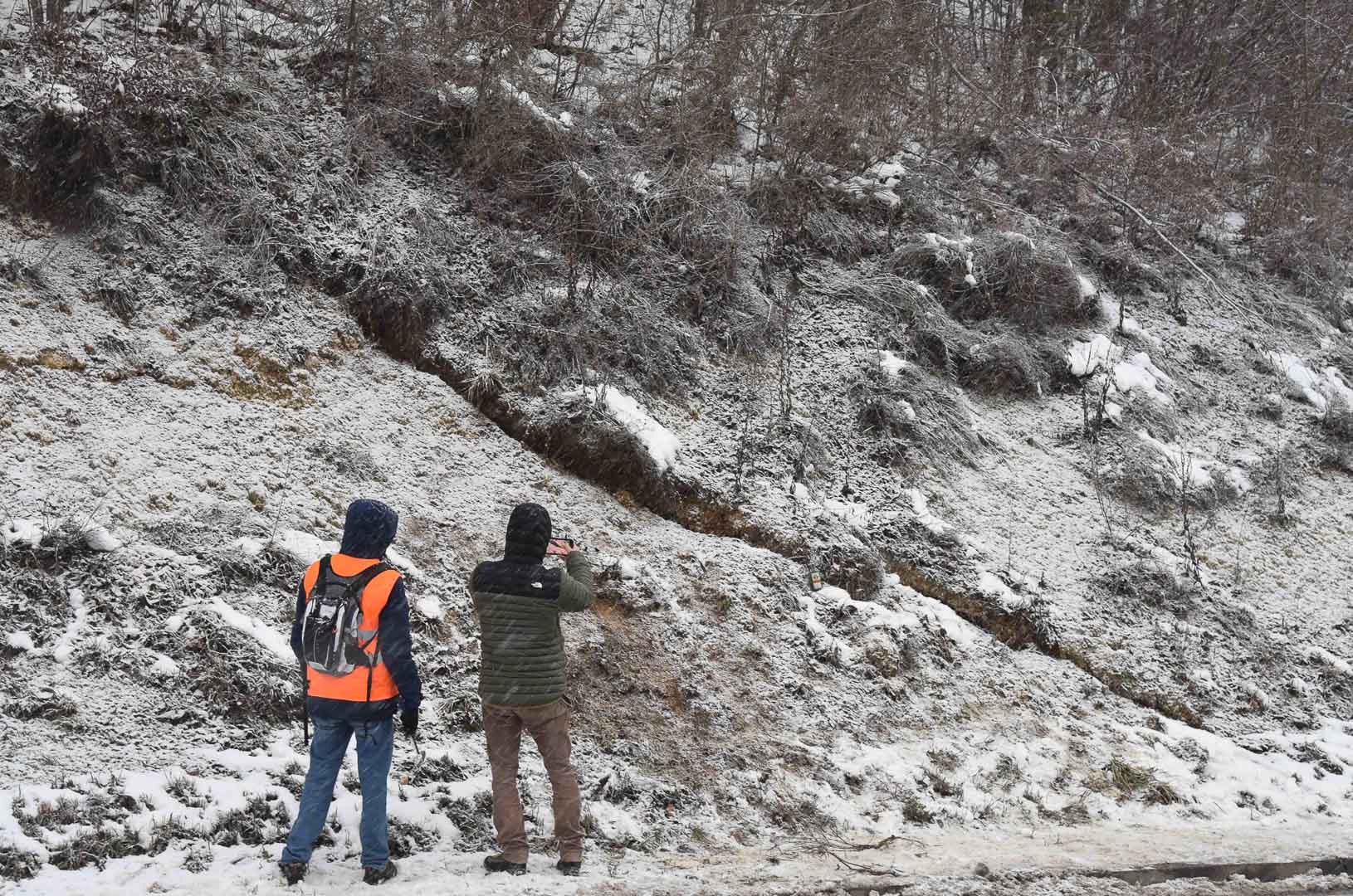

Supplement: Supplementary file 3 — Supplementary Information 3. [file 41598_2021_88378_MOESM3_ESM.zip › 1b (15-01-2021).jpg]

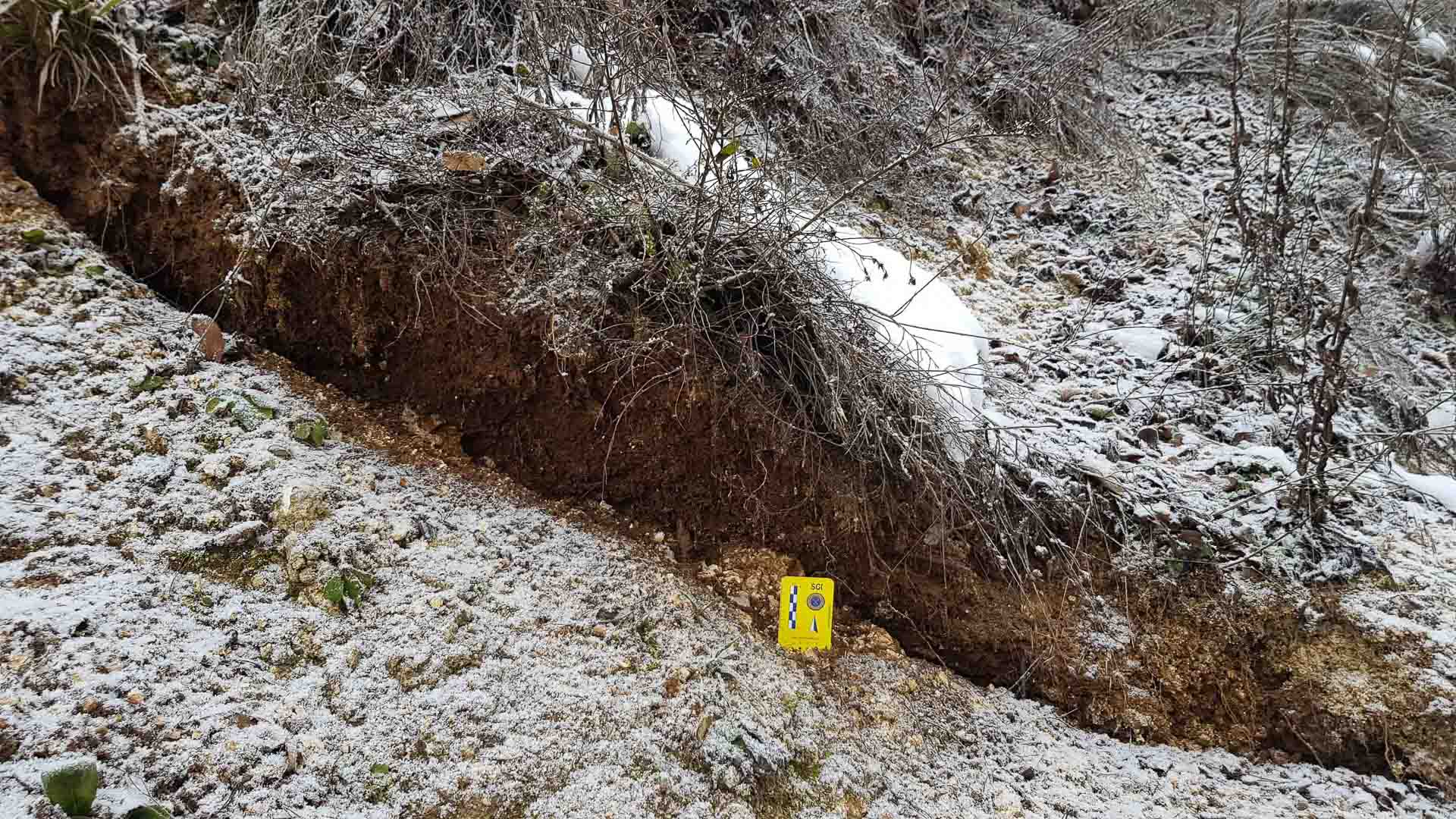

Supplement: Supplementary file 3 — Supplementary Information 3. [file 41598_2021_88378_MOESM3_ESM.zip › 1c (14-01-2021).jpg]

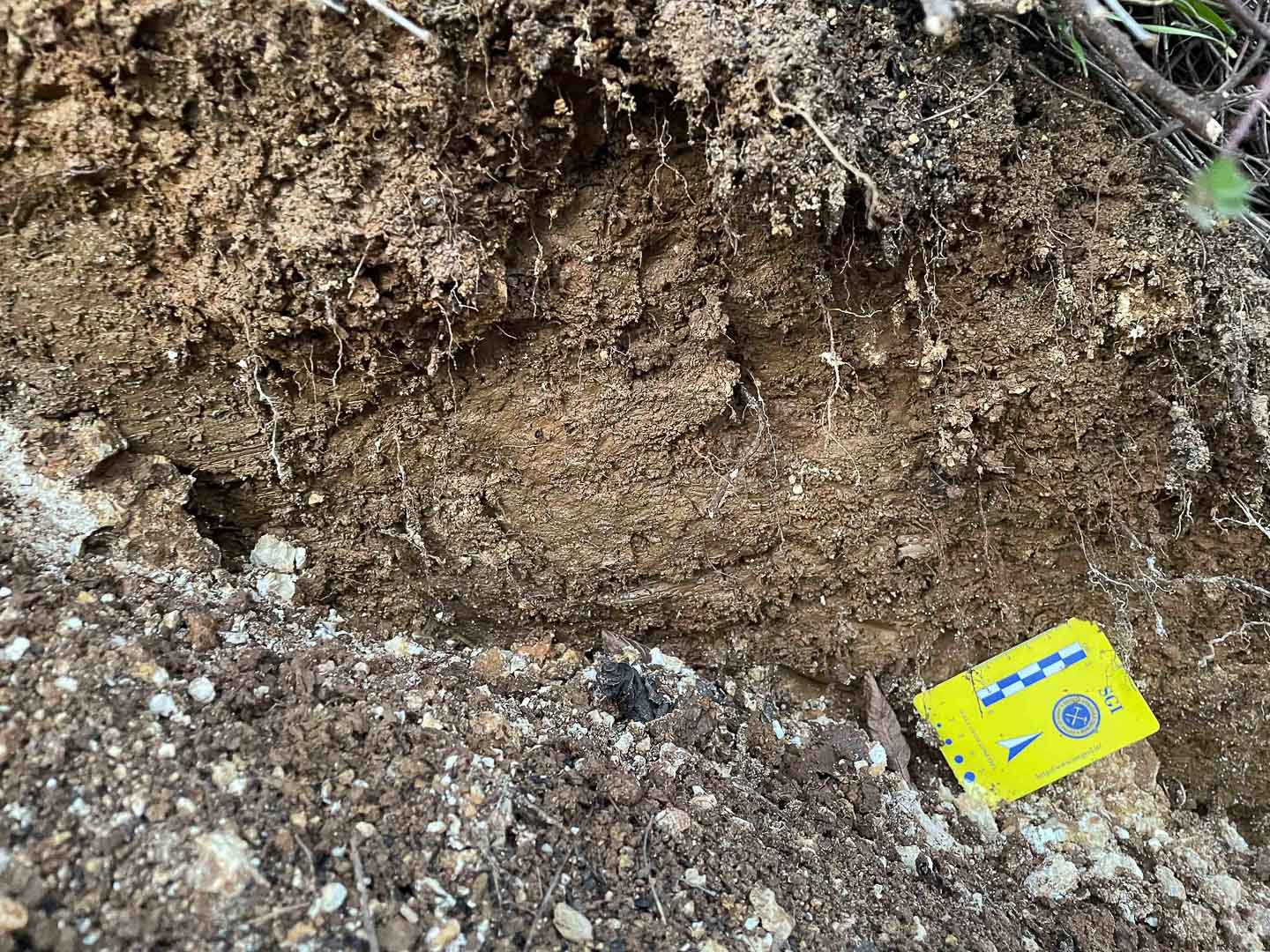

Supplement: Supplementary file 3 — Supplementary Information 3. [file 41598_2021_88378_MOESM3_ESM.zip › 1d (13-01-2021).jpg]

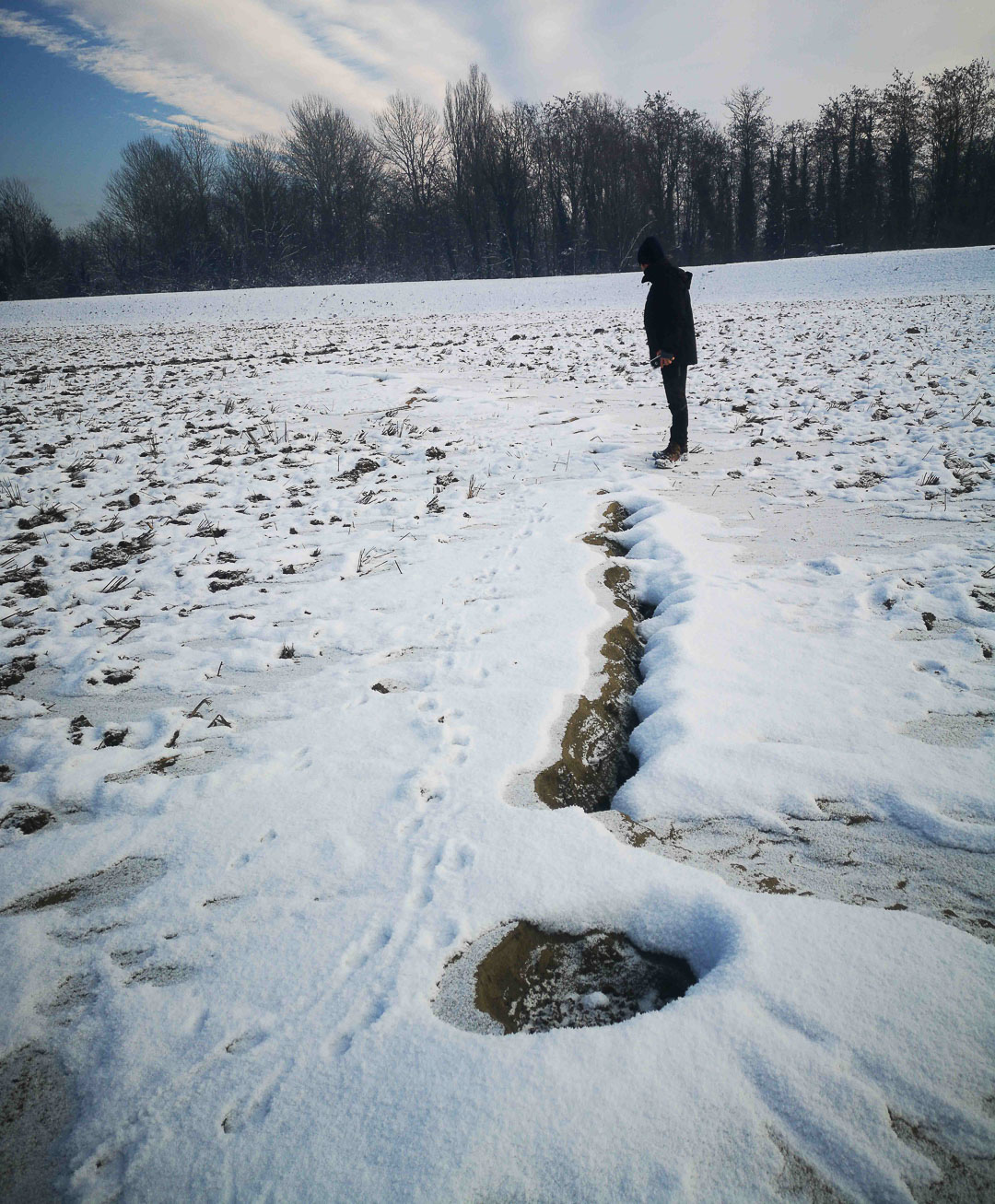

Supplement: Supplementary file 3 — Supplementary Information 3. [file 41598_2021_88378_MOESM3_ESM.zip › 200 (14-01-2021).jpg]

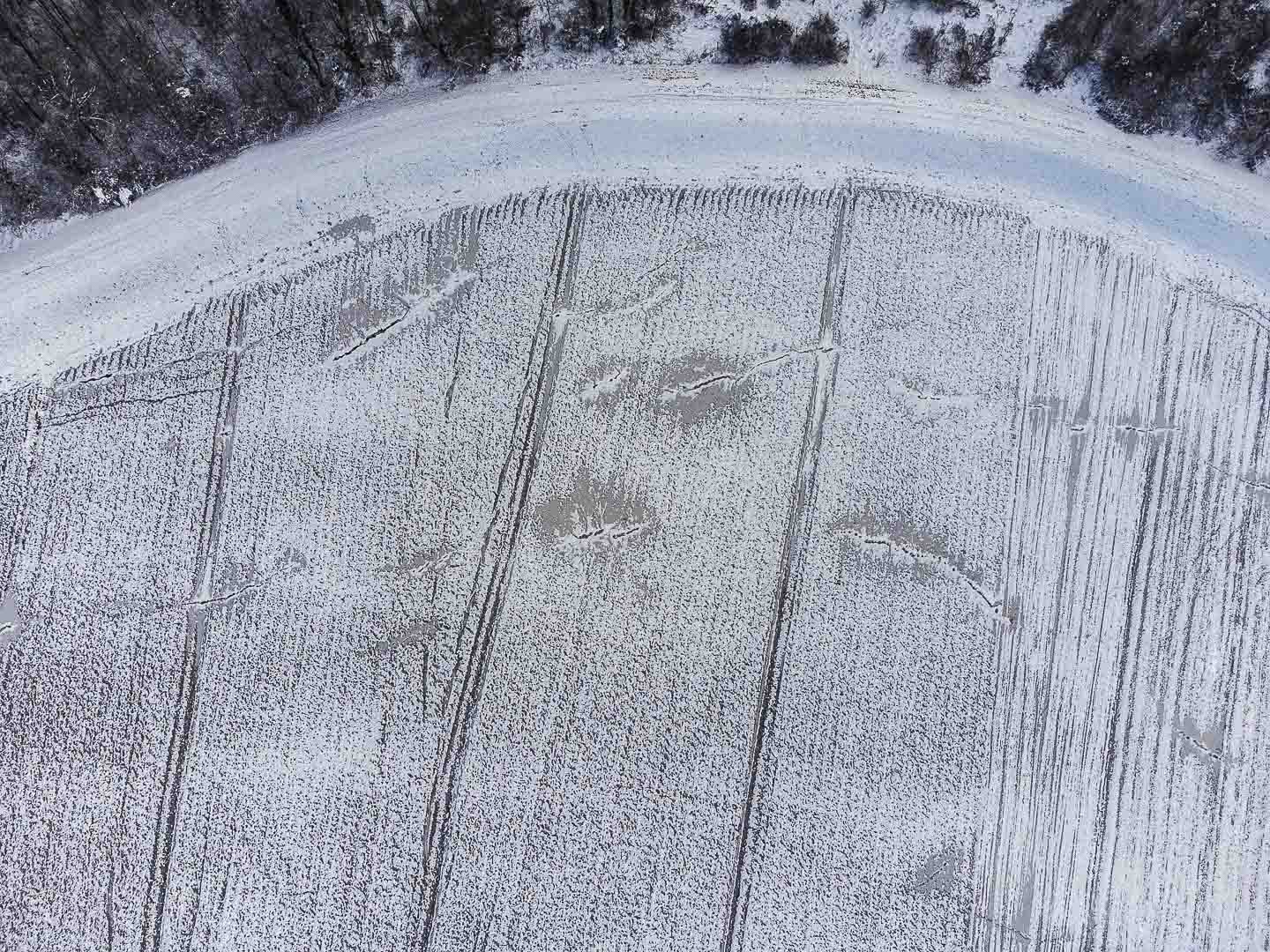

Supplement: Supplementary file 3 — Supplementary Information 3. [file 41598_2021_88378_MOESM3_ESM.zip › 203 (14-01-2021).jpg]

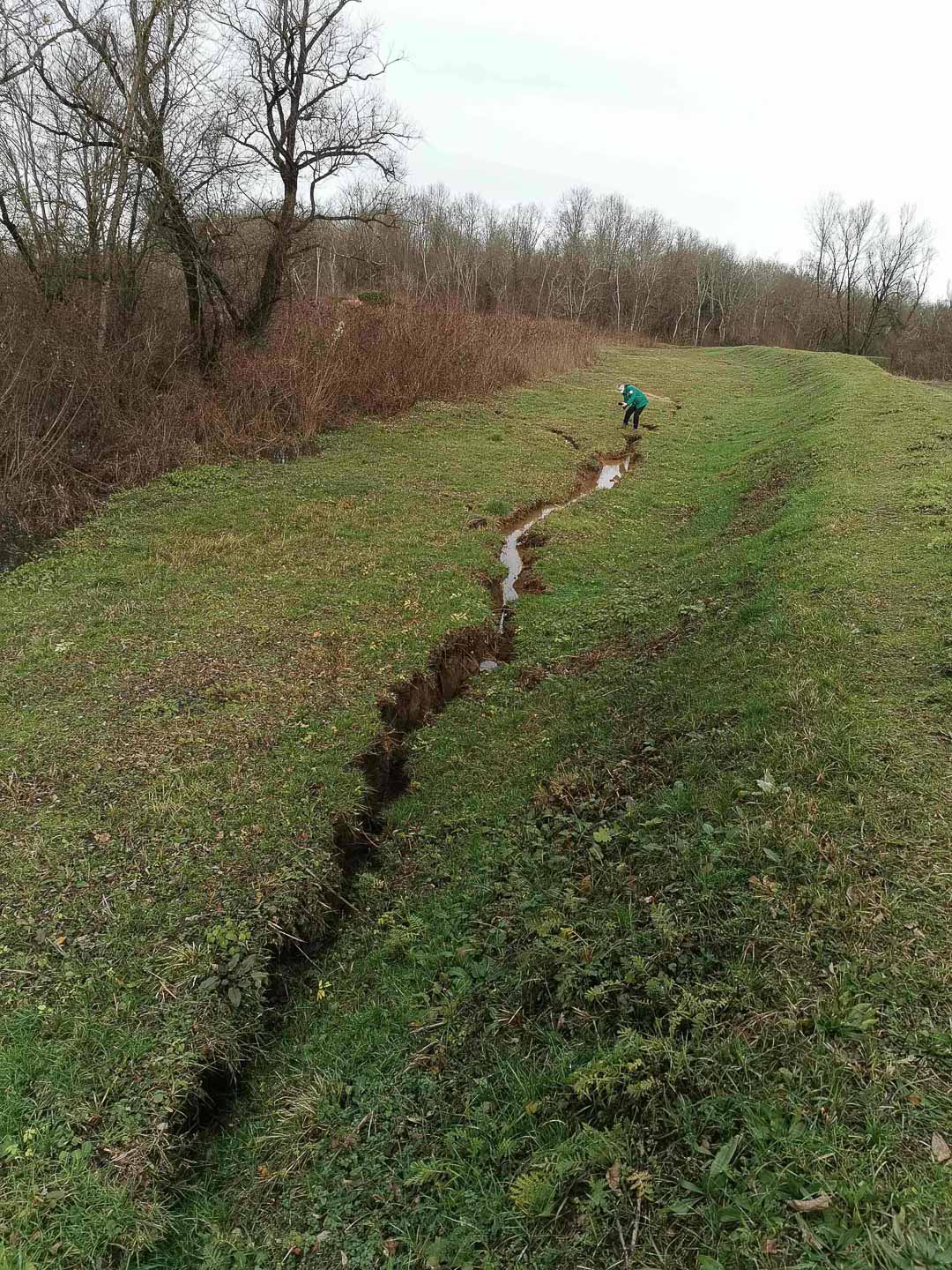

Supplement: Supplementary file 3 — Supplementary Information 3. [file 41598_2021_88378_MOESM3_ESM.zip › 204a (05-01-2021).jpg]

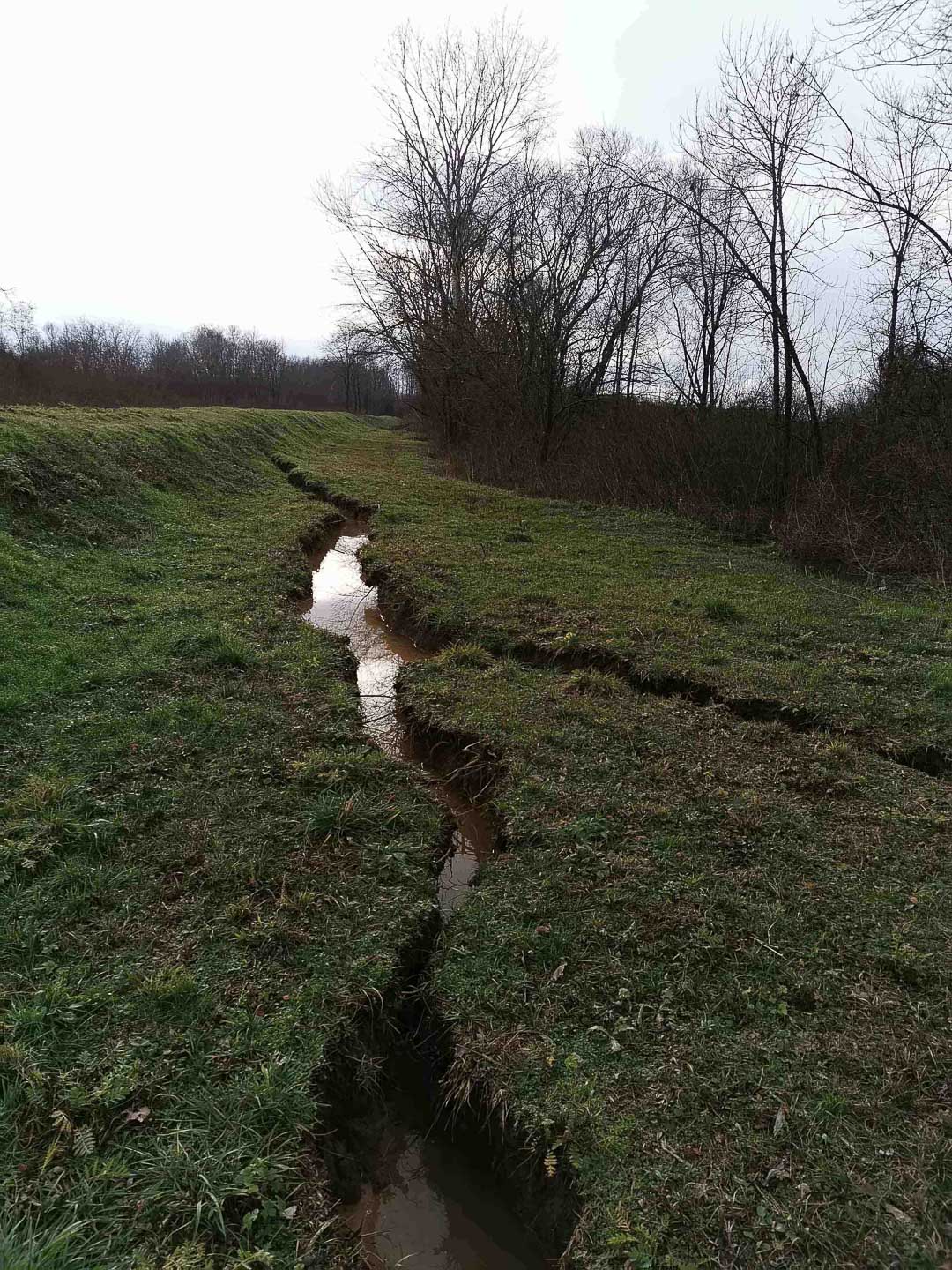

Supplement: Supplementary file 3 — Supplementary Information 3. [file 41598_2021_88378_MOESM3_ESM.zip › 204b (05-01-2021).jpg]

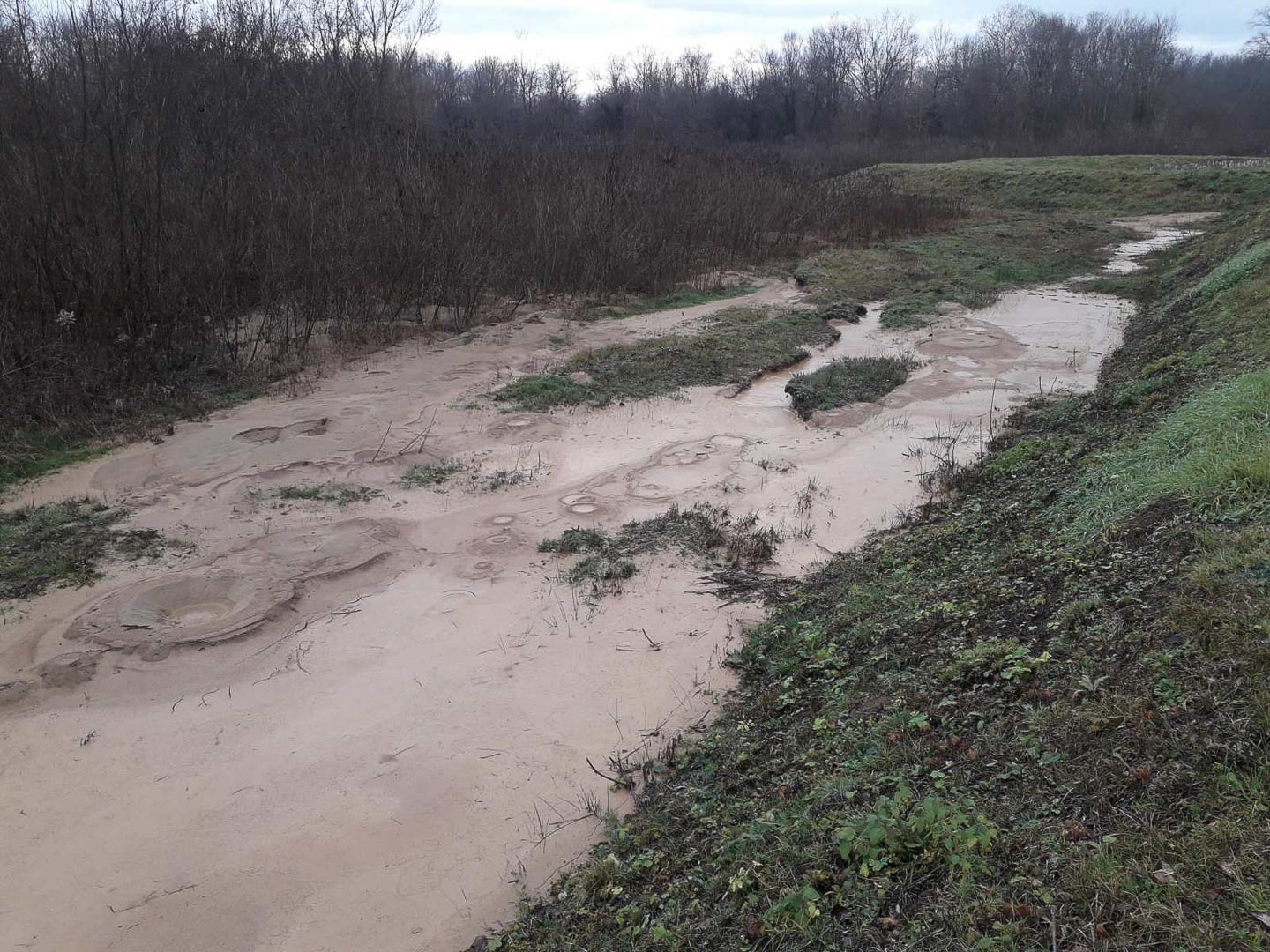

Supplement: Supplementary file 3 — Supplementary Information 3. [file 41598_2021_88378_MOESM3_ESM.zip › 205 (05-01-2021).jpg]

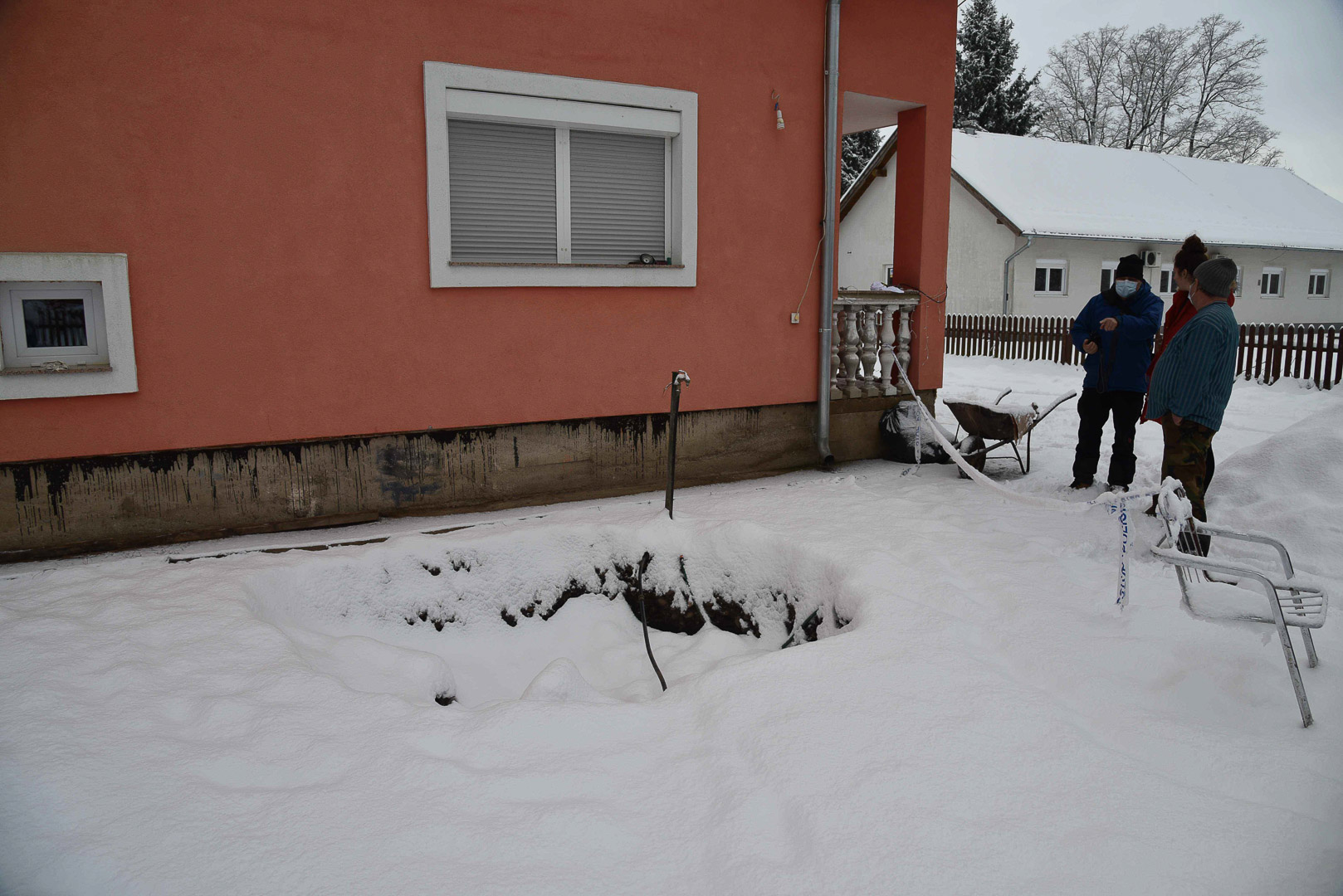

Supplement: Supplementary file 3 — Supplementary Information 3. [file 41598_2021_88378_MOESM3_ESM.zip › 207a (14-01-2021).jpg]

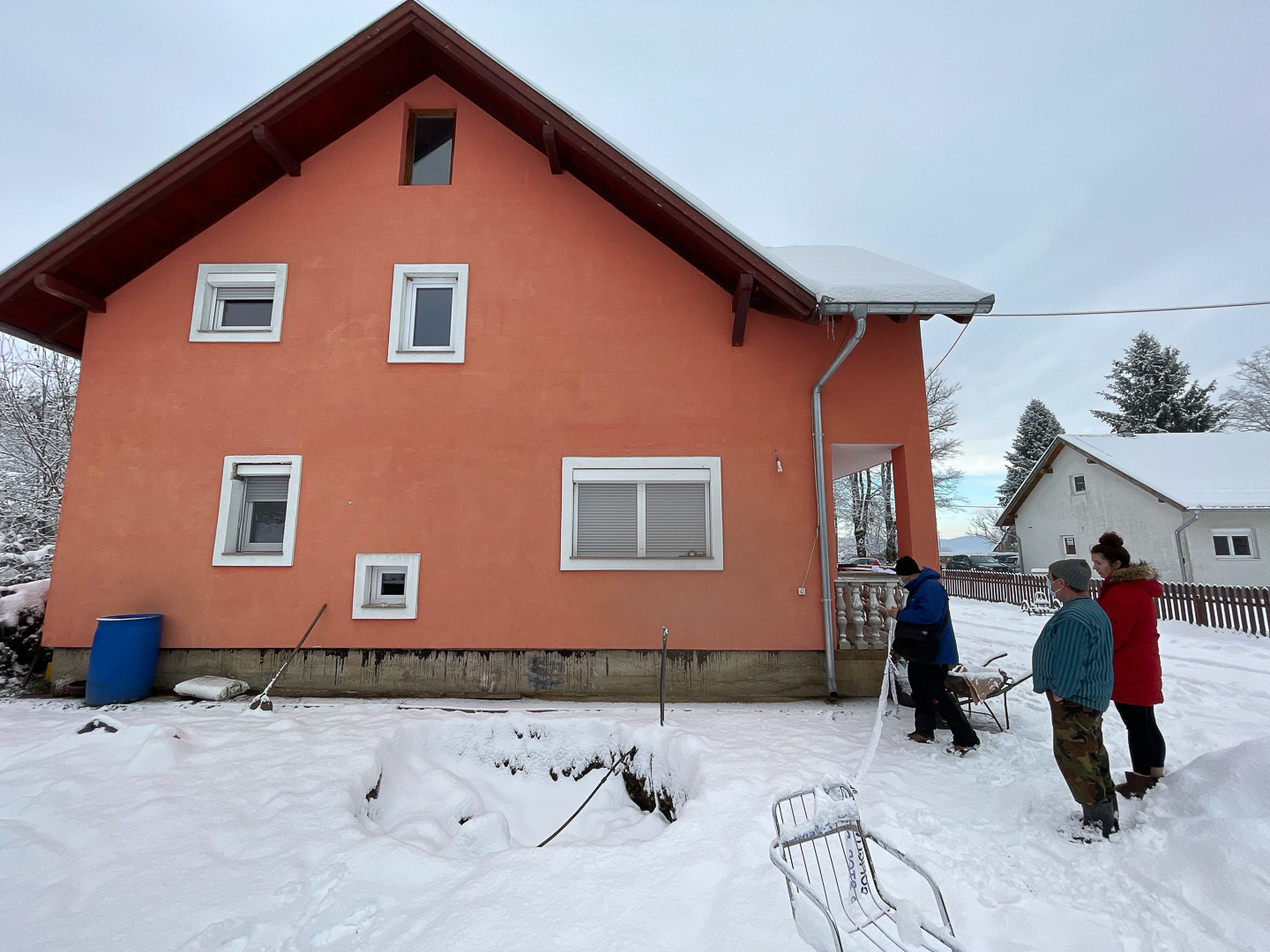

Supplement: Supplementary file 3 — Supplementary Information 3. [file 41598_2021_88378_MOESM3_ESM.zip › 207b (14-01-2021).jpg]

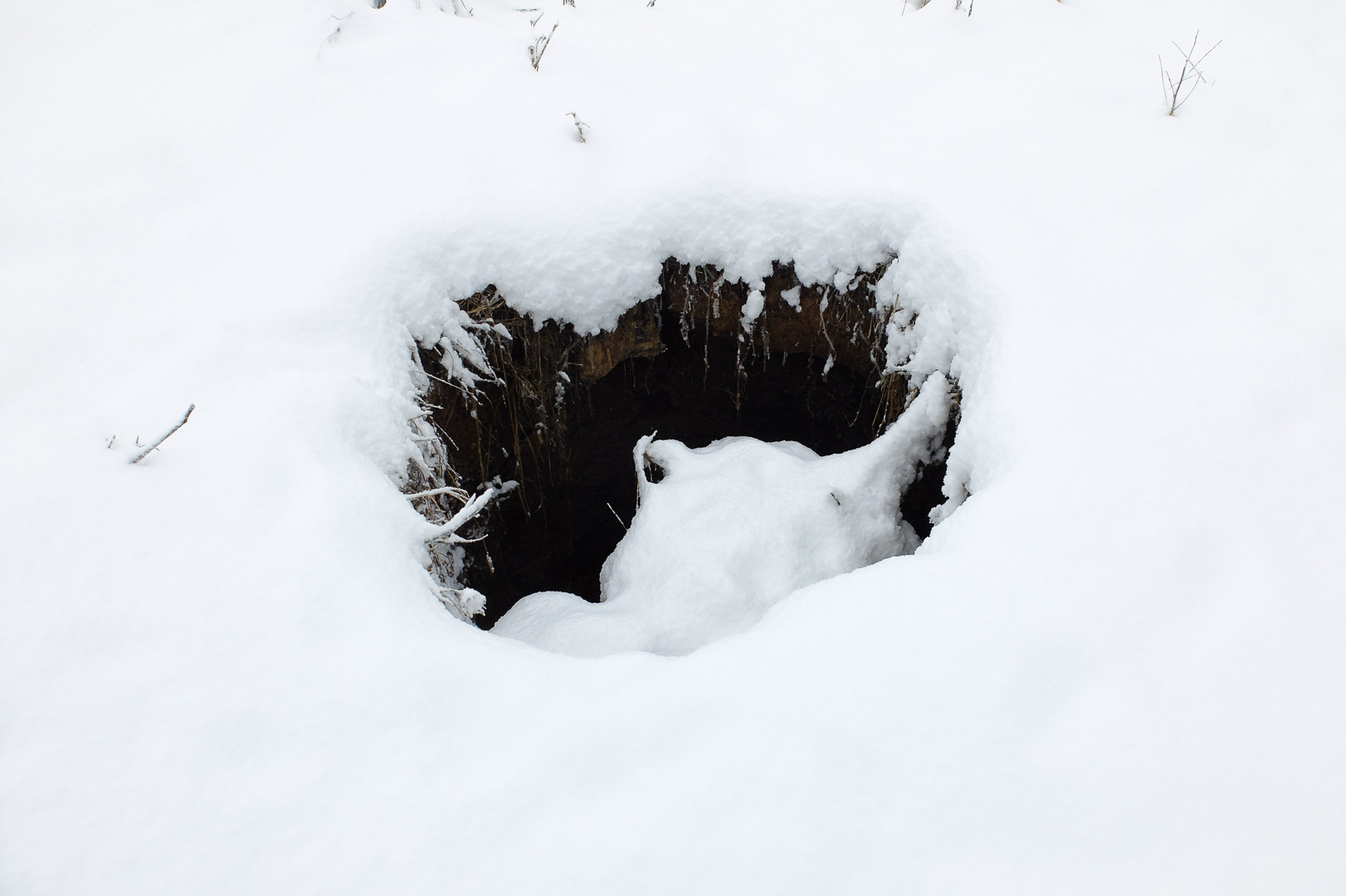

Supplement: Supplementary file 3 — Supplementary Information 3. [file 41598_2021_88378_MOESM3_ESM.zip › 208a (14-01-2021).jpg]

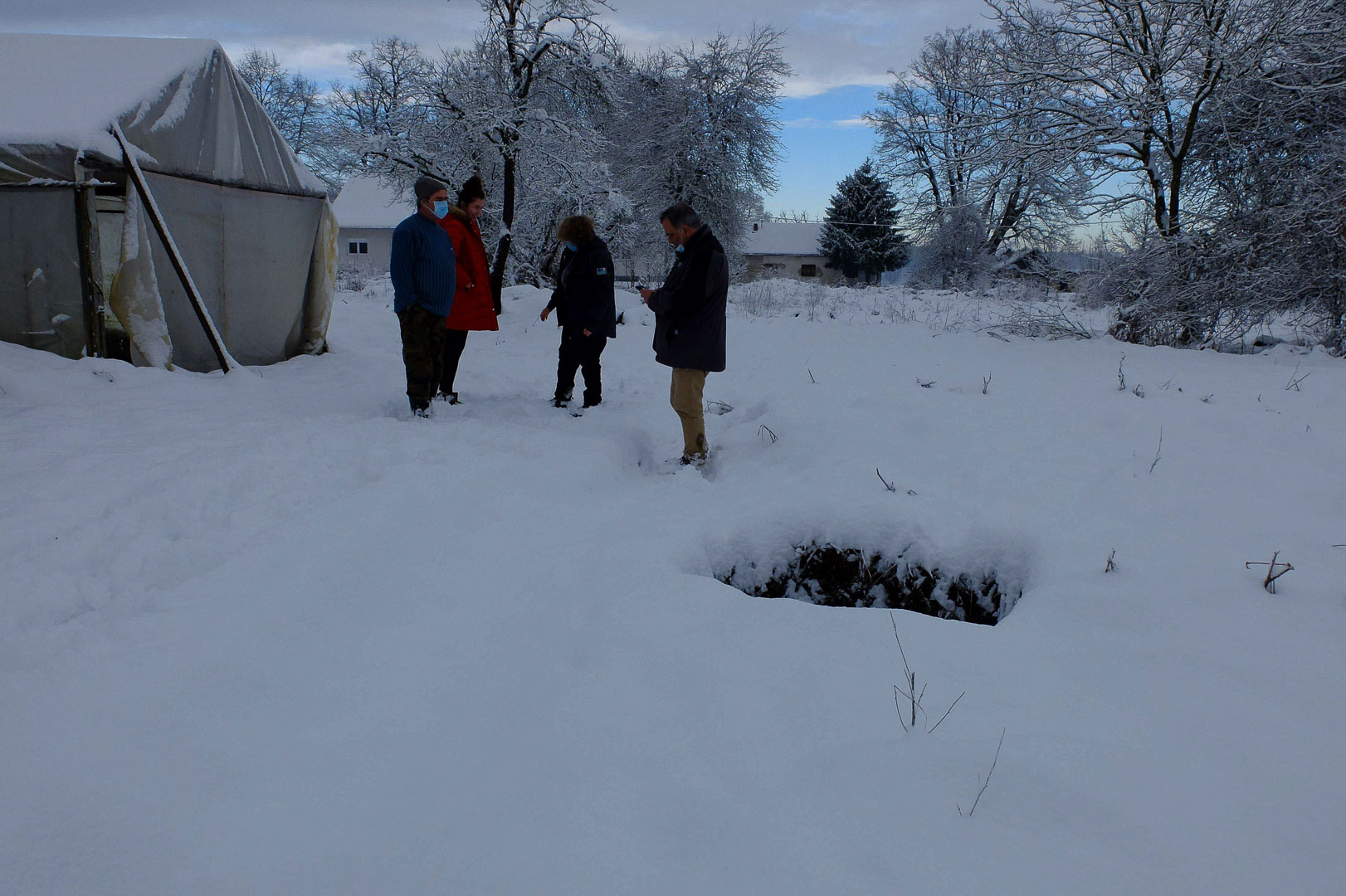

Supplement: Supplementary file 3 — Supplementary Information 3. [file 41598_2021_88378_MOESM3_ESM.zip › 208b (14-01-2021).jpg]

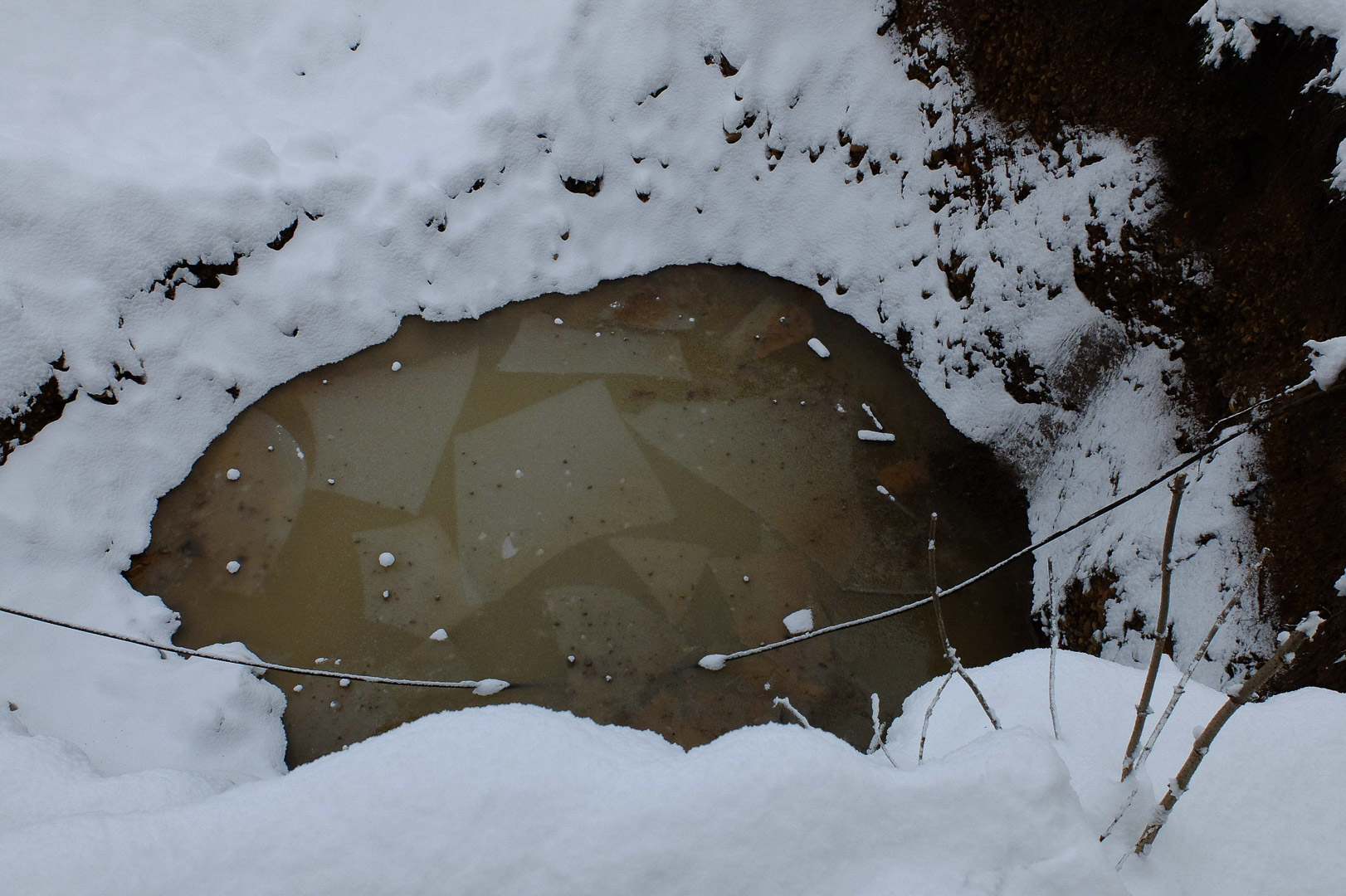

Supplement: Supplementary file 3 — Supplementary Information 3. [file 41598_2021_88378_MOESM3_ESM.zip › 209a (14-01-2021).jpg]

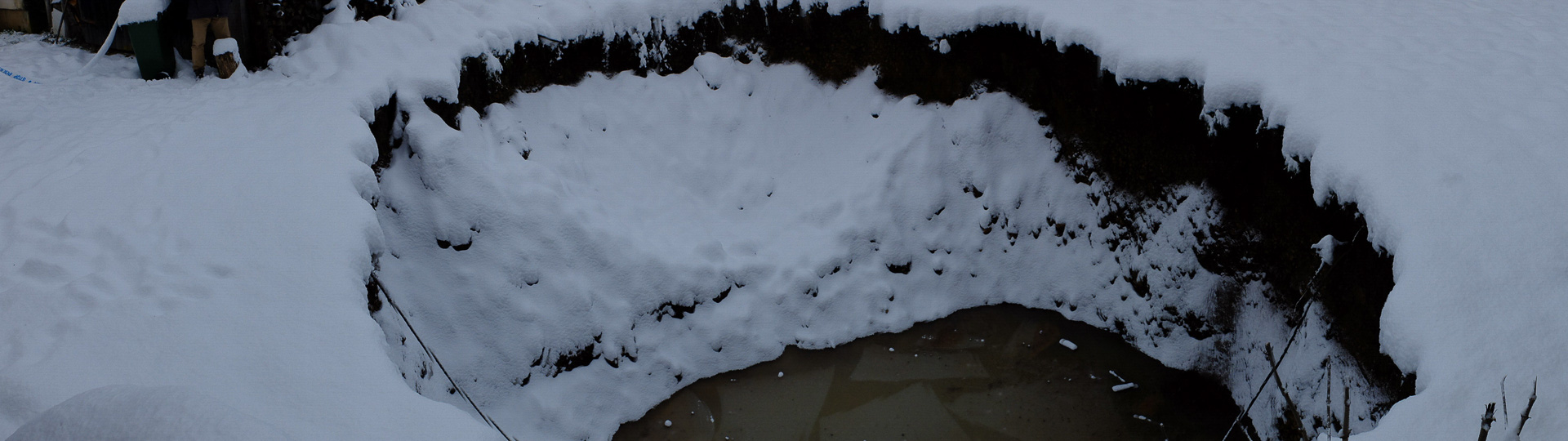

Supplement: Supplementary file 3 — Supplementary Information 3. [file 41598_2021_88378_MOESM3_ESM.zip › 209b (14-01-2021).jpg]

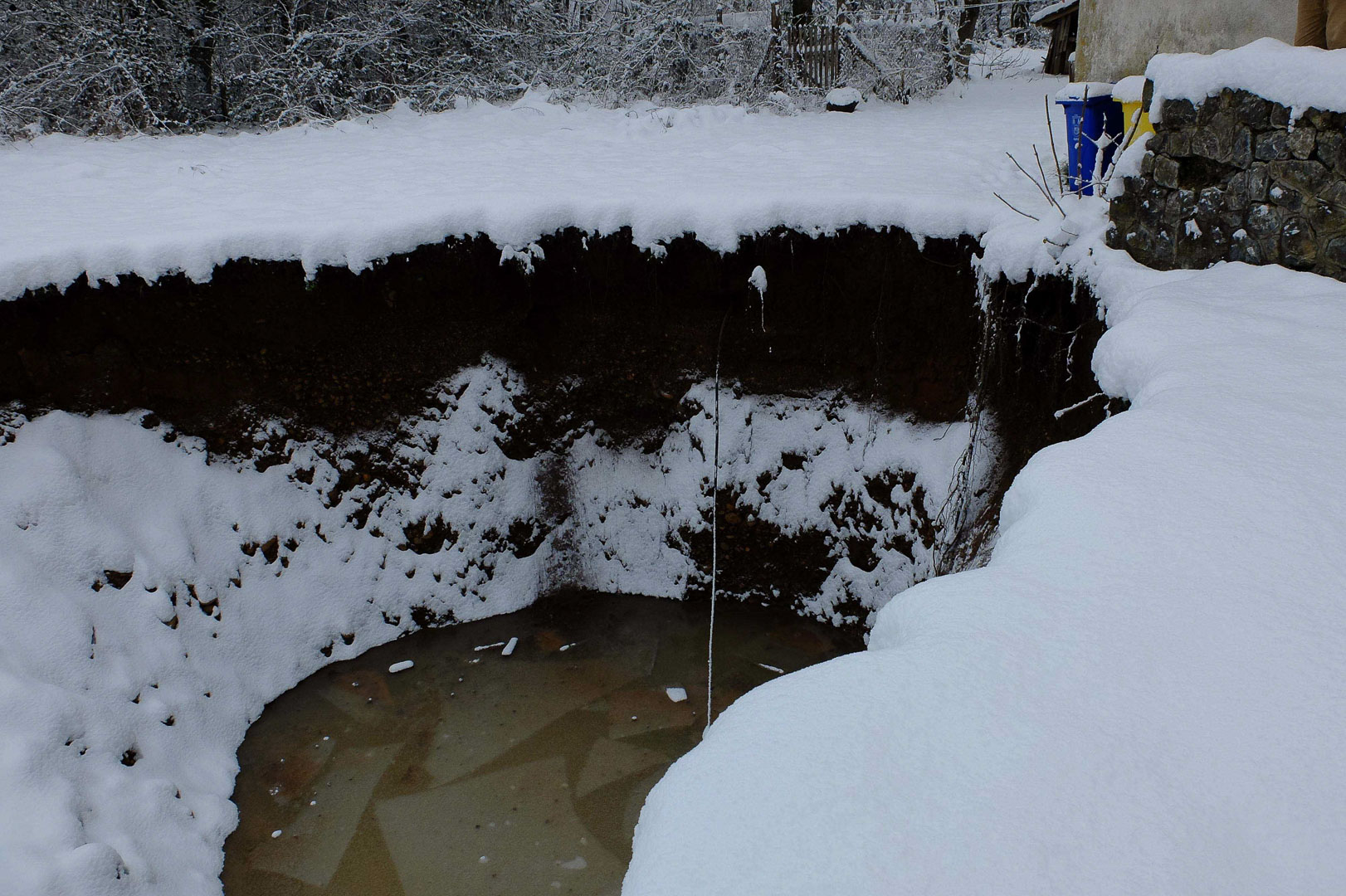

Supplement: Supplementary file 3 — Supplementary Information 3. [file 41598_2021_88378_MOESM3_ESM.zip › 209c (14-01-2021).jpg]

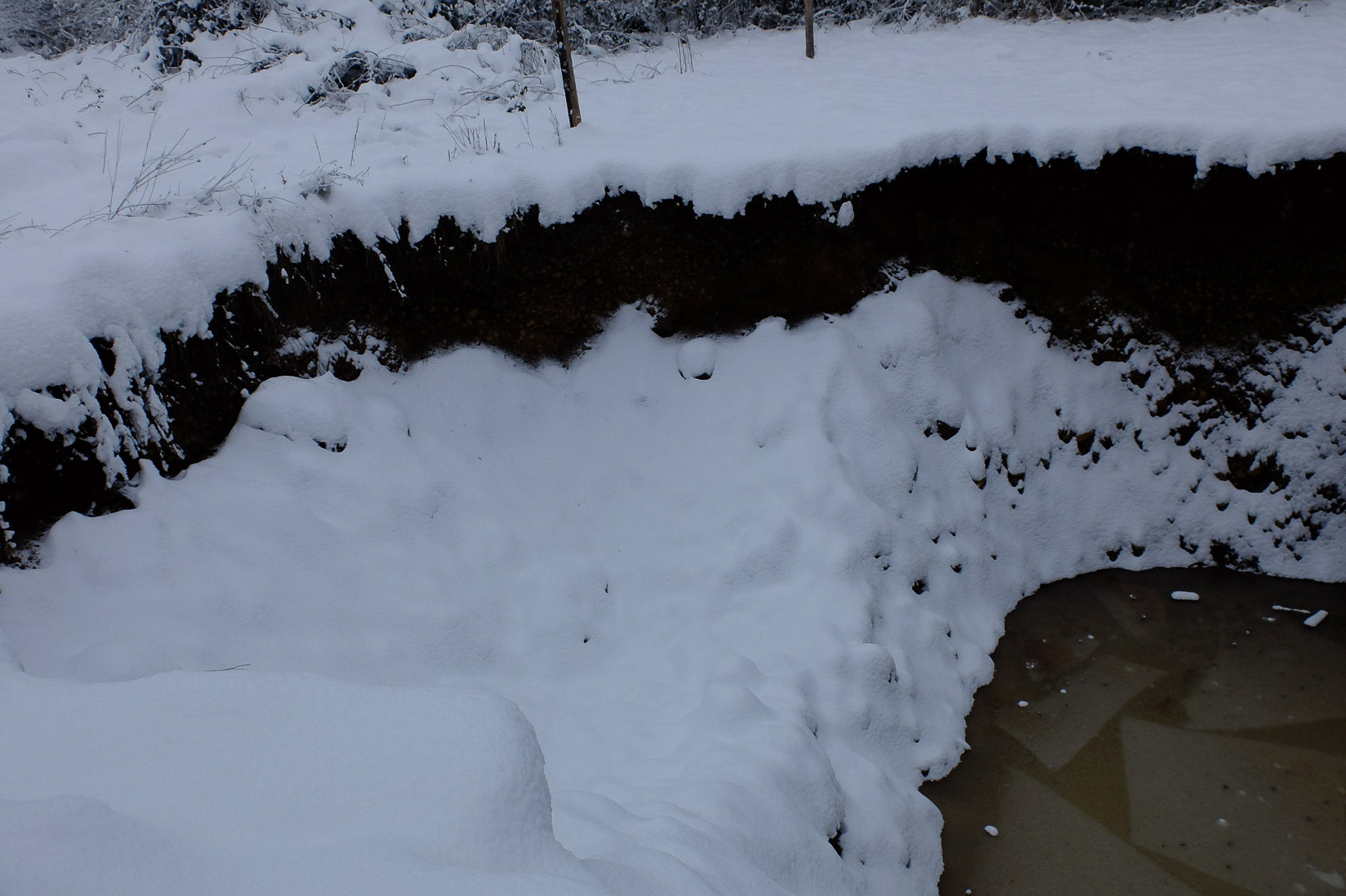

Supplement: Supplementary file 3 — Supplementary Information 3. [file 41598_2021_88378_MOESM3_ESM.zip › 209d (14-01-2021).jpg]

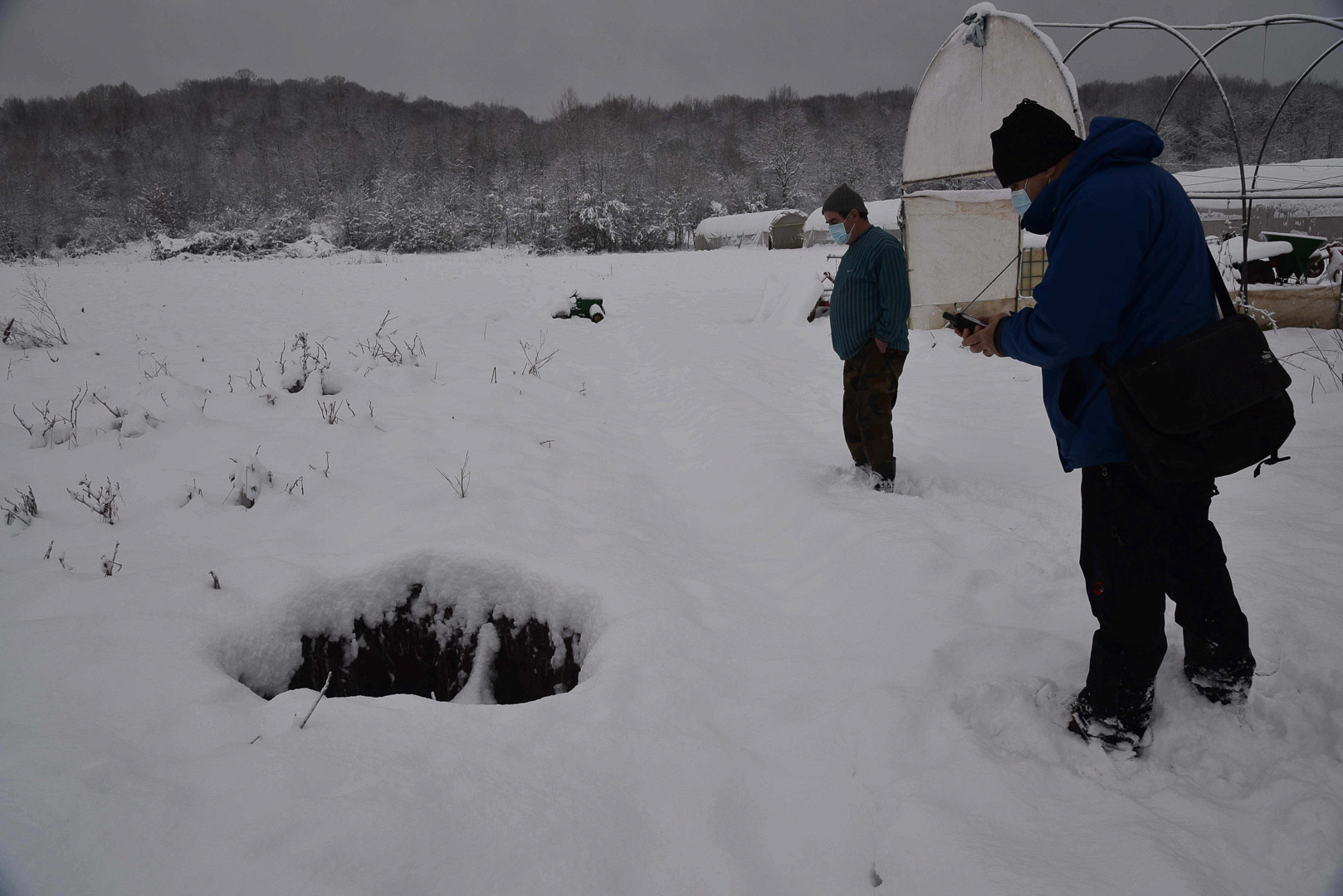

Supplement: Supplementary file 3 — Supplementary Information 3. [file 41598_2021_88378_MOESM3_ESM.zip › 210a (14-01-2021).jpg]

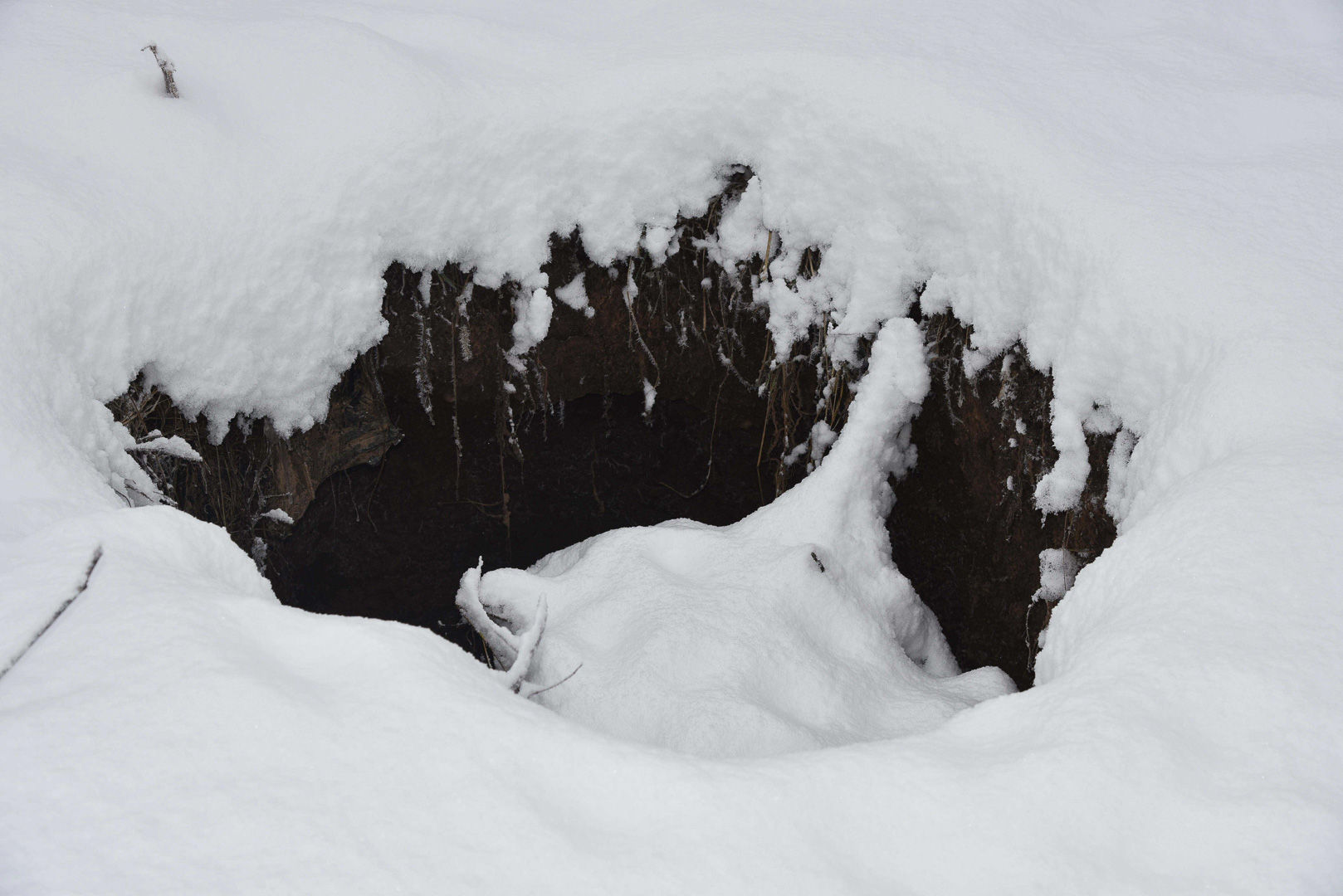

Supplement: Supplementary file 3 — Supplementary Information 3. [file 41598_2021_88378_MOESM3_ESM.zip › 210b (14-01-2021).jpg]

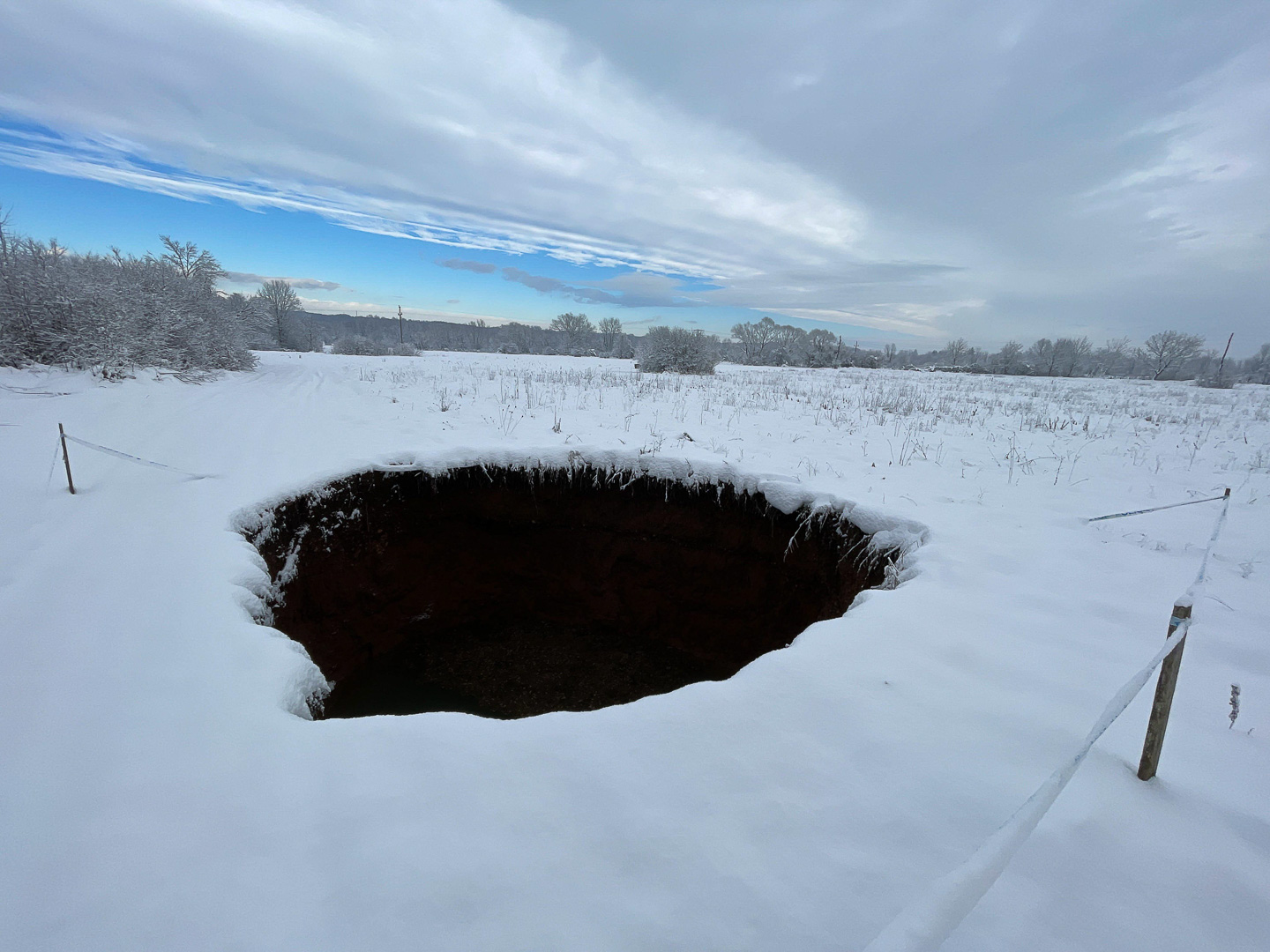

Supplement: Supplementary file 3 — Supplementary Information 3. [file 41598_2021_88378_MOESM3_ESM.zip › 211a (14-01-2021).jpg]

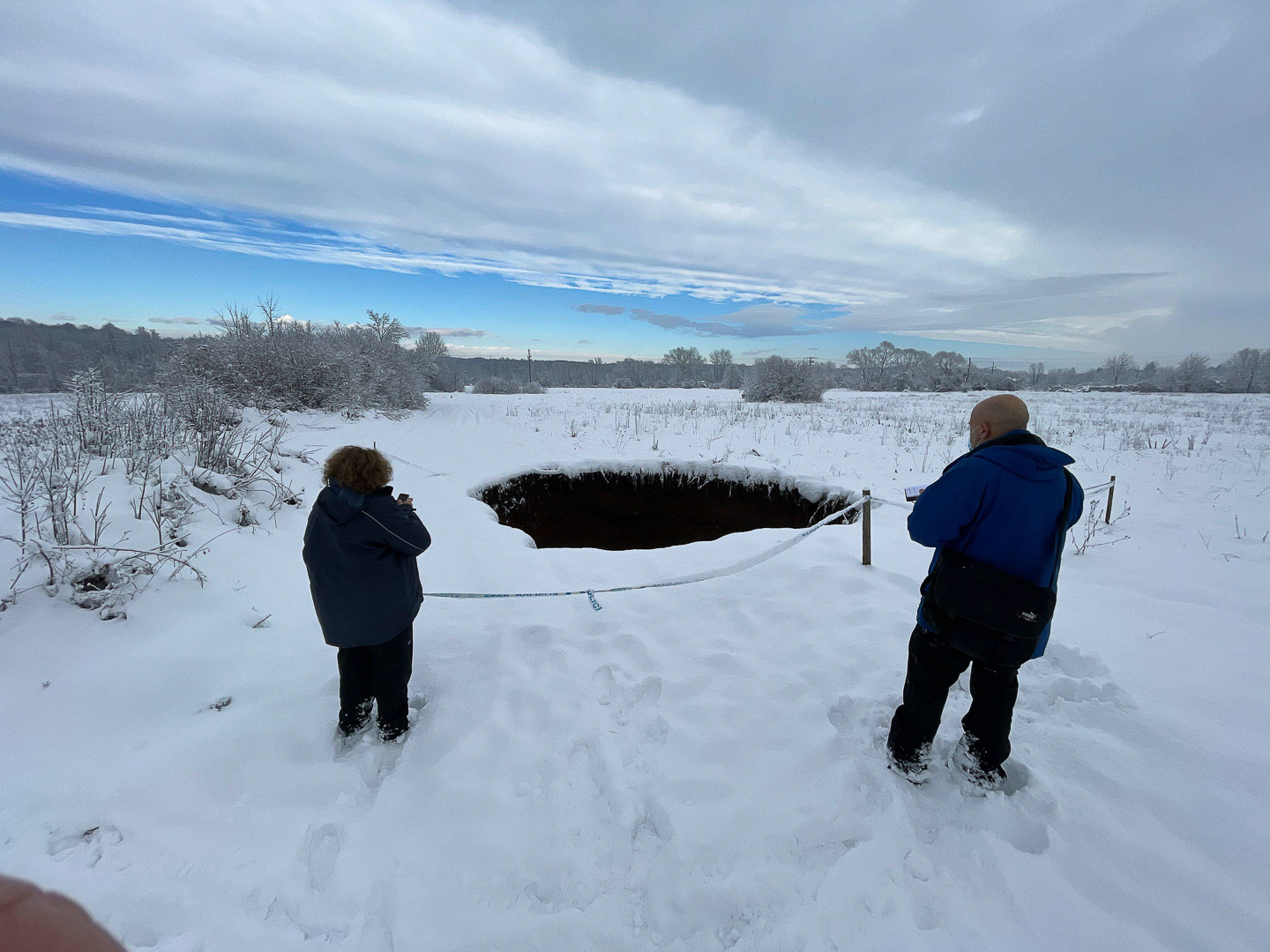

Supplement: Supplementary file 3 — Supplementary Information 3. [file 41598_2021_88378_MOESM3_ESM.zip › 211b (14-01-2021).jpg]

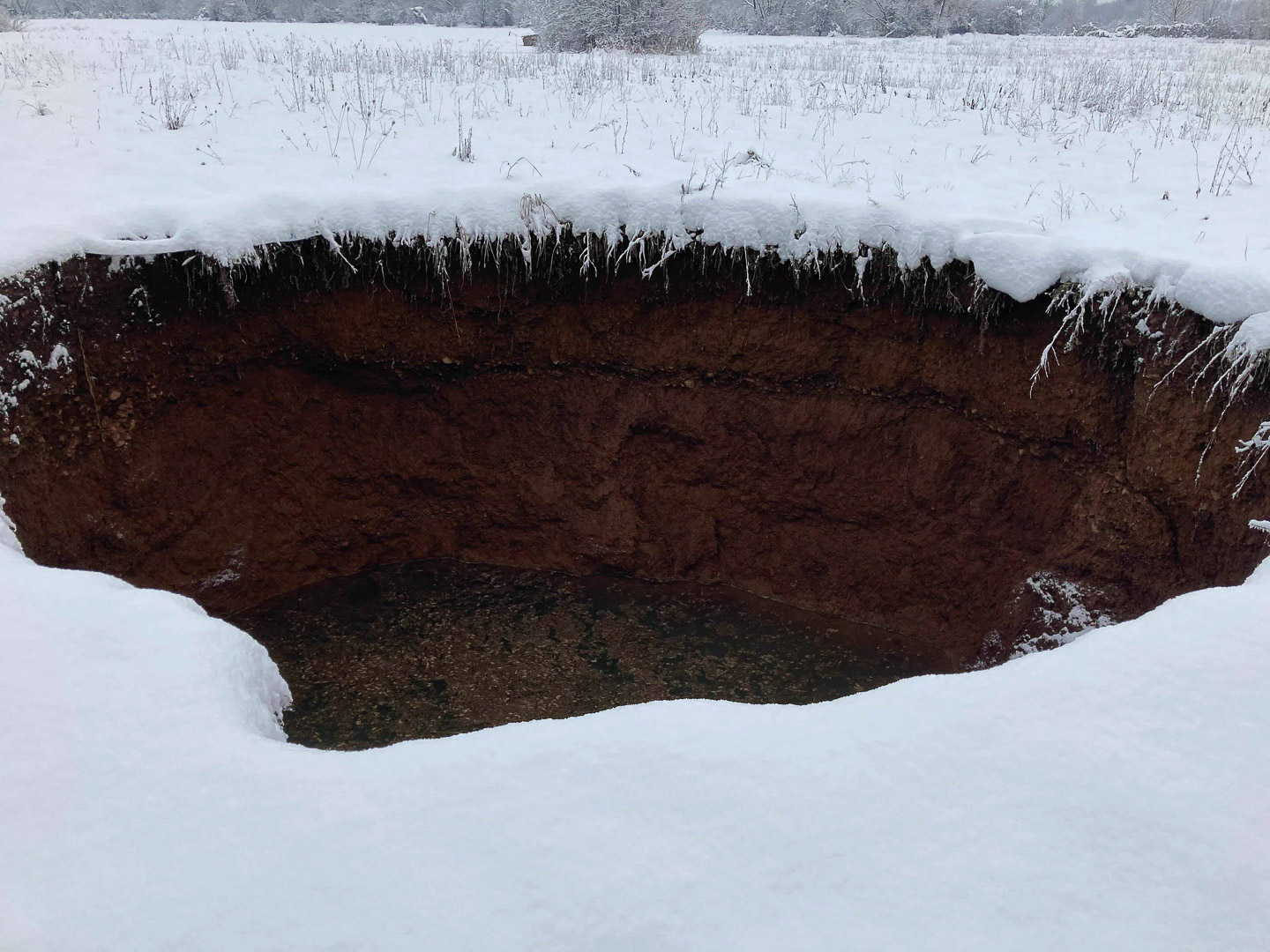

Supplement: Supplementary file 3 — Supplementary Information 3. [file 41598_2021_88378_MOESM3_ESM.zip › 211c (14-01-2021).jpg]

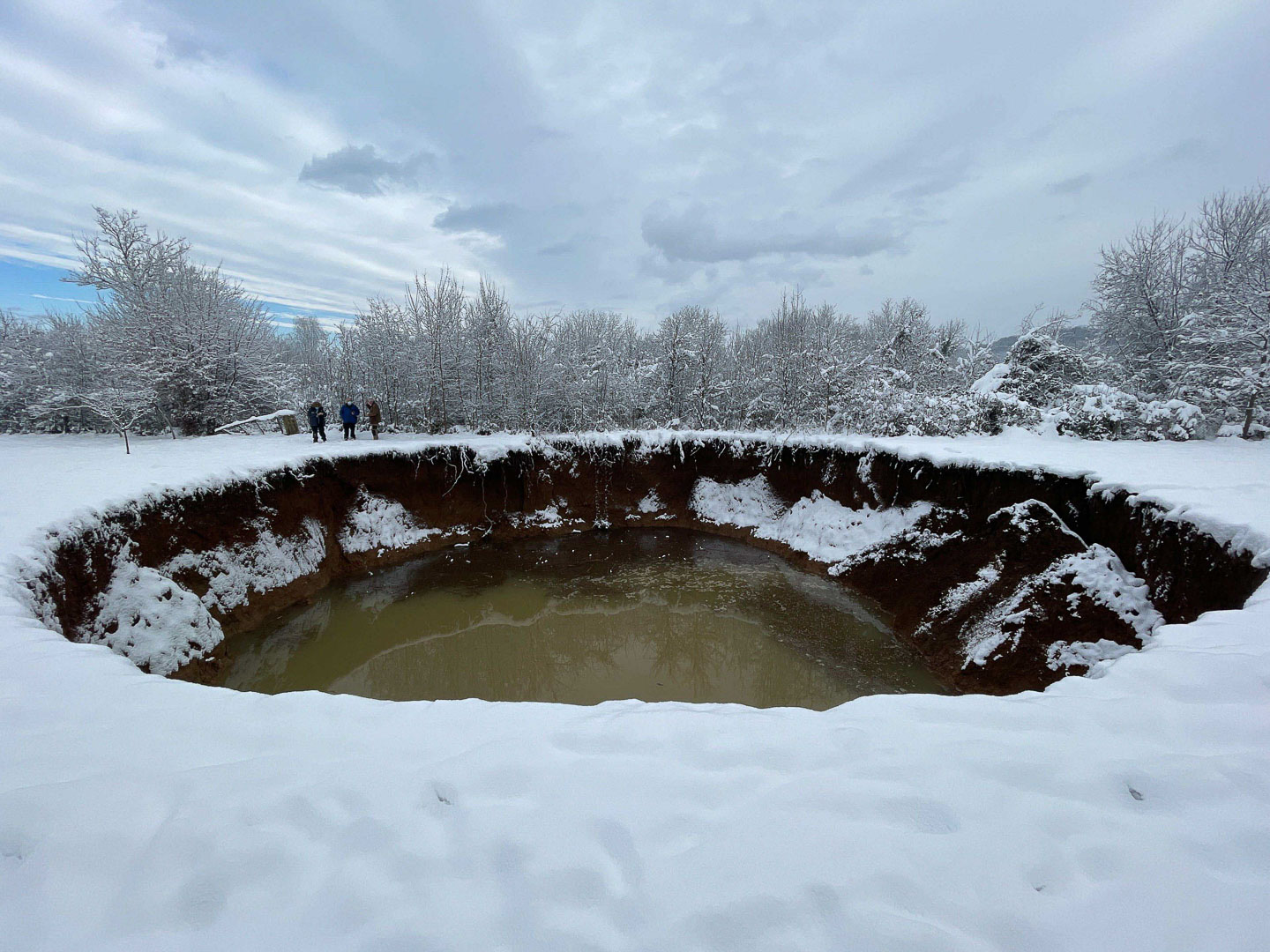

Supplement: Supplementary file 3 — Supplementary Information 3. [file 41598_2021_88378_MOESM3_ESM.zip › 212a (14-01-2021).jpg]

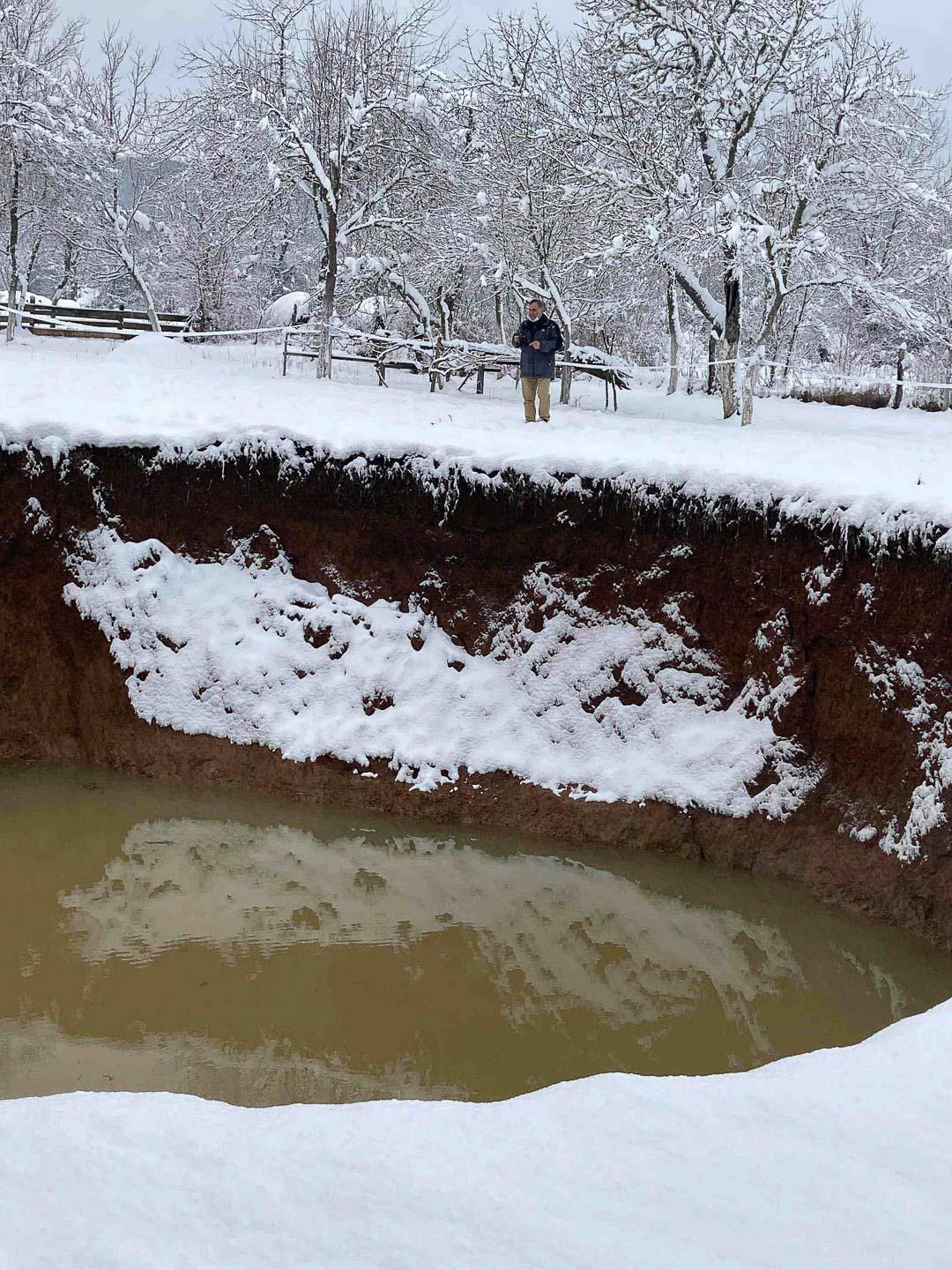

Supplement: Supplementary file 3 — Supplementary Information 3. [file 41598_2021_88378_MOESM3_ESM.zip › 212b (14-01-2021).jpg]

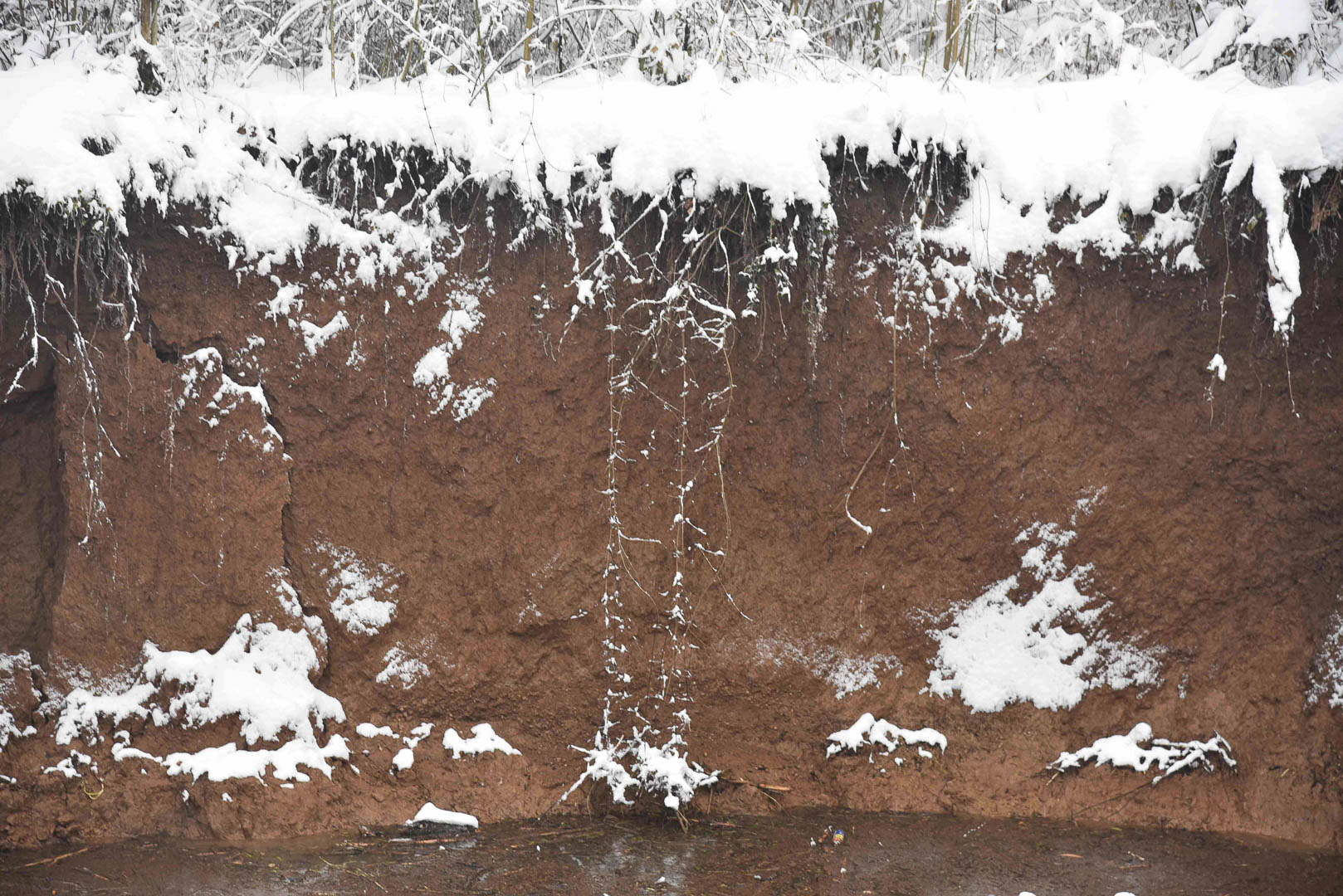

Supplement: Supplementary file 3 — Supplementary Information 3. [file 41598_2021_88378_MOESM3_ESM.zip › 212c (14-01-2021).jpg]

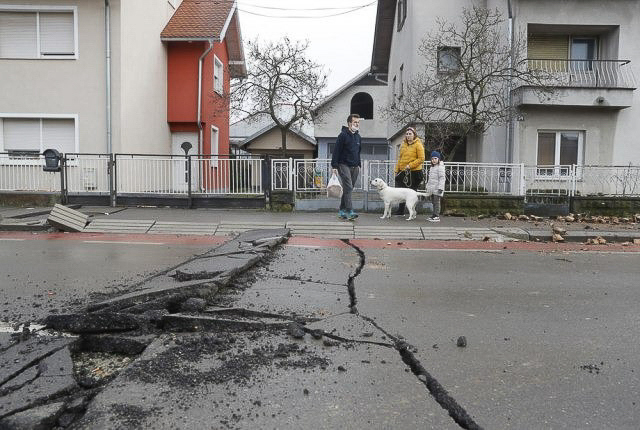

Supplement: Supplementary file 3 — Supplementary Information 3. [file 41598_2021_88378_MOESM3_ESM.zip › 222 (30-12-2020).jpg]

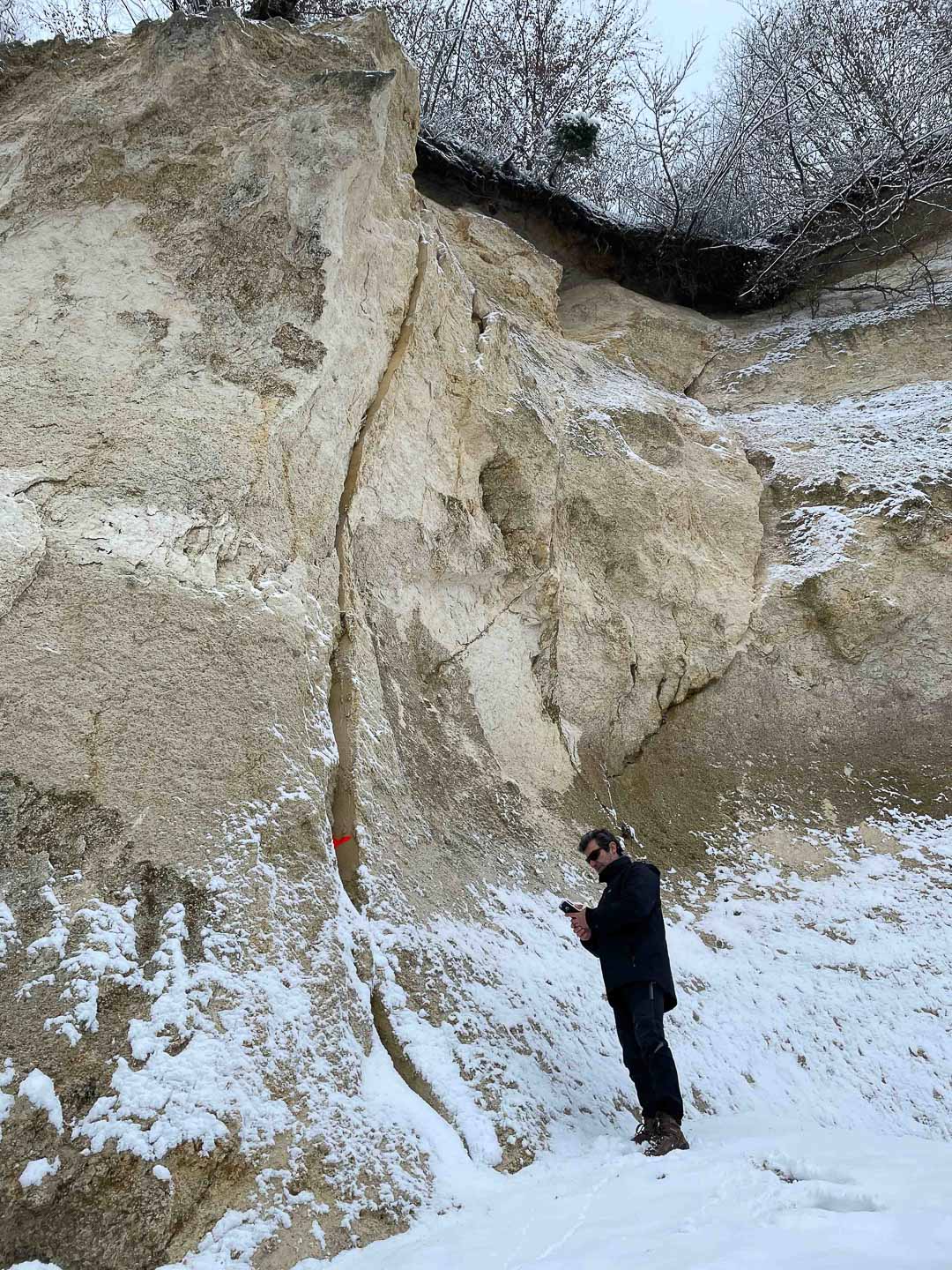

Supplement: Supplementary file 3 — Supplementary Information 3. [file 41598_2021_88378_MOESM3_ESM.zip › 22a (11-01-2021).jpg]

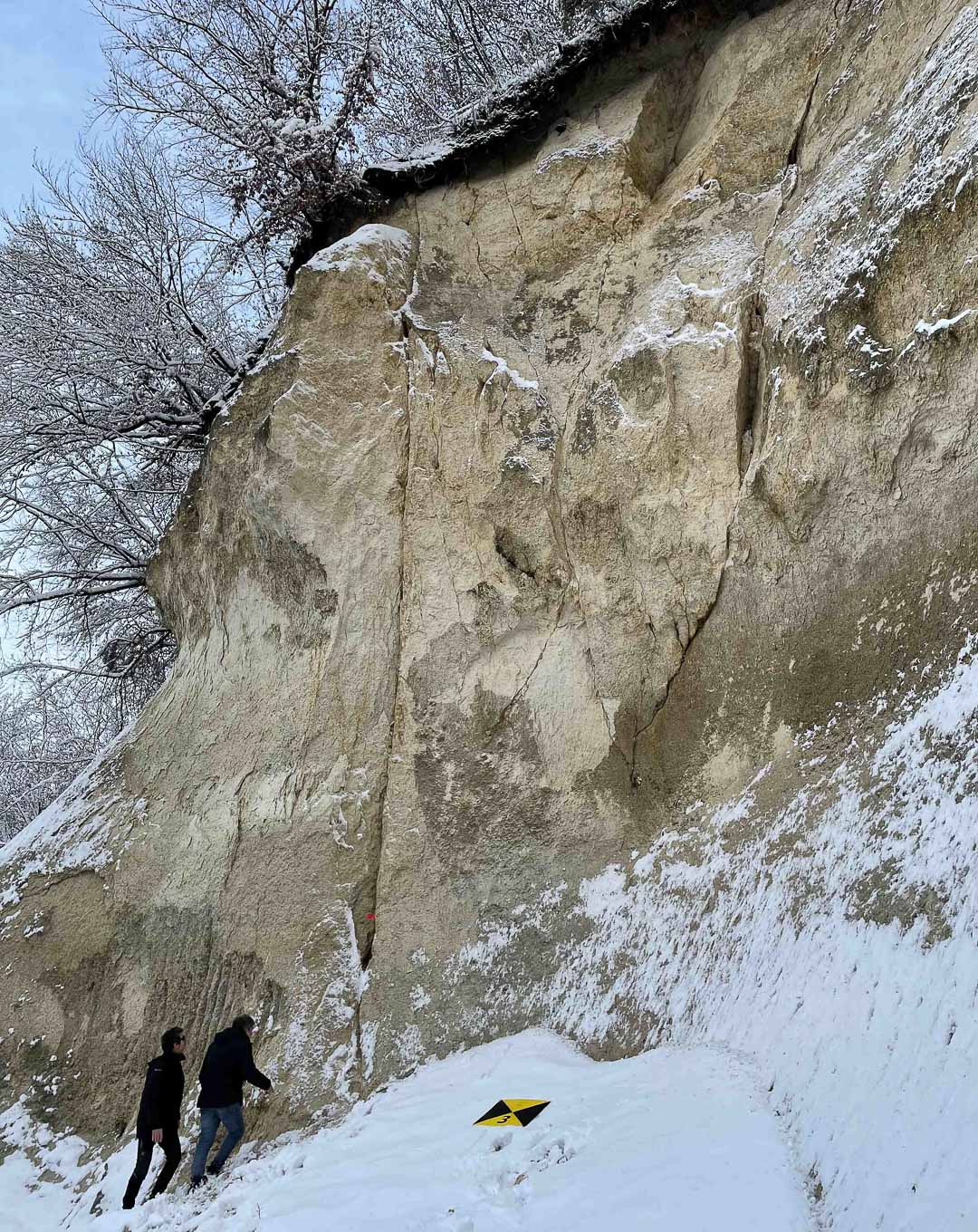

Supplement: Supplementary file 3 — Supplementary Information 3. [file 41598_2021_88378_MOESM3_ESM.zip › 22b (11-01-2021).jpg]

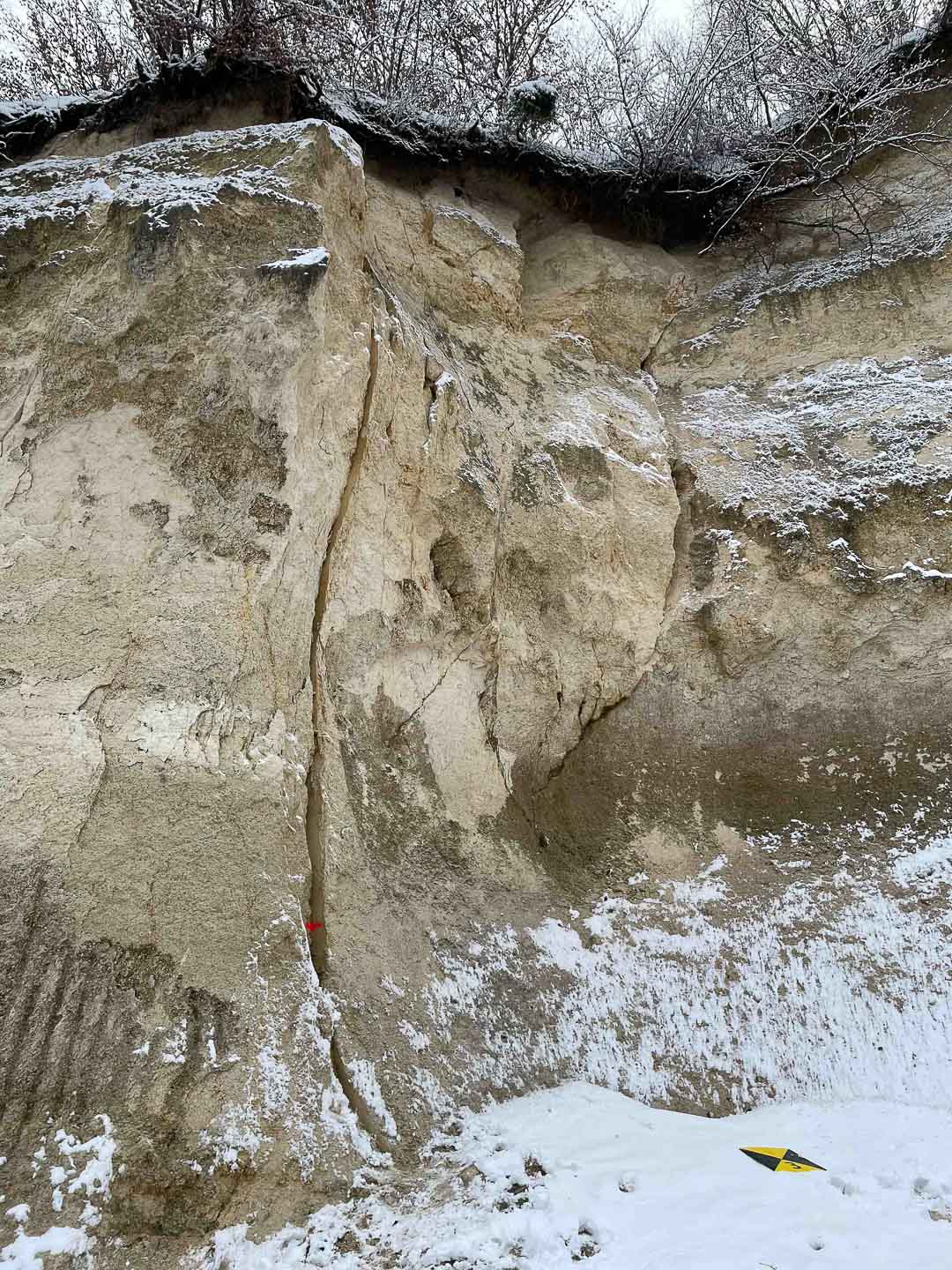

Supplement: Supplementary file 3 — Supplementary Information 3. [file 41598_2021_88378_MOESM3_ESM.zip › 22c (11-01-2021).jpg]

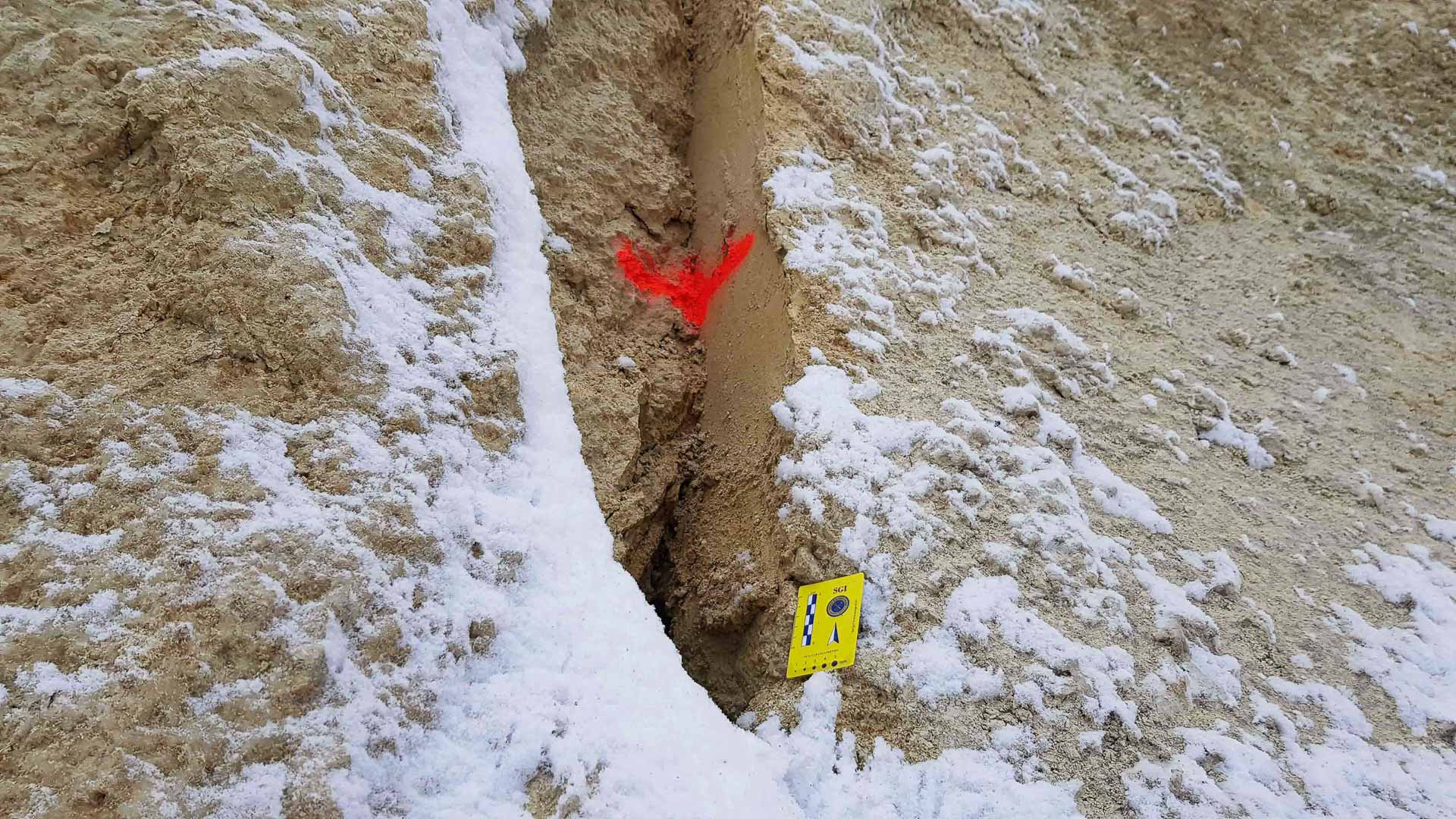

Supplement: Supplementary file 3 — Supplementary Information 3. [file 41598_2021_88378_MOESM3_ESM.zip › 22d (11-01-2021).jpg]

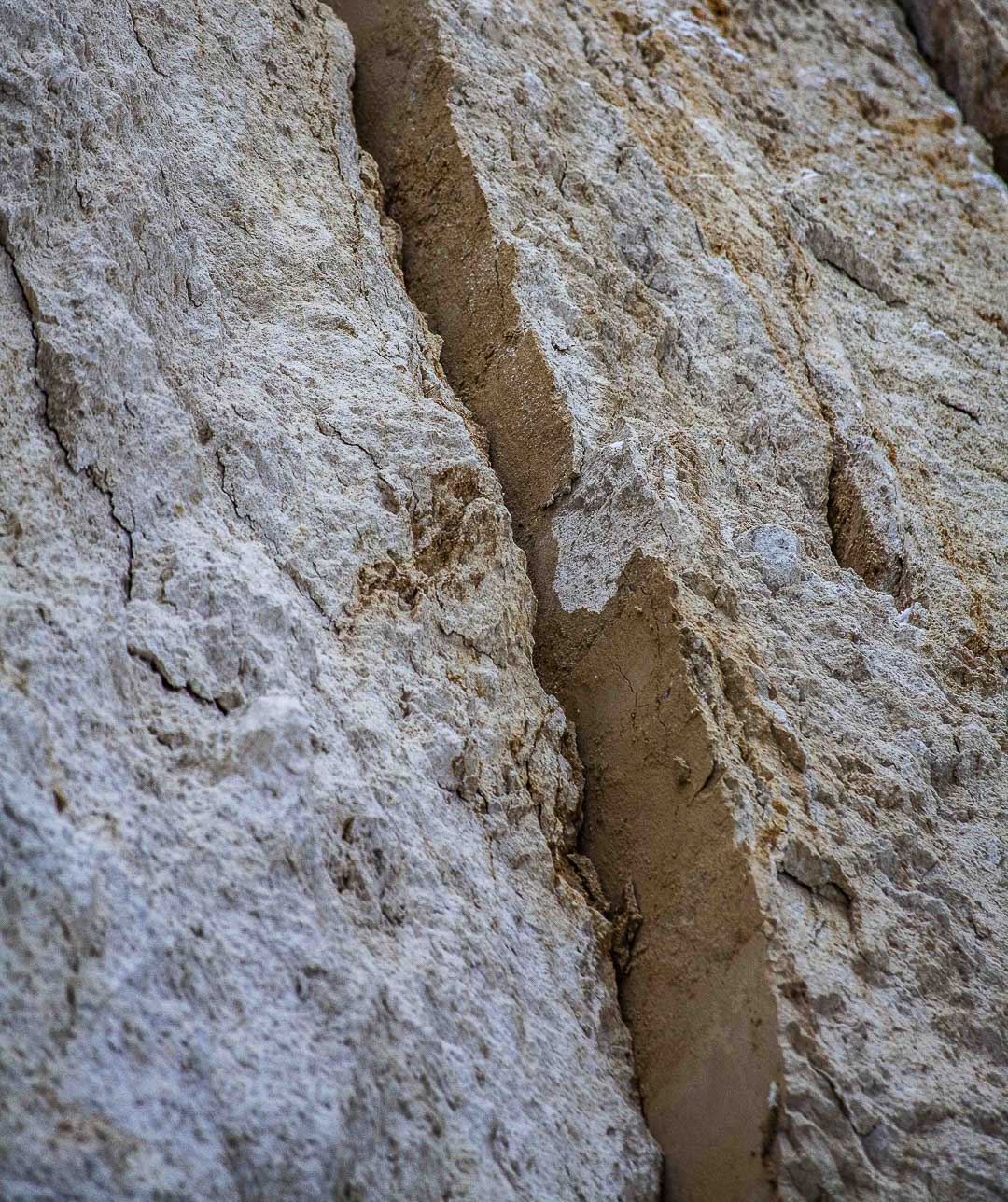

Supplement: Supplementary file 3 — Supplementary Information 3. [file 41598_2021_88378_MOESM3_ESM.zip › 22e (11-01-2021).jpg]

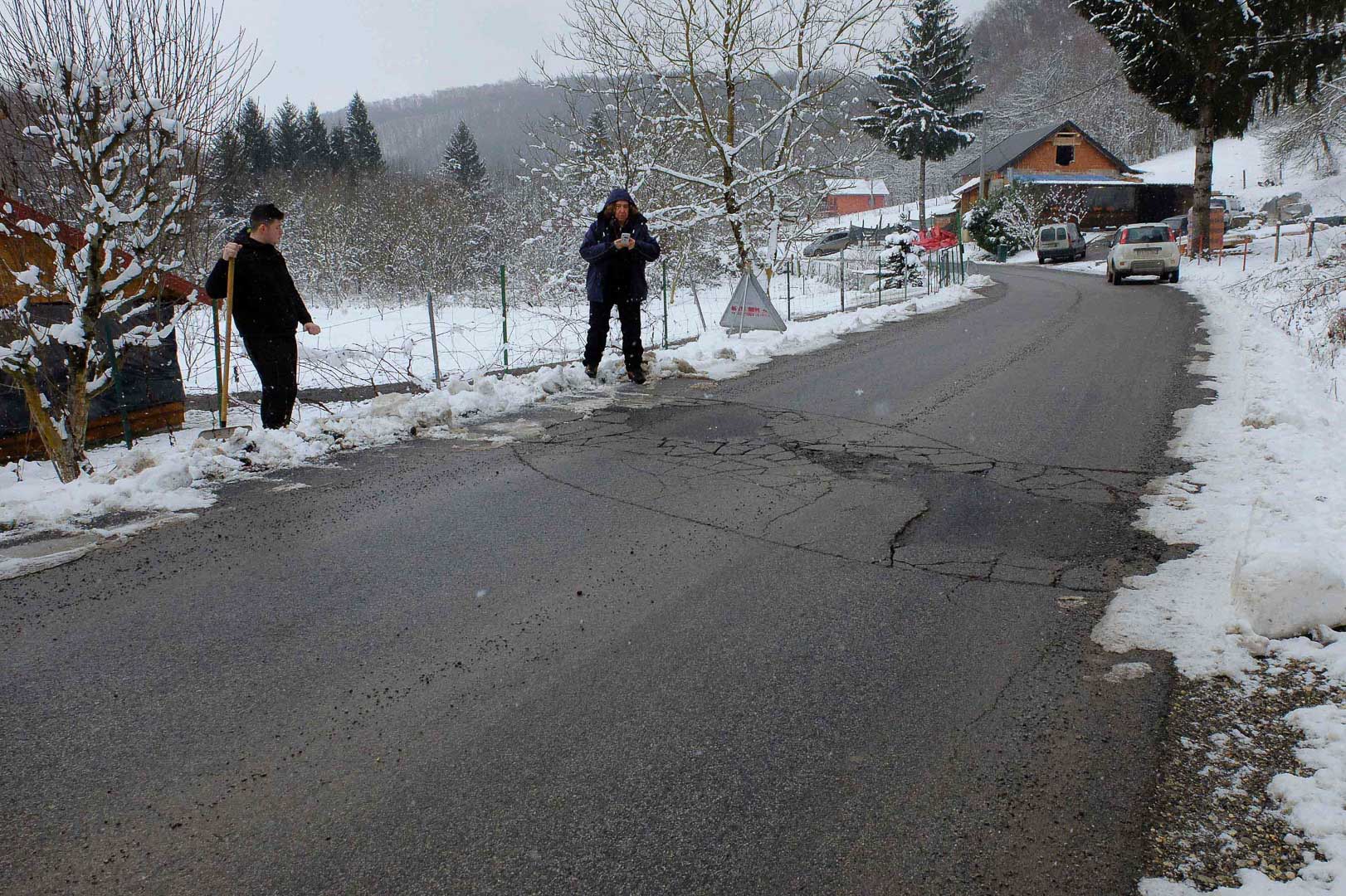

Supplement: Supplementary file 3 — Supplementary Information 3. [file 41598_2021_88378_MOESM3_ESM.zip › 23 (16-01-2021).jpg]

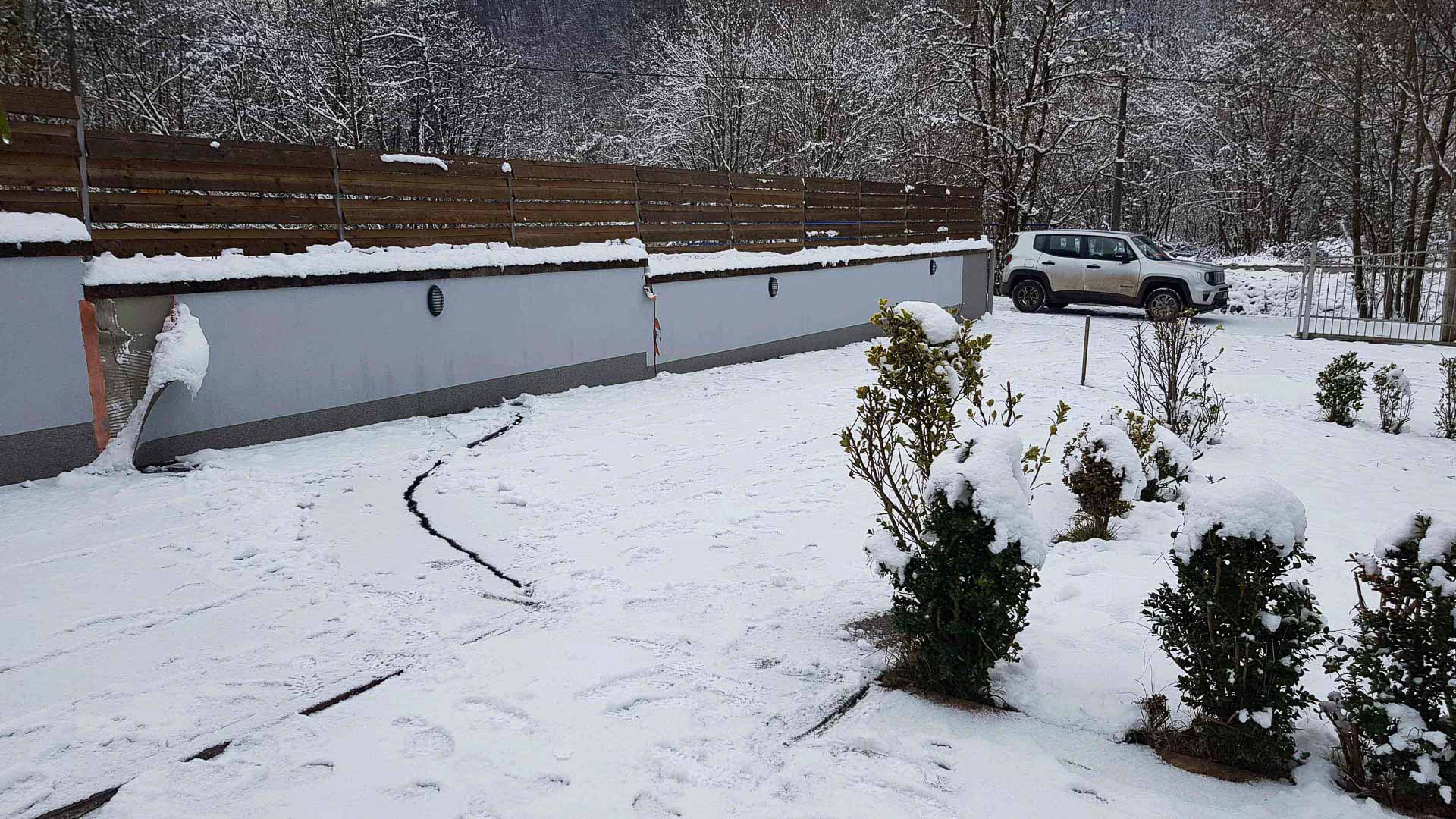

Supplement: Supplementary file 3 — Supplementary Information 3. [file 41598_2021_88378_MOESM3_ESM.zip › 26a (14-01-2021).jpg]

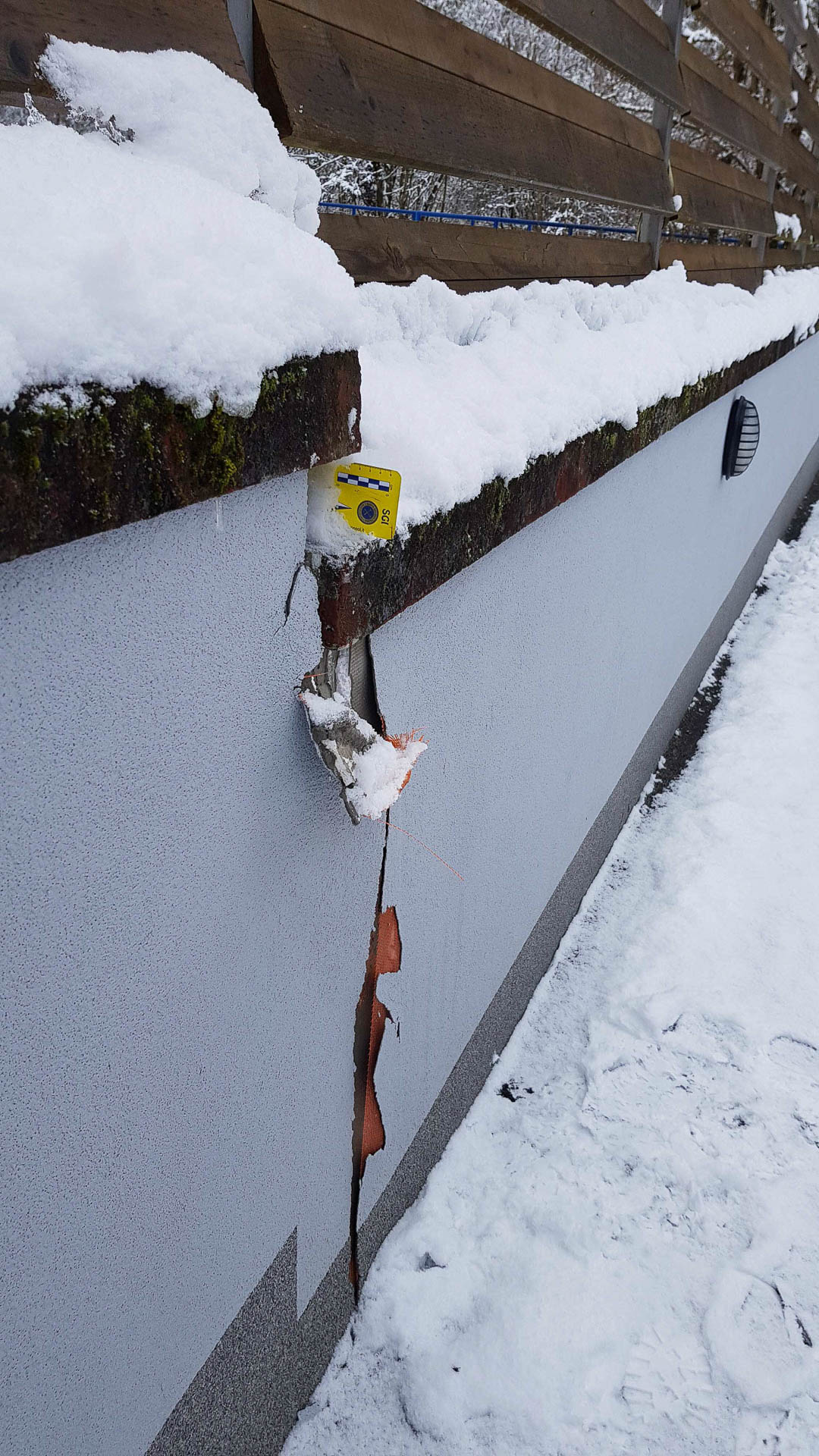

Supplement: Supplementary file 3 — Supplementary Information 3. [file 41598_2021_88378_MOESM3_ESM.zip › 26b (14-01-2021).jpg]

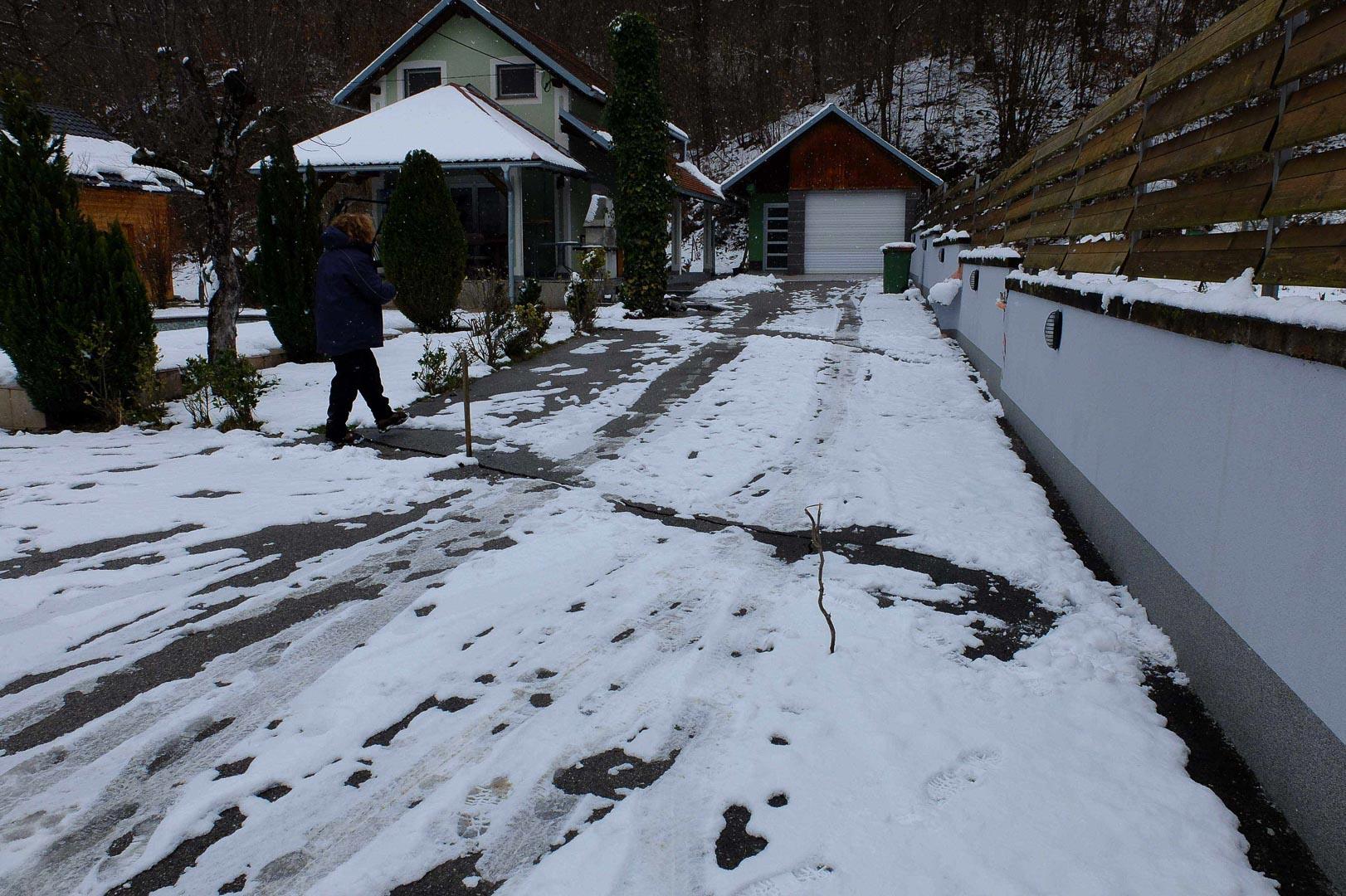

Supplement: Supplementary file 3 — Supplementary Information 3. [file 41598_2021_88378_MOESM3_ESM.zip › 26c (16-01-2021).jpg]

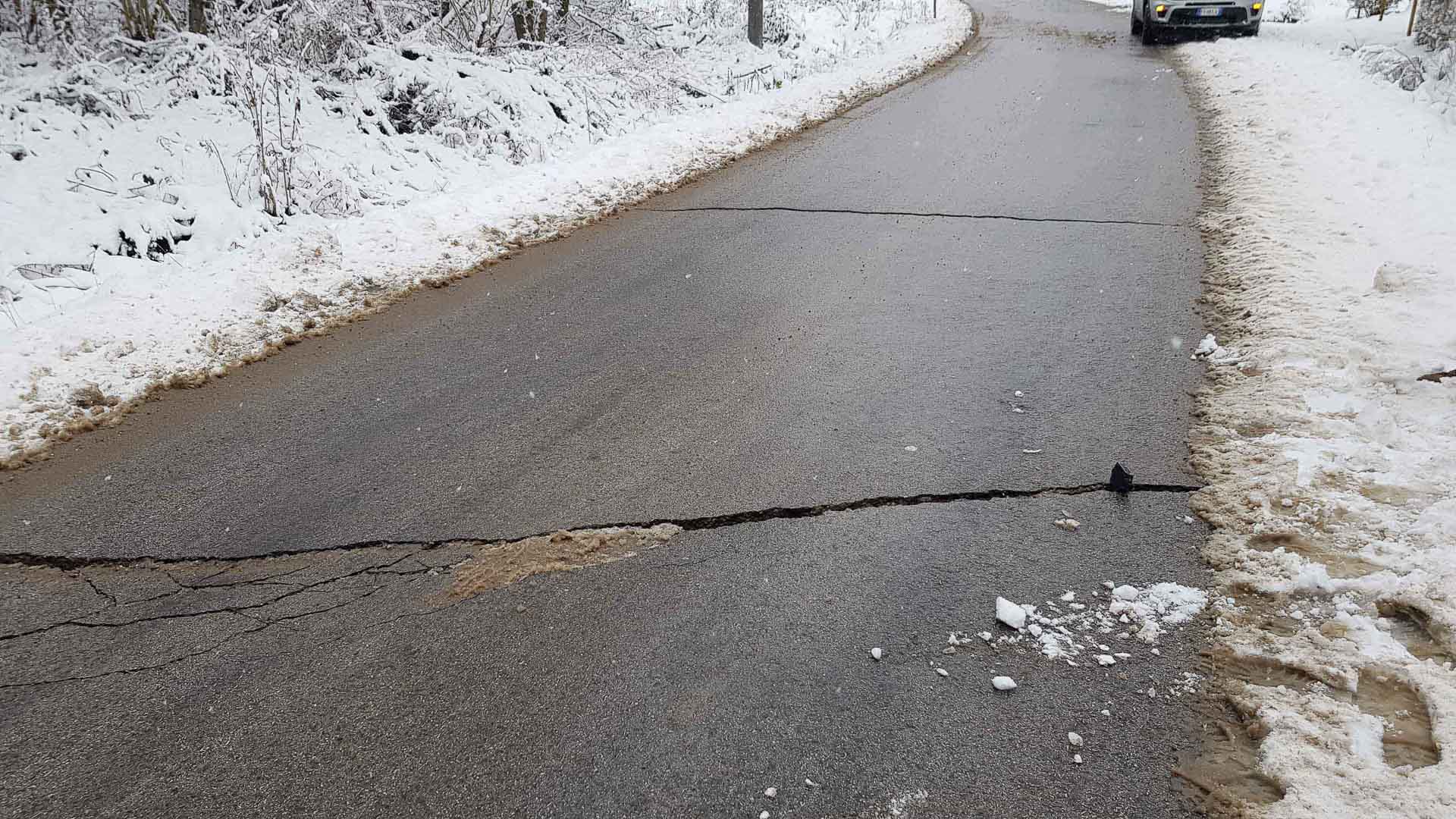

Supplement: Supplementary file 3 — Supplementary Information 3. [file 41598_2021_88378_MOESM3_ESM.zip › 28a (11-01-2021).jpg]

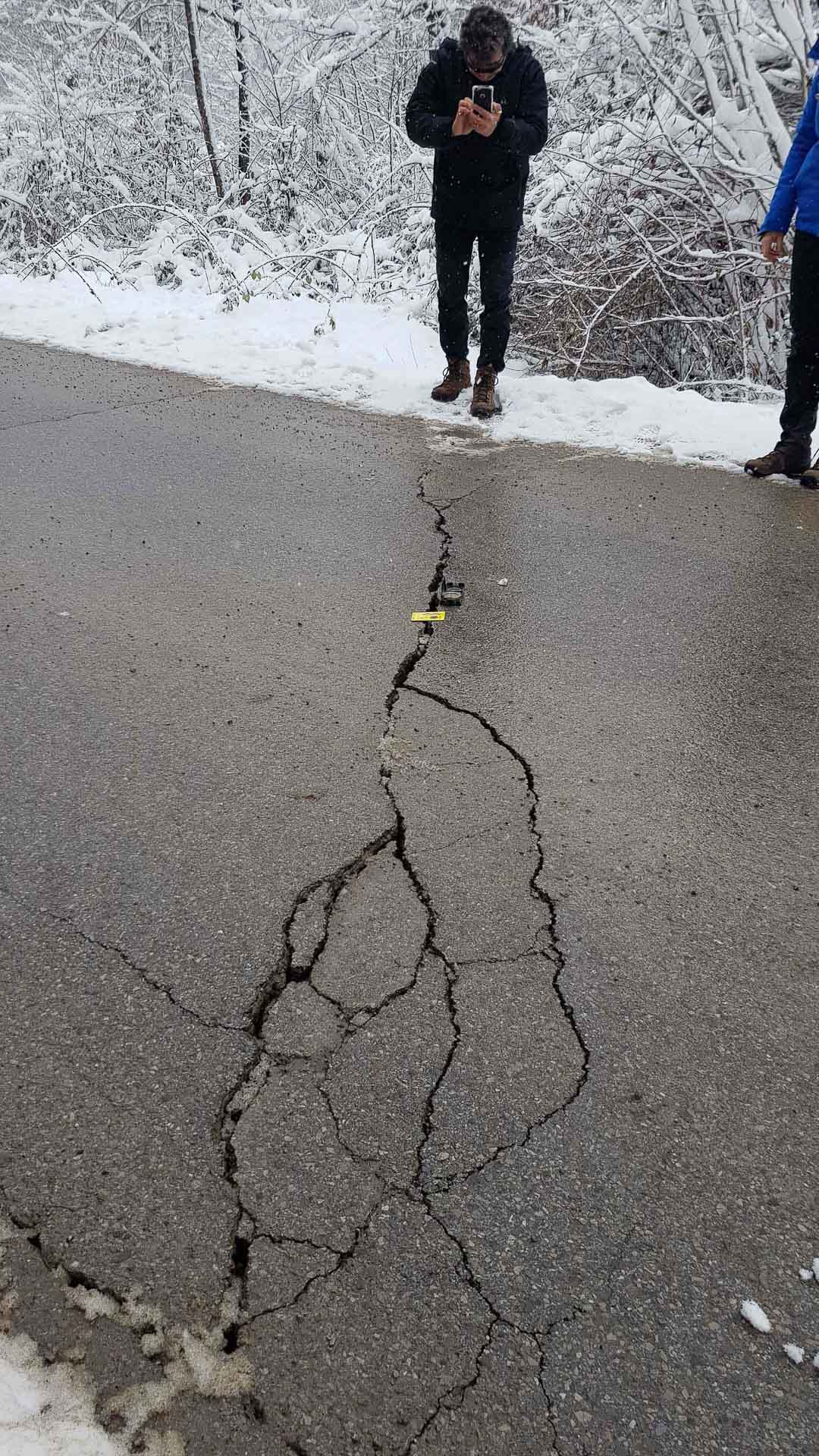

Supplement: Supplementary file 3 — Supplementary Information 3. [file 41598_2021_88378_MOESM3_ESM.zip › 28b (11-01-2021).jpg]

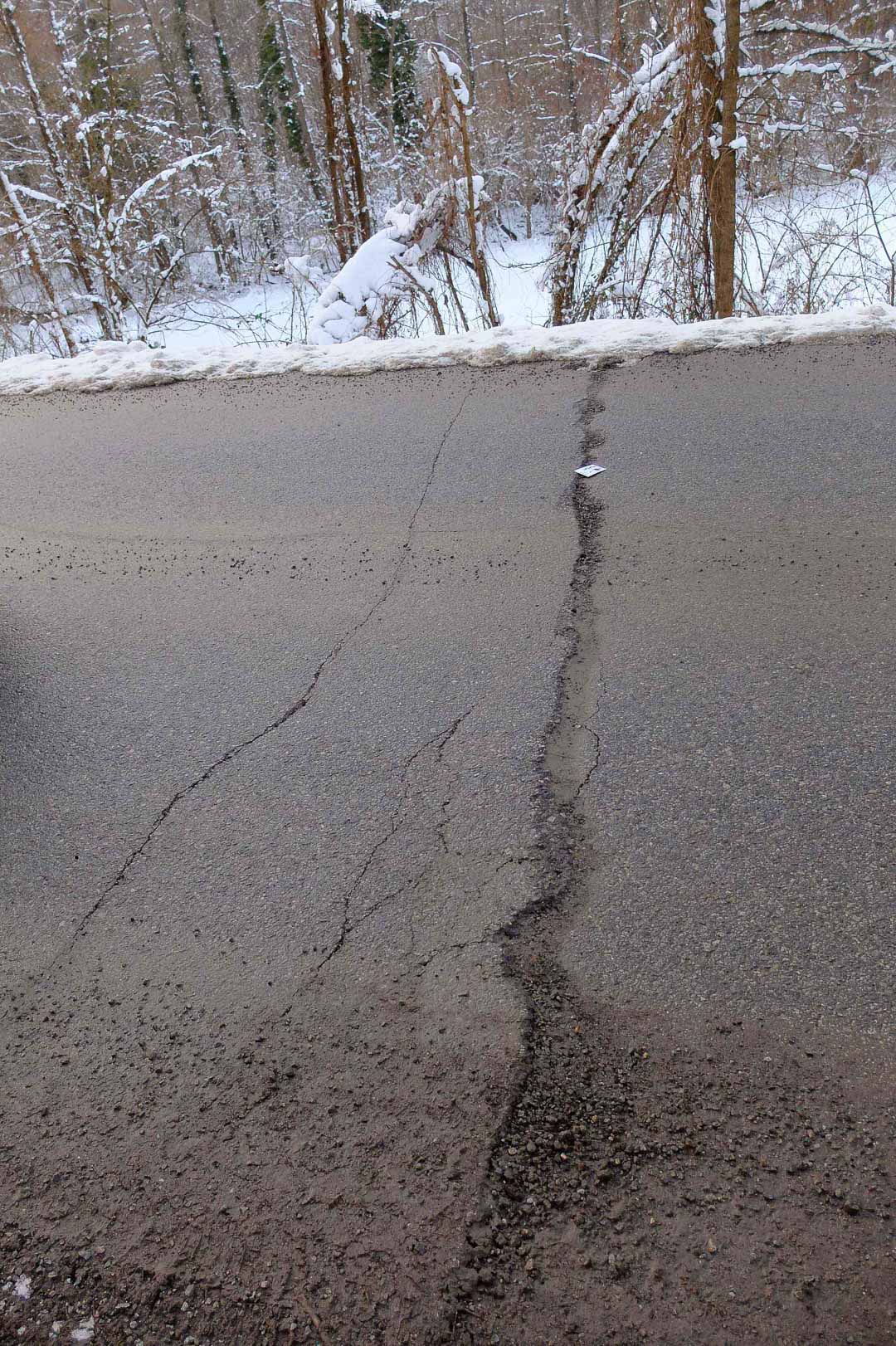

Supplement: Supplementary file 3 — Supplementary Information 3. [file 41598_2021_88378_MOESM3_ESM.zip › 31a (16-01-2021).jpg]

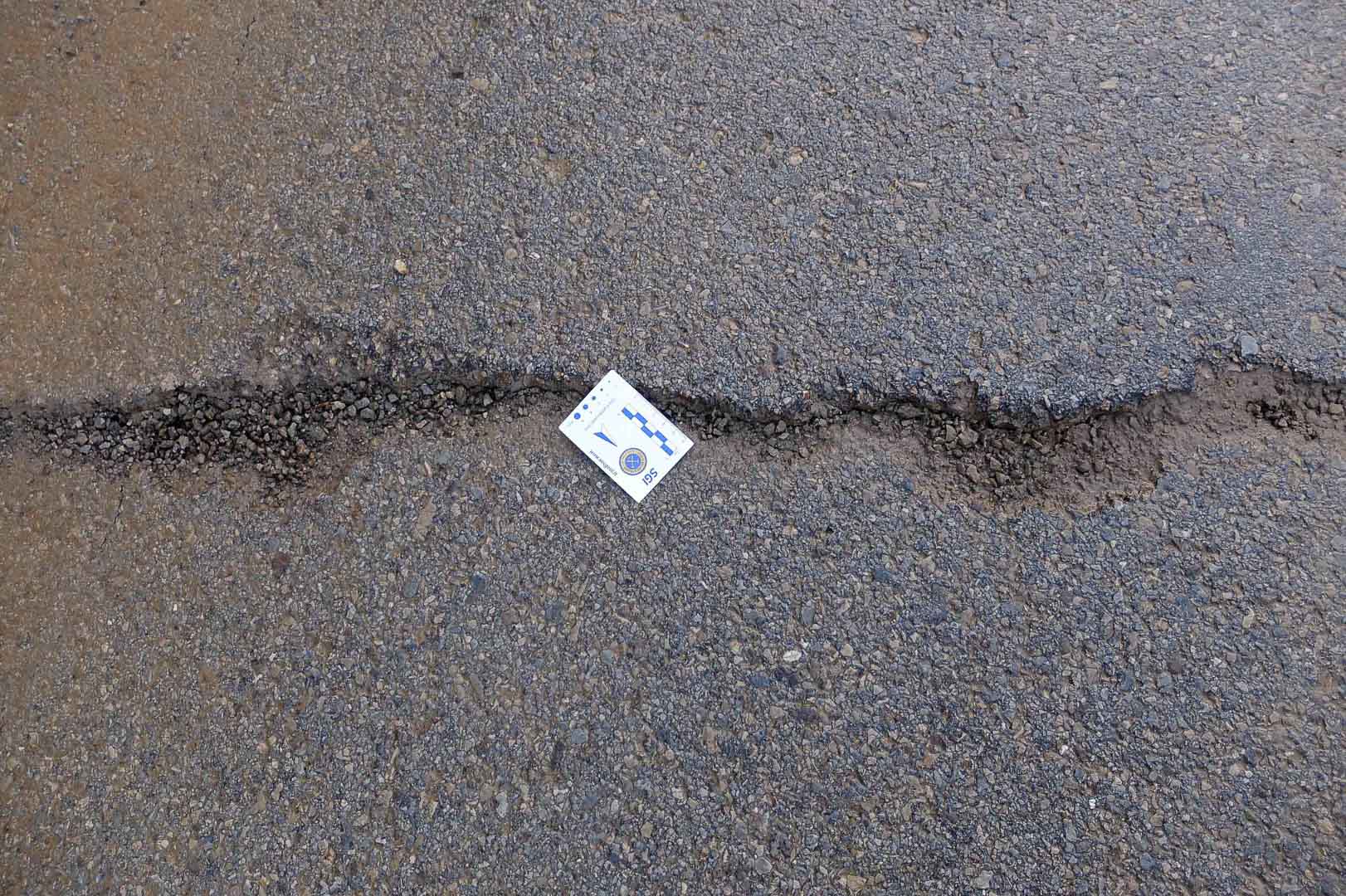

Supplement: Supplementary file 3 — Supplementary Information 3. [file 41598_2021_88378_MOESM3_ESM.zip › 31b (16-01-2021).jpg]

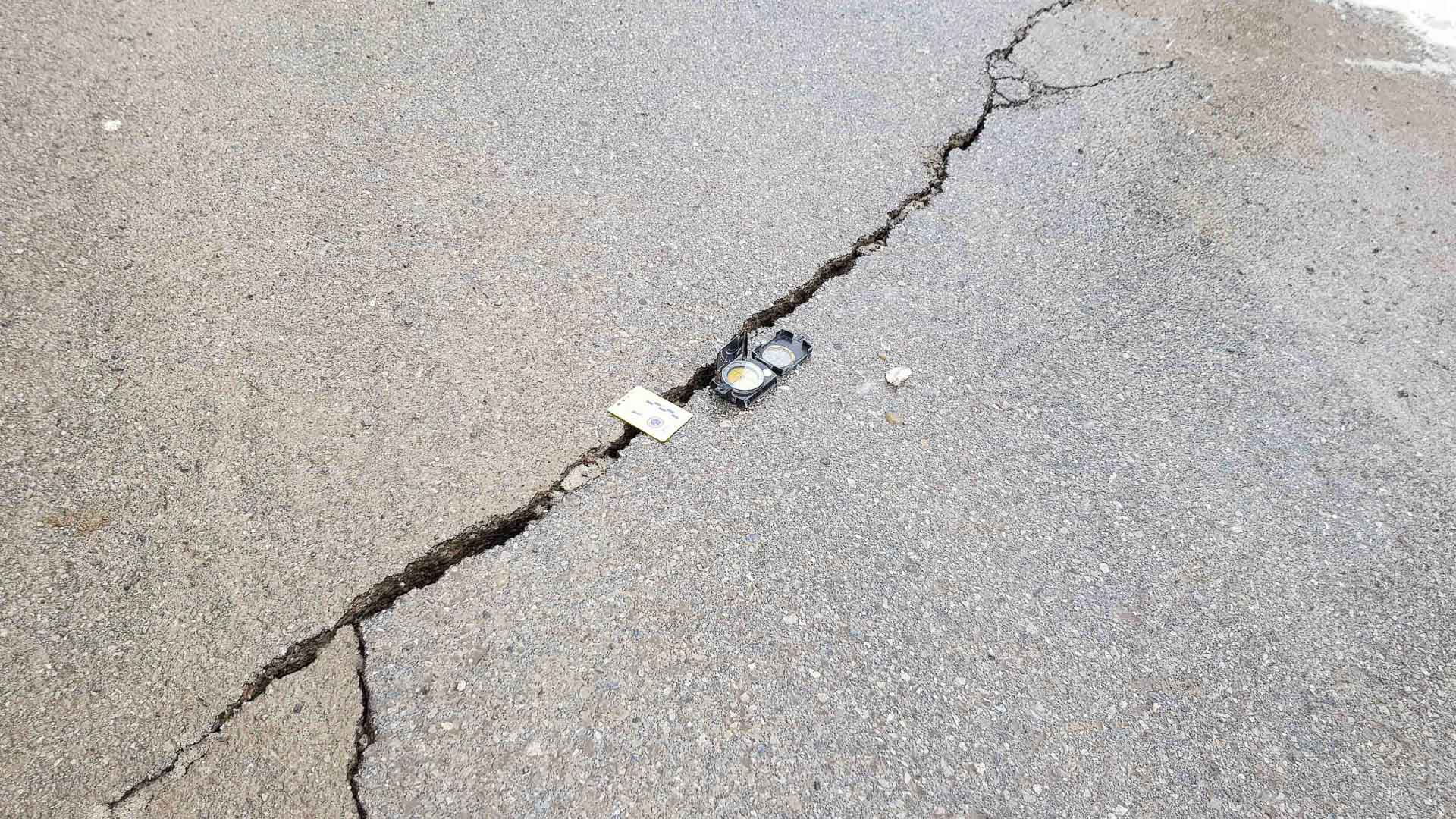

Supplement: Supplementary file 3 — Supplementary Information 3. [file 41598_2021_88378_MOESM3_ESM.zip › 33a (11-01-2021).jpg]

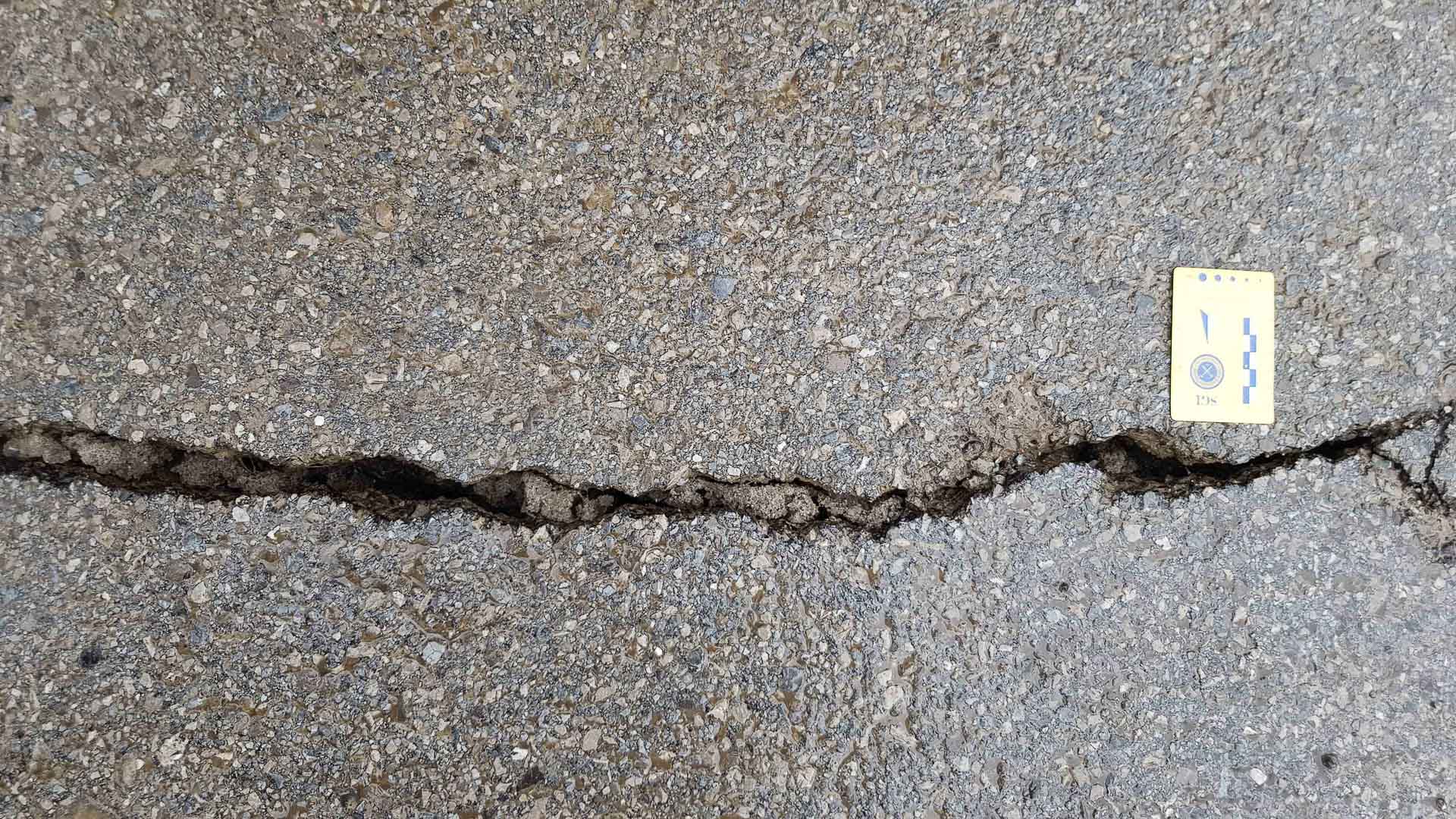

Supplement: Supplementary file 3 — Supplementary Information 3. [file 41598_2021_88378_MOESM3_ESM.zip › 33b (11-01-2021).jpg]

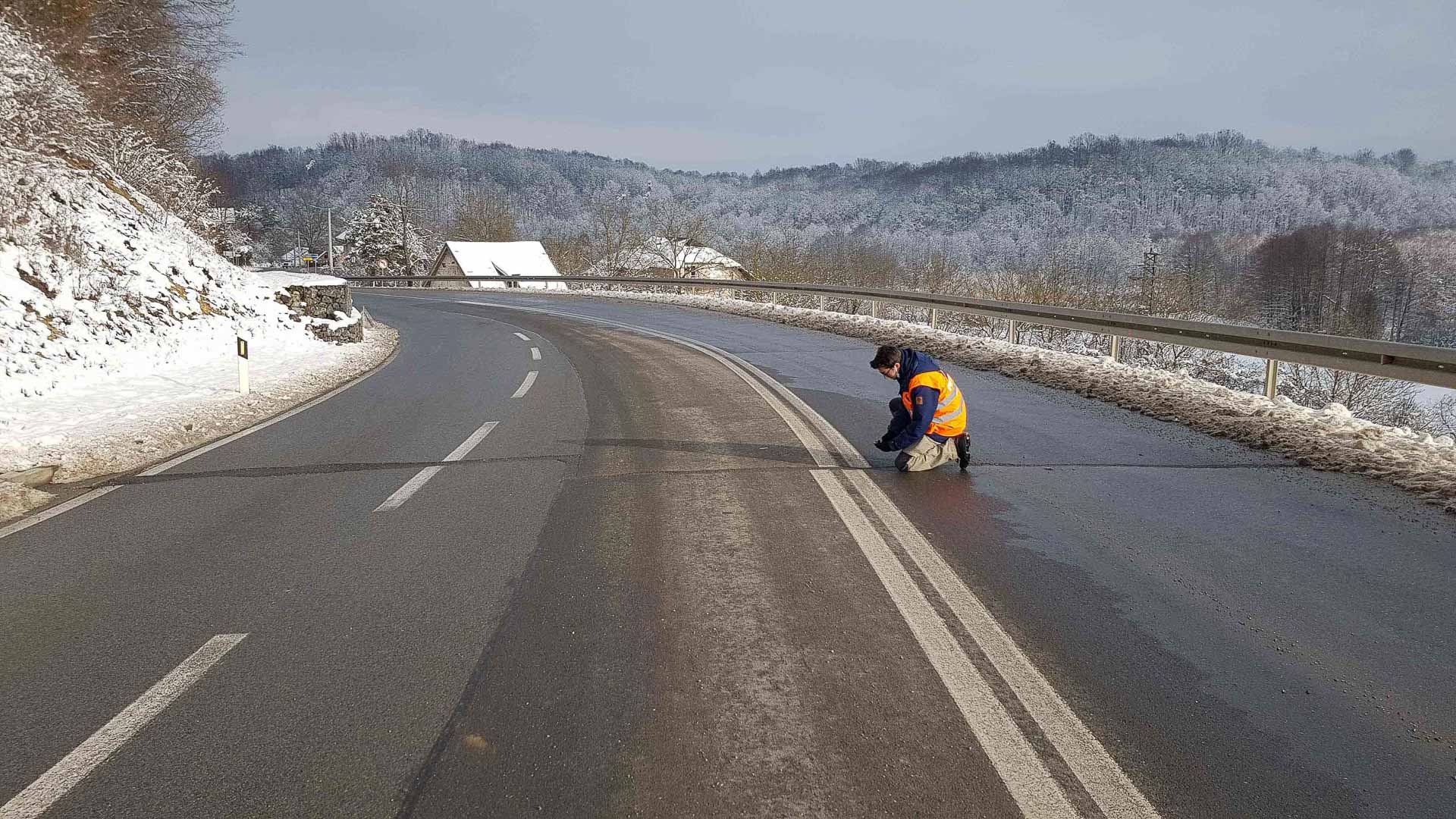

Supplement: Supplementary file 3 — Supplementary Information 3. [file 41598_2021_88378_MOESM3_ESM.zip › 3a (12-01-2021).jpg]

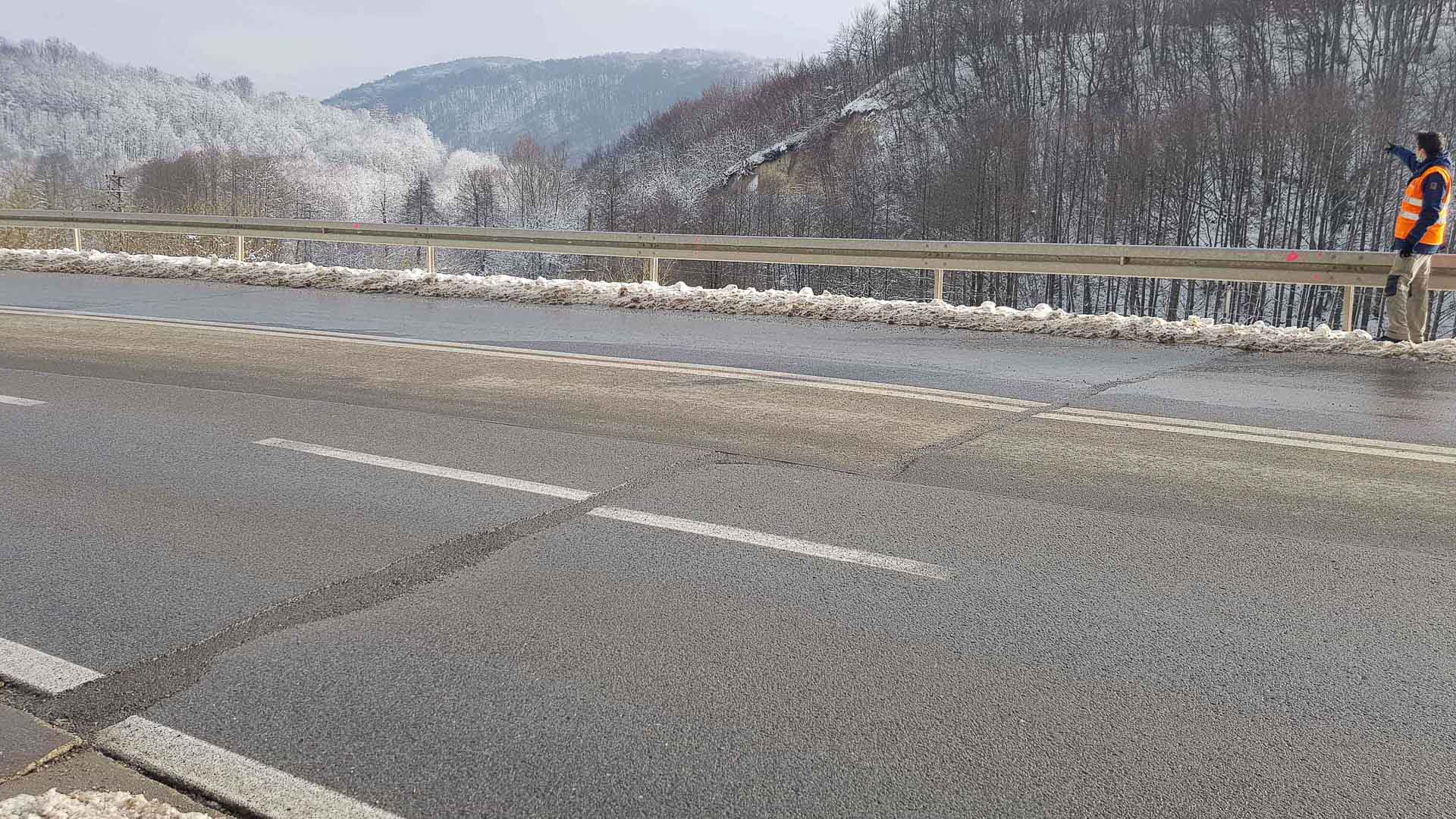

Supplement: Supplementary file 3 — Supplementary Information 3. [file 41598_2021_88378_MOESM3_ESM.zip › 3b (12-01-2021).jpg]

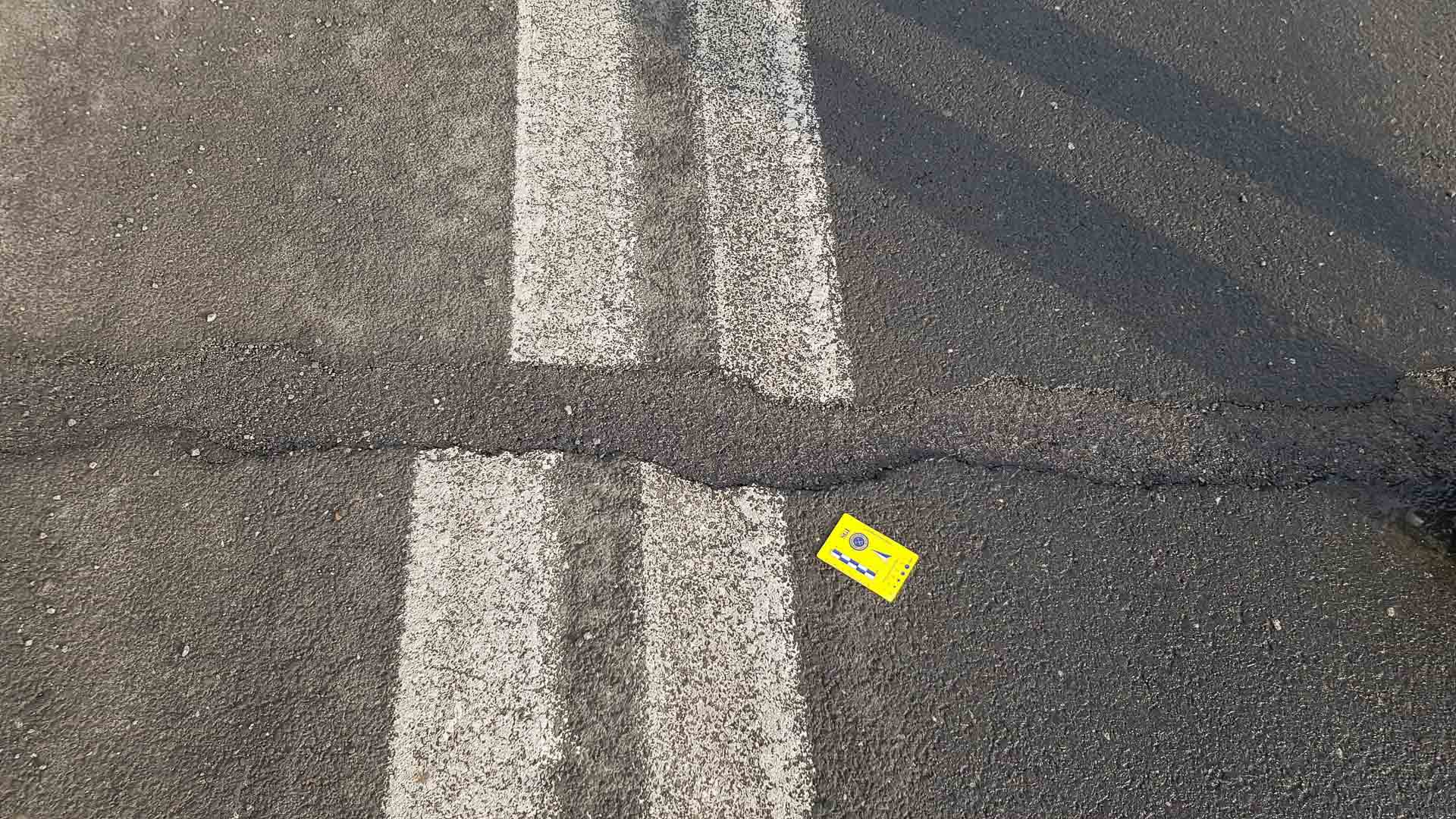

Supplement: Supplementary file 3 — Supplementary Information 3. [file 41598_2021_88378_MOESM3_ESM.zip › 3c (12-01-2021).jpg]

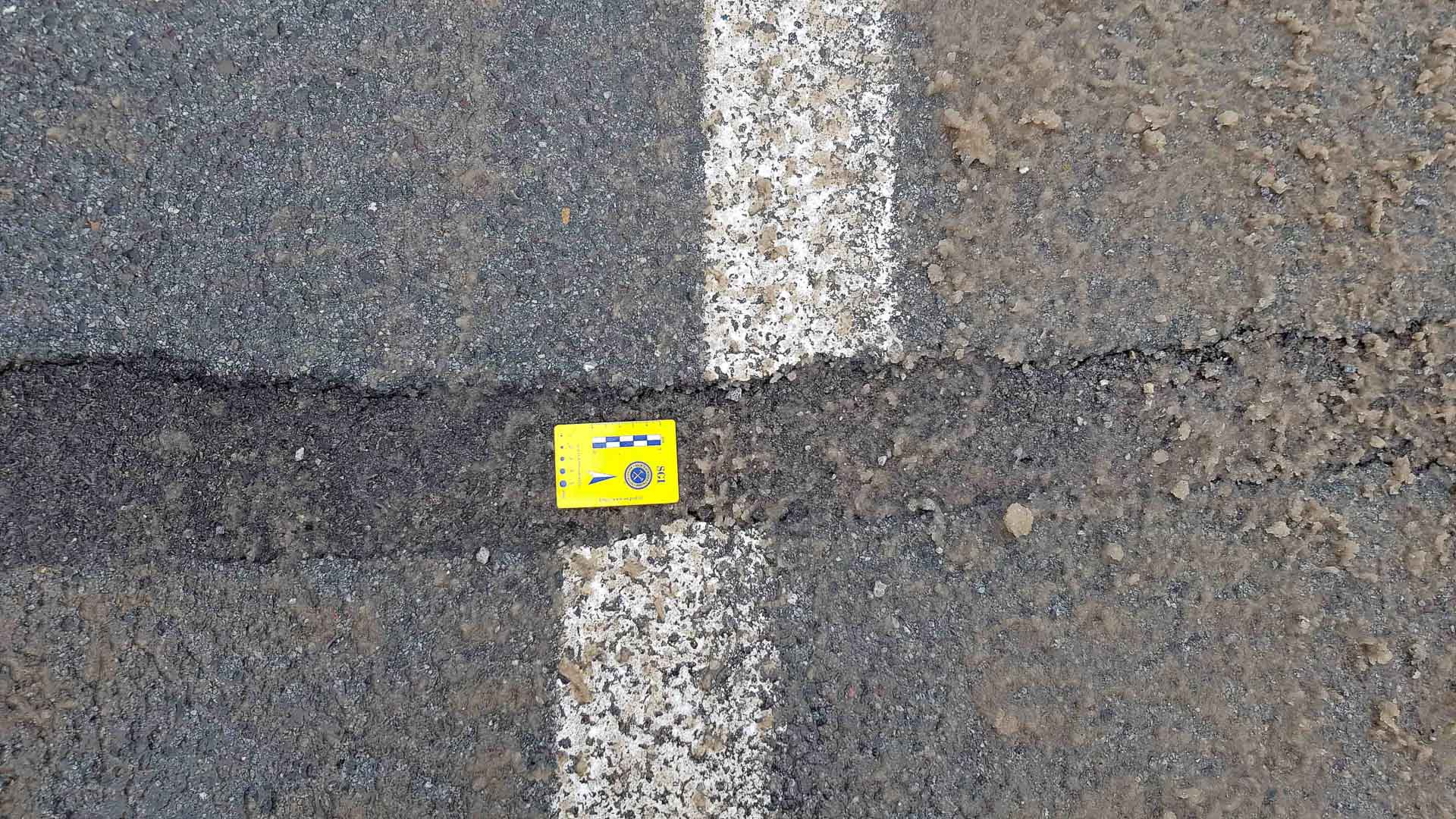

Supplement: Supplementary file 3 — Supplementary Information 3. [file 41598_2021_88378_MOESM3_ESM.zip › 3d (11-01-2021).jpg]

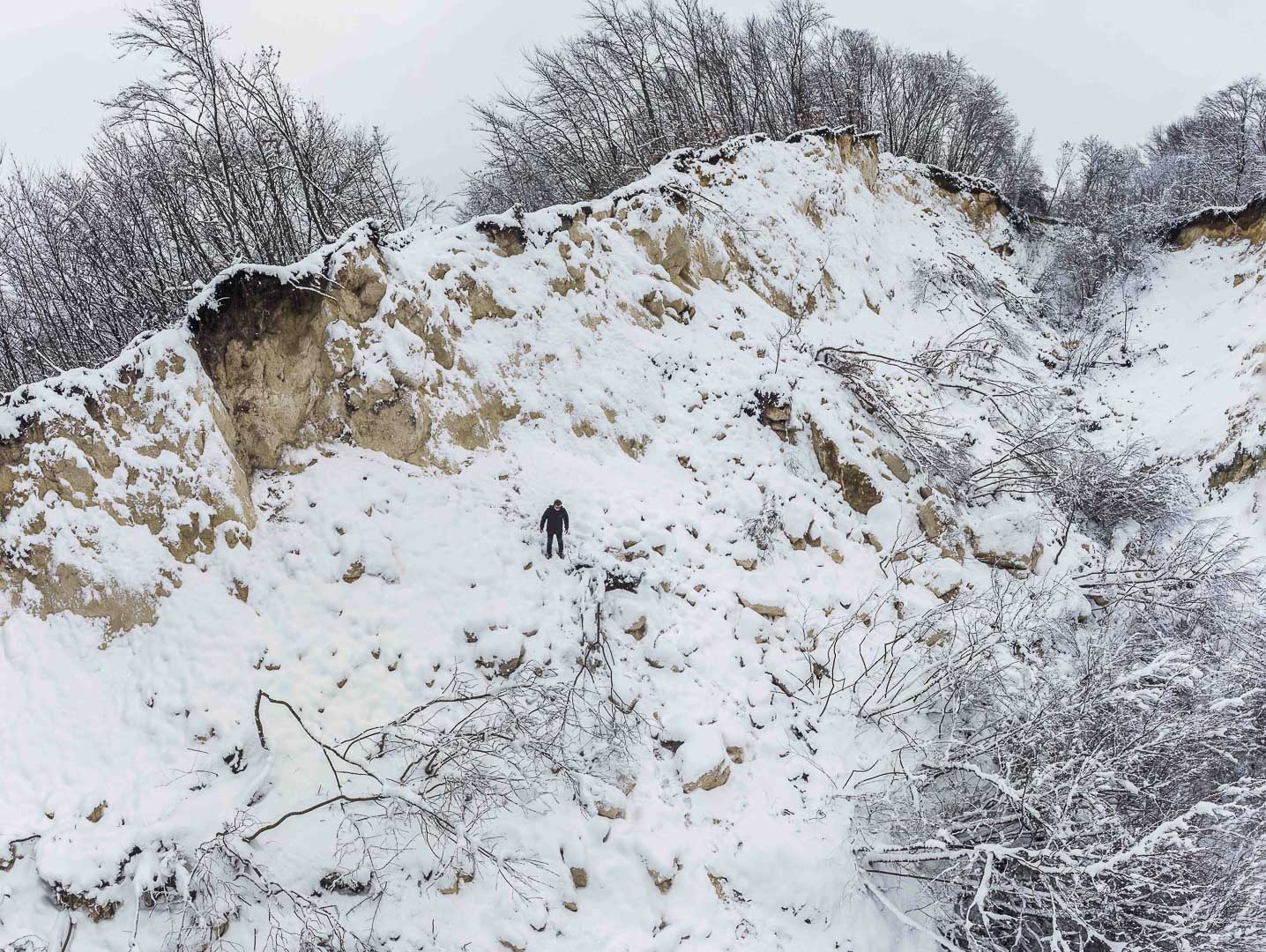

Supplement: Supplementary file 3 — Supplementary Information 3. [file 41598_2021_88378_MOESM3_ESM.zip › 64a (12-01-2021).jpg]

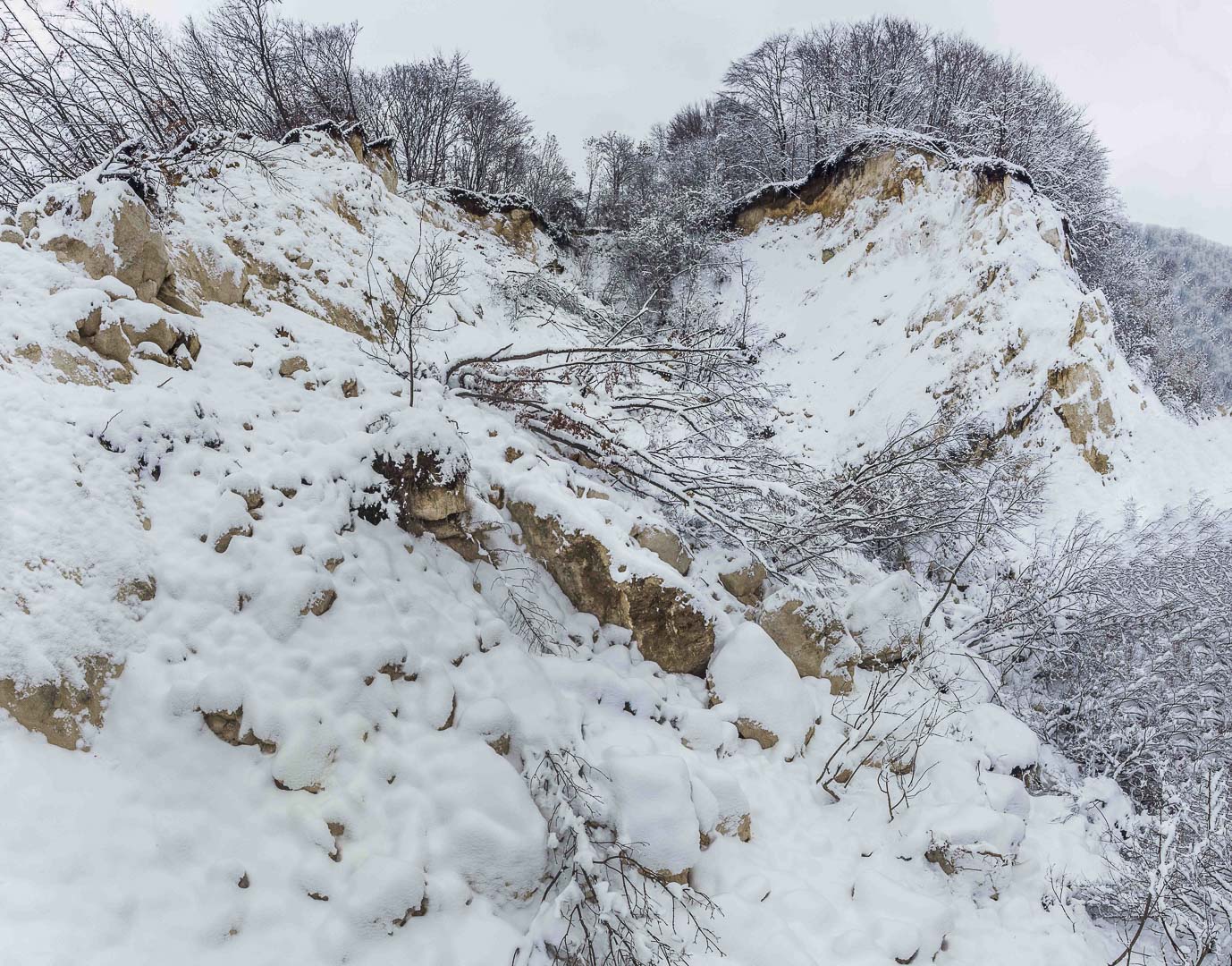

Supplement: Supplementary file 3 — Supplementary Information 3. [file 41598_2021_88378_MOESM3_ESM.zip › 64b (12-01-2021).jpg]

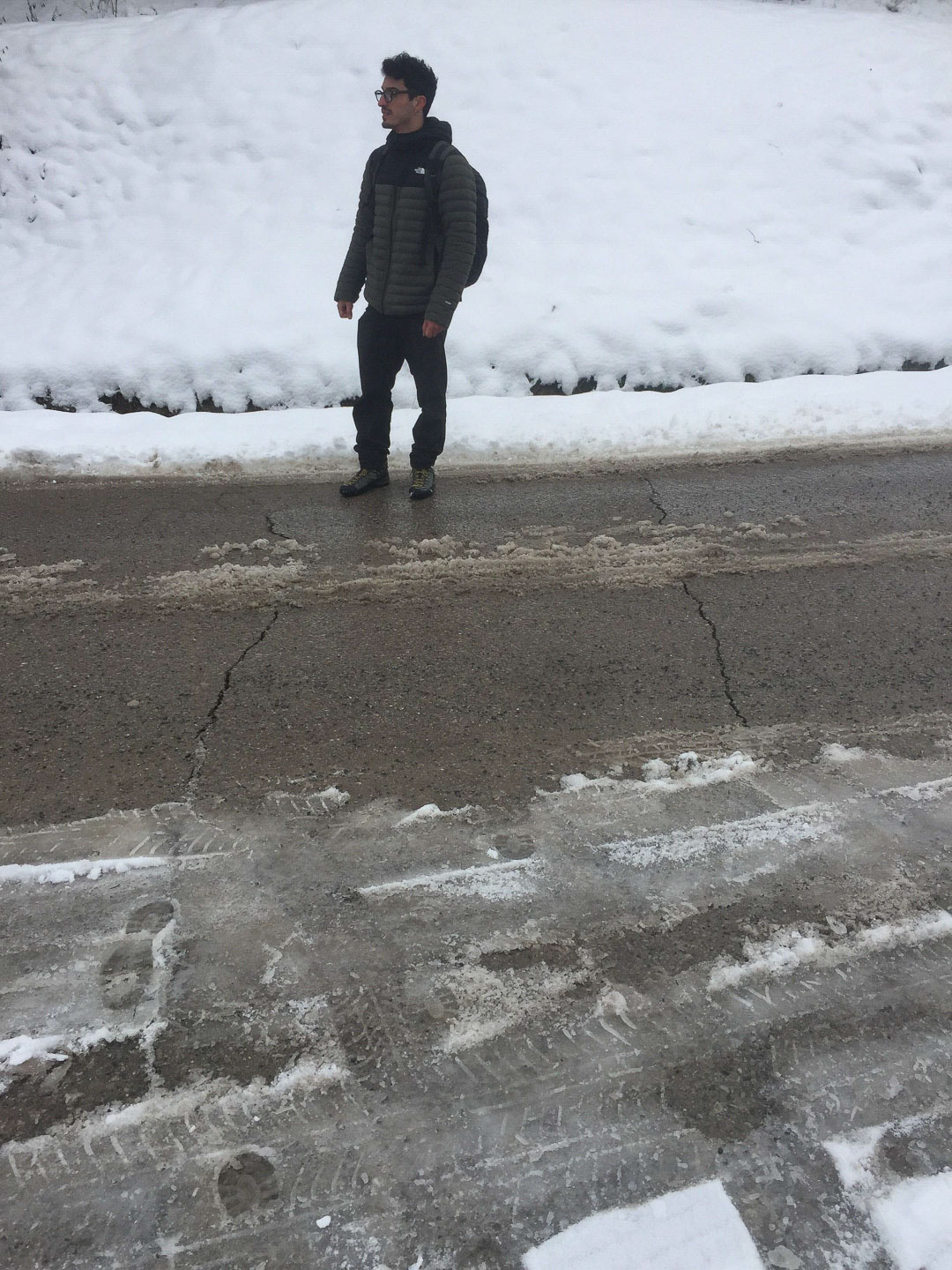

Supplement: Supplementary file 3 — Supplementary Information 3. [file 41598_2021_88378_MOESM3_ESM.zip › 73 (12-01-2021).jpg]

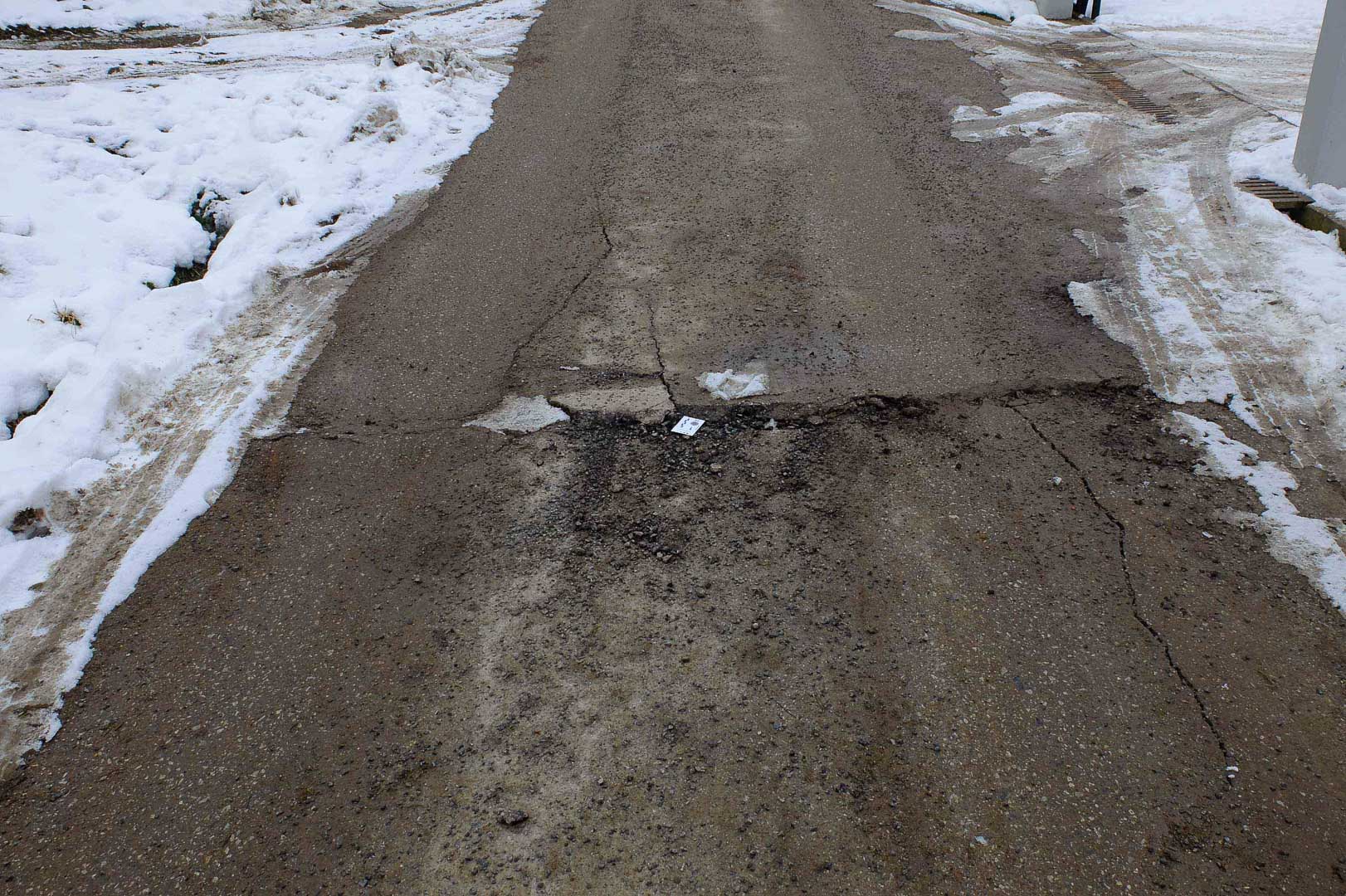

Supplement: Supplementary file 3 — Supplementary Information 3. [file 41598_2021_88378_MOESM3_ESM.zip › 73a (17-01-2021).jpg]

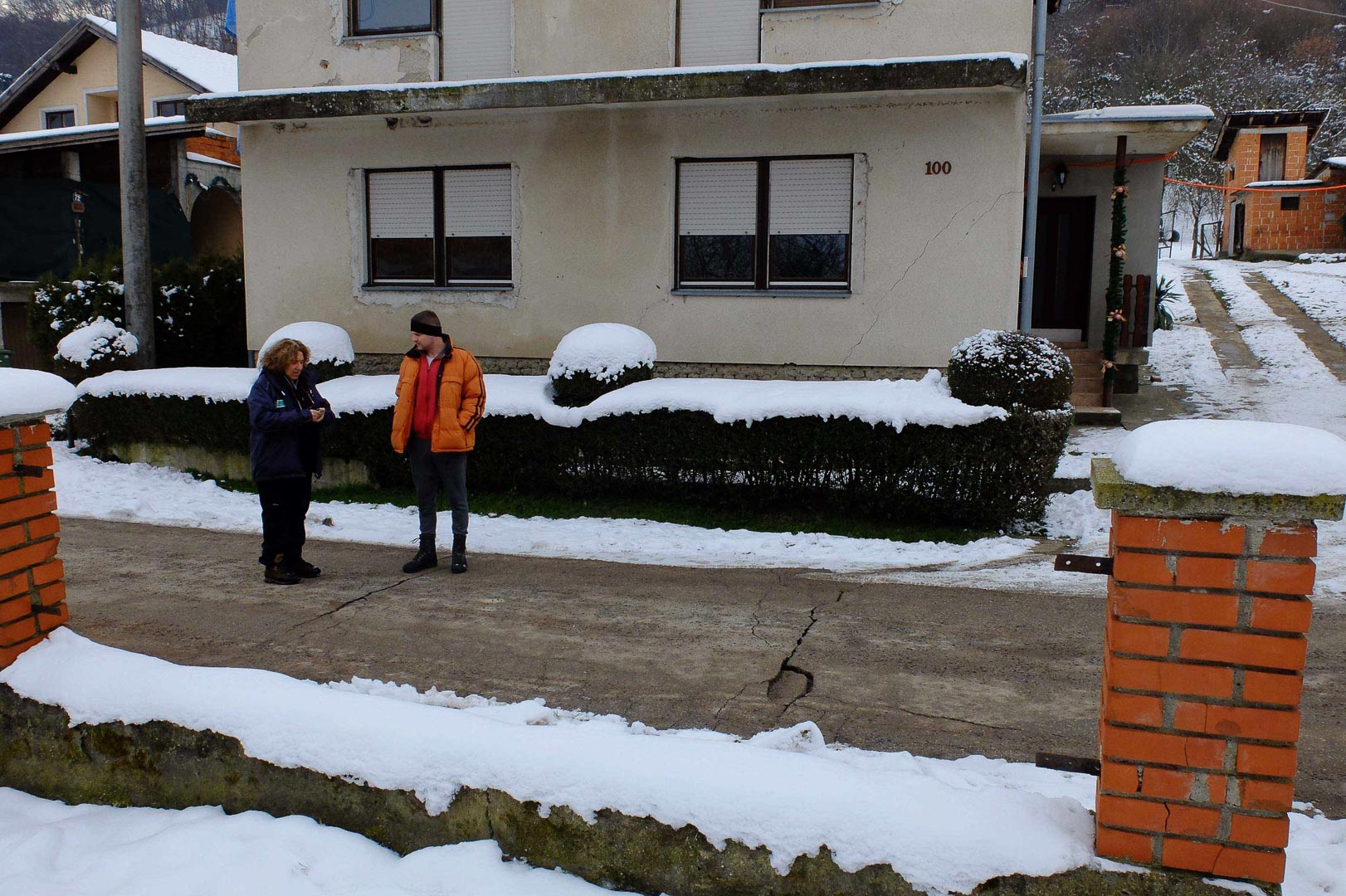

Supplement: Supplementary file 3 — Supplementary Information 3. [file 41598_2021_88378_MOESM3_ESM.zip › 73b (17-01-2021).jpg]

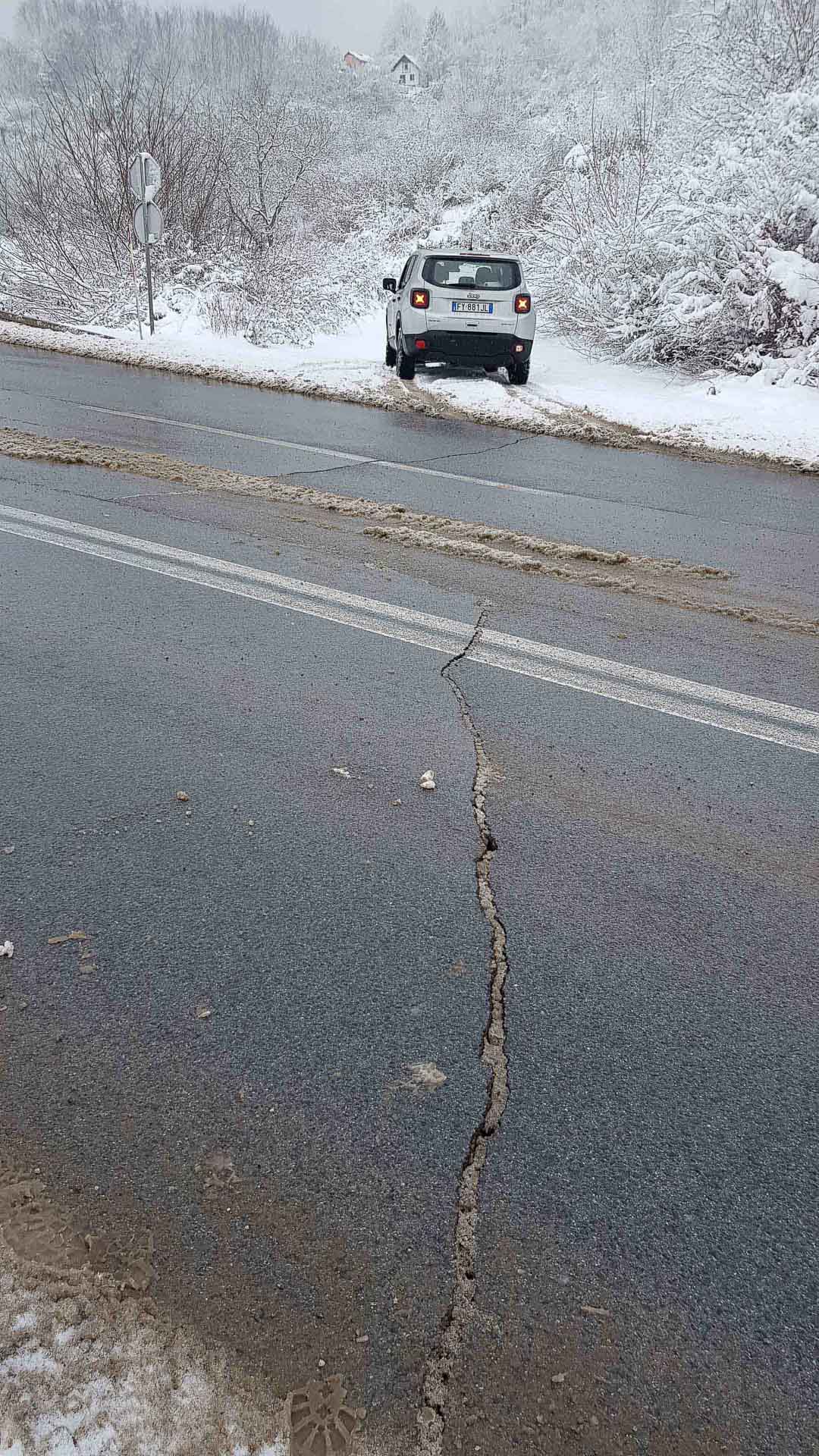

Supplement: Supplementary file 3 — Supplementary Information 3. [file 41598_2021_88378_MOESM3_ESM.zip › 8 (11-01-2021).jpg]

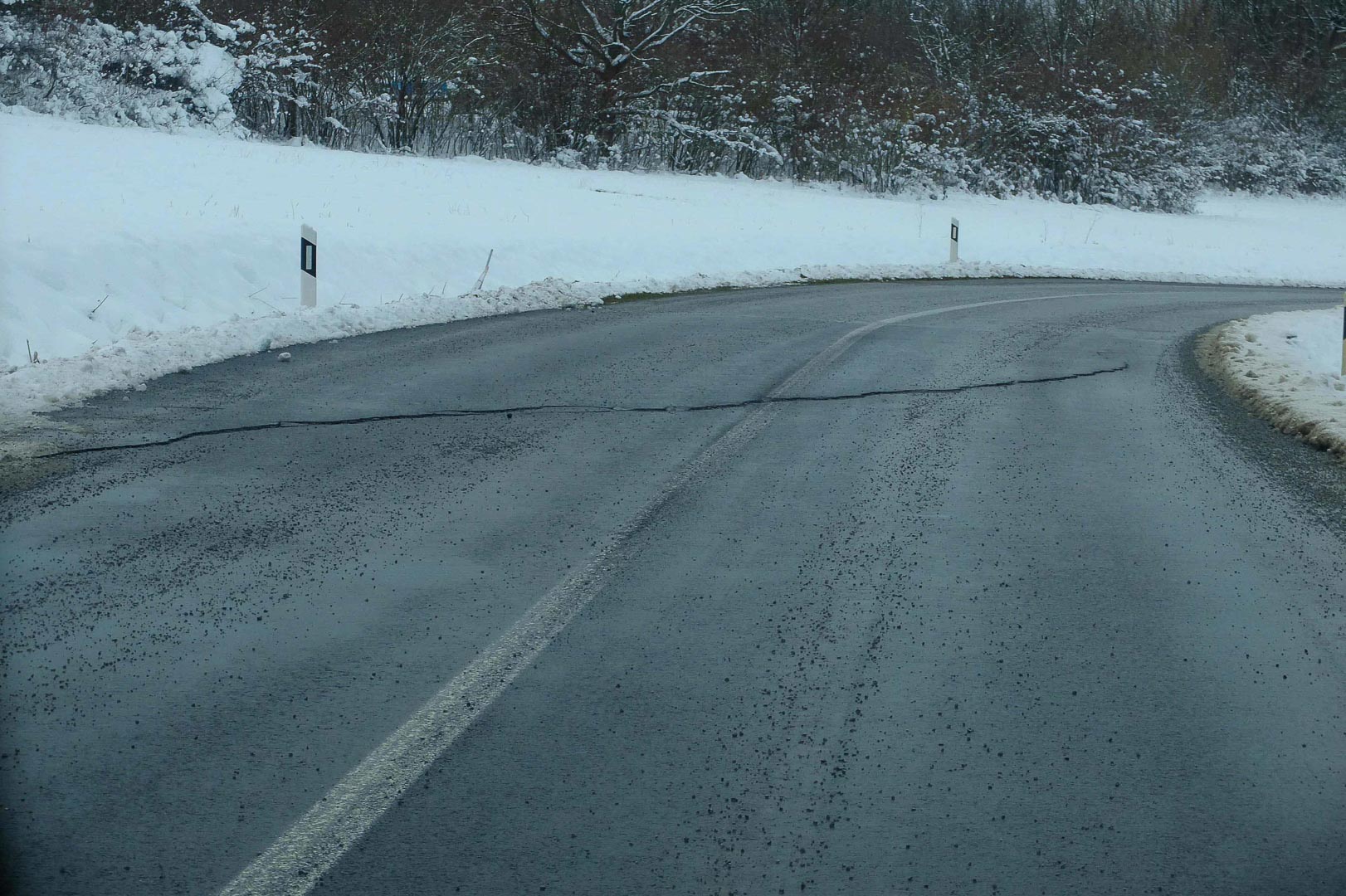

Supplement: Supplementary file 3 — Supplementary Information 3. [file 41598_2021_88378_MOESM3_ESM.zip › 82 (14-01-2021).jpg]
